# Characterization of the synthetic cannabinoid MDMB-CHMCZCA

**DOI:** 10.3762/bjoc.12.279

**Published:** 2016-12-21

**Authors:** Carina Weber, Stefan Pusch, Dieter Schollmeyer, Sascha Münster-Müller, Michael Pütz, Till Opatz

**Affiliations:** 1 Johannes Gutenberg University Mainz, Institute of Organic Chemistry, Duesbergweg 10–14, 55128 Mainz, Germany; 2 Bundeskriminalamt – Federal Criminal Police Office (BKA), Forensic Science Institute, KT 45 – Toxicology, Äppelallee 45, 65203 Wiesbaden, Germany

**Keywords:** chiral HPLC, ECD spectroscopy, NPS, synthetic cannabinoids, VCD spectroscopy

## Abstract

The synthetic cannabinoid MDMB-CHMCZCA was characterized by various spectroscopic techniques including NMR spectroscopy and tandem mass spectrometry. The synthetic sample was found to be of *S*-configuration by VCD spectroscopy and comparison of the data with DFT calculations, while ECD spectroscopy was found to be inconclusive in this case. The enantiomeric purity of samples from test purchases and police seizures was assessed by a self-developed chiral HPLC method.

## Introduction

**Figure 1:**
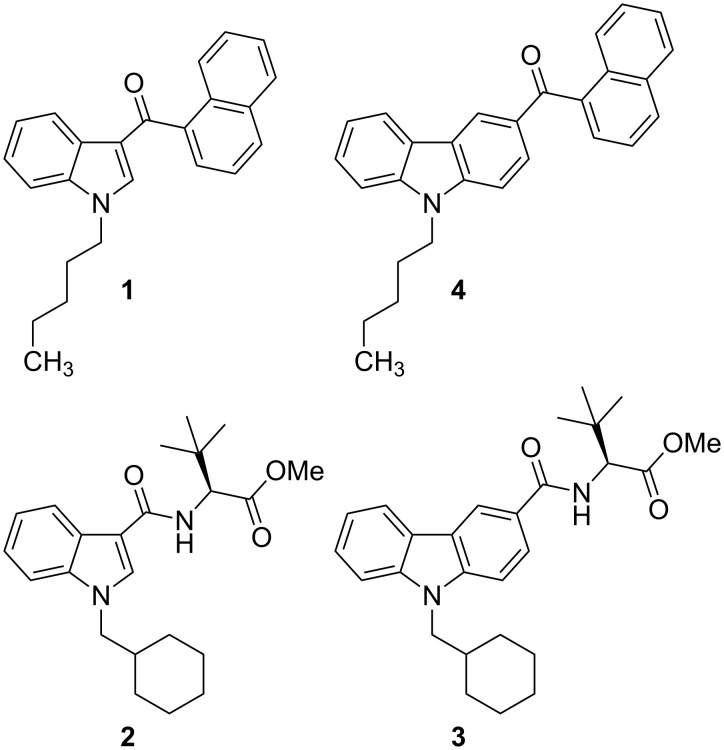
Synthetic cannabinoids JWH-018 (**1**), MDMB-CHMICA (**2**), MDMB-CHMCZCA (**3**), and EG-018 (**4**).

Starting in 2008 with the appearance of “Spice products”, herbal mixtures spiked with synthetic cannabinoid receptor agonists began to spread on the international drug market [1–2]. The cannabimimetic substances are the psychoactive ingredients of those mixtures and serve as a marijuana substitute with the intention to circumvent the narcotics regulations. Within the past years, the number of registered new psychoactive substances (NPS) has increased tremendously and currently lies around 600, 98 of which have only appeared in 2015 [3]. In response, the European authorities collaborate closely in terms of information exchange, risk assessment and control of NPS [4]. The first substance reported to the European Early Warning System (EWS) of the European Monitoring Centre for Drugs and Drug Addiction (EMCDDA) in this context was JWH-018 (**1**, Figure 1), originally developed for medical applications [5].

More recently, the synthetic CB_1_ receptor ligand MDMB-CHMICA (**2**) attracted public and regulatory attention as several cases of severe intoxication and death were linked to this compound [6–7]. Up to November 2016, MDMB-CHMICA is controlled through legislation in 17 of the 30 member states of the EMCDDA [8]. It contains an *N*-alkylated indole core structure with carboxamide substituent in C-3 position, linked to an *tert*-leucine methyl ester and in previous studies, the amino acid was shown to be *S*-configurated [9].

Lately, the new synthetic cannabinoid MDMB-CHMCZCA (**3**) was found on the drug market in the form of pure powder samples or as active substance in herbal mixtures (“Spice products”). Between October 7th, 2015 and October 19th, 2016, seven EU member states reported seizures or samples from test purchases in internet shops containing MDMB-CHMCZCA to the EWS of the EMCDDA. The first reported occurrence was a seizure by Swedish customs in September 2015 [10]. The structure of MDMB-CHMCZCA, a semi-systematic name for **m**ethyl **d**i**m**ethyl**b**utanoate-**c**yclo**h**exyl**m**ethyl**c**arba**z**ole**c**arbox**a**mide, is related to the structure of MDMB-CHMICA, but contains an *N*-alkylated carbazole instead of an indole core. Carbazoles are the core structures of an emerging group of cannabimimetics [11–12]. Several *N*-alkylated carbazole-3-carboxamides were patented by Diaz, Diaz, and Petrov in 2012 as tricyclic cannabinoid receptor modulators, explored against neuropathic pain [13]. Nevertheless, only few carbazole derivatives have appeared as NPS on the drug market so far, one of which is EG-018 (**4**), the carbazole analogue to JWH-018 (**1**) [14–15]. Another example is EG-2201, the carbazole analoge to AM-2201, which is a derivative of JWH-018 with a 5-fluoro substitutent in the *N*-pentyl chain.

As part of the ongoing EU-project “SPICE profiling”, MDMB-CHMCZCA samples from test purchases in online shops and police seizures were analyzed to obtain analytical data and chemical properties. Another aim was to assess the absolute configuration and optical purity of the selected product samples. For this purpose, we used analytical methods such as NMR, tandem mass spectrometry, vibrational and electronic circular dichroism spectroscopy (VCD and ECD), as well as chiral HPLC.

## Results and Discussion

**Figure 2:**
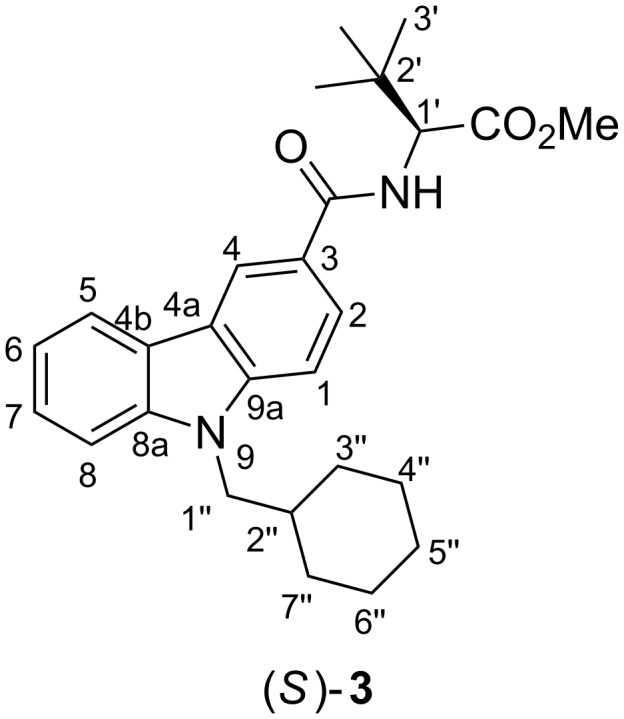
Molecular structure of (*S*)-MDMB-CHMCZCA (**3**) with numbering scheme.

Pure MDMB-CHMCZCA (**3**) was obtained as a so-called “research chemical” (RC) from an online RC shop (test purchase 1, December 2015), a colorless powder with the following analytical key data, supporting the structure from Figure 2.

**Analytical properties of MDMB-CHMCZCA (methyl *N*-{[9-(cyclohexylmethyl)-9*H*-carbazol-3-yl]carbonyl}-3-methyl-L-valinate). **^1^H NMR, COSY, NOESY (600 MHz, CDCl_3_) δ_H_/ppm 8.58 (d, *J* = 1.7 Hz, 1H, H-4), 8.17–8.15 (m, 1H, H-5), 7.92 (dd, *J* = 8.6 Hz, 1.7 Hz, 1H, H-2), 7.52–7.49 (m, 1H, H-7), 7.44–7.42 (m, 1H, H-8), 7.30–7.28 (m, 1H, H-6), 6.77 (d, *J* = 9.3 Hz, 1H, CON*H*), 4.80 (d, *J* = 9.3 Hz, 1H, H-1'), 4.14 (d, *J* = 7.4 Hz, 2H, H_2_-1''), 3.78 (s, 3H, OMe), 2.02–1.99 (m, 1H, H-2''), 1.72–1.64 (m, 5H, H-3_a_'', H-4_a_'', H-5_a_'', H-6_a_'', H-7_a_''), 1.18–1.10 (m, 5H, H-3_b_'', H-4_b_'', H-5_b_'', H-6_b_'', H-7_b_''), 1.10 (s, 9H, C(C*H*_3_)_3_); ^13^C NMR, HSQC, HMBC (151 MHz, CDCl_3_) δ_C_/ppm 172.7 (*C*O_2_Me), 167.9 (*C*ONH), 143.0 (C-9a), 141.6 (C-8a), 126.4 (C-7), 124.7 (C-2, C-3 overlapping), 122.8 (C-4b), 122.7 (C-4a), 120.8 (C-5), 120.1 (C-4), 119.7 (C-6), 109.6 (C-8), 109.9 (C-1), 60.4 (C-1'), 52.1 (O*C*H_3_), 49.9 (C-1''), 38.3 (C-2''), 35.4 (C-2'), 31.6 (C-3'', C-7''), 26.9 (C(*C*H_3_)_3_), 26.4 (C-5''), 25.9 (C-4'', C-6''); [^1^H,^15^N]-HSQC, [^1^H,^15^N]-HMBC (600 MHz, CDCl_3_) δ_N_/ppm 120.9 (N-9), 106.3 (CO*N*H); mp 171.5–172.4 °C; ESIMS *m*/*z*: 457.3 (19.9%, [M + Na]^+^), 435.4 (100%, [M + H]^+^), 290.3 (6.3%, fragment); HRMS-ESI *m*/*z*: calculated for [C_27_H_35_N_2_O_3_ + H]^+^, 435.2648; found, 435.2654; [α]_D_^25^ +38.6° (*c* 0.87, CDCl_3_).

**Figure 3:**
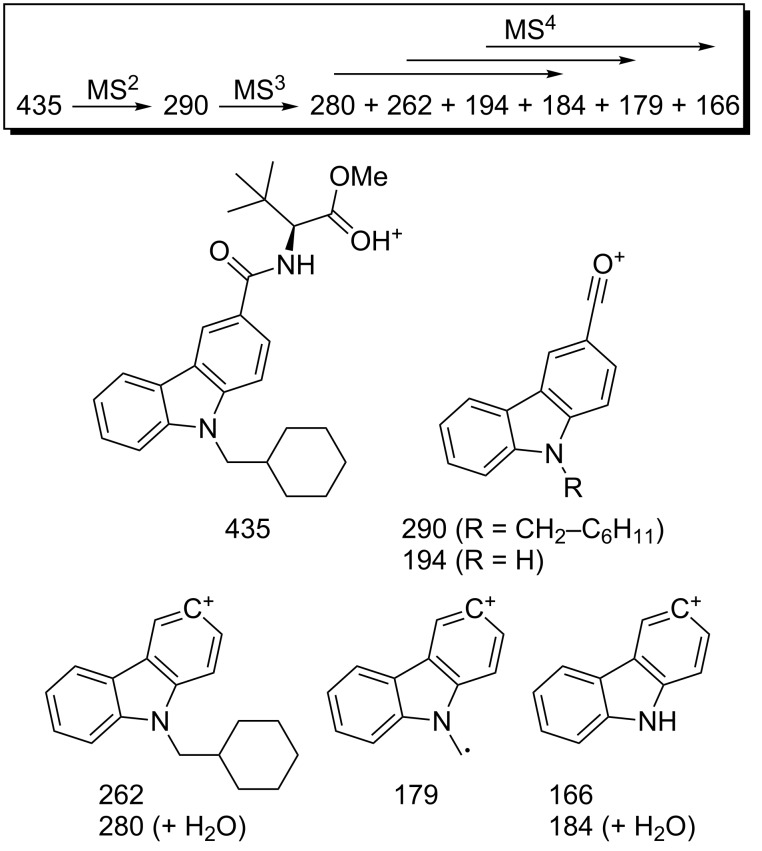
ESI-MS*^n^* pattern of **3** (*m*/*z* values) with probable fragment ion structures.

A small impurity in the sample appears in the COSY experiment (δ_H_ = 3.09 and 1.40 ppm, see the Supporting Information File 1 for details). This is most probably a triethylammonium salt resulting from the use of triethylamine as a base during the preparation of the material. The ESI-MS*^n^* fragmentation pathway of **3** is shown in Figure 3 and starts with cleavage of the amide, followed by loss of CO and/or a cyclohexyl radical or methylenecyclohexane.

**Figure 4:**
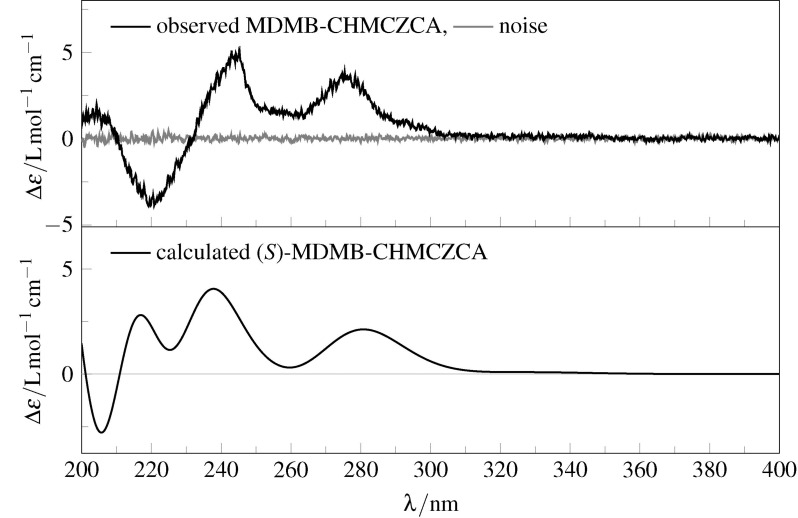
Observed (top) and calculated (bottom) ECD spectra for (*S*)-**3** in acetonitrile, theory level: TD-B3LYP/6-311++G**/IEFPCM//B3PW91/6-311G**/IEFPCM.

As MDMB-CHMCZCA bears a stereogenic center at C-1', the elucidation of the absolute configuration of the sample was attempted by ECD spectroscopy. While the UV spectra can be adequately predicted by TD-DFT calculations (time-dependent density functional theory, see Supporting Information File 1 for details), a comparison of the experimental and calculated ECD spectra did not allow for a safe assignment of the cannabimimetic’s absolute configuration (Figure 4).

The main two positive ECD signals in the range above 230 nm are correctly predicted by TD-DFT; however, the experimental positive/negative sequence is inverted in the range from 200 to 230 nm. Overall, this yields an enantiomeric similarity index (ESI) [16] of only 34% in favor of the *S*-enantiomer, therefore not enabling a reliable assignment of the absolute configuration. The theoretical predictions do not change significantly if the Tamm–Dancoff approximation (TDA) is used (see Supporting Information File 1 for details). The same holds true if Ahlrichs basis sets are used in place of the traditional Pople basis sets irrespective of different solvation models (COSMO or SMD) or the inclusion of diffuse functions (def2-TZVPP vs ma-def2-TZVPP). The use of the range-separated (long-range corrected) CAM-B3LYP and ωB97XD functionals – probably more appropriate for the description of charge-transfer states – also did not lead to an improvement of the data.

**Figure 5:**
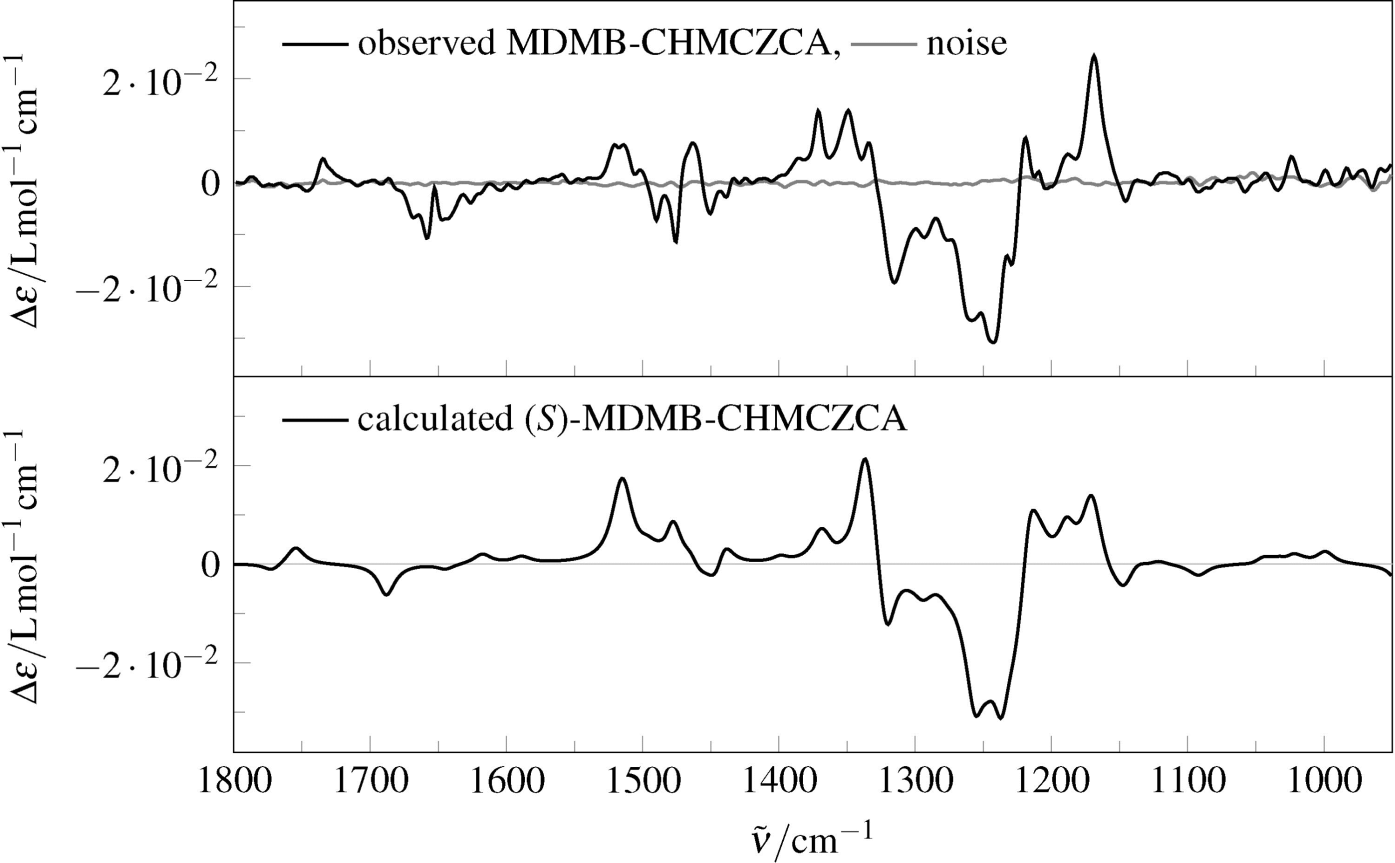
Observed (top) and calculated (bottom) VCD spectra for (*S*)-**3** in chloroform, theory level: B3PW91/6-311G**/IEFPCM.

On the other hand, an assignment was possible by comparison of the experimental and DFT-calculated VCD spectra (Figure 5) with a satisfactory ESI value of 80%.

**Figure 6:**
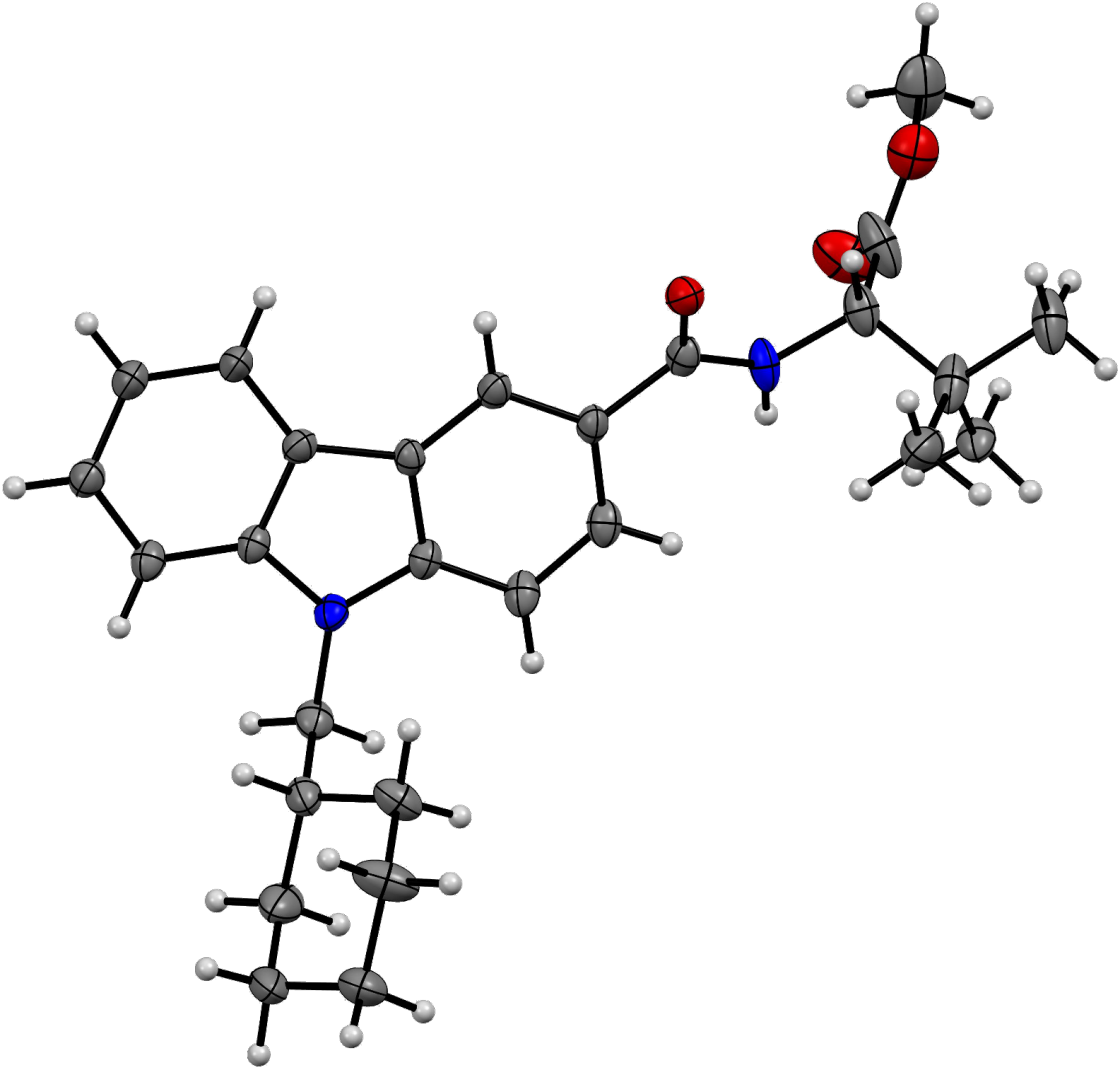
Molecular structure of (*S*)-**3** in the solid state at 110 K (ORTEP-ellipsoids drawn at 30% probability, C black, H gray, N blue, O red).

Eventually, the absolute configuration of (*S*)-**3** could be unambiguously determined by X-ray structure analysis of a single crystal, which was obtained by slow cooling of a hot saturated solution of (*S*)-**3** in cyclohexane (Figure 6, see also CCDC 1521512 for details).

**Figure 7:**
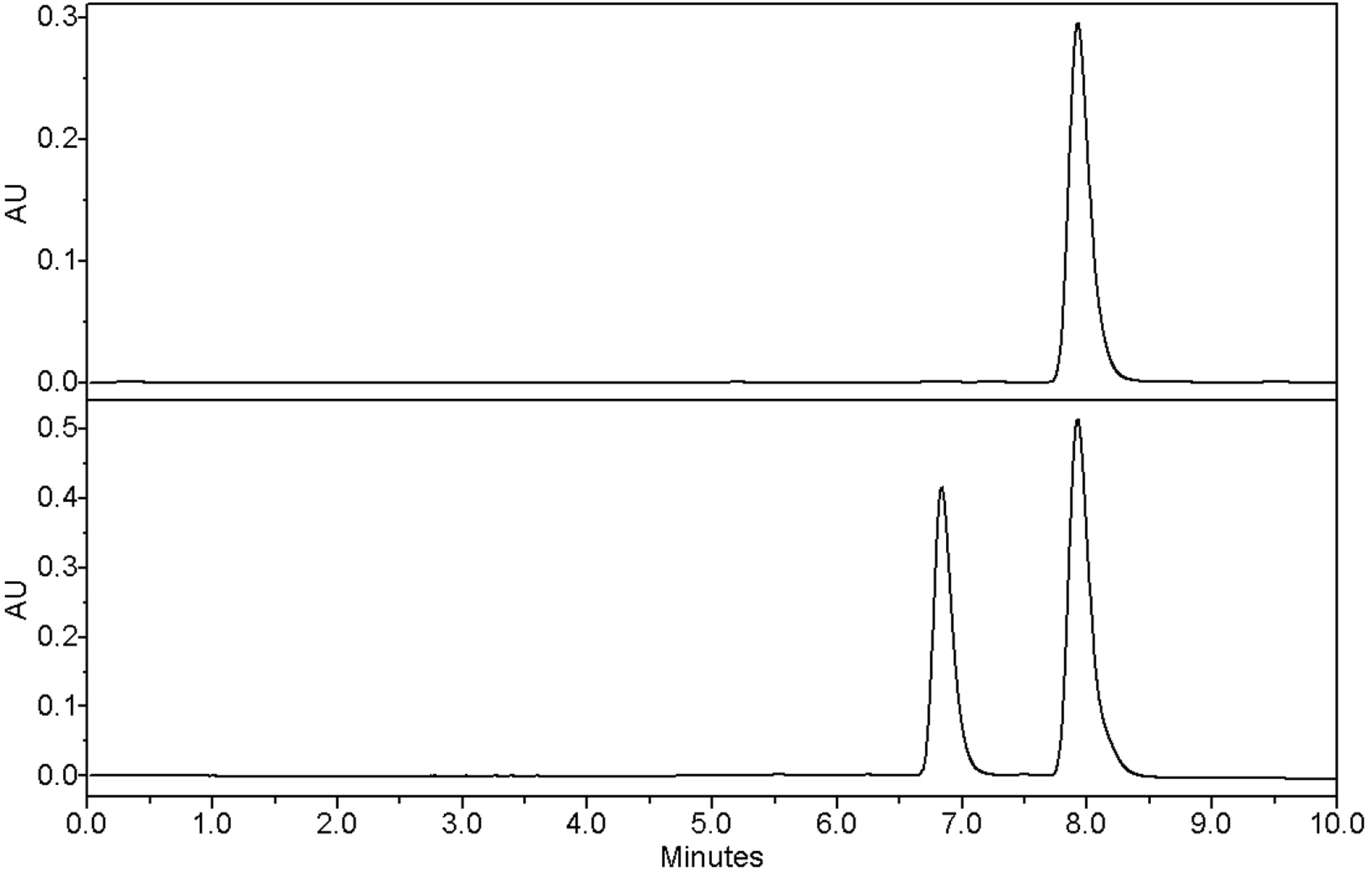
UV traces (254 nm) of the chiral HPLC for commercial (*S*)-**3** (top) and the (*R*/*S*)-**3** (bottom). *t*_R_ = 6.84 min (*R*) and 7.93 min (*S*).

To assess the enantiomeric purity of the material, racemization of a small sample of (*S*)-**3** was attempted by treatment with sodium methoxide in methanol at 80 °C for 12 h under rigorous exclusion of moisture, yielding an *R*/*S* mixture. The HPLC method already developed for the related cannabinoid MDMB-CHMICA [9] could be extended to the separation of the enantiomers. The commercial sample from test purchase 1 was found to be enantiopure (*S*)-**3** within the detection limits (Figure 7). Essentially the same applies to seven other samples (Table 1); small amounts of the (*R*)-enantiomer could only be detected in the case of test purchase 3.

**Table 1:** Enantiomeric excesses of (*S*)-**3** as determined by chiral HPLC.

sample	ee

(*R*/*S*)-**3**	18.5%
test purchase 1 (Dec 2015)^a^	>99.0%
test purchase 2 (May 2016)^a^	>99.0%
test purchase 3 (Jan 2016)^b^	97.0%
test purchase 4 (Mar 2016)^c^	>99.0%
test purchase 5 (Mar 2016)^c^	>99.0%
police seizure 1 (Feb 2015)^c^	>99.0%
police seizure 2 (Mar 2015)^c^	>99.0%
police seizure 3 (Aug 2016)^c^	>99.0%

^a^Pure **3** from internet RC shop. ^b^Extracted **3** (hashish-like resin). ^c^Extracted **3** (herbal mixture).

## Conclusion

A pure sample of the new synthetic cannabinoid MDMB-CHMCZCA (**3**), purchased in an online RC shop, was characterized and the absolute configuration was determined to be (*S*) by VCD spectroscopy and comparison with DFT calculations. Thus, the readily available (*S*)-*tert*-leucine would be a starting material for the synthesis of (*S*)-**3**. ECD spectroscopy was inferior to VCD in this case. The enantiomeric purity of material from five test purchases and three police seizures (pure substances as well as designer drug products) was assessed by chiral HPLC after base-induced racemization of a sample of (*S*)-**3**.

## Experimental

### Isolation of MDMB-CHMCZCA from Spice products

In analogy to the procedure described in [9], hashish-like resin (20 mg) was cut in small pieces, soaked in acetonitrile (3 mL) and sonicated for 20 minutes. The extract was filtered through a syringe filter (0.45 μm) and dried under a steady stream of nitrogen, giving a colorless material. From each herbal blend sample (Spice product), plant material (500 mg) was weighed and extracted twice with acetonitrile (5 mL and 2 mL). The extracts were combined, filtered through a syringe filter (0.45 μm) and dried under a steady stream of nitrogen, giving a green material.

Further extract purification was achieved by preparative normal-phase chromatography with a Sepacore^®^ flash system X50 (Büchi) consisting of two pump modules (max. 50 bar pressure), a UV–vis spectrometer (set to 285 nm), an automated fraction collector and a control unit. A prepacked 4 g silica gel HP column (particle size 15–40 μm, Büchi) was used. Separation was achieved using a gradient program of eluent A (hexane) and B (ethyl acetate) with a flow rate of 20 mL min^−1^, starting with 0% B, increasing over 30 seconds to 10% B. After 120 seconds, eluent B is further increased within 30 seconds to 30%, which is held for 120 seconds. Lastly, eluent B is increased to 100 % over 60 seconds and held for 240 seconds, giving an overall chromatographic runtime of 600 seconds. The fractions containing compound **3** were collected from 4 min to 4.6 min and subsequently evaporated to dryness.

### Nuclear magnetic resonance

All nuclear magnetic resonance (NMR) data were acquired on an Avance-III 600 MHz spectrometer (Bruker) with a 5 mm TCI CryoProbe. The ^1^H and ^13^C chemical shifts (δ) were referenced to the residual solvent signal as internal standard (δ_H_ = 7.26 ppm and δ_C_ = 77.16 ppm) [17–18]. The ^15^N chemical shifts were referenced to an external standard (nitromethane in DMSO-*d*_6_, δ_N_ = 380.2 ppm) and the coupling constants (*J*) are reported in Hz.

### Melting point

The melting point was determined in open capillary tubes with a KSP1N melting point meter (Krüss).

### HPLC/ESI-MS*^n^*

HPLC/ESI-MS*^n^* was performed on a 1200 series HPLC system with a UV diode array detector coupled with a LC/MSD trap XCT mass spectrometer (Agilent Technologies). Mixtures of acetonitrile and water (with 0.1% formic acid) were used as eluents at a total flow rate of 1.0 mL min^−1^ with the following gradient method: acetonitrile/water (+ 0.1% formic acid) = 10:90 (0–0.2 min), 10:90 to 90:10 (0.2–4.0 min), and 90:10 (4.0–6.0 min). An Ascentis Express C_18_ column (length: 3 cm, diameter: 2.1 mm, particle size: 2.7 μm; Supelco) was used at a temperature of 40 °C. The capillary voltage was set to 3500 V and the capillary exit voltage was set according to the respective target mass.

### High-resolution mass spectrometry

High-resolution ESI mass spectrometry was performed on a Waters QTof Ultima 3 instrument (Waters) with a dual electrospray source and an external calibrant.

### Polarimetry

The determination of the optical rotation was carried out at 589 nm and 25 °C in a Perkin-Elmer 241 polarimeter (Perkin Elmer) using a 10 cm path length quartz glass cuvette.

### Electronic spectroscopy

UV and ECD spectra were obtained on a J-815 circular dichroism spectropolarimeter (Jasco) in a 1 mm (path length) quartz glass cuvette and a spectral range of 400–185 nm. The measurements were carried out with an acetonitrile solution of MDMB-CHMCZCA (0.25 mmol/L), a scan speed of 20 nm/min and 8 repetitions. The baseline was corrected by subtraction of a solvent spectrum recorded with the same parameters.

### Vibrational spectroscopy

The infrared (IR) and vibrational circular dichroism (VCD) spectra were recorded with a Tensor 27 IR spectrometer (Bruker Optics) equipped with a PMA50 module for polarization modulation measurements. The photoelastic modulator was optimized for 1400 cm^−1^. The IR data were collected within 16 scans in a spectral range of 4000–800 cm^−1^. The measurement to obtain the VCD data was carried out with a solution of MDMB-CHMCZCA in CDCl_3_ (0.168 mol/L) in a 100 µm BaF_2_ sample cell with an accumulation time of 6 h (25,560 scans). The resolution in the spectral range of 1800–800 cm^−1^ was set to 4 cm^−1^. The IR and VCD spectra were baseline corrected by subtraction of a solvent spectrum recorded using the same measurement parameters.

### Computational methods

Initially, 445 MMFF [19] and 470 PM6 [20] conformational candidates for (*S*)-**3** were generated employing a sparse systematic search algorithm with Spartan’10 [21]. Both sets were combined, the candidates were optimized and vibrational frequencies were calculated with PM6 [20] using Gaussian 09, Rev. D.01 [22]. Duplicates were removed based on comparison of the electronic energies and dipole moments as well as the free enthalpies and dipole moments, yielding 740 survivors. DFT reoptimization at the B3LYP/6-31G* level [23–28] with IEFPCM (integral equation formalism polarizable continuum model) solvation [29] for chloroform, tight geometry convergence criteria, and an ultrafine integration grid yielded 619 conformers after removal of duplicates based on comparison of the electronic energies and dipole moments. The 242 conformers within a relative electronic energy range up to 10 kcal/mol were selected from this set. The geometries were reoptimized and vibrational frequencies were calculated at the B3LYP/6-311G** level [23–28] with IEFPCM solvation [29] for chloroform, tight geometry convergence criteria, and an ultrafine integration grid. Duplicates were removed based on comparison of the electronic energies and dipole moments as well as the free enthalpies and dipole moments (235 survivors). The 43 conformers within a relative electronic energy range up to 5 kcal/mol were selected from this set, the structures were confirmed as local minima (no imaginary frequencies), and enthalpy-Boltzmann-averaged IR and VCD spectra were generated. Using SpecDis 1.64 [16], the experimental and calculated IR spectra were fitted in the range from 950 cm^–1^ to 1550 cm^–1^ with screening values of 2 cm^–1^ to 10 cm^–1^ for the line broadening γ as well as 0.9 to 1.1 for the scaling factor *s*. The experimental and calculated VCD spectra were then compared using these optimized parameters. The set of 242 conformers mentioned above was also subjected to a reoptimization and vibrational frequency calculation at the B3LYP/6-311G** level [23–28] with IEFPCM solvation [29] for acetonitrile, tight geometry convergence criteria, and an ultrafine integration grid using Gaussian 09, Rev. D.01 [22]. Duplicates were removed based on comparison of the electronic energies and dipole moments as well as the free enthalpies and dipole moments (234 survivors). The 46 conformers within a relative electronic energy range up to 5 kcal/mol were selected from this set, the structures were confirmed as local minima (no imaginary frequencies), and the geometries were used for the excited-state calculations. ECD spectra were calculated using TD-DFT (number of states: 75) at the TD- and TDA-B3LYP/6-311++G** [23–28,30] as well as the TD-CAM-B3LYP/def2-TZVPP [31–33] and TD-ωB97XD/def2-TZVPP [32–35] levels with IEFPCM solvation [29] for acetonitrile and an ultrafine integration grid. ECD spectra were also calculated using TD-DFT (with TDA, number of roots: 100, size of the expansion space: 600) with Orca 3.0.3 [36] employing the B3LYP [23–26] or CAM-B3LYP [31] functionals, the def2-TZVPP basis set [32–33], the RIJCOSX approximation [37] together with the def2-TZVPP/J basis set, tight SCF criteria, enhanced grid settings (Grid5 FinalGrid6 GridX4) and COSMO solvation [38] for acetonitrile. Enthalpy-Boltzmann-averaged UV and ECD spectra were then generated in all cases. Using SpecDis 1.64 [16], the experimental and calculated UV spectra were fitted in the range from 200 nm to 400 nm using screening values of 0.1 eV to 0.5 eV for the line broadening σ/γ as well as −60 nm to +60 nm for the shift value *s*; the experimental and calculated ECD spectra were then compared using these optimized parameters.

### Racemization

Analogously as described in [9], (*S*)-**3** (2.5 mg) was added to a freshly prepared solution of sodium (10 mg) in dry methanol (3 mL) under nitrogen and stirred at 80 °C for 12 h. The mixture was quenched by addition of acetic acid (2 mL). Water (30 mL) was added and the mixture was extracted with ethyl acetate (50 mL). The organic layer was washed with saturated aqueous NaHCO_3_ (20 mL) and brine (20 mL), and dried over sodium sulfate. The solvent was removed in vacuo to afford (*R*/*S*)-**3** as a colorless solid (2.3 mg, 92%).

### Chiral HPLC

Chiral HPLC was performed on an Alliance 2695 HPLC (Waters) coupled to a 996 PDA detector (Waters). Mixtures of hexane and 2-propanol were used as eluents at a total flow rate of 0.6 mL/min with an isocratic ratio of hexane/2-propanol = 20:80. A Chiralpak IA-3 column (length: 25 cm, diameter: 4.6 mm, particle size: 3 μm; Daicel) was used at a temperature of 40 °C.

## Supporting Information

File 1 NMR spectra, UV and ECD spectra, IR and VCD spectra, HPLC/ESI-MS*^n^*, chiral HPLC, and computational chemistry.

File 2 Chemical information file of compound (*S*)-**3**.
CCDC 1521512 contains the supplementary crystallographic data for this paper. The data can be obtained free of charge from The Cambridge Crystallographic Data Centre via http://www.ccdc.cam.ac.uk/structures
