# Supplementary material for: Characterization of the synthetic cannabinoid MDMB-CHMCZCA
Source: Beilstein J Org Chem. 2016 Dec 21;12:2808–15. doi: 10.3762/bjoc.12.279 (PMC5238538; doi:10.3762/bjoc.12.279)
Supplement: File 1 — NMR spectra, UV and ECD spectra, IR and VCD spectra, HPLC/ESI-MSn, chiral HPLC, and computational chemistry. [file Beilstein_J_Org_Chem-12-2808-s001.pdf]

Supporting Information

for

Characterization of the synthetic cannabinoid MDMB-CHMCZCA

Carina Weber[1§], Stefan Pusch[1§], Dieter Schollmeyer[1], Sascha Münster-Müller[2], Michael Pütz[2]* and Till Opatz[1]*

Address: [1]Johannes Gutenberg University Mainz, Institute of Organic Chemistry, Duesbergweg 10–14, 55128 Mainz, Germany and [2]Bundeskriminalamt – Federal Criminal Police Office (BKA), Forensic Science Institute, KT 45 – Toxicology, Äppelallee 45, 65203 Wiesbaden, Germany

Email: Michael Pütz - michael.puetz@bka.bund.de; Till Opatz - opatz@uni-mainz.de

[§]Equal contributors

*Corresponding author

NMR spectra, UV and ECD spectra, IR and VCD spectra, HPLC/ESI-MS^{n}, chiral HPLC, and computational chemistry

Contents

1 NMR Spectra **S3**

2 UV and ECD Spectra **S8**

2.1 TD-B3LYP/6-311++G**/IEFPCM . . . S8
2.2 TDA-B3LYP/6-311++G**/IEFPCM . . . S9
2.3 TDA-RIJCOSX-B3LYP/def2-TZVPP/COSMO . . . S10
2.4 TDA-RIJCOSX-B3LYP/def2-TZVPP/SMD . . . S11
2.5 TDA-RIJCOSX-B3LYP/ma-def2-TZVPP/COSMO . . . S12
2.6 TDA-RIJCOSX-CAM-B3LYP/def2-TZVPP/COSMO . . . S13
2.7 TD-CAM-B3LYP/def2-TZVPP/IEFPCM . . . S14
2.8 TD-ωB97XD/def2-TZVPP/IEFPCM . . . S15

3 IR and VCD Spectra **S16**

4 Crystal Structure Determination **S17**

5 HPLC/ESI-MS^n **S19**

6 Chiral HPLC **S37**

7 Computational Chemistry **S42**

7.1 Keyword Lines . . . S42
7.2 Boltzmann Weightings . . . S43
7.3 Geometries . . . S45

8 Bibliography **S61**

1 NMR Spectra

This section contains the NMR spectra of pure (S)-**3** from test purchase 1 (internet shop, Dec 2015).

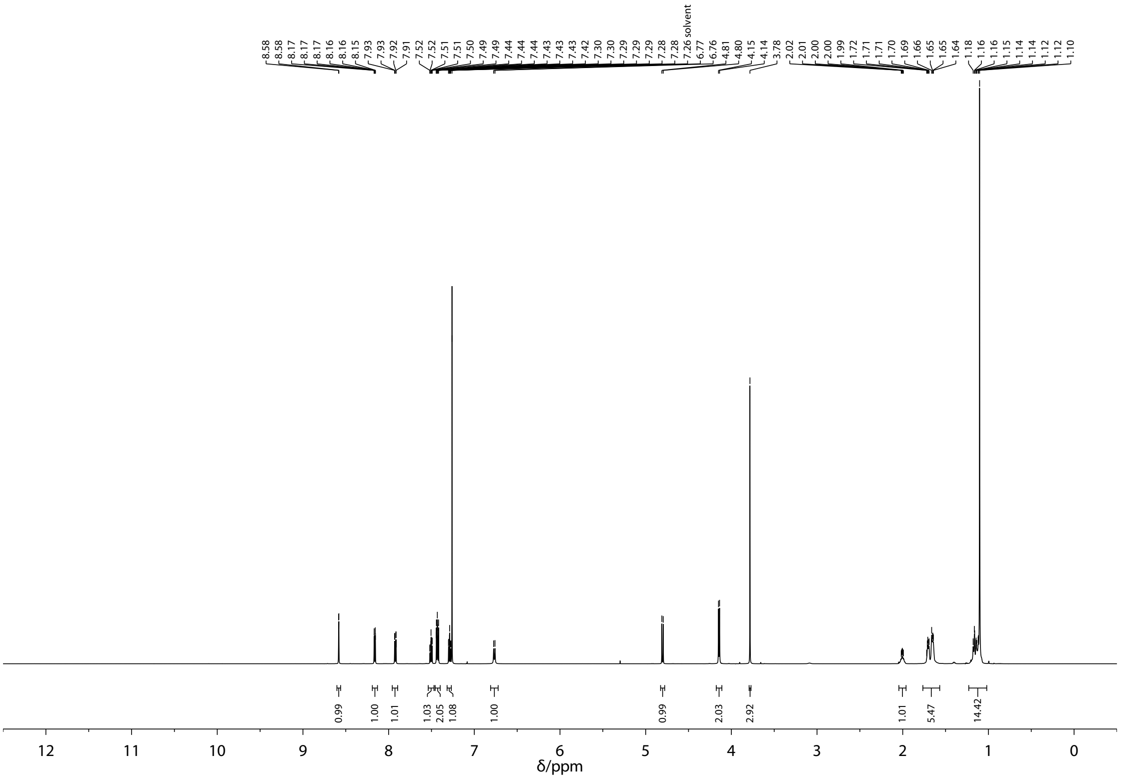

Figure 1.1: ^{1}H NMR spectrum (600 MHz, $CDCl_3$) of **3**.

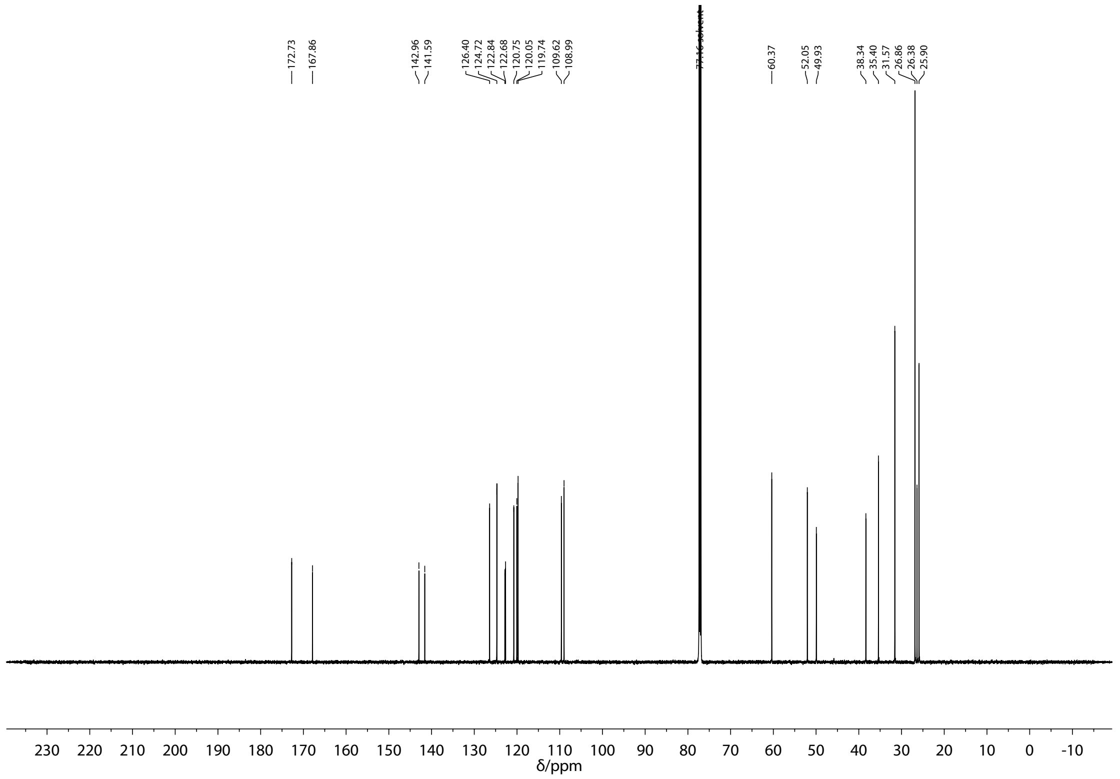

Figure 1.2: ^{13}C NMR spectrum (151 MHz, $CDCl_3$) of **3**.

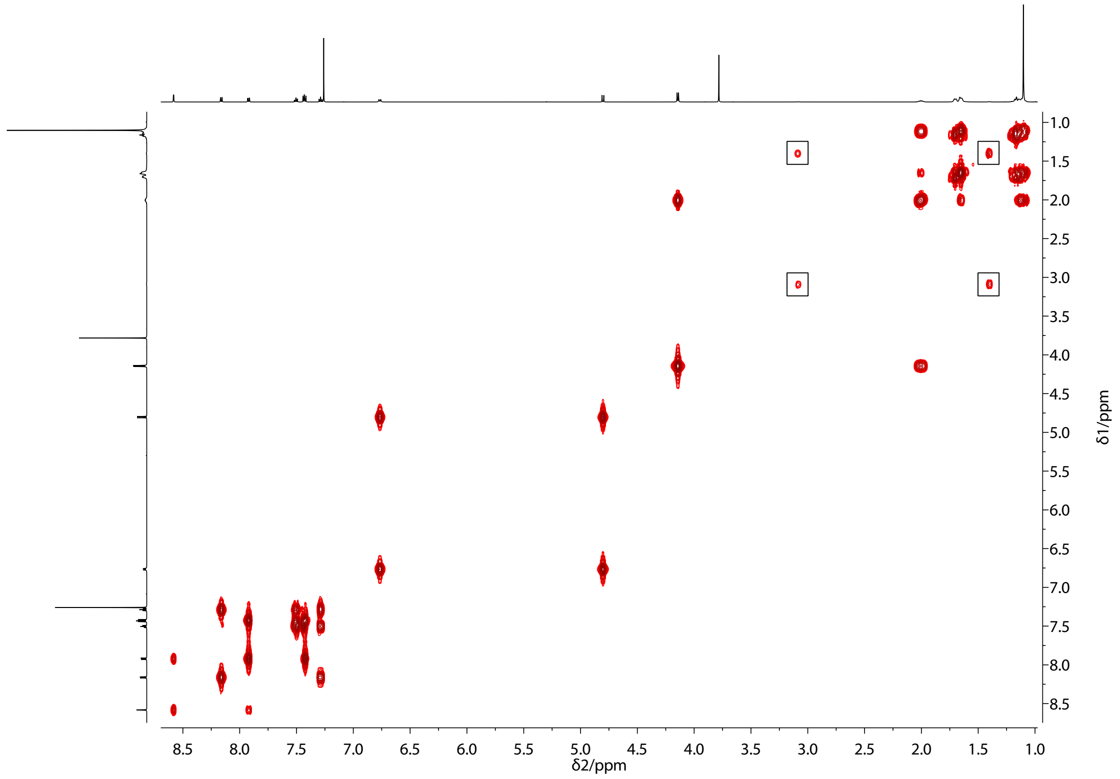

Figure 1.3: COSY spectrum (600 MHz, $CDCl_3$) of **3** (triethylammonium impurity marked with black boxes).

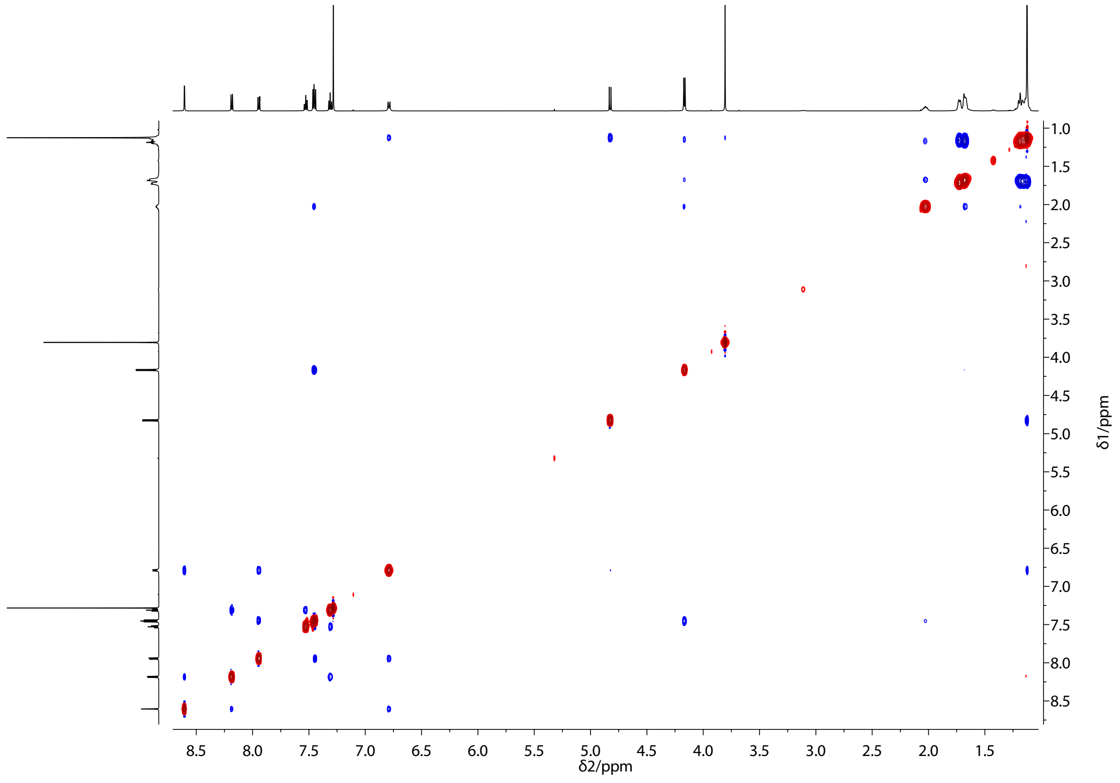

Figure 1.4: NOESY spectrum (600 MHz, $CDCl_3$) of **3**.

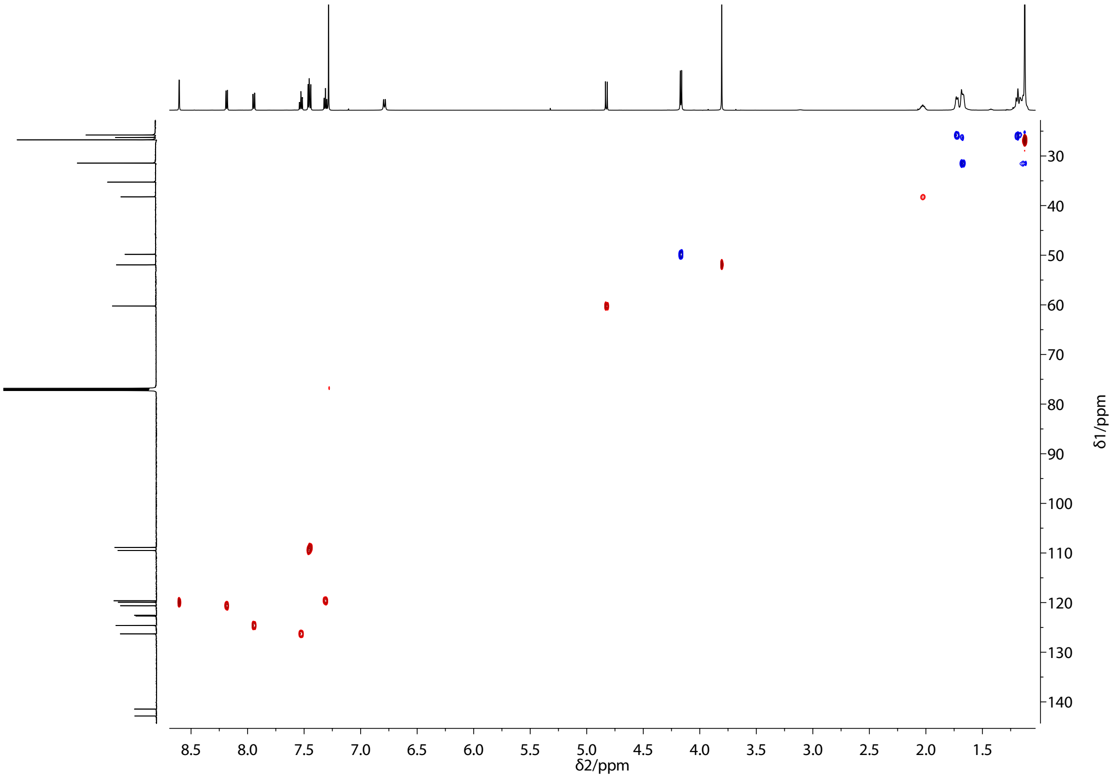

Figure 1.5: HSQC spectrum (600 MHz, $CDCl_3$) of **3**.

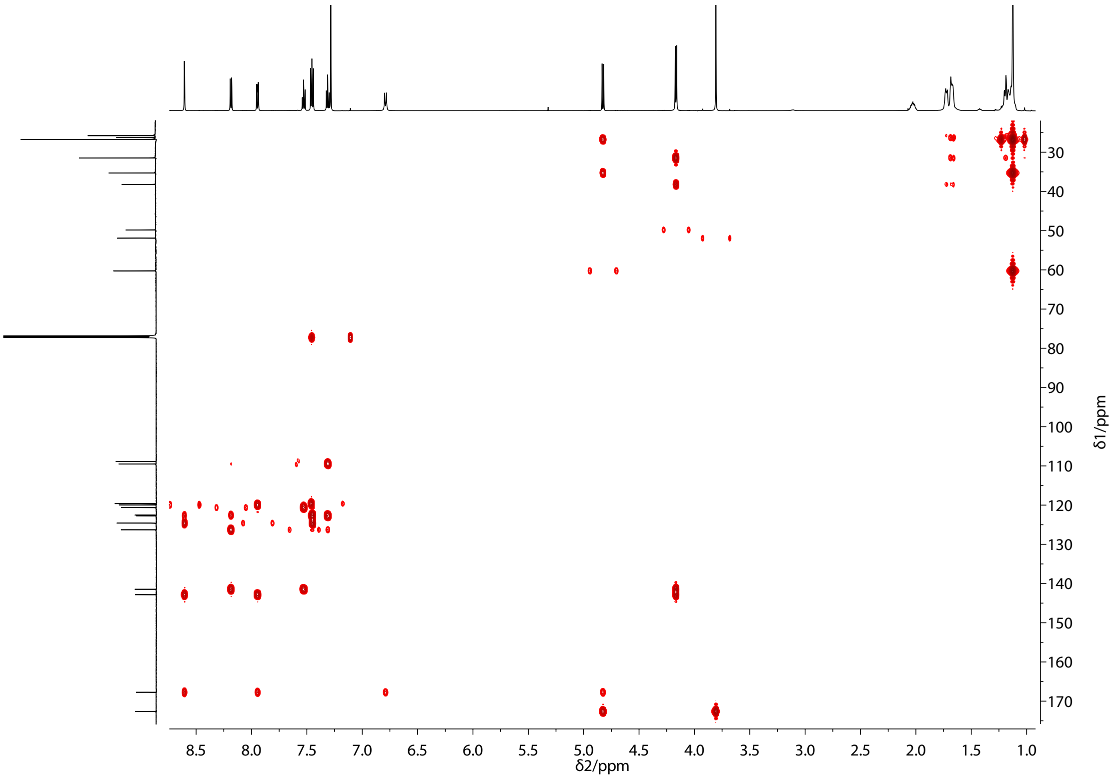

Figure 1.6: HMBC spectrum (600 MHz, $CDCl_3$) of **3**.

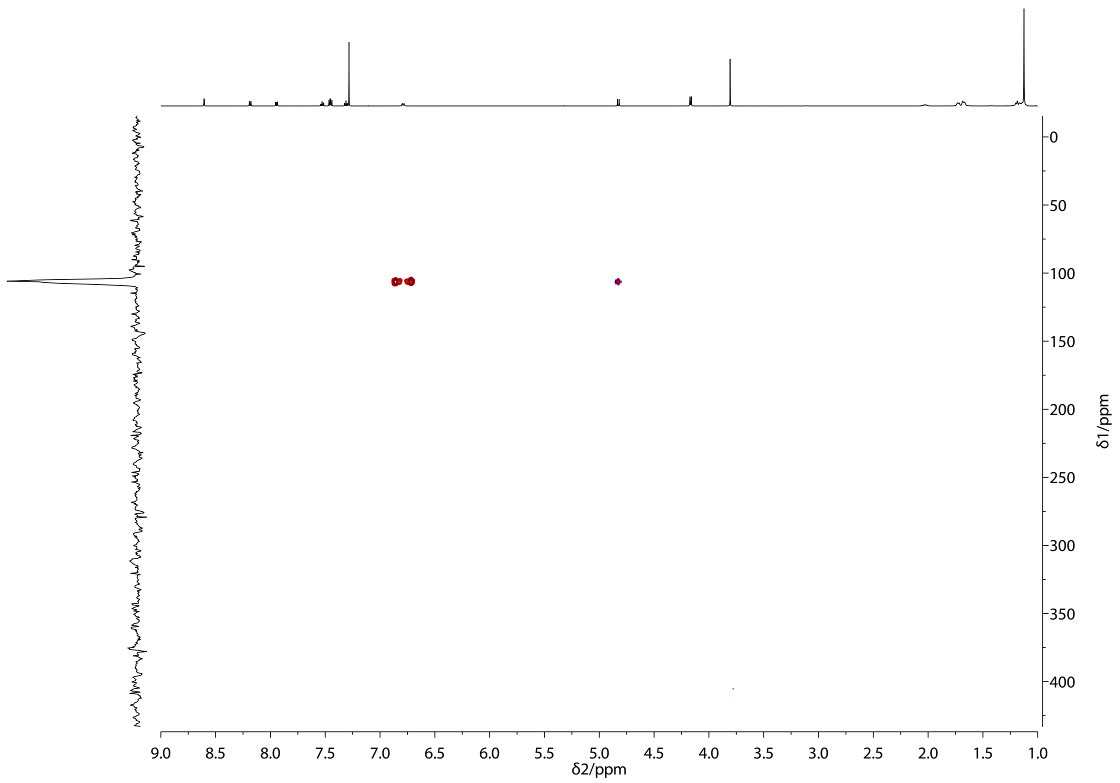

Figure 1.7: [^{1}H,^{15}N]-HSQC spectrum (600 MHz, $CDCl_3$) of **3**.

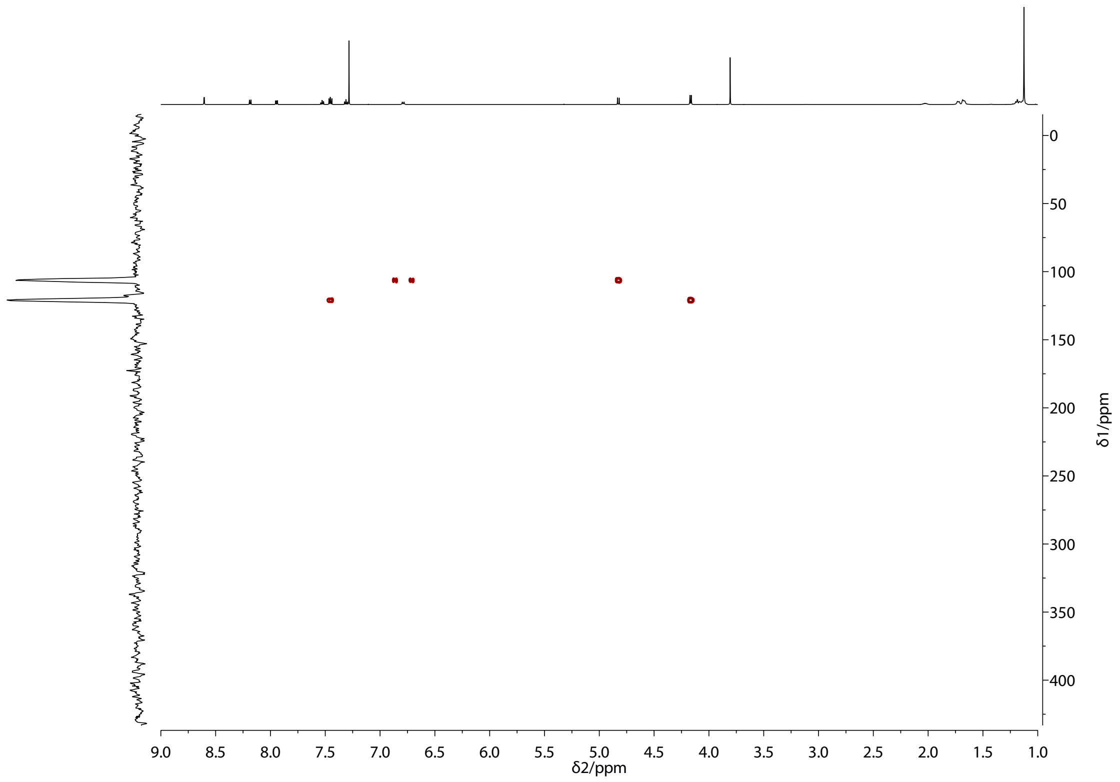

Figure 1.8: [^{1}H,^{15}N]-HMBC spectrum (600 MHz, $CDCl_3$) of **3**.

2 UV and ECD Spectra

This section contains the UV and ECD spectra of pure (S)-**3** from test purchase 1 (internet shop, Dec 2015).

2.1 TD-B3LYP/6-311++G**/IEFPCM

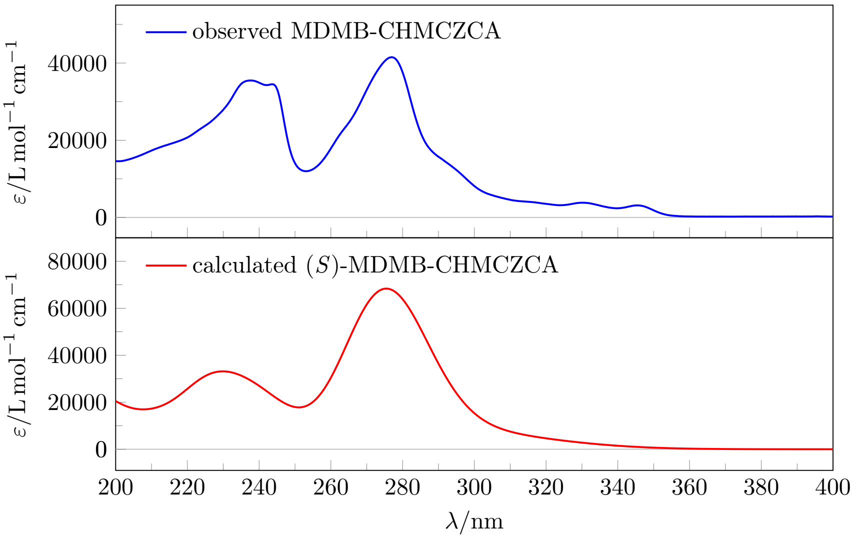

Figure 2.1: Observed (top) and calculated (bottom) UV spectra for (S)-**3** in MeCN.

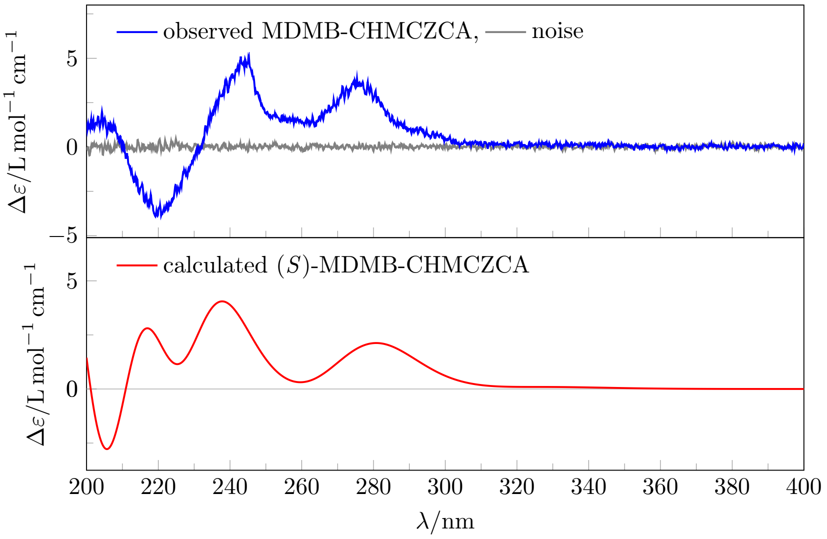

Figure 2.2: Observed (top) and calculated (bottom) ECD spectra for (S)-**3** in MeCN.

Fitting results: $\sigma/\gamma = 0.27\,\mathrm{eV}$, $s = 3\,\mathrm{nm}$, similarity UV: 0.9465, similarity ECD: 0.6473, similarity enantiomeric ECD: 0.3034, ESI: 0.3439.

2.2 TDA-B3LYP/6-311++G**/IEFPCM

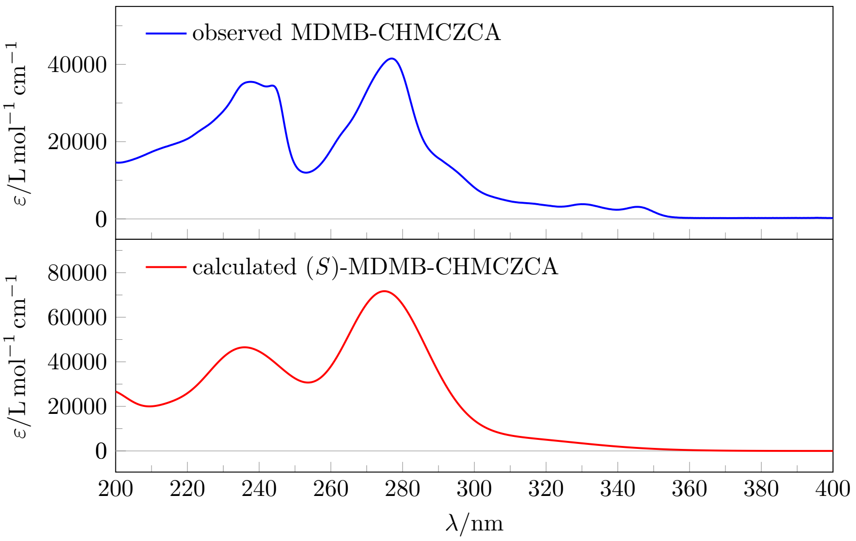

Figure 2.3: Observed (top) and calculated (bottom) UV spectra for (*S*)-**3** in MeCN.

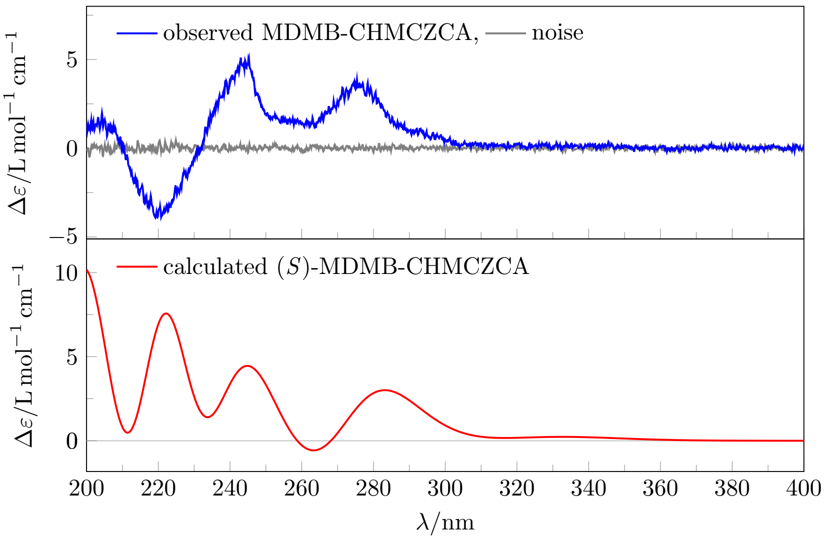

Figure 2.4: Observed (top) and calculated (bottom) ECD spectra for (*S*)-**3** in MeCN.

Fitting results: $\sigma/\gamma = 0.28\,\text{eV}$, $s = 11\,\text{nm}$, similarity UV: 0.9762, similarity ECD: 0.4830, similarity enantiomeric ECD: 0.4130, ESI: 0.0700.

2.3 TDA-RIJCOSX-B3LYP/def2-TZVPP/COSMO

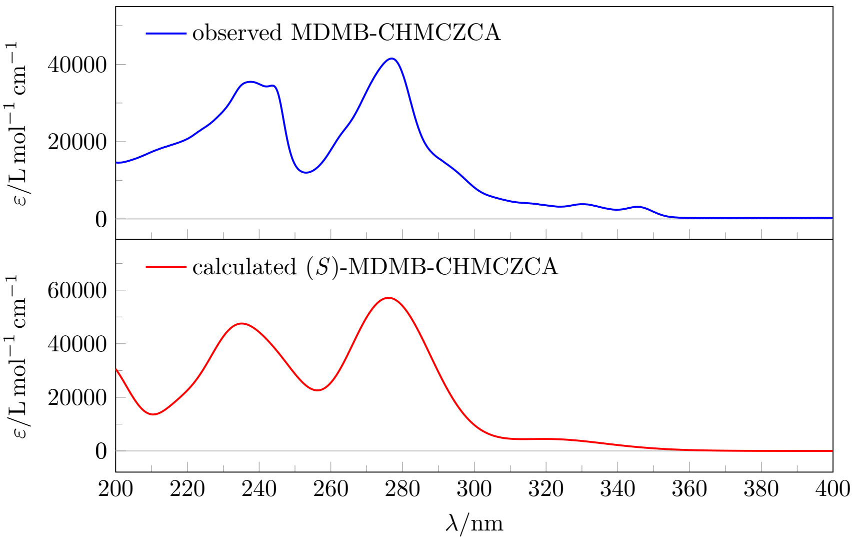

Figure 2.5: Observed (top) and calculated (bottom) UV spectra for (*S*)-**3** in MeCN.

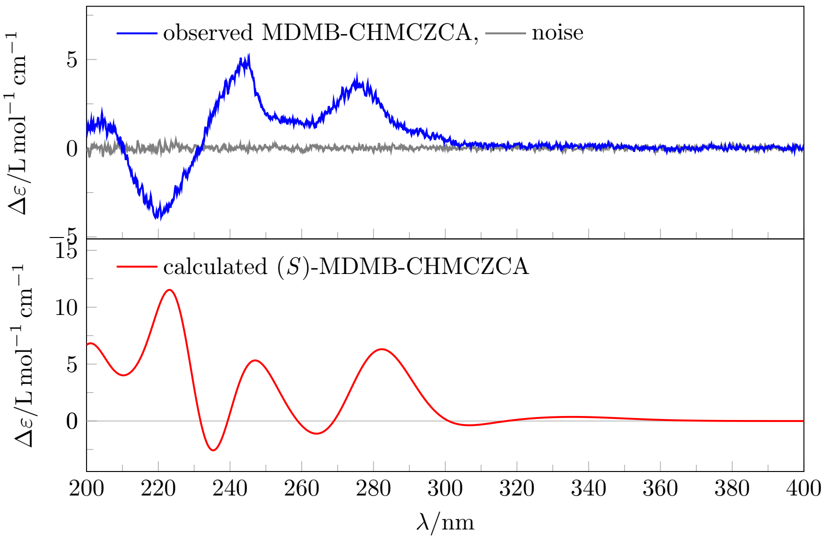

Figure 2.6: Observed (top) and calculated (bottom) ECD spectra for (*S*)-**3** in MeCN.

Fitting results: $\sigma/\gamma = 0.24\,\text{eV}$, $s = 14\,\text{nm}$, similarity UV: 0.9854, similarity ECD: 0.4137, similarity enantiomeric ECD: 0.5367, ESI: 0.1230 (inverted).

2.4 TDA-RIJCOSX-B3LYP/def2-TZVPP/SMD

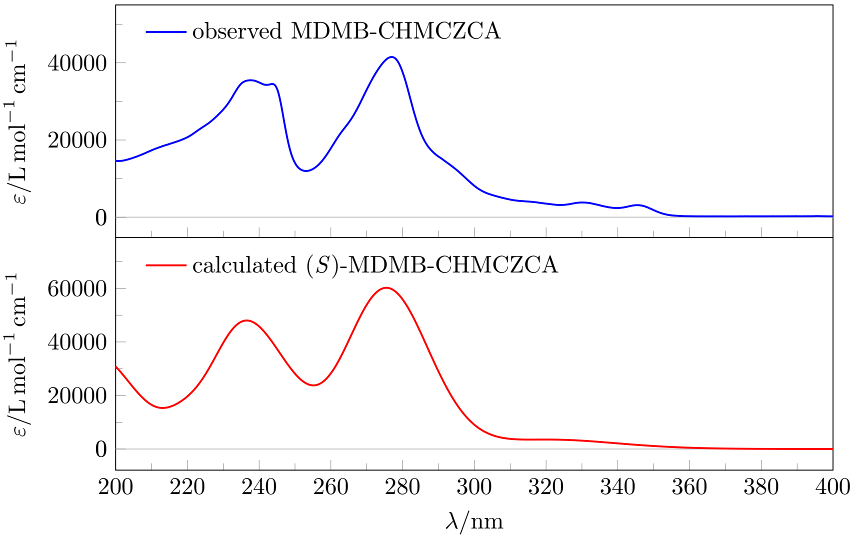

Figure 2.7: Observed (top) and calculated (bottom) UV spectra for (*S*)-**3** in MeCN.

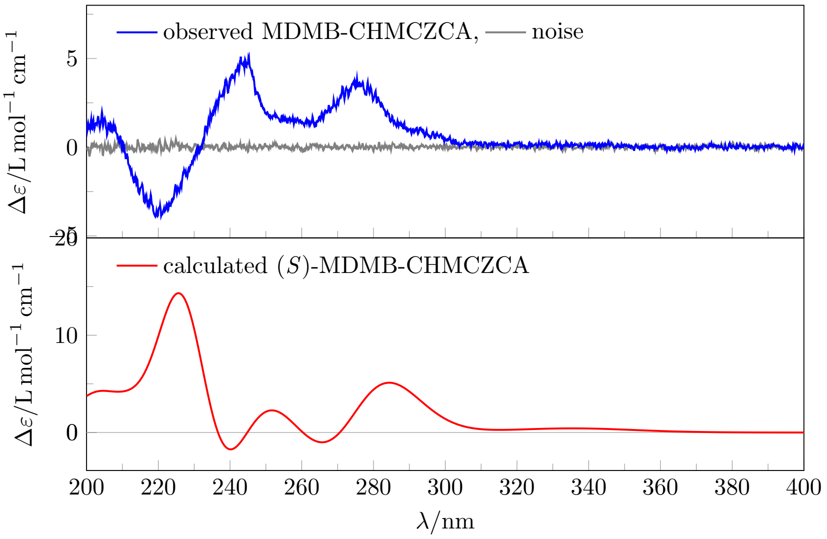

Figure 2.8: Observed (top) and calculated (bottom) ECD spectra for (*S*)-**3** in MeCN.

Fitting results: $\sigma/\gamma = 0.26\,\text{eV}$, $s = 16\,\text{nm}$, similarity UV: 0.9833, similarity ECD: 0.2221, similarity enantiomeric ECD: 0.6548, ESI: 0.4327 (inverted).

2.5 TDA-RIJCOSX-B3LYP/ma-def2-TZVPP/COSMO

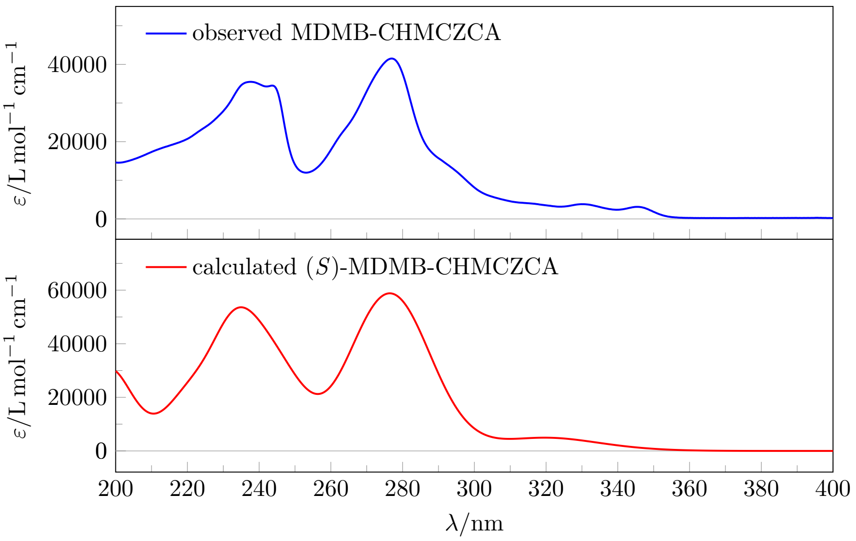

Figure 2.9: Observed (top) and calculated (bottom) UV spectra for (*S*)-**3** in MeCN.

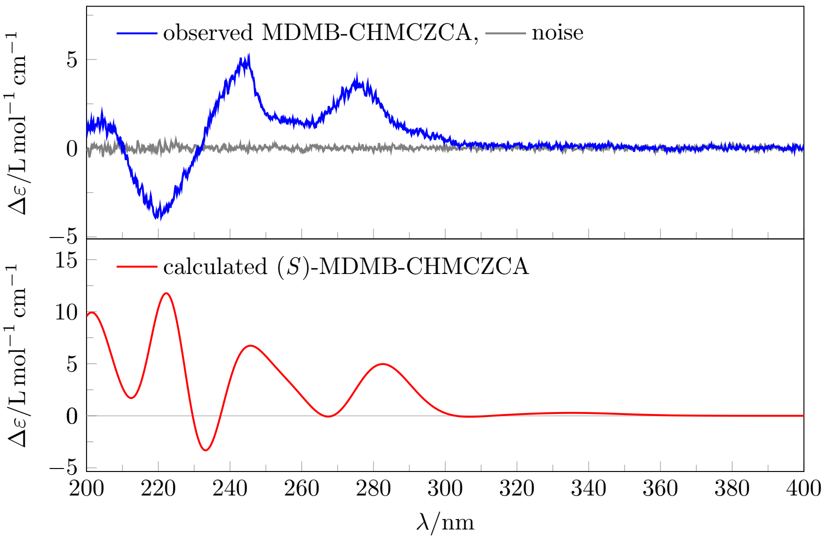

Figure 2.10: Observed (top) and calculated (bottom) ECD spectra for (*S*)-**3** in MeCN.

Fitting results: $\sigma/\gamma = 0.22\,\mathrm{eV}$, $s = 11\,\mathrm{nm}$, similarity UV: 0.9871, similarity ECD: 0.4887, similarity enantiomeric ECD: 0.3950, ESI: 0.0937.

2.6 TDA-RIJCOSX-CAM-B3LYP/def2-TZVPP/COSMO

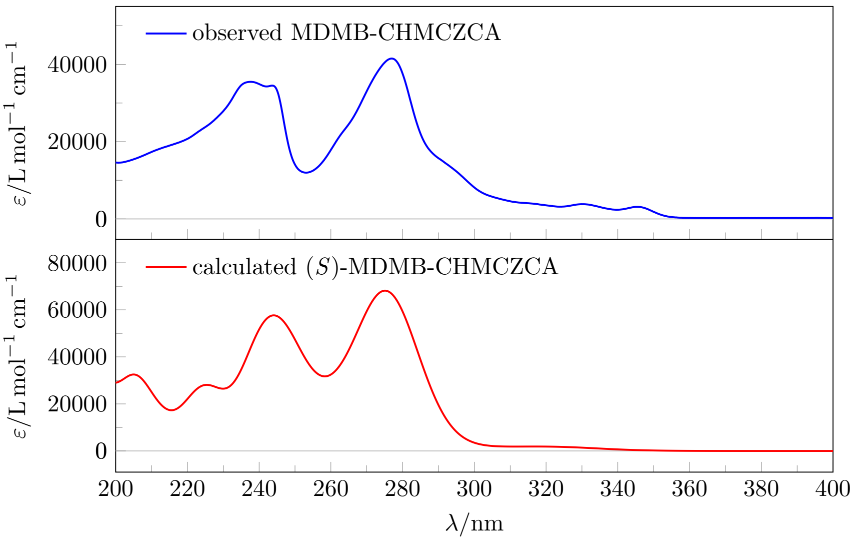

Figure 2.11: Observed (top) and calculated (bottom) UV spectra for (*S*)-**3** in MeCN.

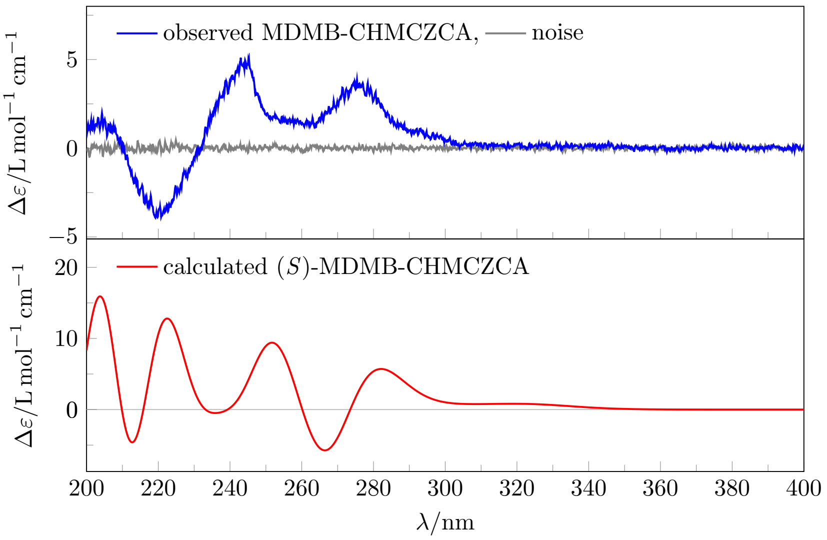

Figure 2.12: Observed (top) and calculated (bottom) ECD spectra for (*S*)-**3** in MeCN.

Fitting results: $\sigma/\gamma = 0.25\,\mathrm{eV}$, $s = 34\,\mathrm{nm}$, similarity UV: 0.9589, similarity ECD: 0.3943, similarity enantiomeric ECD: 0.3910, ESI: 0.0033.

2.7 TD-CAM-B3LYP/def2-TZVPP/IEFPCM

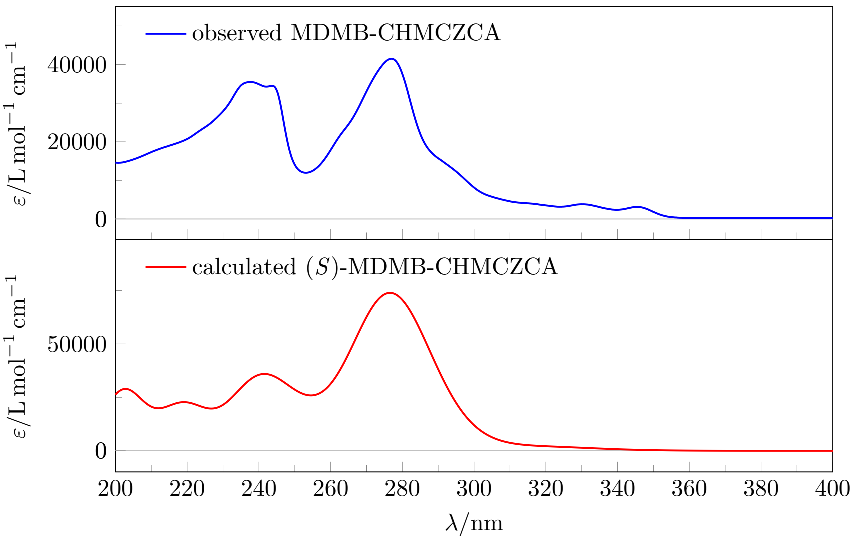

Figure 2.13: Observed (top) and calculated (bottom) UV spectra for (*S*)-**3** in MeCN.

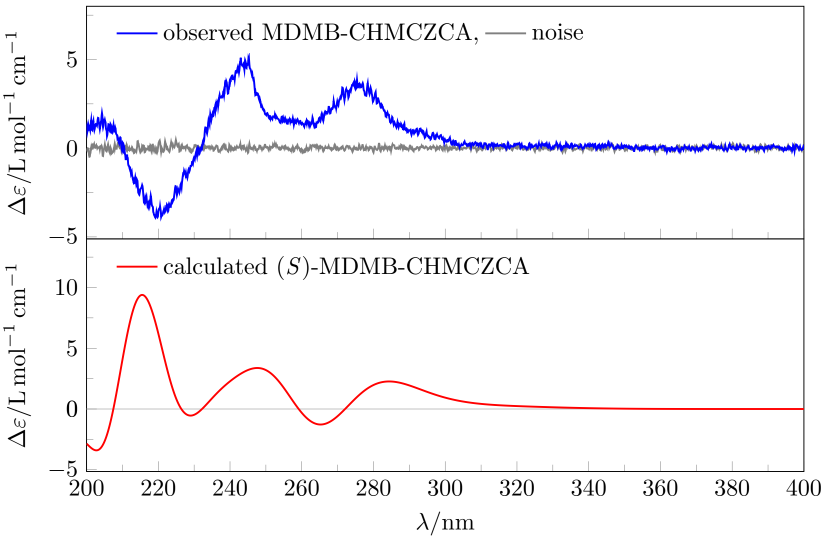

Figure 2.14: Observed (top) and calculated (bottom) ECD spectra for (*S*)-**3** in MeCN.

Fitting results: $\sigma/\gamma = 0.29\,\text{eV}$, $s = 24\,\text{nm}$, similarity UV: 0.9431, similarity ECD: 0.3274, similarity enantiomeric ECD: 0.4607, ESI: 0.1333 (inverted).

2.8 TD-ωB97XD/def2-TZVPP/IEFPCM

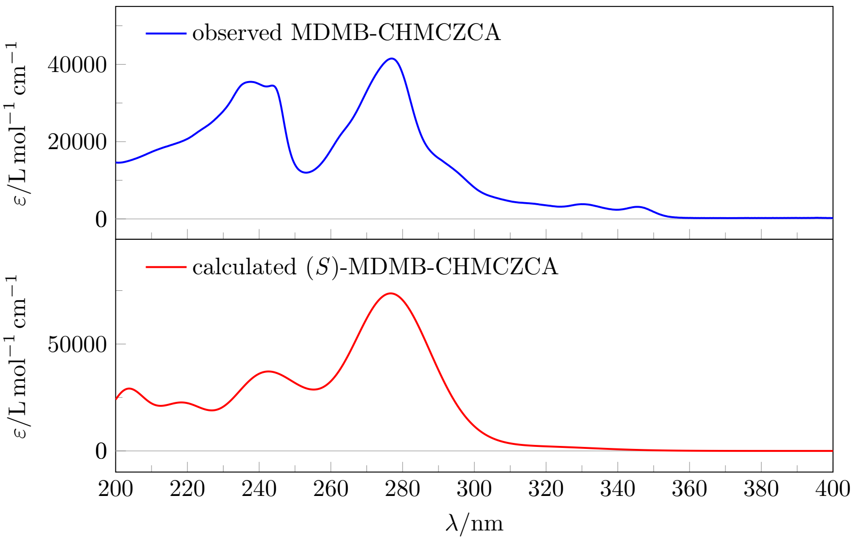

Figure 2.15: Observed (top) and calculated (bottom) UV spectra for (*S*)-**3** in MeCN.

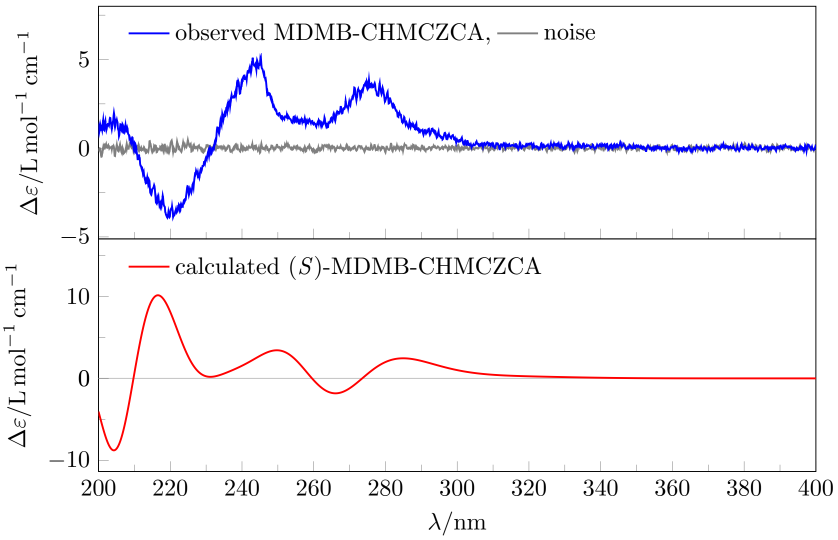

Figure 2.16: Observed (top) and calculated (bottom) ECD spectra for (*S*)-**3** in MeCN.

Fitting results: $\sigma/\gamma = 0.29\,\text{eV}$, $s = 26\,\text{nm}$, similarity UV: 0.9428, similarity ECD: 0.2600, similarity enantiomeric ECD: 0.4873, ESI: 0.2274 (inverted).

3 IR and VCD Spectra

This section contains the IR and VCD spectra of pure (*S*)-**3** from test purchase 1 (internet shop, Dec 2015).

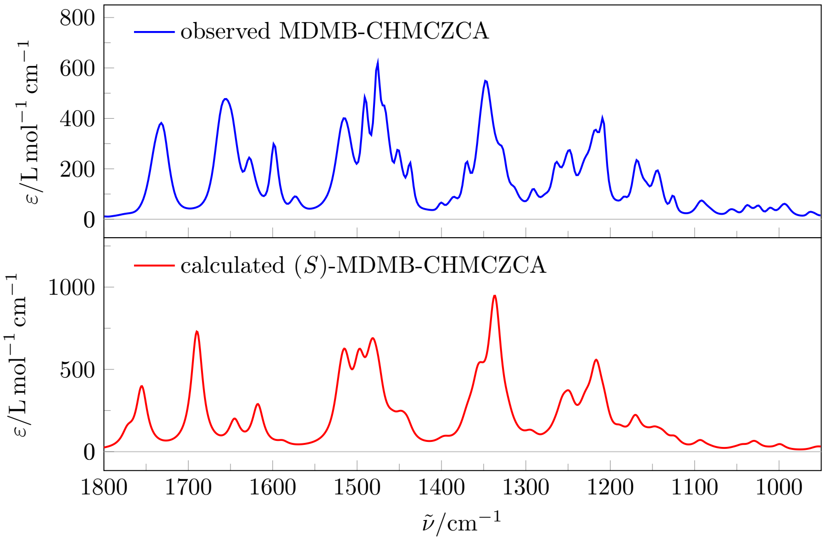

Figure 3.1: Observed (top) and calculated (bottom) IR spectra for (*S*)-**3** in $CDCl_3$.

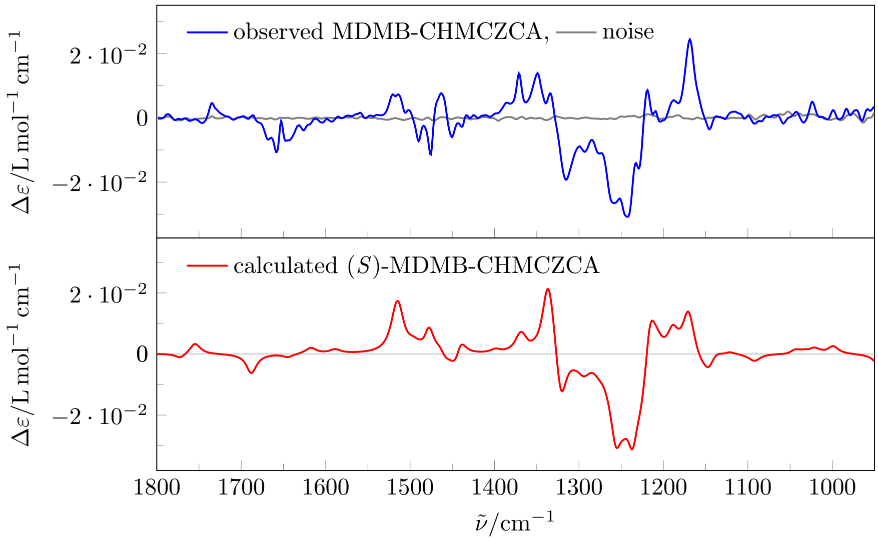

Figure 3.2: Observed (top) and calculated (bottom) VCD spectra for pure (*S*)-**3** in $CDCl_3$.

Fitting results: $\gamma = 8\,\mathrm{cm}^{-1}$, $s = 0.982$, similarity IR: 0.9535, similarity VCD: 0.8263, similarity enantiomeric VCD: 0.0255, ESI: 0.8008.

4 Crystal Structure Determination

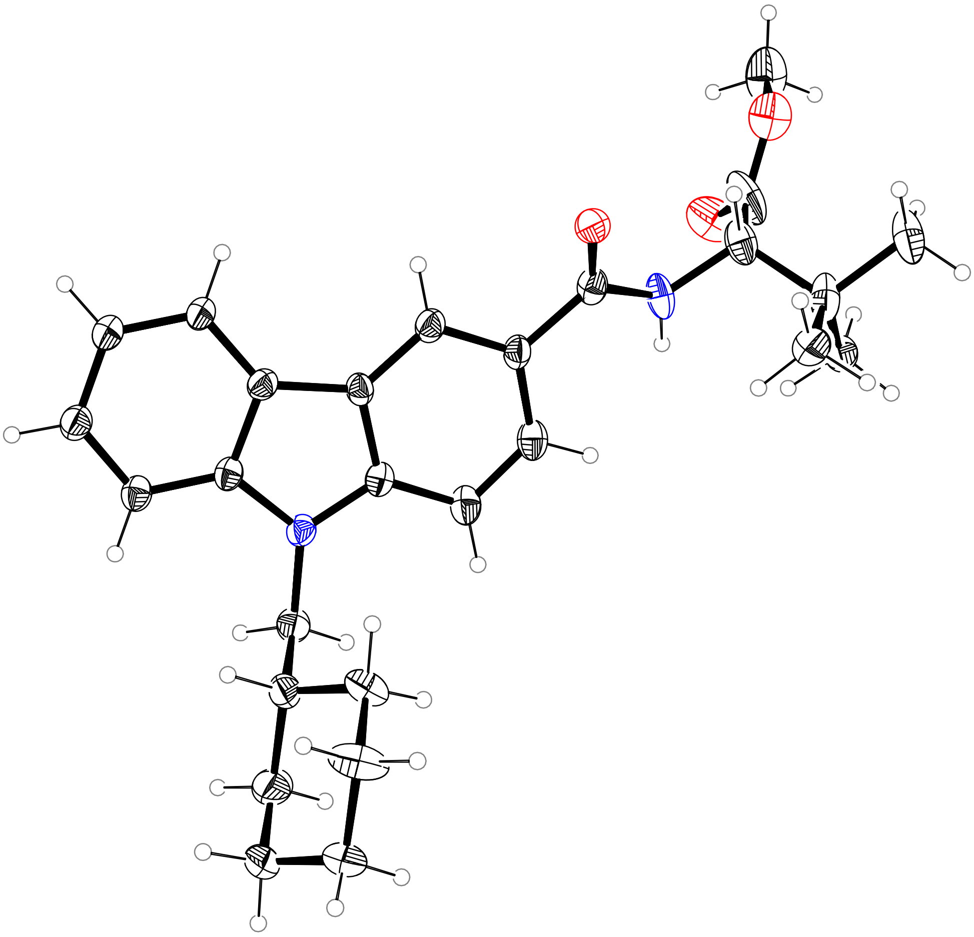

Figure 4.1: Molecular structure of (S)-**3** in the solid state (ORTEP-ellipsoids drawn at 30 % probability, C: black, H: gray, N: blue, O: red).

Table 4.1: Crystal structure determination of (S)-**3**.

Crystal data:	
molecular formula	$C_{27}H_{34}N_2O_3$
molar mass	$434.6\,g\,mol^{-1}$
crystal system	orthorhombic
space group	$P2_12_12_1$
absorption coefficient (μ)	$0.62\,mm^{-1}$ (corrected with 6 crystal faces)
transmission	$T_{min} = 0.91$, $T_{max} = 0.98$
habitus	colorless needle
crystal size	$0.04 \times 0.05 \times 0.2\,mm^3$
lattice constants (from 21059 reflections	
with $2.8° < \theta < 68.1°$)	$a = 5.61656(18)$ Å
	$b = 13.3920(5)$ Å

	$c = 31.5475(11)$ Å
	$V = 2372.91(14)$ Å^3
	$Z = 4$
	$F(000) = 936$
temperature	110 K
density (calculated)	1.216 g cm^{-3}
Data collection:	
diffractometer	STOE IPDS 2T
radiation	Cu-K_α IµS mirror
scan type	ω scans
scan width	1°
scan range	$2° \leq \theta \leq 68.2°$
	$-6 \leq h \leq 6$
	$-16 \leq k \leq 16$
	$-37 \leq l \leq 24$
collected reflections	17495
independent reflections	4263 ($R_{\text{int}} = 0.033$)
observed reflections	3283 ($I/\sigma_I \geq 2$)
Data correction, structure solution and refinement:	
corrections	Lorentz and polarization correction
solution	SHELXT-2014 [1]
refinement	SHELXL-2014 [2] (full-matrix method); 317 refined parameters, weighted refinement: $w = [\sigma^2(F_o^2) + (0.0975 \cdot P)^2 + 2.22 \cdot P]^{-1}$ with $P = \frac{1}{3}[\max(F_o^2, 0) + 2 \cdot F_c^2]$; hydrogen atoms generated with appropriate geometric constraints and allowed to ride on their respective parent atoms, NH localized and refined, non-hydrogen atoms anisotropically refined
discrepancy factors	$wR_2 = 0.2109$
	$R_1 = 0.0657$ (observed reflections)
	$R_1 = 0.0923$ (all reflections)
goodness-of-fit (S)	1.121
Flack parameter (x)	$-0.069(136)$
maximum change of parameters	$0.001 \cdot esd$
maximum peak height in differential Fourier synthesis	$0.53\,e\,$Å^{-3}, $-0.30\,e\,$Å^{-3}
comment	ester oxygen atom and one methyl group are disordered

5 HPLC/ESI-MS^n

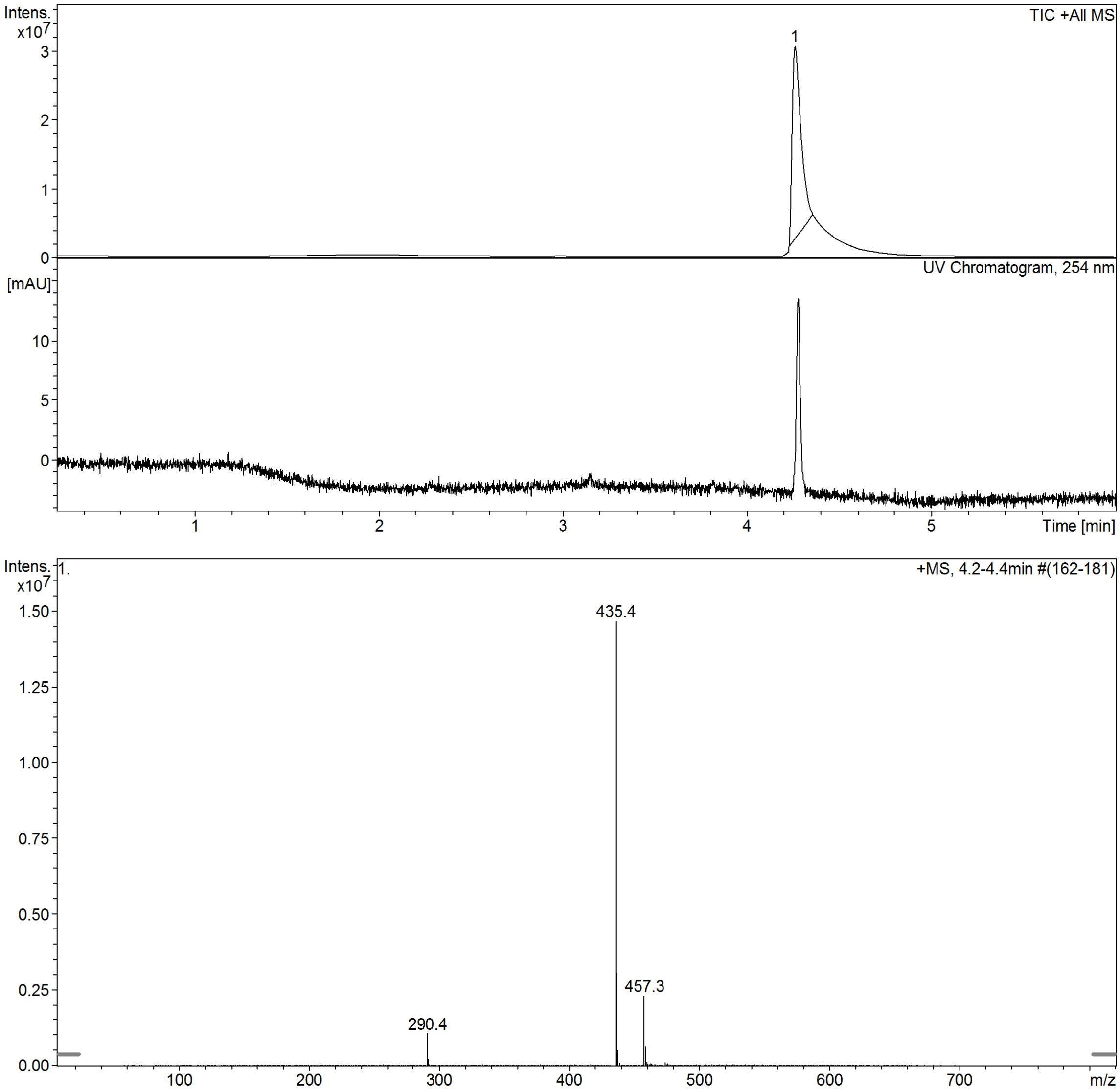

Figure 5.1: HPLC/ESI-MS of pure (*S*)-**3** from test purchase 1 (internet shop, Dec 2015).

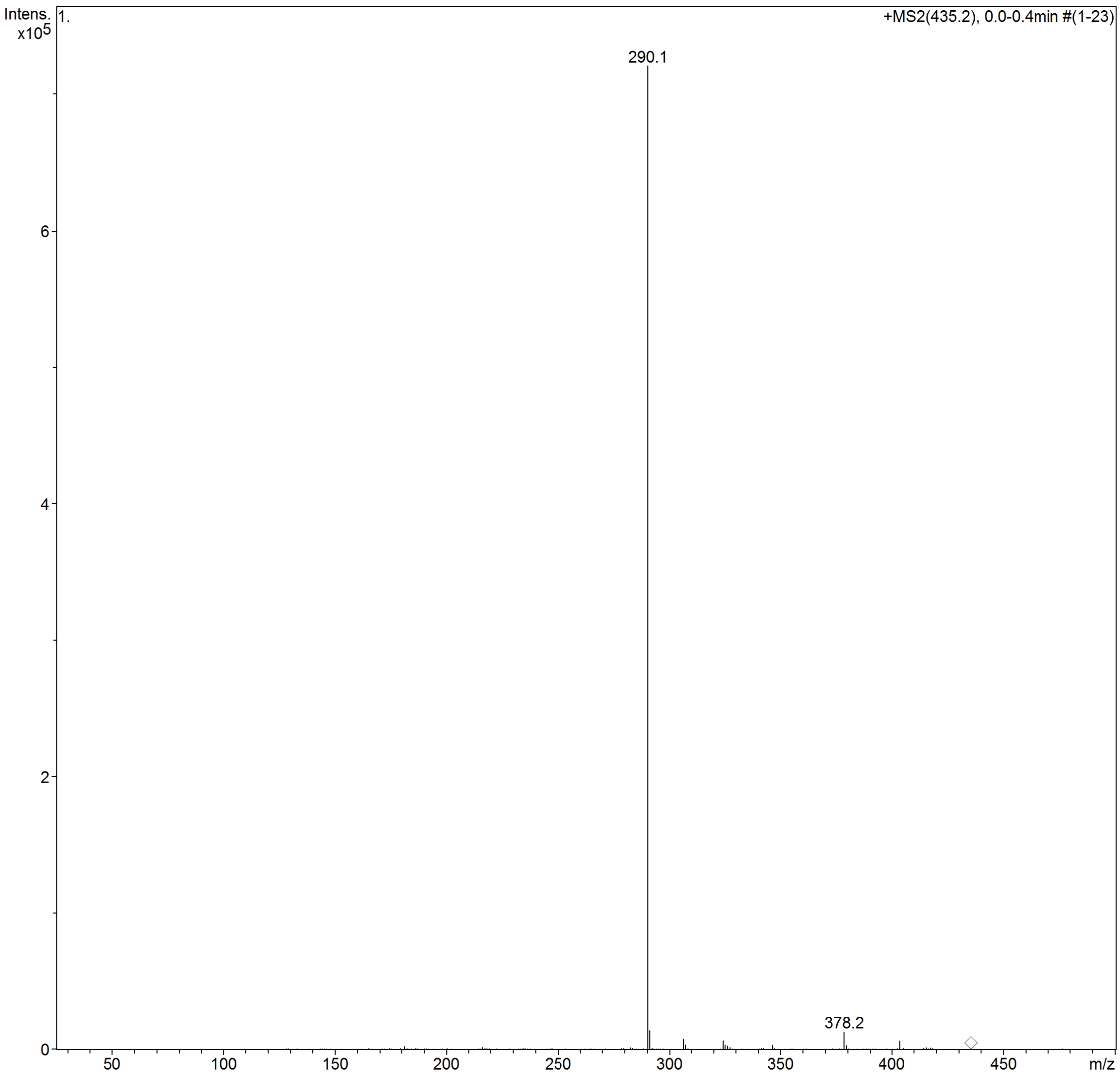

Figure 5.2: ESI-MS^2(435) of pure (*S*)-**3** from test purchase 1 (internet shop, Dec 2015).

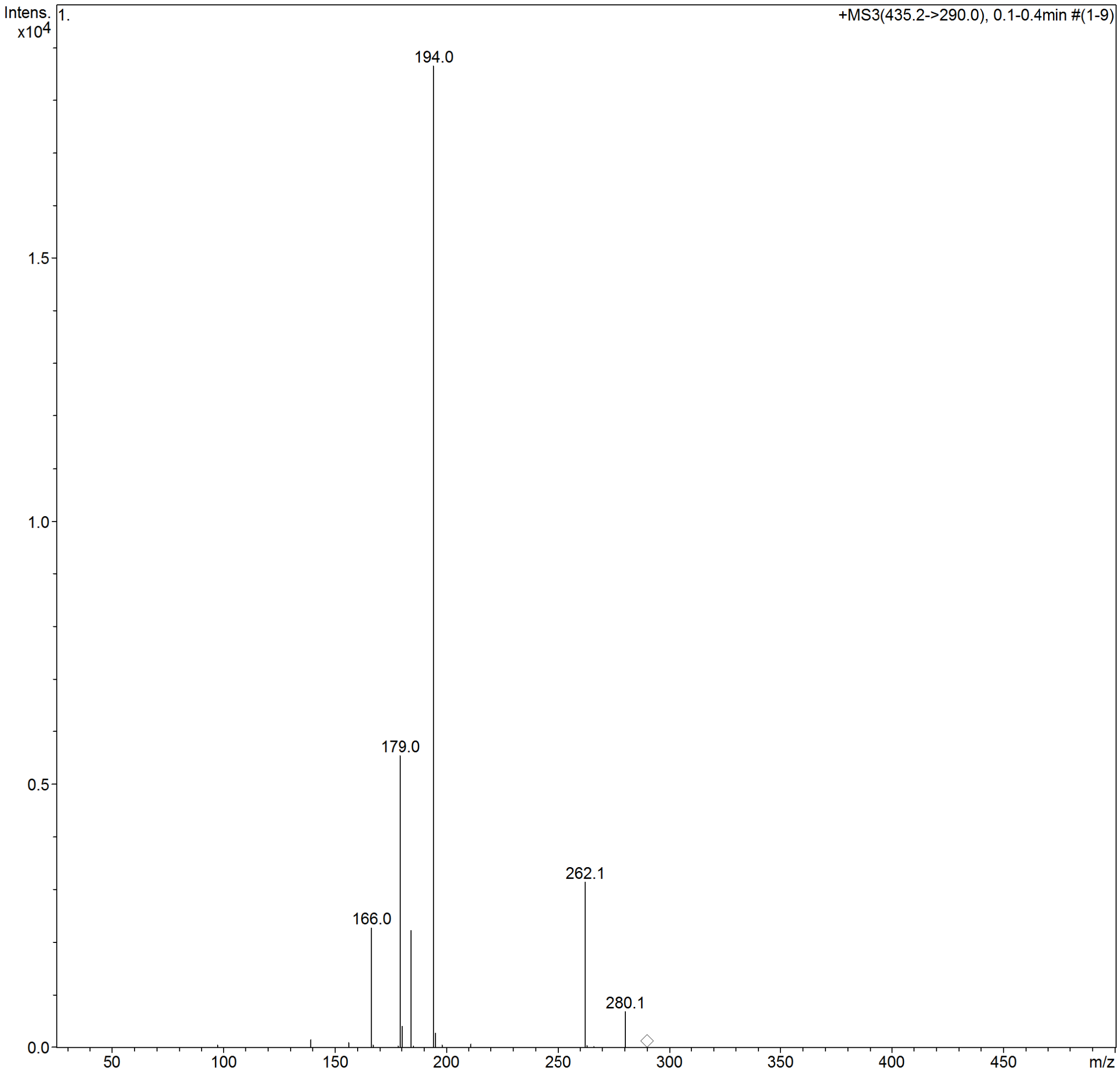

Figure 5.3: ESI-MS^3(435→290) of pure (S)-**3** from test purchase 1 (internet shop, Dec 2015).

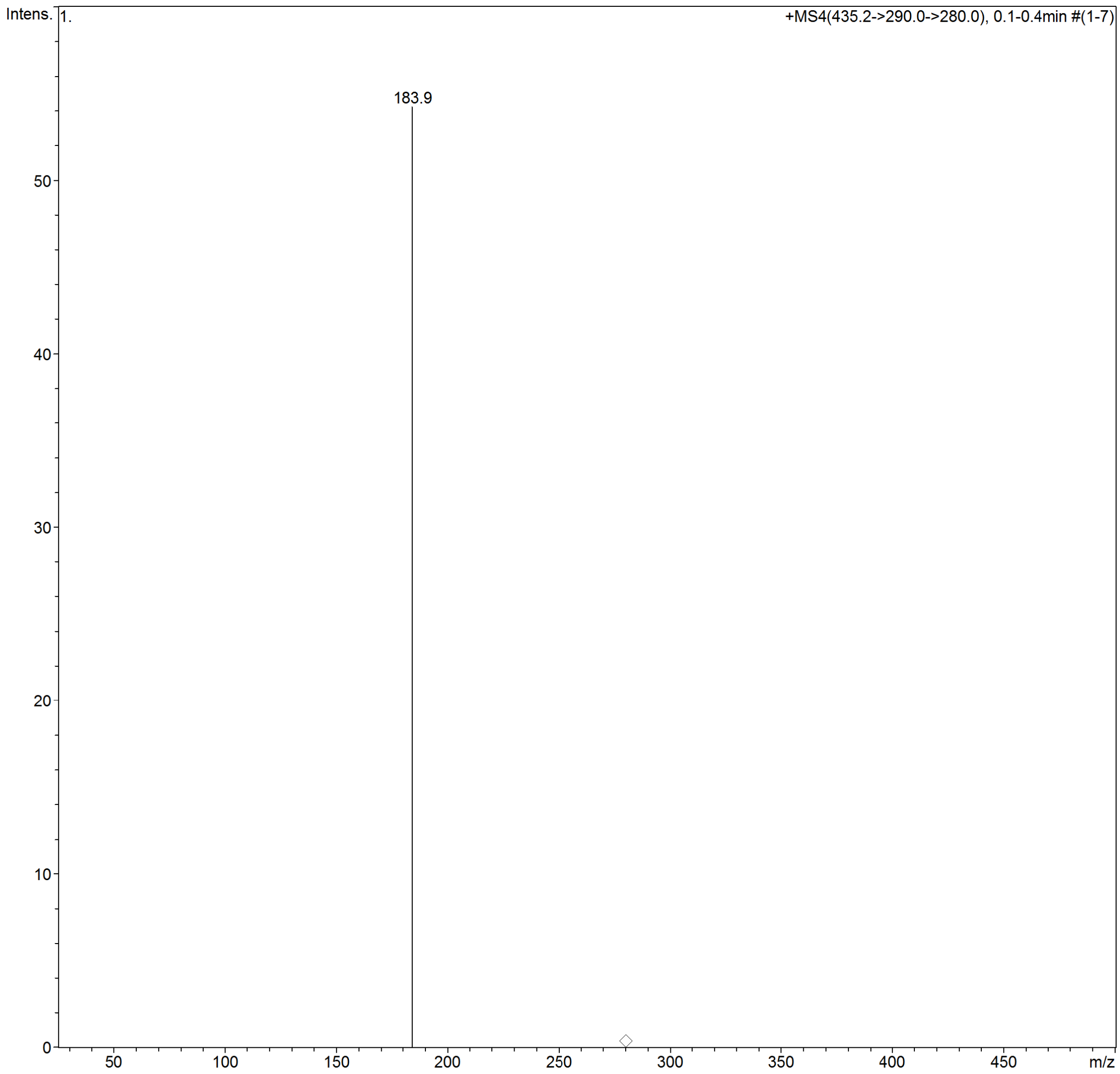

Figure 5.4: ESI-MS^4(435→290→280) of pure (*S*)-**3** from test purchase 1 (internet shop, Dec 2015).

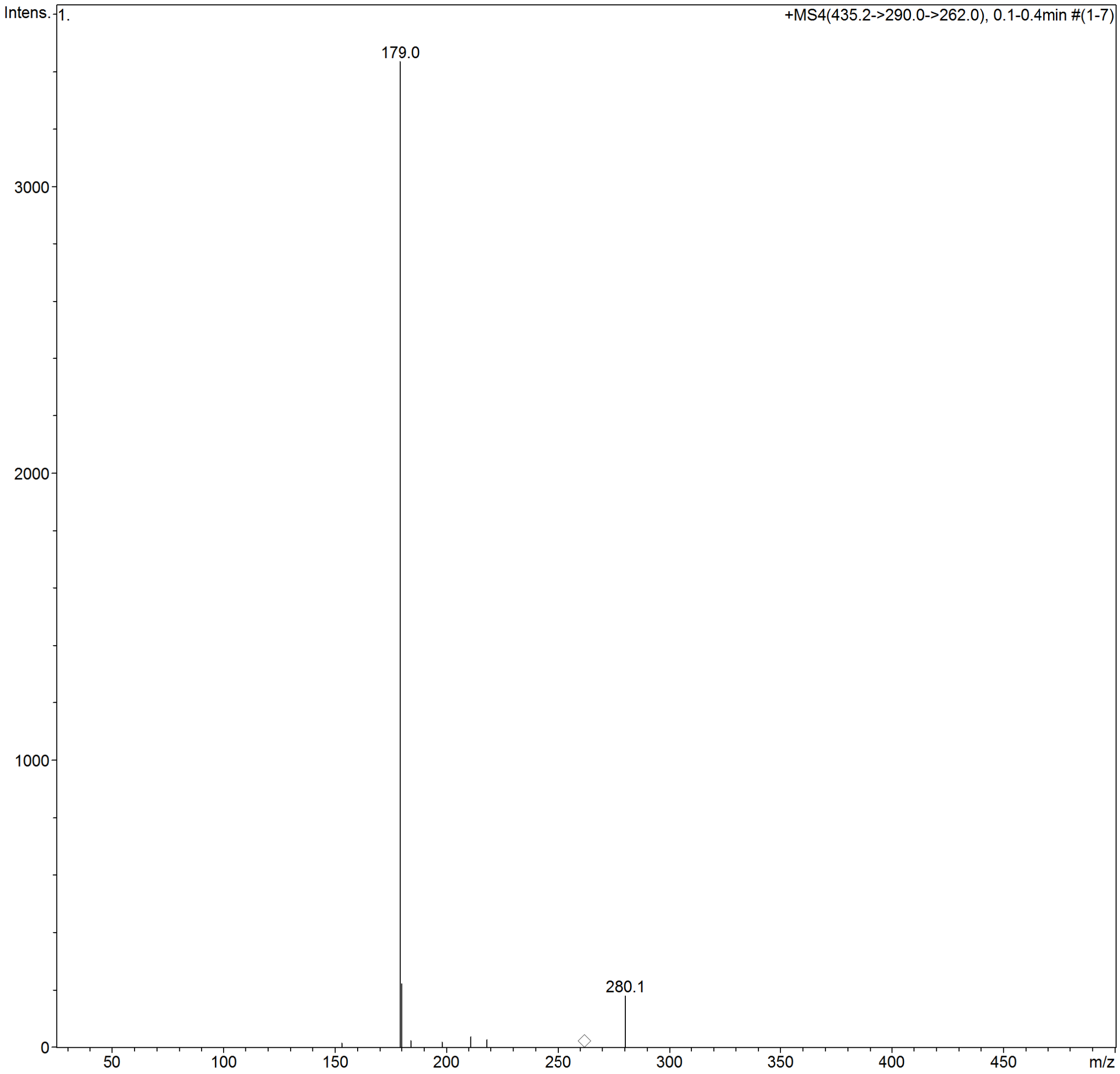

Figure 5.5: ESI-MS^4(435→290→262) of pure (*S*)-**3** from test purchase 1 (internet shop, Dec 2015).

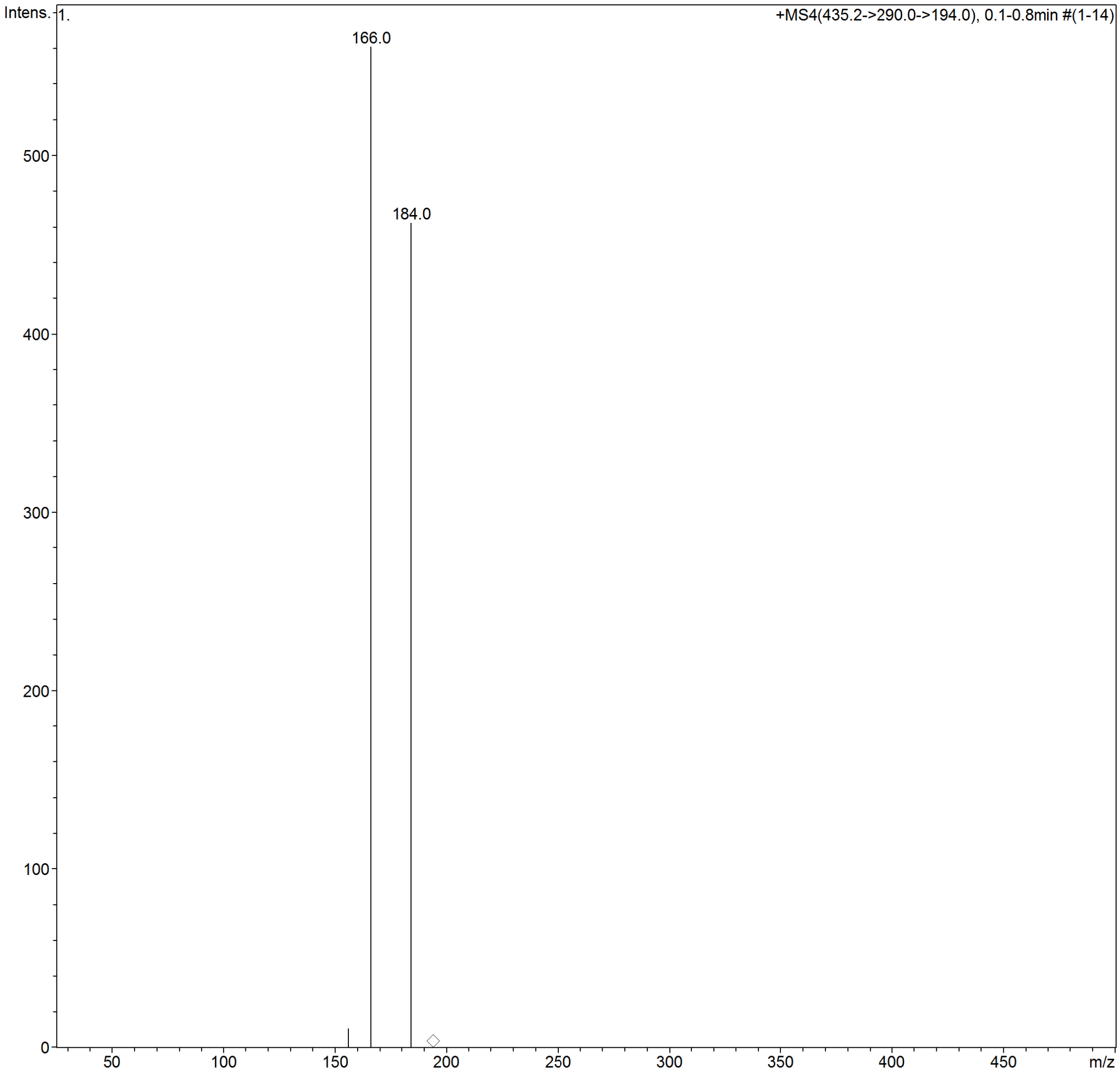

Figure 5.6: ESI-MS^4(435→290→194) of pure (*S*)-**3** from test purchase 1 (internet shop, Dec 2015).

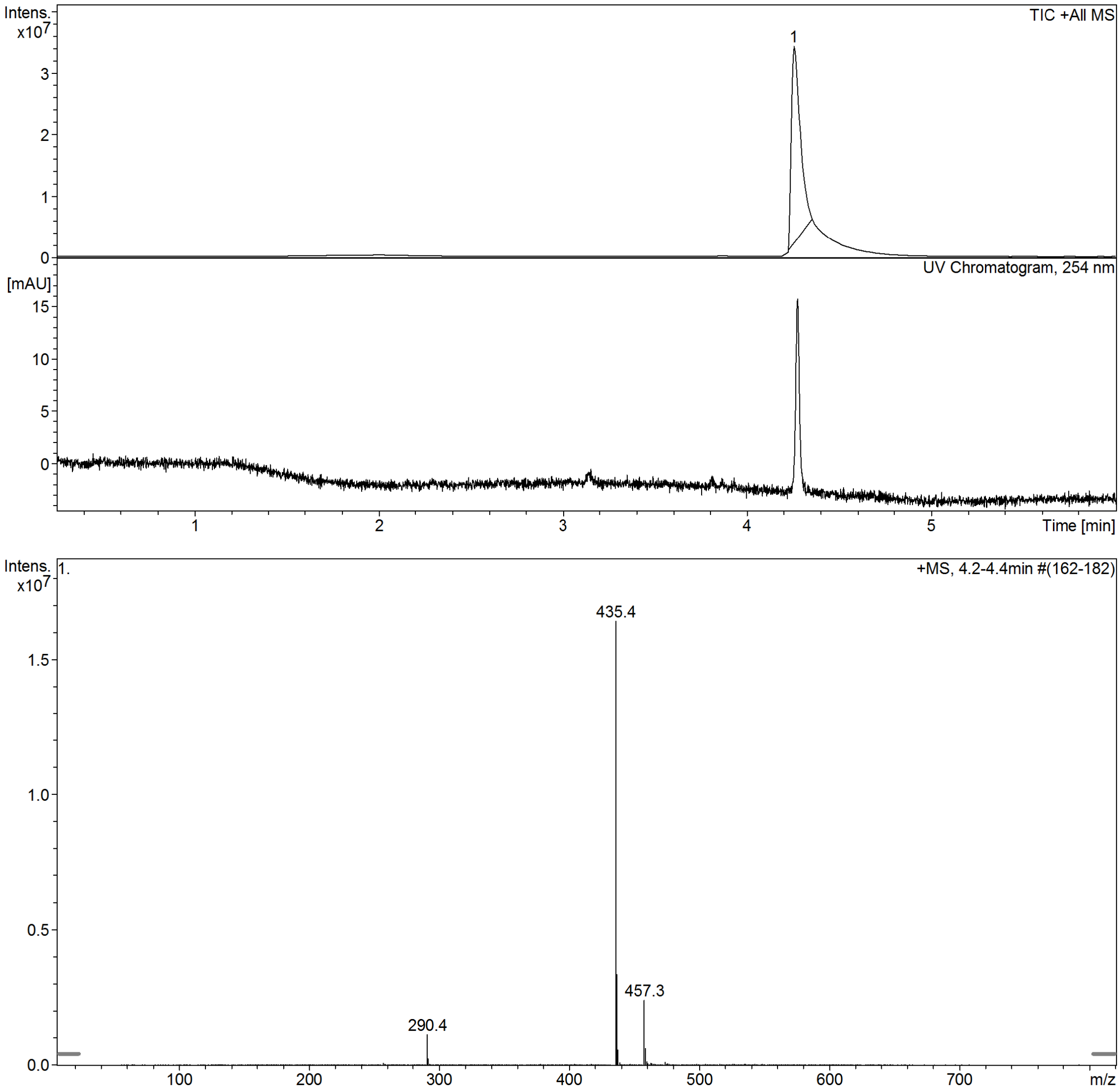

Figure 5.7: HPLC/ESI-MS of (R/S)-**3** after treatment of pure (S)-**3** from test purchase 1 (internet shop, Dec 2015) with NaOMe/MeOH.

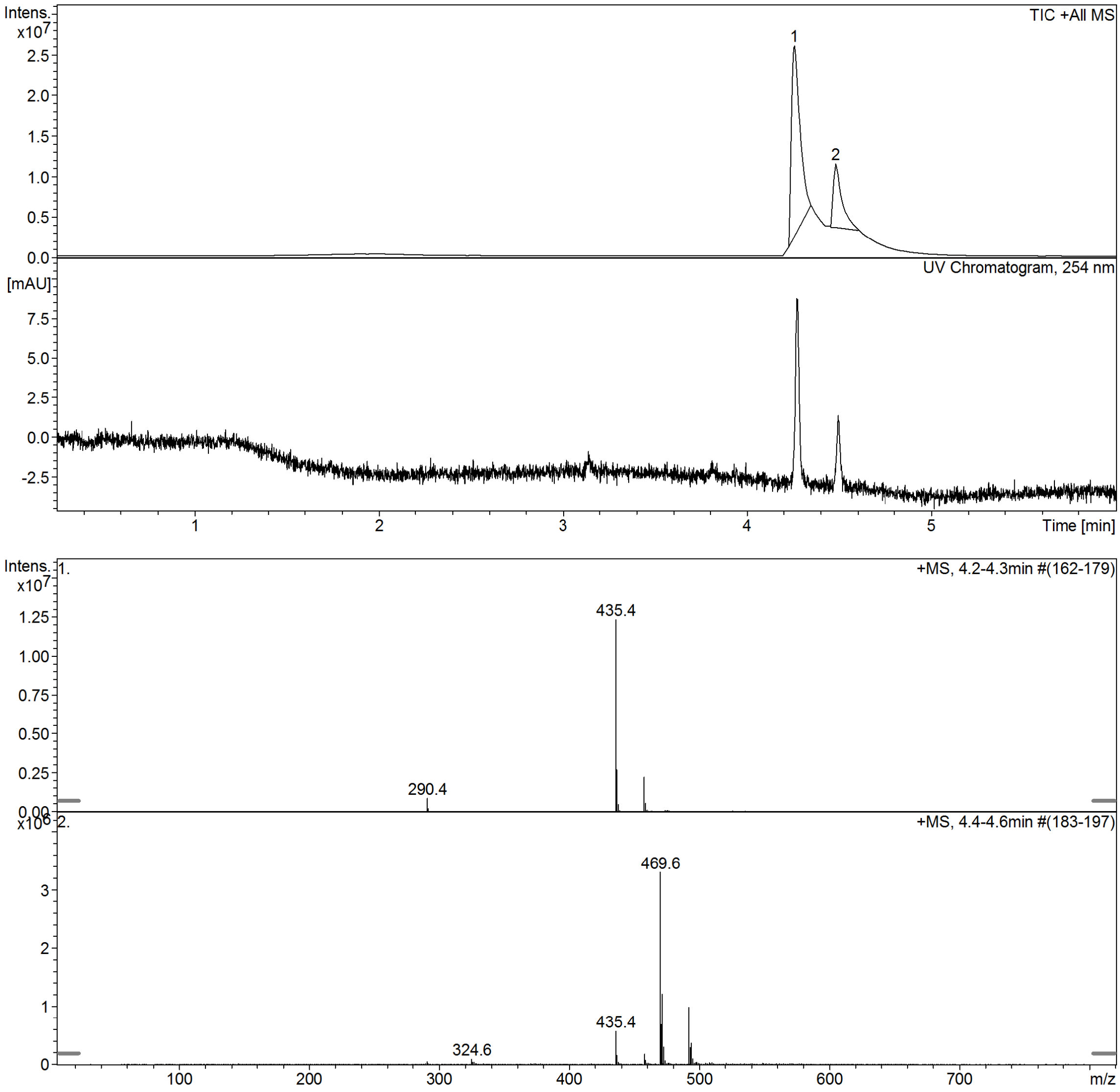

Figure 5.8: HPLC/ESI-MS of “pure” (*S*)-**3** from test purchase 2 (internet shop, May 2016, contaminant: chloro-MDMB-CHMCZCA).

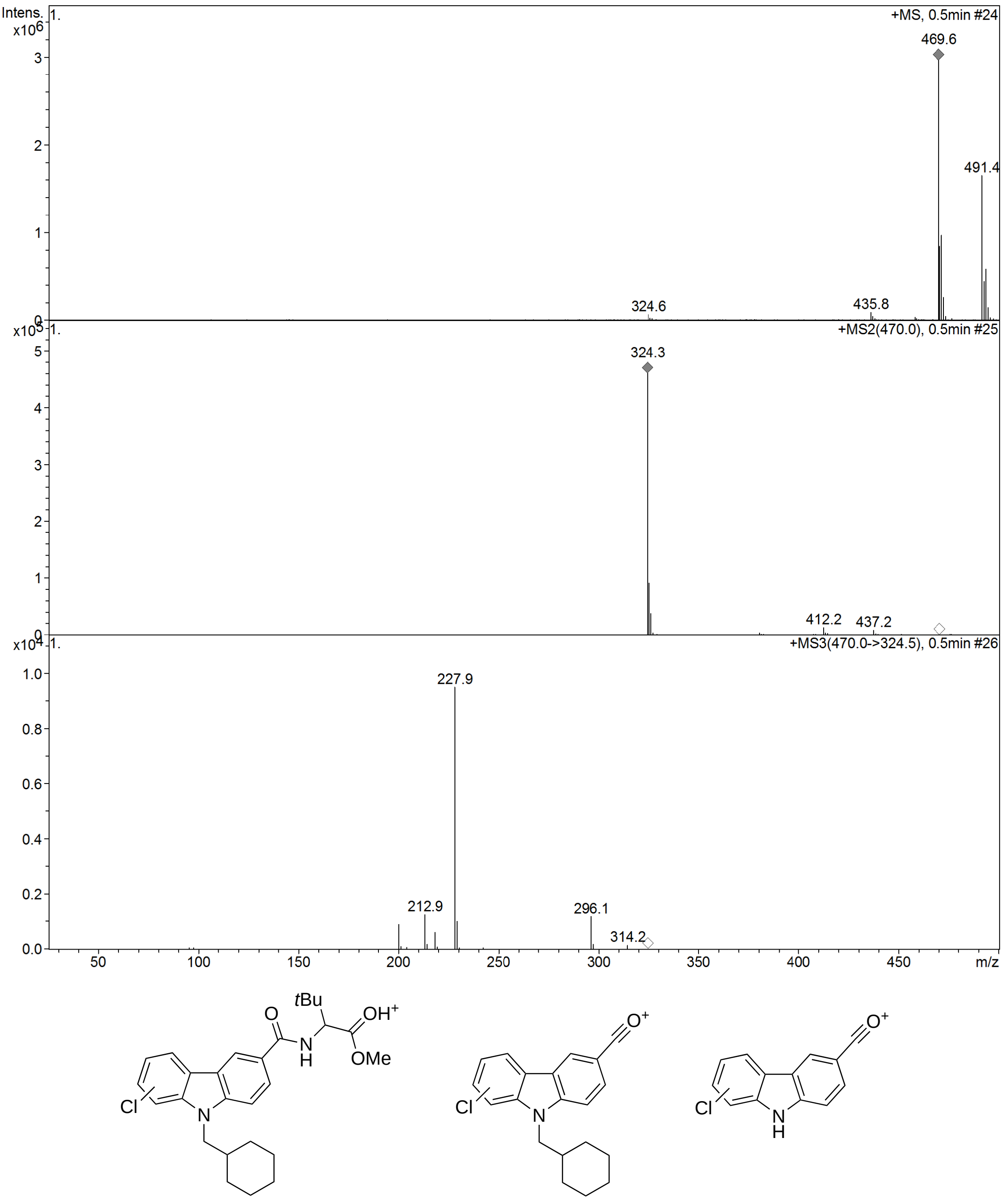

469 324 228

Figure 5.9: ESI-MS^n of chloro-MDMB-CHMCZCA contained as impurity in "pure" (S)-**3** from test purchase 2 (internet shop, May 2016).

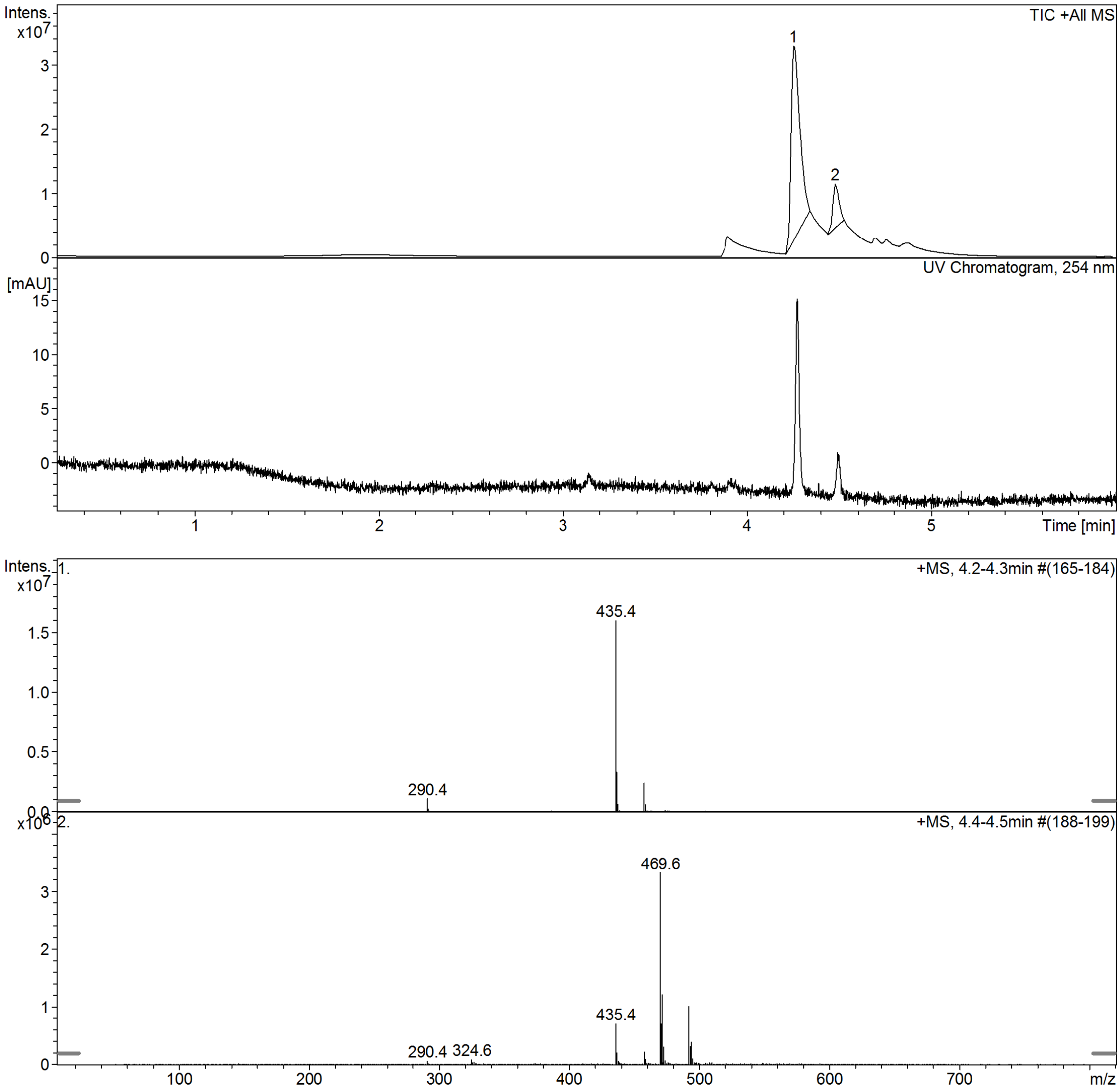

Figure 5.10: HPLC/ESI-MS of extracted (*S*)-**3** from test purchase 3 (hashish-like resin, Jan 2016, contaminant: chloro-MDMB-CHMCZCA) .

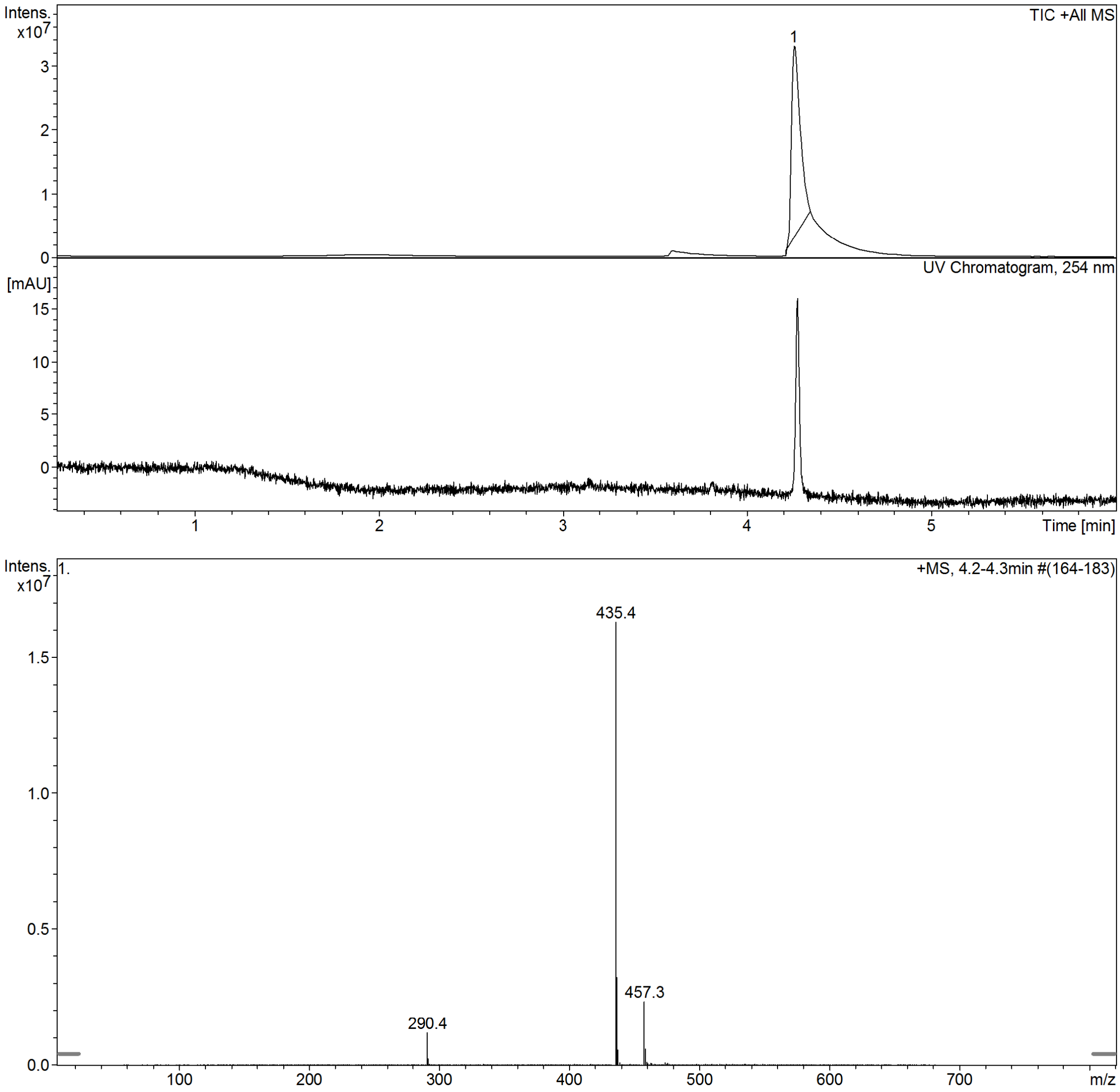

Figure 5.11: HPLC/ESI-MS of extracted (S)-**3** from test purchase 4 (herbal mixture, Mar 2016).

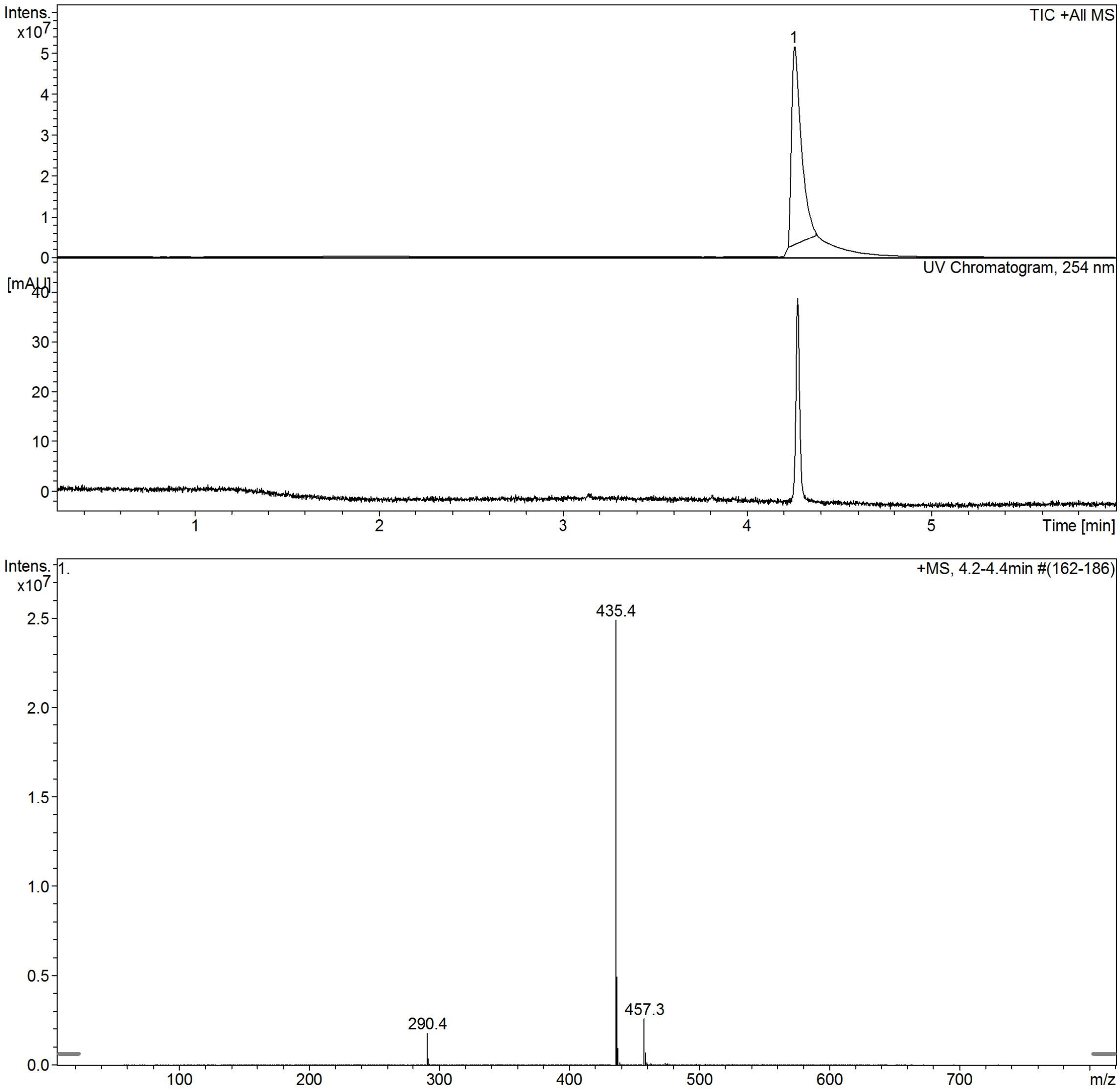

Figure 5.12: HPLC/ESI-MS of extracted (*S*)-**3** from test purchase 5 (herbal mixture, Mar 2016).

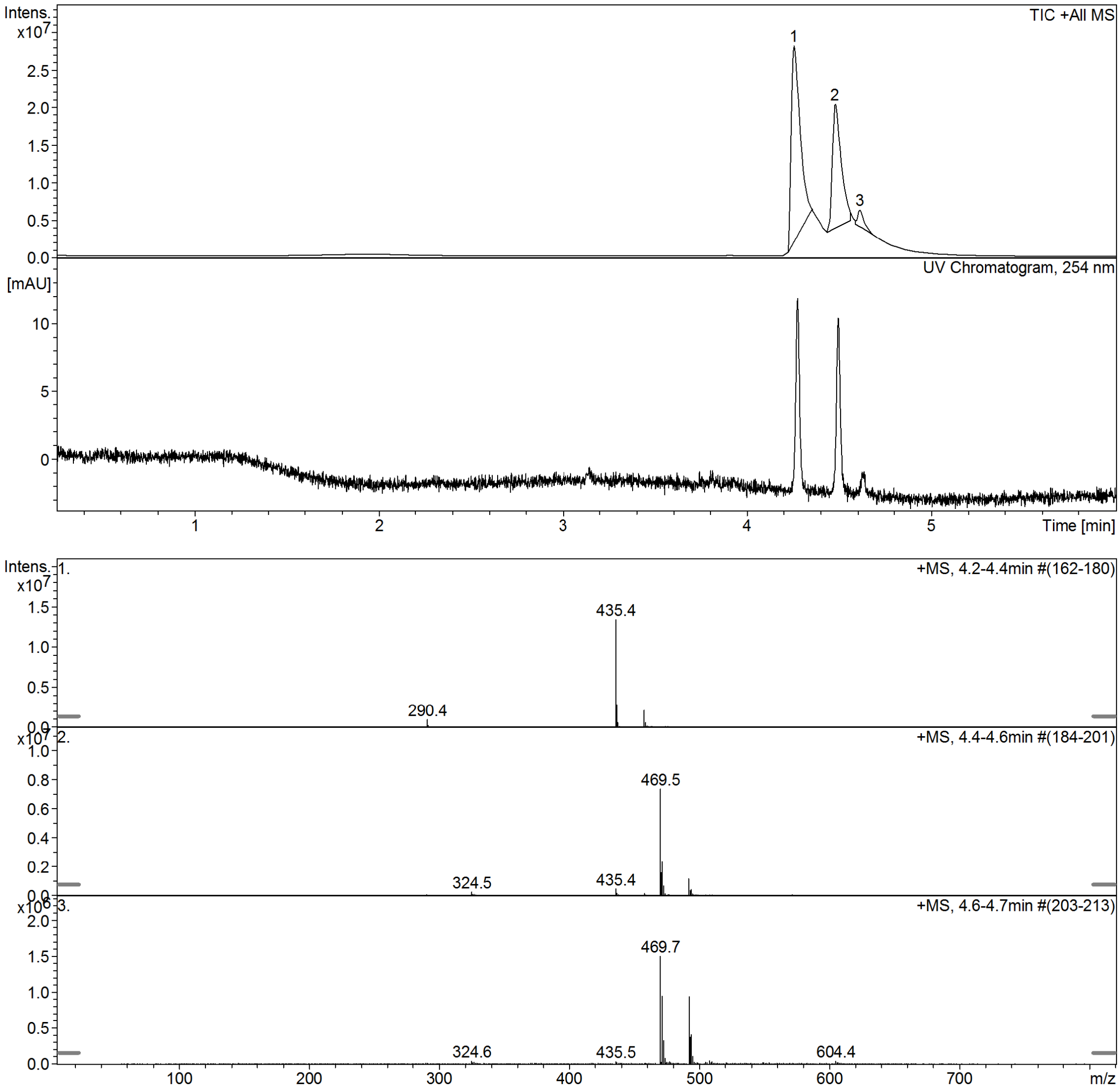

Figure 5.13: HPLC/ESI-MS of extracted (*S*)-**3** from police seizure 1 (herbal mixture, Feb 2015, contaminant: chloro-MDMB-CHMCZCA).

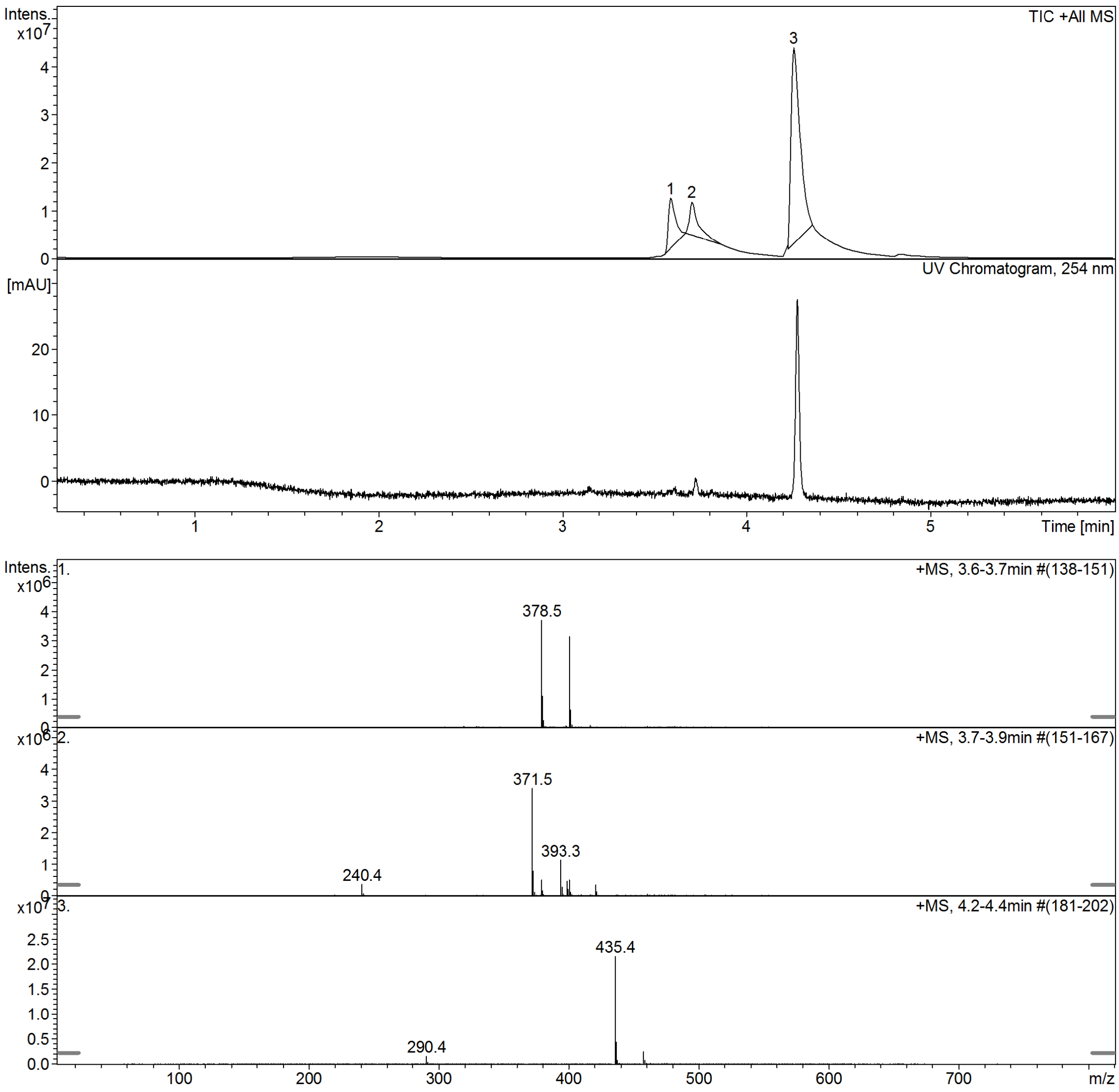

Figure 5.14: HPLC/ESI-MS of extracted (*S*)-**3** from police seizure 2 (herbal mixture, Feb 2015, contaminants: 5F-ADB/5F-MDMB-PINACA and MMB-CHMICA).

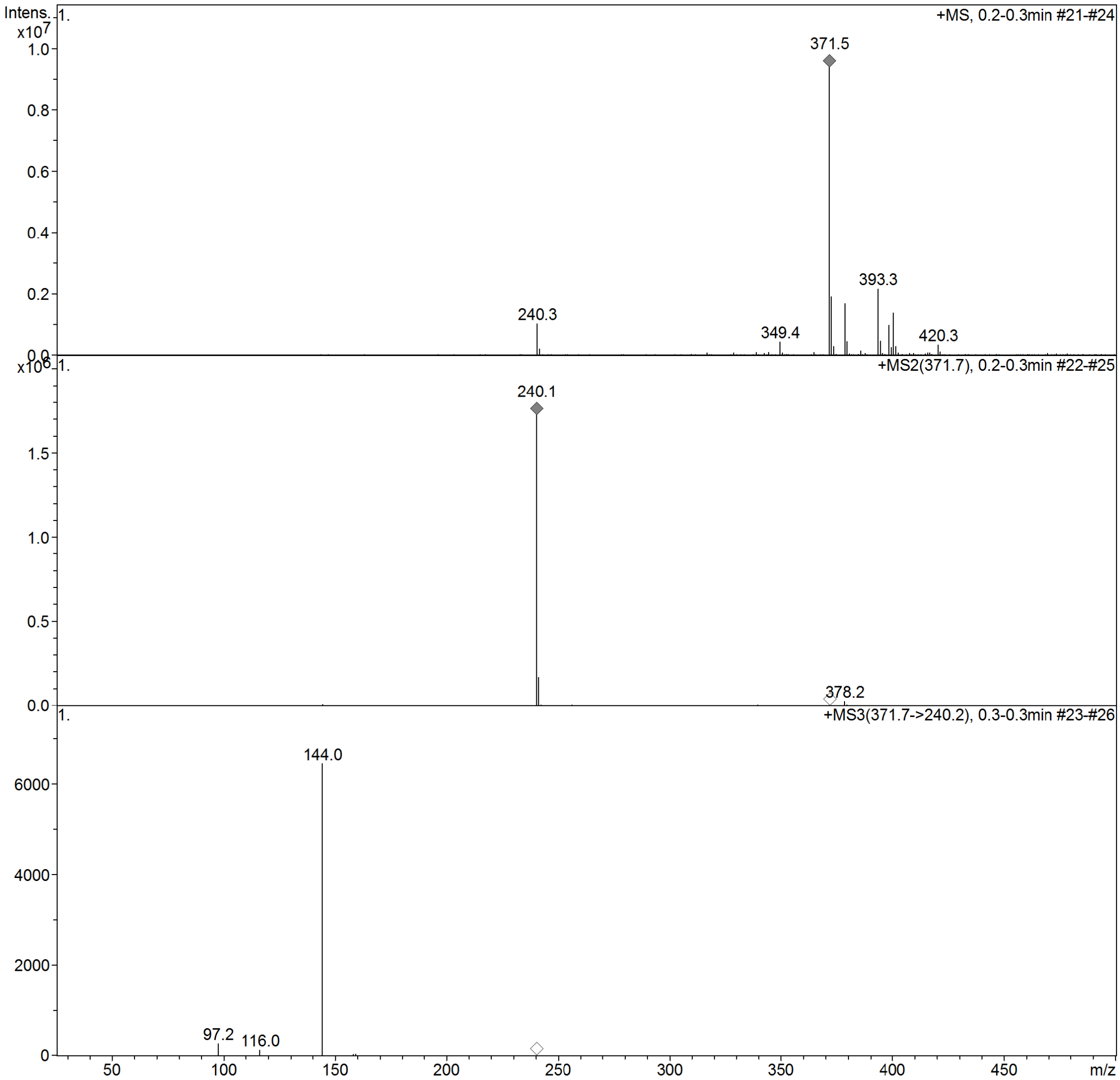

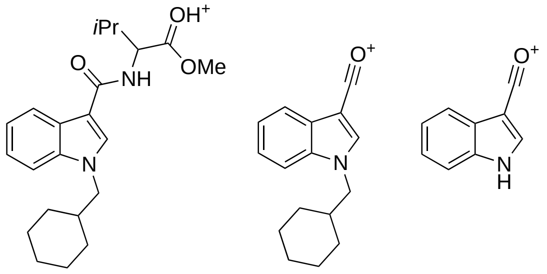

371 240 144

Figure 5.15: ESI-MS^n of MMB-CHMICA contained as impurity in extracted (*S*)-**3** from police seizure 2 (herbal mixture, Mar 2015).

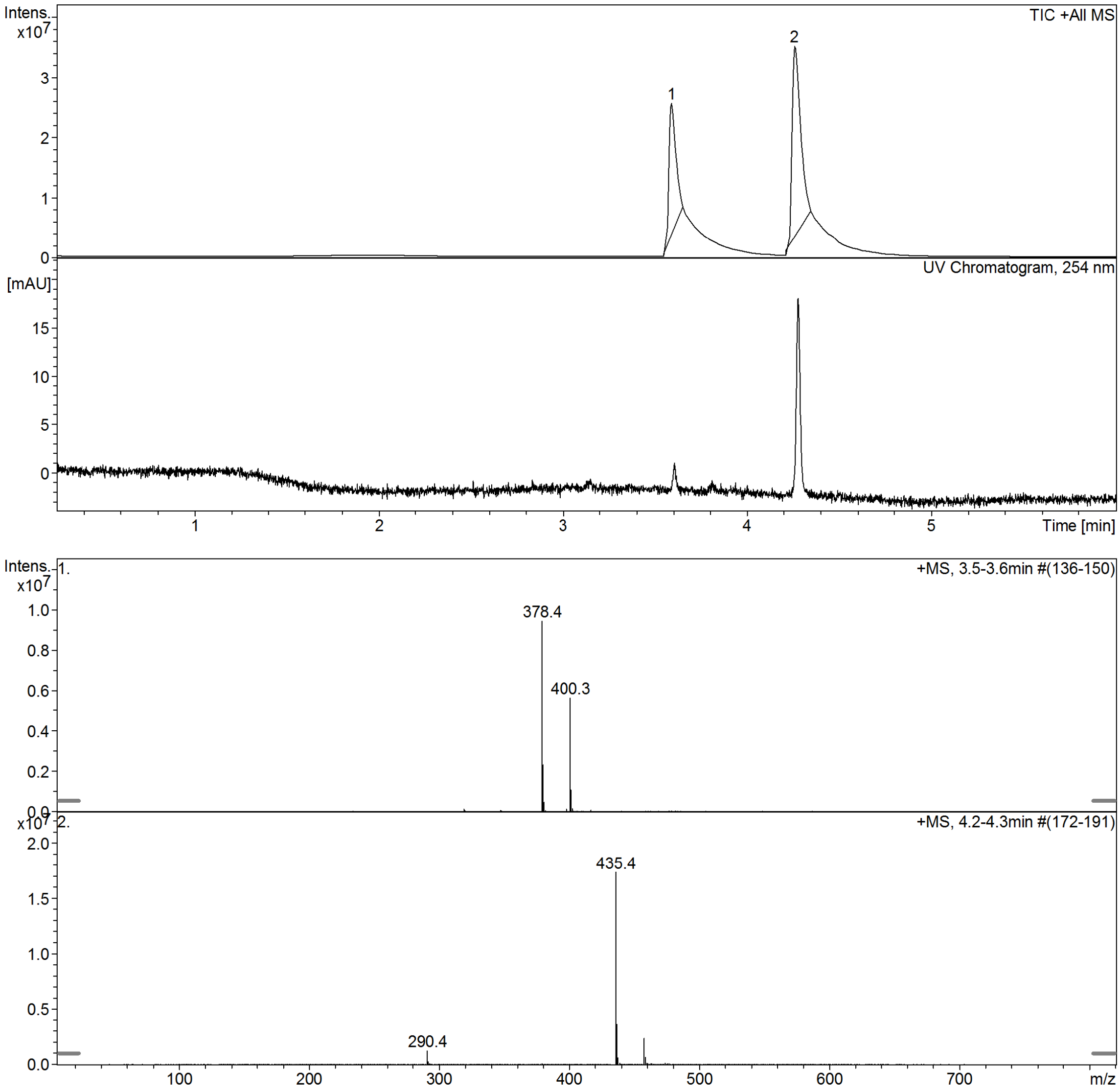

Figure 5.16: HPLC/ESI-MS of extracted (S)-**3** from police seizure 3 (herbal mixture, Aug 2016, contaminant: 5F-ADB/5F-MDMB-PINACA).

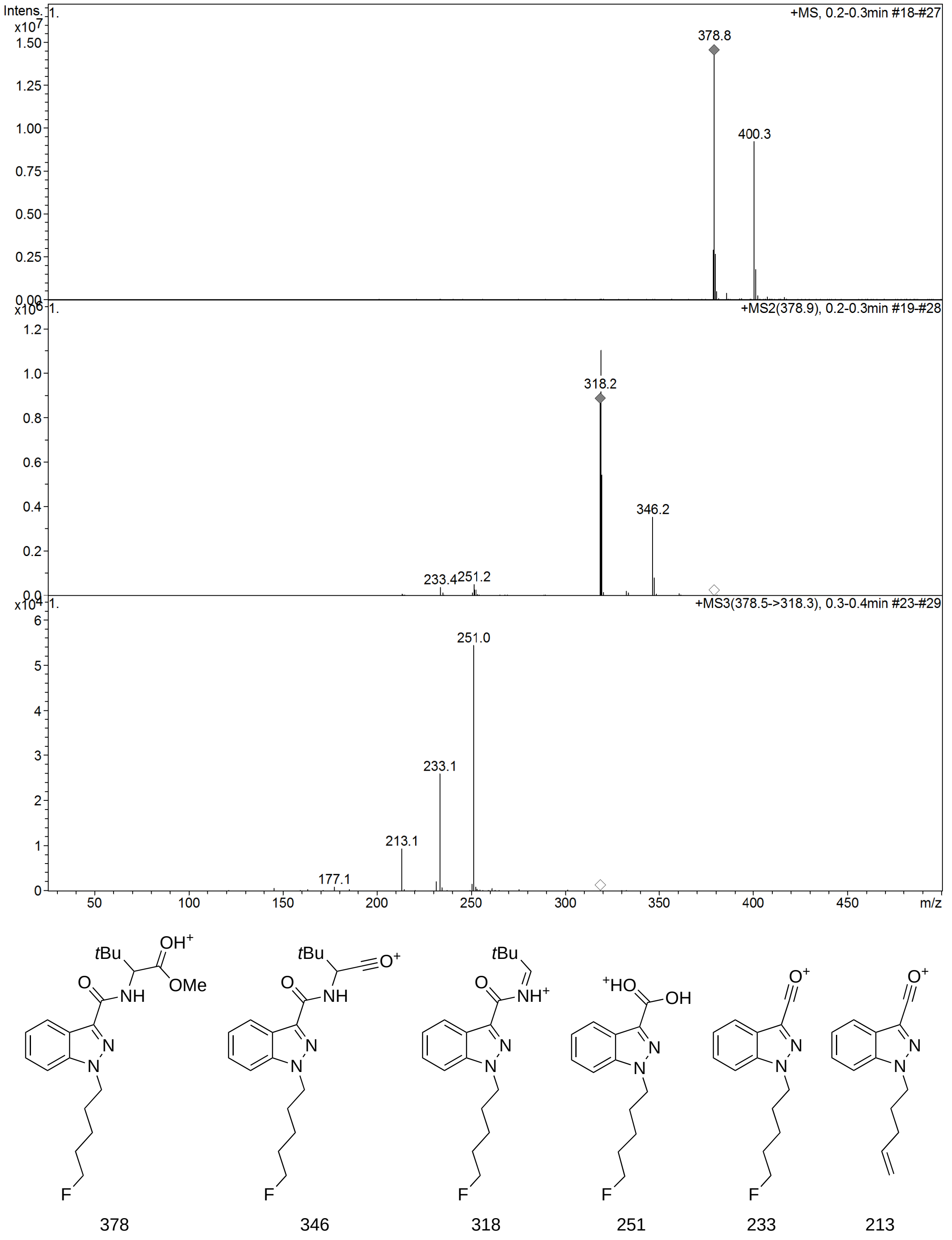

Figure 5.17: ESI-MS^n of 5F-ADB/5F-MDMB-PINACA contained as impurity in extracted (S)-**3** from police seizure 3 (herbal mixture, Aug 2016).

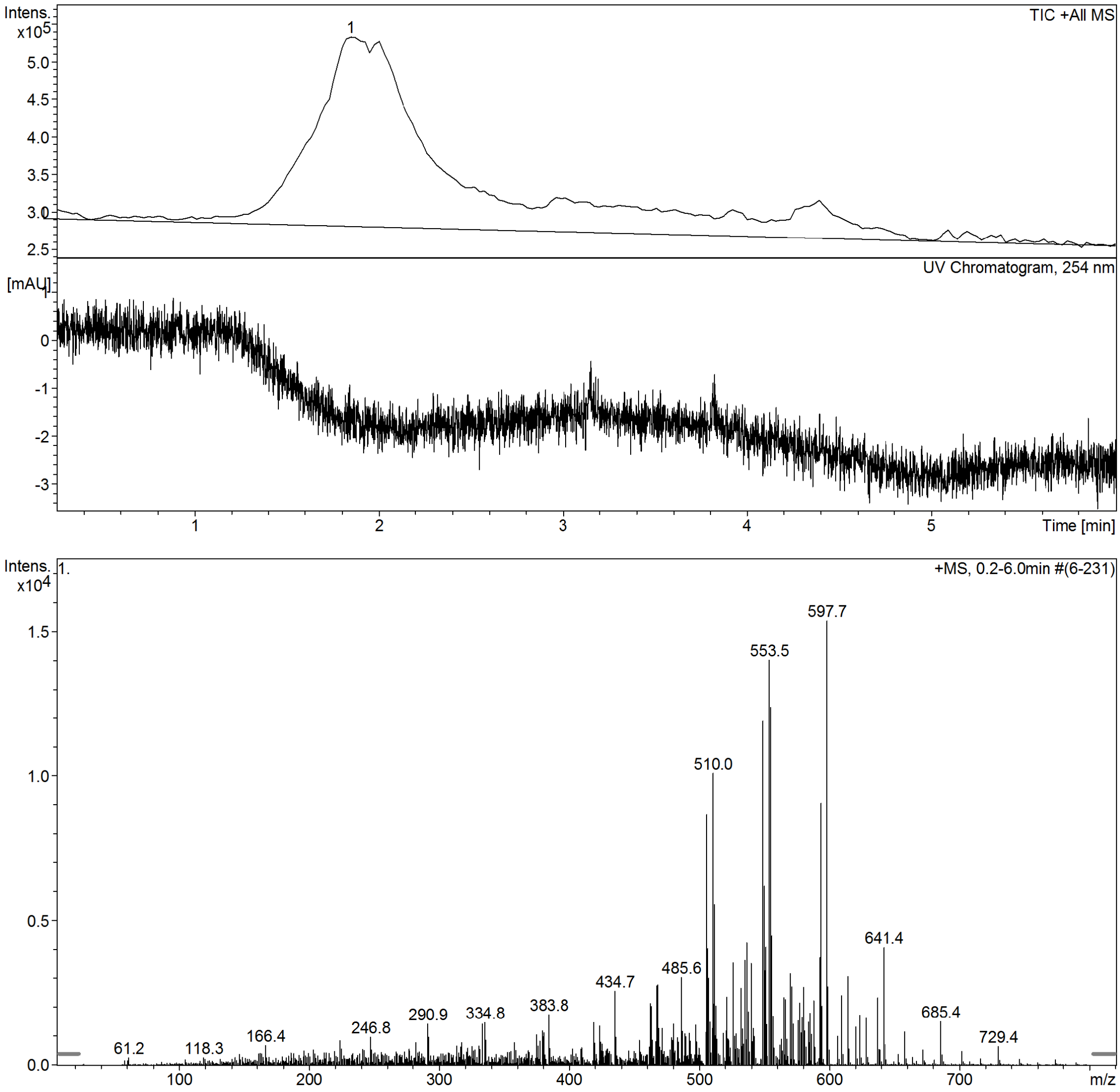

Figure 5.18: HPLC/ESI-MS of a blank sample.

6 Chiral HPLC

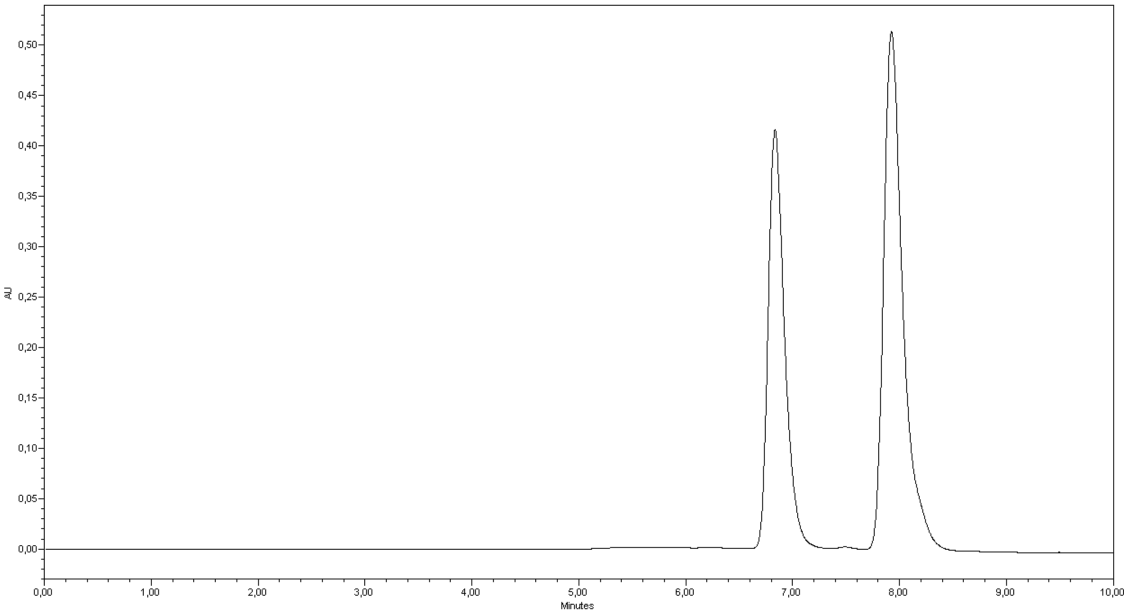

Figure 6.1: Chiral HPLC of (R/S)-**3**.

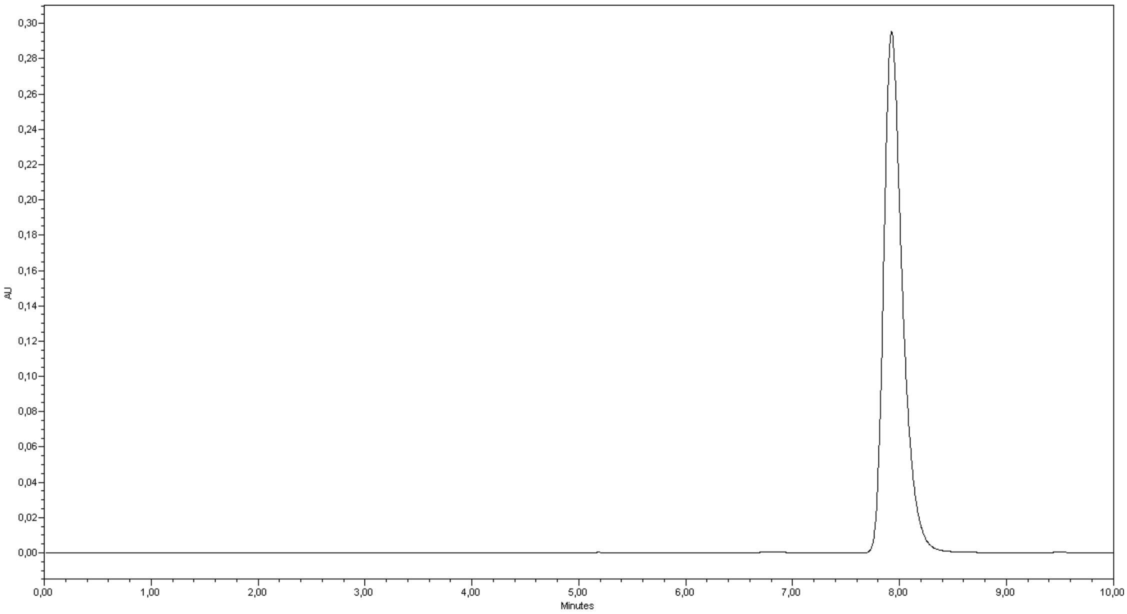

Figure 6.2: Chiral HPLC of pure (S)-**3** from test purchase 1 (internet shop, Dec 2015).

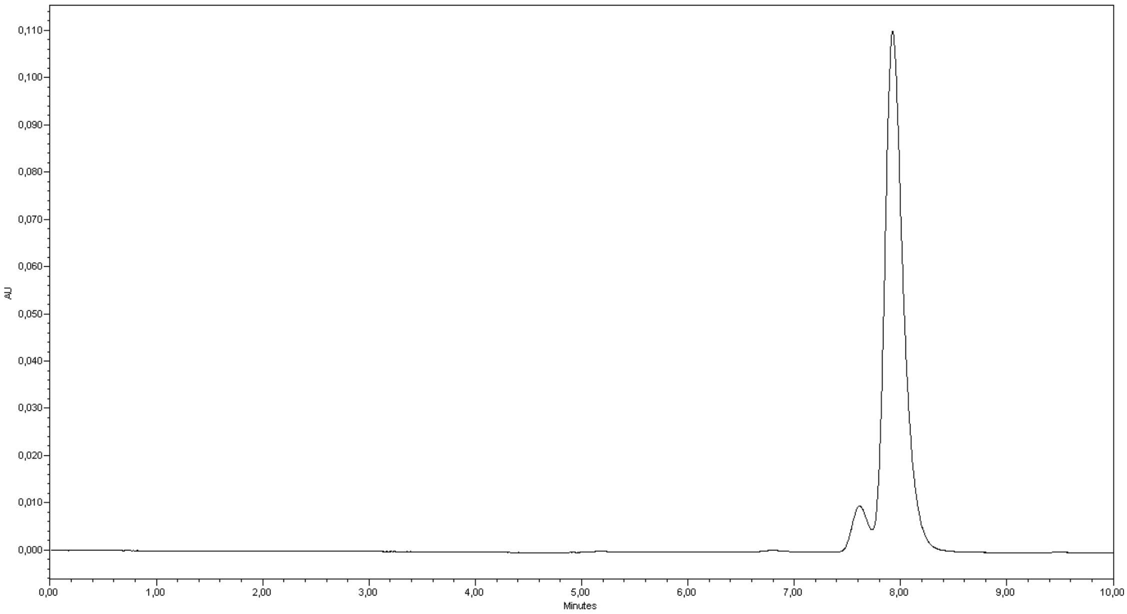

Figure 6.3: Chiral HPLC of “pure” (S)-**3** from test purchase 2 (internet shop, May 2016).

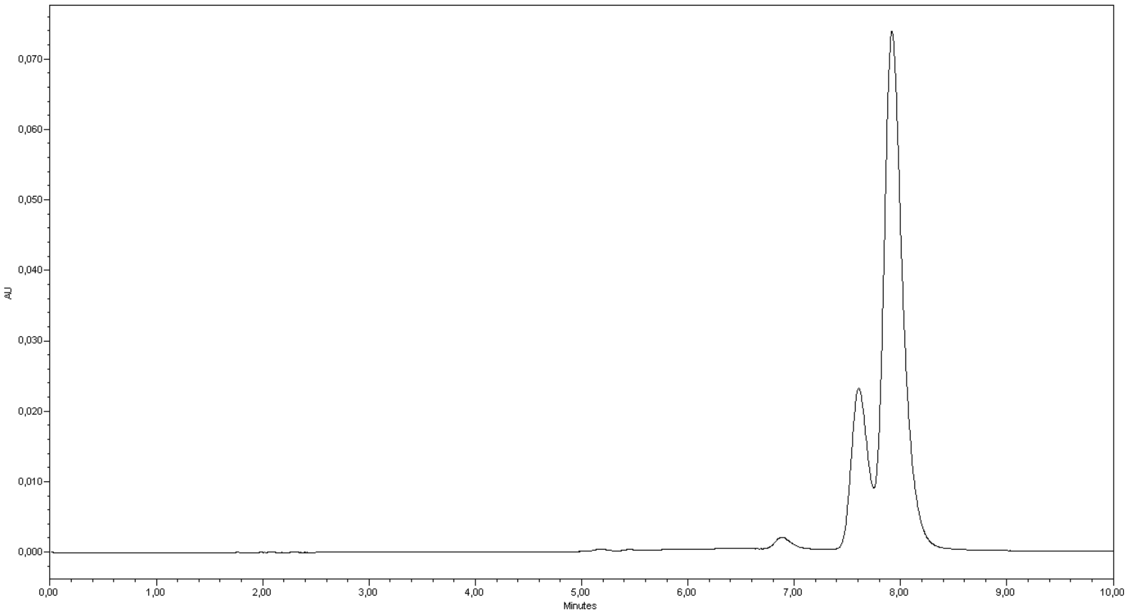

Figure 6.4: Chiral HPLC of extracted (S)-**3** from test purchase 3 (hashish-like resin, Jan 2016).

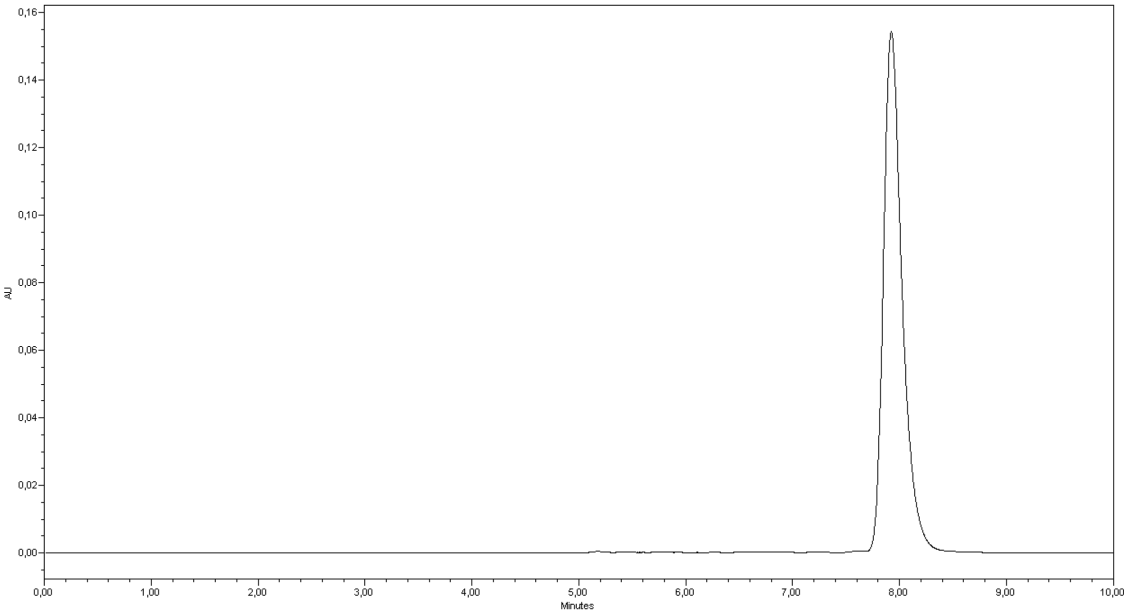

Figure 6.5: Chiral HPLC of extracted (*S*)-**3** from test purchase 4 (herbal mixture, Mar 2016).

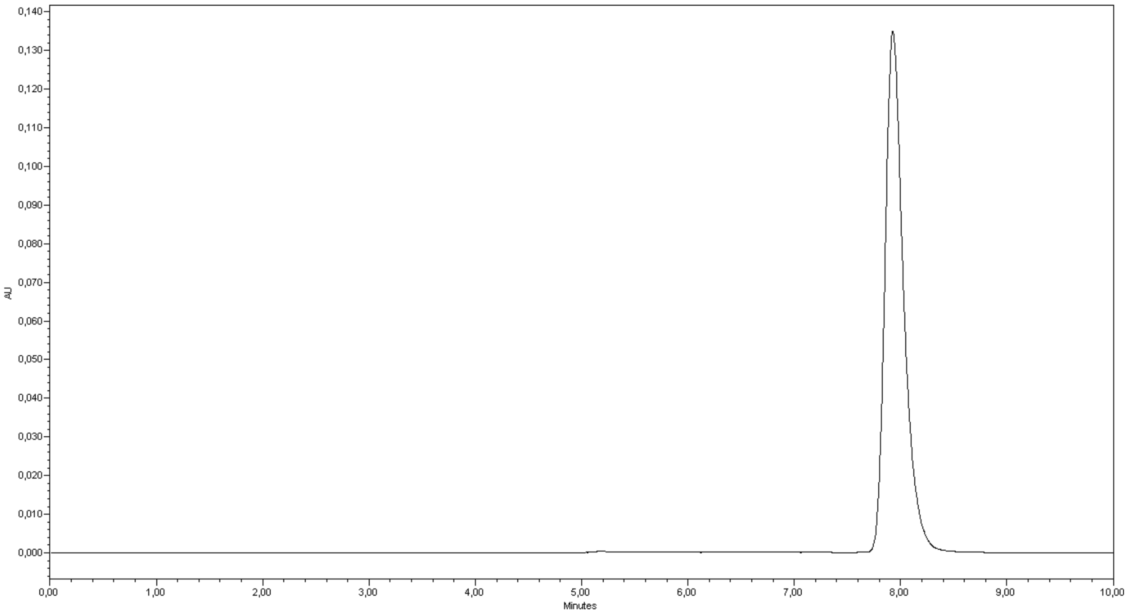

Figure 6.6: Chiral HPLC of extracted (*S*)-**3** from test purchase 5 (herbal mixture, Mar 2016).

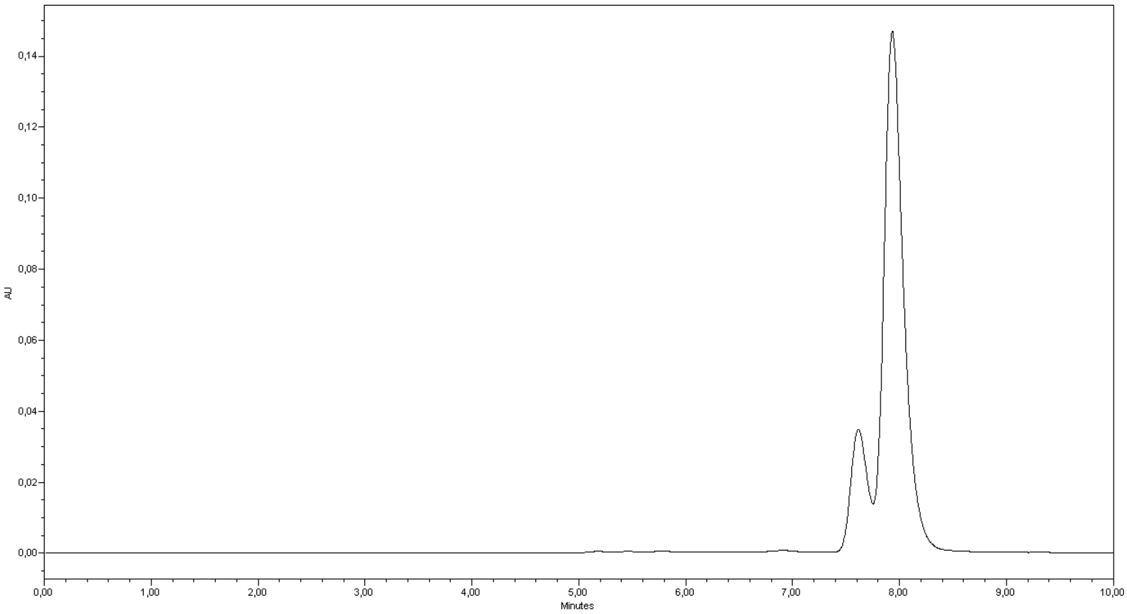

Figure 6.7: Chiral HPLC of extracted (*S*)-**3** from police seizure 1 (herbal mixture, Feb 2015).

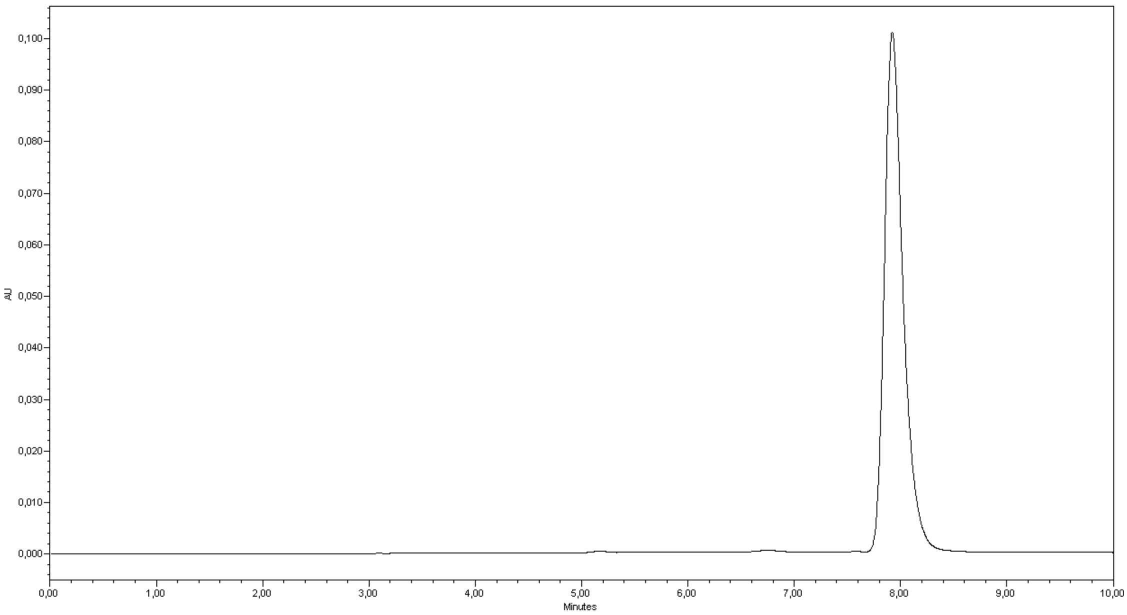

Figure 6.8: Chiral HPLC of extracted (*S*)-**3** from police seizure 2 (herbal mixture, Mar 2015).

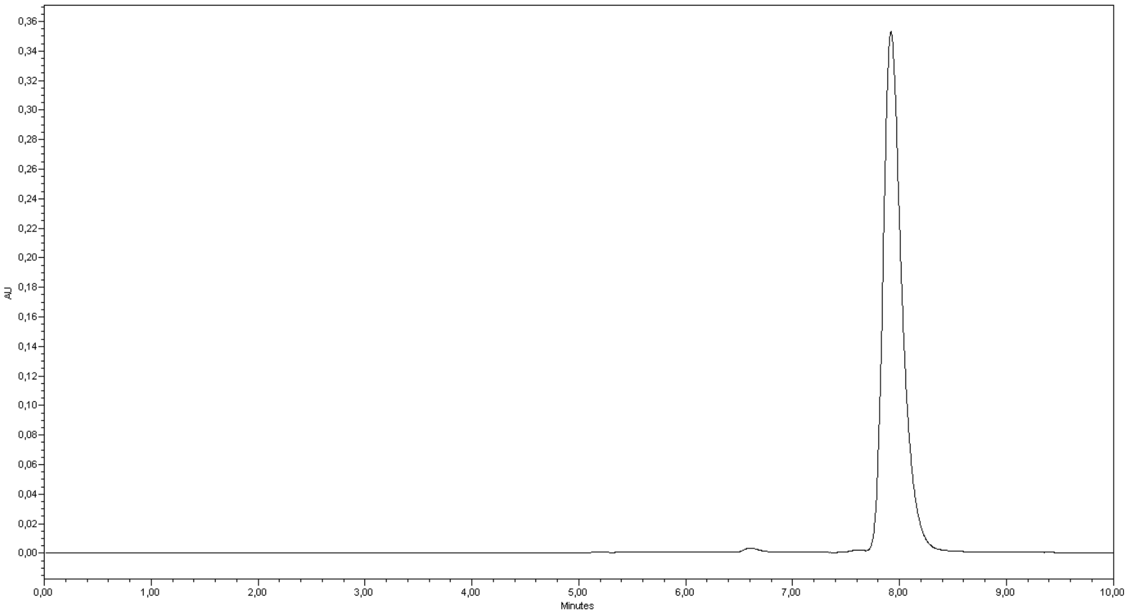

Figure 6.9: Chiral HPLC of extracted (*S*)-**3** from police seizure 3 (herbal mixture, Aug 2016).

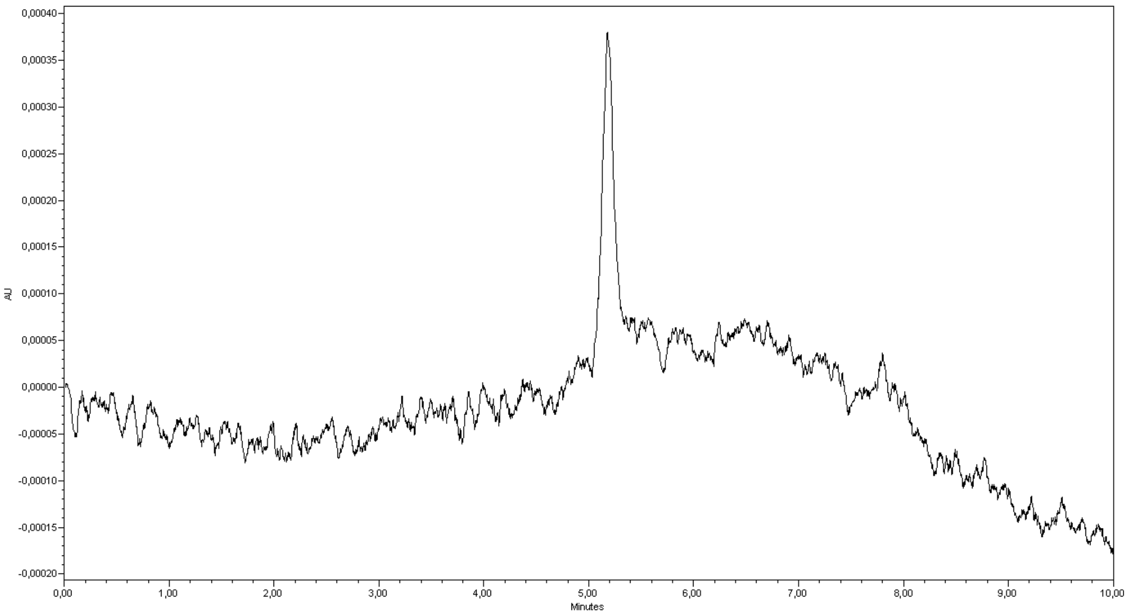

Figure 6.10: Chiral HPLC of a blank sample.

7 Computational Chemistry

7.1 Keyword Lines

- conformational analysis using MMFF or PM6 (Spartan):

```
SEARCHMETHOD=SPARSE FINDBOATS KEEPALL CONF_SELECTION_RULE=5
```

- semiempirical geometry optimization (Gaussian):

```
#p opt=tight pm6
```

- DFT geometry optimization at double-ζ level (Gaussian):

```
#p opt=tight b3pw91 6-31g(d) scrf=(iefpcm,solvent=chloroform) int=grid=ultrafine
```

- DFT geometry optimization and frequency analysis at triple-ζ level in chloroform (Gaussian):

```
#p opt=tight freq=vcd b3pw91 6-311g(d,p) scrf=(iefpcm,solvent=chloroform)
int=grid=ultrafine
```

- DFT geometry optimization and frequency analysis at triple-ζ level in acetonitrile (Gaussian):

```
#p opt=tight freq=vcd b3pw91 6-311g(d,p) scrf=(iefpcm,solvent=acetonitrile)
int=grid=ultrafine
```

- TD-DFT/TDA calculations (Gaussian):

```
#p td(nstates=75,singlets) b3lyp 6-311++g(d,p) scrf=(iefpcm,solvent=acetonitrile)
int=grid=ultrafine
```

```
#p tda(nstates=75,singlets) b3lyp 6-311++g(d,p) scrf=(iefpcm,solvent=acetonitrile)
int=grid=ultrafine
```

```
#p td(nstates=75,singlets) cam-b3lyp def2tzvpp scrf=(iefpcm,solvent=acetonitrile)
int=grid=ultrafine
```

```
#p td(nstates=75,singlets) wb97xd def2tzvpp scrf=(iefpcm,solvent=acetonitrile)
int=grid=ultrafine
```

- TDA calculations (Orca):

```
! B3LYP def2-TZVPP def2-TZVPP/J RIJCOSX TightSCF Grid5 FinalGrid6 GridX4
! COSMO(acetonitrile)
%tddft maxdim 600 nroots 100 end
```

```
! B3LYP def2-TZVPP def2-TZVPP/J RIJCOSX TightSCF Grid5 FinalGrid6 GridX4
! COSMO
%cosmo smd true solvent "ACETONITRILE" end
%tddft maxdim 600 nroots 100 end
```

```
! B3LYP ma-def2-TZVPP AutoAux RIJCOSX TightSCF Grid5 FinalGrid6 GridX4
! COSMO(acetonitrile)
%tddft maxdim 600 nroots 100 end
```

```
! CAM-B3LYP def2-TZVPP def2-TZVPP/J RIJCOSX TightSCF Grid5 FinalGrid6 GridX4
! COSMO(acetonitrile)
%tddft maxdim 600 nroots 100 end
```

7.2 Boltzmann Weightings

Table 7.1: Boltzmann Analysis for (*S*)-**3** in chloroform.

Conformer	$\frac{H}{E_\mathrm{h}}$	$\frac{\Delta H}{\mathrm{kJ/mol}}$	$\frac{\Delta H}{\mathrm{kcal/mol}}$	Fraction	Boltzmann Factor
mmff-M5	−1383.842090	0.0000	0.0000	0.092	1.000
mmff-M6	−1383.841955	0.3544	0.0847	0.079	0.867
mmff-M7	−1383.841945	0.3807	0.0910	0.079	0.858
mmff-M1	−1383.841923	0.4385	0.1048	0.077	0.838
mmff-M3	−1383.841914	0.4621	0.1104	0.076	0.830
mmff-M8	−1383.841910	0.4726	0.1130	0.076	0.826
mmff-M4	−1383.841877	0.5592	0.1337	0.073	0.798
mmff-M2	−1383.841804	0.7509	0.1795	0.068	0.739
pm6-M5	−1383.841521	1.4939	0.3571	0.050	0.547
pm6-M3	−1383.841495	1.5622	0.3734	0.049	0.532
pm6-M1	−1383.841430	1.7328	0.4142	0.046	0.497
pm6-M2	−1383.841406	1.7958	0.4292	0.045	0.484
pm6-M4	−1383.841403	1.8037	0.4311	0.044	0.483
mmff-M13	−1383.840261	4.8020	1.1477	0.013	0.144
mmff-M9	−1383.840223	4.9018	1.1716	0.013	0.138
mmff-M11	−1383.840205	4.9491	1.1829	0.013	0.136
mmff-M14	−1383.840183	5.0068	1.1967	0.012	0.133
mmff-M15	−1383.840164	5.0567	1.2086	0.012	0.130
mmff-M10	−1383.840139	5.1224	1.2243	0.012	0.127
mmff-M12	−1383.840123	5.1644	1.2343	0.012	0.124
mmff-M16	−1383.840121	5.1696	1.2356	0.012	0.124
pm6-M21	−1383.839841	5.9047	1.4113	0.009	0.092
pm6-M19	−1383.839769	6.0938	1.4564	0.008	0.086
pm6-M25	−1383.839704	6.2644	1.4972	0.007	0.080
pm6-M28	−1383.839684	6.3170	1.5098	0.007	0.078
mmff-M17	−1383.838766	8.7272	2.0858	0.003	0.030
mmff-M18	−1383.838670	8.9792	2.1461	0.003	0.027
mmff-M19	−1383.838668	8.9845	2.1473	0.002	0.027
mmff-M20	−1383.838653	9.0238	2.1568	0.002	0.026
pm6-M14	−1383.838295	9.9638	2.3814	0.002	0.018
pm6-M13	−1383.838153	10.3366	2.4705	0.001	0.015
pm6-M17	−1383.838111	10.4469	2.4969	0.001	0.015
mmff-M21	−1383.836942	13.5161	3.2304	0.000	0.004
mmff-M22	−1383.836897	13.6342	3.2587	0.000	0.004
mmff-M23	−1383.836859	13.7340	3.2825	0.000	0.004
mmff-M24	−1383.836804	13.8784	3.3170	0.000	0.004
pm6-M44	−1383.836566	14.5033	3.4664	0.000	0.003
pm6-M41	−1383.836541	14.5689	3.4821	0.000	0.003
pm6-M47	−1383.836521	14.6214	3.4946	0.000	0.003
pm6-M265	−1383.834580	19.7175	4.7126	0.000	0.000
mmff-M76	−1383.834210	20.6889	4.9448	0.000	0.000
mmff-M74	−1383.834193	20.7336	4.9554	0.000	0.000
mmff-M95	−1383.834139	20.8754	4.9893	0.000	0.000

Table 7.2: Boltzmann Analysis for (*S*)-**3** in acetonitrile.

Conformer	$\frac{H}{E_h}$	$\frac{\Delta H}{\text{kJ/mol}}$	$\frac{\Delta H}{\text{kcal/mol}}$	Fraction	Boltzmann Factor
mmff-M5	−1383.846910	0.0000	0.0000	0.085	1.000
mmff-M7	−1383.846813	0.2547	0.0609	0.076	0.902
mmff-M1	−1383.846807	0.2704	0.0646	0.076	0.897
mmff-M8	−1383.846796	0.2993	0.0715	0.075	0.886
mmff-M6	−1383.846751	0.4175	0.0998	0.071	0.845
mmff-M4	−1383.846730	0.4726	0.1130	0.070	0.826
mmff-M3	−1383.846730	0.4726	0.1130	0.070	0.826
mmff-M2	−1383.846671	0.6275	0.1500	0.066	0.776
pm6-M5	−1383.846307	1.5832	0.3784	0.045	0.528
pm6-M3	−1383.846272	1.6751	0.4004	0.043	0.509
pm6-M1	−1383.846234	1.7748	0.4242	0.041	0.489
pm6-M4	−1383.846227	1.7932	0.4286	0.041	0.485
pm6-M2	−1383.846163	1.9612	0.4687	0.038	0.453
mmff-M13	−1383.845480	3.7545	0.8973	0.019	0.220
mmff-M9	−1383.845462	3.8017	0.9086	0.018	0.216
mmff-M11	−1383.845445	3.8464	0.9193	0.018	0.212
mmff-M14	−1383.845413	3.9304	0.9394	0.017	0.205
mmff-M16	−1383.845399	3.9671	0.9482	0.017	0.202
mmff-M15	−1383.845397	3.9724	0.9494	0.017	0.201
mmff-M10	−1383.845378	4.0223	0.9613	0.017	0.197
mmff-M12	−1383.845280	4.2796	1.0228	0.015	0.178
pm6-M21	−1383.845113	4.7180	1.1276	0.013	0.149
pm6-M25	−1383.845071	4.8283	1.1540	0.012	0.142
pm6-M28	−1383.845037	4.9176	1.1753	0.012	0.137
pm6-M19	−1383.845030	4.9359	1.1797	0.012	0.136
mmff-M17	−1383.843576	8.7534	2.0921	0.003	0.029
mmff-M20	−1383.843532	8.8689	2.1197	0.002	0.028
mmff-M18	−1383.843478	9.0107	2.1536	0.002	0.026
pm6-M14	−1383.843050	10.1344	2.4222	0.001	0.017
pm6-M17	−1383.842942	10.4180	2.4900	0.001	0.015
pm6-M13	−1383.842919	10.4784	2.5044	0.001	0.015
mmff-M24	−1383.842184	12.4081	2.9656	0.001	0.007
mmff-M21	−1383.842162	12.4659	2.9794	0.001	0.007
mmff-M22	−1383.842137	12.5315	2.9951	0.001	0.006
pm6-M47	−1383.841886	13.1905	3.1526	0.000	0.005
pm6-M44	−1383.841828	13.3428	3.1890	0.000	0.005
pm6-M41	−1383.841789	13.4452	3.2135	0.000	0.004
pm6-M265	−1383.840406	17.0763	4.0813	0.000	0.001
mmff-M96	−1383.840086	17.9164	4.2821	0.000	0.001
mmff-M97	−1383.840020	18.0897	4.3235	0.000	0.001
mmff-M76	−1383.839985	18.1816	4.3455	0.000	0.001
mmff-M95	−1383.839980	18.1947	4.3486	0.000	0.001
mmff-M74	−1383.839966	18.2315	4.3574	0.000	0.001
mmff-M98	−1383.839923	18.3444	4.3844	0.000	0.001
mmff-M77	−1383.839910	18.3785	4.3926	0.000	0.001
mmff-M75	−1383.839901	18.4021	4.3982	0.000	0.001

7.3 Geometries

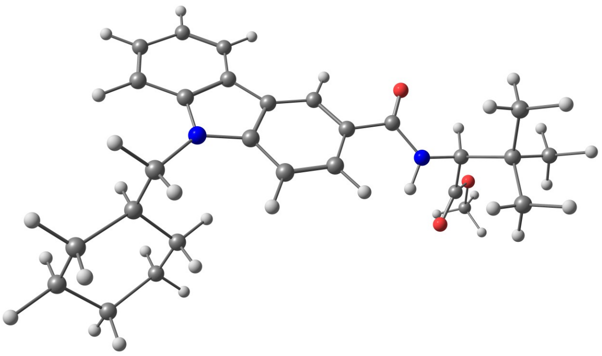

Figure 7.1: Lowest-energy conformer of (*S*)-**3** in chloroform.

In the following, the atomic coordinates for the optimized conformers of (*S*)-**3** in chloroform are given in xyz format:

```
66
mmff-M10
C -3.45636 4.03974 0.69932
C -3.85457 2.84817 0.10756
C -2.86437 1.92309 -0.22433
C -1.49639 2.18644 0.03362
C -1.12330 3.39101 0.63311
C -2.10669 4.31263 0.96286
N -2.98315 0.67732 -0.82391
C -1.72441 0.13497 -0.96270
C -0.76372 1.03552 -0.43646
C -1.33798 -1.07829 -1.53894
C 0.01211 -1.37828 -1.57220
C 0.98160 -0.51245 -1.03158
C 0.58779 0.70369 -0.47258
C 2.40818 -0.94647 -1.12573
N 3.27938 -0.33008 -0.27325
O 2.76923 -1.80176 -1.92659
C 4.67286 -0.71912 -0.19090
C 5.00924 -1.24818 1.19749
C 5.65308 0.41270 -0.65362
C 5.53455 1.64668 0.24785
C 7.09536 -0.10767 -0.63678
C 5.28325 0.79343 -2.09267
O 4.26598 -0.67191 2.15407
O 5.85486 -2.07754 1.42343
C 4.51281 -1.11389 3.49844
C -4.21363 0.04370 -1.26692
C -4.73594 -1.05423 -0.33333
C -5.94350 -1.75262 -0.96930
C -6.49403 -2.86233 -0.07196
C -6.83423 -2.33941 1.32409
C -5.63413 -1.63772 1.96065
C -5.09276 -0.52668 1.05956
H -4.21003 4.77369 0.96487
H -4.90341 2.65269 -0.08189
H -0.07907 3.60522 0.83652
H -1.83204 5.25313 1.42789
H -2.06390 -1.76826 -1.95306
H 0.35457 -2.30292 -2.02168
H 1.32021 1.41061 -0.09691
H 2.90152 0.17721 0.51100
H 4.79875 -1.56275 -0.87327
H 6.19407 2.43929 -0.11775
H 4.51512 2.04401 0.25110
H 5.81908 1.42831 1.28109
H 7.76502 0.65959 -1.03684
H 7.43410 -0.35806 0.37067
H 7.20028 -1.00255 -1.25770
H 4.26520 1.18500 -2.15471
H 5.96589 1.56662 -2.45776
H 5.35791 -0.06881 -2.76156
H 5.54579 -0.90778 3.78191
H 3.82464 -0.54914 4.12300
H 4.31911 -2.18378 3.58533
H -4.96840 0.82587 -1.38520
H -4.03888 -0.37162 -2.26534
H -3.93877 -1.80211 -0.21571
H -6.73187 -1.00809 -1.15059
H -5.66869 -2.16089 -1.94896
H -7.37723 -3.31422 -0.53580
H -5.74419 -3.66003 0.01360
H -7.66921 -1.62952 1.25005
H -7.17842 -3.16005 1.96252
H -5.90731 -1.22618 2.93804
H -4.83914 -2.37302 2.14340
H -5.85539 0.25859 0.95752
H -4.21826 -0.05768 1.52182
66
mmff-M11
C 3.37645 4.00033 0.78904
C 3.76776 2.84697 0.12121
C 2.80426 1.85985 -0.08821
C 1.47024 2.02349 0.35689
C 1.10394 3.19156 1.02809
C 2.06100 4.17375 1.24192
N 2.92673 0.63649 -0.72880
C 1.69989 0.00786 -0.71392
C 0.75858 0.83512 -0.04811
C 1.32491 -1.23521 -1.23056
C 0.00947 -1.63266 -1.07542
C -0.94488 -0.82344 -0.43004
C -0.56031 0.41167 0.09208
C -2.33079 -1.37026 -0.31793
N -3.31360 -0.46633 -0.03102
O -2.56487 -2.56435 -0.47018
C -4.71180 -0.84588 0.00629
C -5.50831 -0.08541 -1.04615
C -5.34245 -0.72695 1.43623
C -5.32602 0.72509 1.92633
C -6.78223 -1.25408 1.41555
C -4.51210 -1.59641 2.38875
O -4.99957 1.13298 -1.28451
O -6.49220 -0.51274 -1.59677
C -5.68479 1.92284 -2.26942
C 4.15401 0.12943 -1.31859
C 5.15768 -0.44106 -0.30978
C 6.46658 -0.80126 -1.02190
C 7.50183 -1.37356 -0.05198
C 6.94757 -2.57239 0.71841
C 5.64037 -2.21825 1.42779
C 4.60887 -1.65203 0.45043
H 4.10764 4.78249 0.96447
H 4.78896 2.72673 -0.22159
H 0.08580 3.33154 1.37641
H 1.79125 5.08591 1.76293
H 2.03514 -1.88125 -1.73252
H -0.31686 -2.59470 -1.45217
H -1.26300 1.03792 0.63215
H -3.11186 0.51373 -0.14817
H -4.75385 -1.89792 -0.28453
H -5.72403 0.77905 2.94396
H -4.30886 1.12732 1.94989
H -5.93683 1.37764 1.29602
H -7.19107 -1.24058 2.43038
H -7.43524 -0.65102 0.78126
H -6.82055 -2.28521 1.05122
H -3.47901 -1.24664 2.45202
H -4.94373 -1.55970 3.39349
H -4.49864 -2.64031 2.06255
H -6.71549 2.10333 -1.96115
H -5.13384 2.85854 -2.32887
H -5.67822 1.41246 -3.23337
H 3.88250 -0.63636 -2.05042
H 4.61893 0.94594 -1.88186
H 5.37943 0.34572 0.42520
H 6.25408 -1.54338 -1.80469
H 6.87289 0.08092 -1.53050
H 8.40844 -1.65637 -0.59745
H 7.79745 -0.59170 0.66029
H 6.76522 -3.39871 0.01807
H 7.68743 -2.93342 1.44076
H 5.23057 -3.09834 1.93437
H 5.84223 -1.47426 2.21000
H 4.33340 -2.43165 -0.27402
H 3.69247 -1.38064 0.98417
66
mmff-M12
C 3.34844 4.08249 0.47143
C 3.74726 2.88197 -0.10153
C 2.79306 1.87213 -0.22836
C 1.45957 2.06153 0.21128
C 1.08557 3.27731 0.78699
C 2.03342 4.28260 0.91439
N 2.91928 0.59850 -0.76453
C 1.69774 -0.03347 -0.68685
C 0.75419 0.83825 -0.08640
C 1.33321 -1.31673 -1.10314
C 0.02142 -1.71127 -0.91072
C -0.93608 -0.85938 -0.32813
C -0.56072 0.41704 0.09227
C -2.31880 -1.40343 -0.16992
N -3.30856 -0.48212 0.02254
O -2.54401 -2.60819 -0.20810
C -4.70500 -0.86491 0.08521
C -5.49967 -0.20655 -1.03517
C -5.34730 -0.62131 1.49408
C -5.35777 0.87087 1.84392
C -6.77794 -1.17262 1.51524
C -4.51036 -1.38218 2.52993
O -4.98853 0.98222 -1.38925
O -6.48551 -0.68186 -1.54129
C -5.67516 1.67708 -2.44247
C 4.12001 0.01603 -1.33965
C 4.85272 -0.97228 -0.42502
C 5.36709 -0.32066 0.86209
C 6.11795 -1.32380 1.73968
C 7.26248 -1.98951 0.97523
C 6.76287 -2.63644 -0.31698
C 6.00432 -1.63505 -1.18964
H 4.07455 4.88140 0.57981
H 4.77007 2.74386 -0.43161
H 0.06777 3.43488 1.12877
H 1.75778 5.23216 1.36000
H 2.04818 -1.99140 -1.55925
H -0.29942 -2.70249 -1.20836
H -1.26850 1.07788 0.58202
H -3.11087 0.48300 -0.18869
H -4.73977 -1.93919 -0.10960
H -5.76191 1.01470 2.85034
H -4.34766 1.29099 1.83264
H -5.97613 1.45031 1.15237
H -7.19627 -1.06516 2.52056
H -7.43485 -0.64796 0.81854
H -6.79517 -2.23557 1.25587
H -3.48499 -1.00729 2.56982
H -4.95346 -1.26188 3.52305
H -4.47327 -2.45100 2.30124
H -6.70190 1.89579 -2.14590
H -5.11750 2.59772 -2.59756
H -5.68036 1.07417 -3.35138
H 3.83797 -0.48404 -2.27262
H 4.78847 0.83535 -1.61807
H 4.13985 -1.75967 -0.14177
H 6.04427 0.50386 0.59700
H 4.53595 0.12178 1.42045
H 6.49834 -0.82361 2.63655
H 5.41679 -2.09450 2.08666
H 8.02071 -1.23323 0.73070
H 7.75817 -2.73561 1.60542
H 7.60052 -3.06270 -0.87917
H 6.09631 -3.47284 -0.06806
H 6.69905 -0.85590 -1.53472
H 5.62137 -2.13205 -2.08866
66
mmff-M13
C 4.89721 3.50582 0.78545
C 4.75012 2.30333 0.10570
C 3.45705 1.80612 -0.05919
C 2.32961 2.49990 0.44141
C 2.50510 3.70498 1.12370
C 3.79041 4.20077 1.29294
N 3.03700 0.65044 -0.70105
C 1.66162 0.58549 -0.63232
C 1.17410 1.71776 0.07236
C 0.78413 -0.37103 -1.14742
C -0.57393 -0.18940 -0.94085
C -1.07674 0.92069 -0.23412
C -0.19329 1.87954 0.25928
C -2.53378 1.15527 -0.00180
N -3.34948 0.07105 -0.15683
O -2.96644 2.26070 0.30597
C -4.76750 0.13416 0.13421
C -5.13703 -0.83076 1.25346
C -5.66341 -0.05656 -1.13769
C -5.47456 -1.45356 -1.73936
C -7.13703 0.15968 -0.77336
C -5.24940 1.00284 -2.16663
O -4.33645 -1.90700 1.28032
O -6.05151 -0.66500 2.02173
C -4.60995 -2.87297 2.30766
C 3.91936 -0.28954 -1.37048
C 4.65759 -1.25295 -0.43398
C 5.68301 -2.06887 -1.22940
C 6.44975 -3.04462 -0.33527
C 5.50114 -3.96008 0.43927
C 4.47488 -3.15057 1.23241
C 3.70882 -2.18087 0.33048
H 5.89270 3.91361 0.92684
H 5.61572 1.77677 -0.27935
H 1.64957 4.24718 1.51269
H 3.94298 5.13579 1.82084
H 1.13978 -1.23133 -1.70155
H -1.25394 -0.91667 -1.37157
H -0.59752 2.73976 0.78055
H -2.92961 -0.84440 -0.17944
H -4.95871 1.13699 0.52297
H -6.06582 -1.54798 -2.65501
H -4.42883 -1.63733 -2.00360
H -5.79713 -2.24245 -1.05375
H -7.75052 0.09420 -1.67701
H -7.50145 -0.58504 -0.06300
H -7.29337 1.14867 -0.33178
H -4.21090 0.87305 -2.47984
H -5.88332 0.92265 -3.05480
H -5.35867 2.01258 -1.76087
H -5.61389 -3.28206 2.18624
H -3.86199 -3.65245 2.18273
H -4.52223 -2.41187 3.29220
H 3.32307 -0.85296 -2.09352
H 4.64594 0.28847 -1.95212
H 5.20587 -0.65017 0.30376
H 5.15921 -2.63227 -2.01492
H 6.38193 -1.39680 -1.74104
```

H 7.14452 -3.63800 -0.93909
H 7.06262 -2.47471 0.37572
H 4.97476 -4.61553 -0.26779
H 6.06890 -4.61529 1.10850
H 3.77360 -3.81920 1.74278
H 4.99016 -2.58165 2.01779
H 3.11489 -2.75625 -0.39422
H 3.00052 -1.59266 0.92279
66
mmff-M14
C 4.77442 3.68075 -0.23392
C 4.63947 2.41000 0.31067
C 3.38143 1.80970 0.25113
C 2.27604 2.46838 -0.33854
C 2.43920 3.74406 -0.88124
C 3.69004 4.34310 -0.82639
N 2.97728 0.57042 0.72516
C 1.63343 0.41594 0.45986
C 1.15152 1.57324 -0.20674
C 0.78229 -0.65318 0.74757
C -0.54779 -0.54683 0.37434
C -1.04659 0.59452 -0.28379
C -0.18412 1.64805 -0.58290
C -2.46886 0.73524 -0.71898
N -3.36582 -0.10301 -0.12032
O -2.80402 1.55367 -1.56844
C -4.78863 -0.01209 -0.37783
C -5.54986 0.35290 0.89027
C -5.36566 -1.29154 -1.07495
C -5.20494 -2.52616 -0.18074
C -6.84718 -1.08008 -1.40904
C -4.59034 -1.50282 -2.38144
O -4.95071 -0.11769 1.99453
O -6.58056 0.97834 0.90988
C -5.59784 0.18278 3.24125
C 3.84371 -0.38249 1.39703
C 4.75348 -1.18683 0.46087
C 3.96692 -2.05573 -0.52517
C 4.89759 -2.86954 -1.42590
C 5.86520 -3.72229 -0.60510
C 6.65209 -2.86637 0.38797
C 5.72162 -2.04556 1.28268
H 5.74271 4.16916 -0.20014
H 5.48793 1.91010 0.76355
H 1.60024 4.26018 -1.33630
H 3.83274 5.33380 -1.24398
H 1.14126 -1.55245 1.23346
H -1.19845 -1.39254 0.56963
H -0.57911 2.51056 -1.10725
H -3.08606 -0.58045 0.72143
H -4.92523 0.82697 -1.06361
H -5.57159 -3.41448 -0.70356
H -4.15522 -2.70447 0.07153
H -5.76902 -2.43327 0.75167
H -7.22311 -1.94373 -1.96579
H -7.46121 -0.96353 -0.51363
H -6.98943 -0.19111 -2.03102
H -3.52811 -1.67559 -2.19334
H -4.98670 -2.37489 -2.91011
H -4.68249 -0.63511 -3.04095
H -6.59862 -0.25073 3.26361
H -4.97124 -0.26210 4.01073
H -5.66670 1.26245 3.38000
H 4.45295 0.16888 2.12154
H 3.21039 -1.05965 1.97696
H 5.35025 -0.47204 -0.12327
H 3.32268 -2.74304 0.04170
H 3.30387 -1.43214 -1.13324
H 4.30783 -3.50152 -2.09819
H 5.47029 -2.18445 -2.06516
H 5.29640 -4.48347 -0.05397
H 6.55112 -4.26313 -1.26579
H 7.30240 -3.49671 1.00378
H 7.31156 -2.18491 -0.16575
H 5.14109 -2.72590 1.92213
H 6.30879 -1.40987 1.95583
66
mmff-M15
C 4.92268 3.52972 0.65777
C 4.77008 2.31275 0.00616
C 3.47597 1.81441 -0.14617
C 2.35290 2.52300 0.34654
C 2.53397 3.74279 1.00087
C 3.82029 4.24028 1.15307
N 3.04487 0.64368 -0.75540
C 1.67140 0.58534 -0.67038
C 1.19193 1.73435 0.01001
C 0.79187 -0.38231 -1.16066
C -0.56468 -0.19683 -0.94590
C -1.06042 0.92745 -0.25671
C -0.17382 1.89926 0.20591
C -2.51510 1.16342 -0.00943
N -3.33084 0.07559 -0.13547
O -2.94561 2.27291 0.28628
C -4.74433 0.14062 0.17714
C -5.09530 -0.81772 1.30786
C -5.66033 -0.05860 -1.07914
C -5.48029 -1.45924 -1.67507
C -7.12802 0.15944 -0.69255
C -5.26327 0.99436 -2.12133
O -4.29443 -1.89404 1.32763
O -5.99692 -0.64756 2.09014
C -4.55191 -2.85483 2.36399
C 3.87962 -0.35059 -1.40754
C 4.12226 -1.62014 -0.58329
C 4.85983 -1.34270 0.72998
C 5.11318 -2.63012 1.51625
C 5.87120 -3.65875 0.67665
C 5.14872 -3.93835 -0.64164
C 4.88719 -2.64966 -1.42297
H 5.92030 3.93626 0.78724
H 5.63545 1.77518 -0.36324
H 1.68073 4.29359 1.38278
H 3.97741 5.18673 1.65876
H 1.14659 -1.25085 -1.70302
H -1.24891 -0.93217 -1.35568
H -0.57524 2.77068 0.71047
H -2.91009 -0.83963 -0.14898
H -4.93025 1.14584 0.56238
H -6.08610 -1.55949 -2.58053
H -4.43878 -1.64410 -1.95487
H -5.79148 -2.24405 -0.97960
H -7.75580 0.08865 -1.58592
H -7.48110 -0.58116 0.02781
H -7.27758 1.15095 -0.25431
H -4.23011 0.86212 -2.45082
H -5.91172 0.90857 -2.99844
H -5.36546 2.00669 -1.72031
H -5.55709 -3.26549 2.25922
H -3.80511 -3.63430 2.23221
H -4.45039 -2.38851 3.34473
H 3.40894 -0.61373 -2.36128
H 4.83327 0.12383 -1.65459
H 3.14191 -2.05076 -0.33381
H 5.82290 -0.86400 0.50245
H 4.29227 -0.63267 1.33990
H 5.66658 -2.40433 2.43384
H 4.15083 -3.05632 1.83000
H 6.87855 -3.27696 0.46211
H 6.00362 -4.58812 1.24080
H 5.72934 -4.63582 -1.25468
H 4.19041 -4.43156 -0.43084
H 5.84713 -2.21343 -1.73420
H 4.33268 -2.87130 -2.34247
66
mmff-M16
C 4.75170 3.71236 -0.03354
C 4.62099 2.41575 0.44664
C 3.36964 1.80749 0.34466
C 2.26686 2.48680 -0.22864
C 2.42579 3.78865 -0.70699
C 3.66968 4.39527 -0.60660
N 2.96598 0.54185 0.74784
C 1.62840 0.39404 0.45396
C 1.14692 1.57895 -0.16036
C 0.78373 -0.69390 0.68450
C -0.54408 -0.57817 0.30454
C -1.04301 0.59038 -0.30355
C -0.18561 1.66292 -0.54570
C -2.46386 0.74263 -0.73989
N -3.36233 -0.10732 -0.16008
O -2.79616 1.58028 -1.57130
C -4.78513 -0.00761 -0.41473
C -5.54432 0.33679 0.86046
C -5.36597 -1.27279 -1.13400
C -5.20509 -2.52374 -0.26284
C -6.84786 -1.05302 -1.46079
C -4.59376 -1.46111 -2.44581
O -4.94499 -0.15439 1.95560
O -6.57356 0.96412 0.89208
C -5.58980 0.12579 3.20824
C 3.78863 -0.46348 1.39865
C 4.26801 -1.59053 0.47633
C 4.97008 -2.67747 1.29859
C 5.46102 -3.82730 0.41730
C 6.36193 -3.32492 -0.71125
C 5.66752 -2.23777 -1.53208
C 5.18344 -1.08963 -0.64475
H 5.71601 4.20491 0.03573
H 5.47087 1.90213 0.88035
H 1.58797 4.31809 -1.14867
H 3.80911 5.40630 -0.97343
H 1.14573 -1.61056 1.13481
H -1.19292 -1.43471 0.45326
H -0.58277 2.54738 -1.03038
H -3.08355 -0.60049 0.67304
H -4.91992 0.84414 -1.08507
H -5.57472 -3.40182 -0.80062
H -4.15496 -2.70820 -0.01691
H -5.76632 -2.44679 0.67272
H -7.22607 -1.90537 -2.03319
H -7.45985 -0.95299 -0.56202
H -6.99037 -0.15207 -2.06521
H -3.53139 -1.63885 -2.26324
H -4.99253 -2.32266 -2.98971
H -4.68601 -0.58122 -3.08900
H -6.59075 -0.30758 3.22520
H -4.96211 -0.33206 3.96918
H -5.65787 1.20304 3.36488
H 4.64671 0.04517 1.84663
H 3.21086 -0.88534 2.22842
H 3.38107 -2.04213 0.00948
H 5.82715 -2.23114 1.82288
H 4.29417 -3.05792 2.07351
H 5.99045 -4.56643 1.02793
H 4.59380 -4.34377 -0.01538
H 7.28613 -2.91639 -0.28051
H 6.66040 -4.15763 -1.35702
H 6.34199 -1.85368 -2.30456
H 4.80780 -2.67383 -2.05819
H 6.05362 -0.58978 -0.19579
H 4.66538 -0.33781 -1.24861
66
mmff-M17
C -3.85505 3.73148 1.10996
C -4.17253 2.62416 0.33406
C -3.13086 1.78656 -0.06616
C -1.79012 2.06441 0.29395
C -1.49776 3.17879 1.08256
C -2.53397 4.00724 1.48852
N -3.16814 0.63698 -0.84653
C -1.87316 0.19361 -1.02850
C -0.98214 1.04012 -0.32084
C -1.39092 -0.87240 -1.79306
C -0.02485 -1.08684 -1.82286
C 0.87368 -0.28036 -1.09980
C 0.38760 0.79428 -0.35536
C 2.32557 -0.61408 -1.20999
N 3.14034 -0.07573 -0.25528
O 2.75677 -1.32241 -2.11356
C 4.54597 -0.41679 -0.16934
C 4.78838 -0.97815 1.22437
C 5.48555 0.78929 -0.51122
C 5.24925 1.95841 0.45102
C 6.95220 0.34710 -0.44320
C 5.16868 1.23590 -1.94383
O 5.72626 -1.92497 1.23108
O 4.19407 -0.61135 2.21224
C 6.03758 -2.49597 2.51349
C -4.35428 0.08683 -1.49013
C -5.11868 -0.99636 -0.71069
C -5.70003 -0.49233 0.61503
C -6.50648 -1.57591 1.33382
C -5.67828 -2.84165 1.55022
C -5.10809 -3.35426 0.22849
C -4.29944 -2.27316 -0.49132
H -4.65111 4.39532 1.43069
H -5.20045 2.42639 0.05636
H -0.47414 3.39794 1.36845
H -2.32267 4.87796 2.09963
H -2.05603 -1.51887 -2.35166
H 0.38672 -1.89658 -2.41361
H 1.06096 1.46494 0.16801
H 2.72448 0.26938 0.59741
H 4.73192 -1.20613 -0.90065
H 5.88708 2.80280 0.17323
H 4.21141 2.30121 0.41032
H 5.47913 1.69109 1.48603
H 7.59683 1.16368 -0.78167
H 7.25832 0.08146 0.57152
H 7.13802 -0.51713 -1.08816
H 4.13378 1.57383 -2.03583
H 5.82163 2.06755 -2.22548
H 5.32507 0.42200 -2.65736
H 5.14945 -2.95831 2.94605
H 6.80295 -3.24371 2.32026
H 6.41412 -1.72794 3.19060
H -5.02619 0.92334 -1.70420
H -4.04427 -0.31514 -2.45947
H -5.96579 -1.25750 -1.36371
H -4.88163 -0.15965 1.26613
H -6.33521 0.38223 0.43506
H -6.87478 -1.19138 2.29084
H -7.39338 -1.82321 0.73473
H -4.85226 -2.62044 2.23932
H -6.28599 -3.61700 2.02891
H -4.48101 -4.23584 0.39844
H -5.93391 -3.67876 -0.41909
H -3.40789 -2.03454 0.10252
H -3.94424 -2.65736 -1.45416
66
mmff-M18
C 3.68194 3.92716 0.43694
C 4.00763 2.74515 -0.21551
C 3.01723 1.76938 -0.33306
C 1.71568 1.98391 0.18118
C 1.41492 3.17781 0.83947
C 2.40243 4.14401 0.96647
N 3.07088 0.52067 -0.94130
C 1.81739 -0.05359 -0.86478
C 0.94370 0.81546 -0.16307
C 1.35850 -1.27320 -1.37016
C 0.03412 -1.60993 -1.15626
C -0.84947 -0.76244 -0.46204
C -0.38438 0.45207 0.04154
C -2.25376 -1.24142 -0.28874
N -3.17467 -0.29444 0.05924
O -2.55499 -2.42021 -0.44252
C -4.59034 -0.59600 0.12917
C -5.29981 0.34862 -0.83054
C -5.15757 -0.51879 1.58759
C -4.97538 0.88569 2.17325
C -6.64391 -0.89575 1.59262
C -4.38740 -1.53241 2.44299
O -6.36897 -0.21336 -1.39380
O -4.93288 1.47837 -1.05992
C -7.11706 0.61826 -2.29730
C 4.21200 -0.02223 -1.66750
C 5.19044 -0.89296 -0.86122
C 4.55041 -2.17452 -0.31562
C 5.56393 -3.04889 0.42579
C 6.26378 -2.27426 1.54143
C 6.91450 -1.00290 0.99839
C 5.90266 -0.12560 0.25829
H 4.43864 4.69808 0.53859
H 5.00328 2.59617 -0.61435
H 0.42117 3.35082 1.23956
H 2.18412 5.07714 1.47425
H 2.01139 -1.94725 -1.91027
H -0.35447 -2.55196 -1.52463
H -1.03290 1.11035 0.60994
H -2.94536 0.67822 -0.08399
H -4.72003 -1.61934 -0.22910
H -5.34192 0.90854 3.20384
H -3.92057 1.17422 2.19169
H -5.52496 1.64148 1.60553
H -7.00741 -0.93779 2.62365
H -7.25747 -0.16997 1.05357
H -6.80714 -1.87783 1.13844
H -3.32043 -1.29936 2.47268
H -4.76584 -1.51366 3.46952
H -4.50319 -2.54861 2.05561
H -6.48384 0.94174 -3.12435
H -7.93086 -0.00531 -2.65941
H -7.50742 1.49258 -1.77449
H 3.81840 -0.60223 -2.50768
H 4.75280 0.82299 -2.10390
H 5.95636 -1.19325 -1.59297
H 3.73449 -1.90918 0.36869
H 4.10132 -2.74557 -1.13597
H 5.06359 -3.93491 0.83061
H 6.31597 -3.41344 -0.28708
H 5.52845 -2.00442 2.31121
H 7.01250 -2.90575 2.03160
H 7.37645 -0.43225 1.81101
H 7.72522 -1.27821 0.31019
H 5.15779 0.24728 0.97274
H 6.41008 0.75283 -0.15625
66
mmff-M19
C -5.15707 -3.20809 0.80830
C -4.96717 -2.06915 0.03638
C -3.66249 -1.60313 -0.12948
C -2.56561 -2.27785 0.45804
C -2.78289 -3.41619 1.23628
C -4.08069 -3.87520 1.40922
N -3.19665 -0.51074 -0.85265
C -1.81818 -0.49417 -0.77338
C -1.38041 -1.56703 0.04560
C -0.89697 0.36081 -1.38270
C 0.45084 0.15297 -1.13751
C 0.90190 -0.88761 -0.30248
C -0.02278 -1.75675 0.27341
C 2.34558 -1.13963 -0.01414
N 3.19610 -0.09910 -0.25991
O 2.73897 -2.22003 0.41203
C 4.59752 -0.15414 0.10455
C 4.85968 0.99281 1.07026
C 5.54905 -0.12963 -1.13935
C 5.33230 1.13944 -1.97071
C 7.01089 -0.20840 -0.68323
C 5.22927 -1.36180 -1.99462
O 5.77089 0.67823 1.99074
O 4.30182 2.06474 1.01286
C 6.10063 1.71338 2.93253
C -4.01388 0.37079 -1.67625
C -4.53324 1.65352 -1.00530
C -3.41350 2.62075 -0.60441
C -3.96438 3.91405 -0.00055
C -4.88553 3.63100 1.18510
C -6.01090 2.67707 0.78680
C -5.46405 1.38227 0.18188
H -6.16418 -3.58666 0.94878
H -5.81331 -1.56669 -0.41572
H -1.94773 -3.93728 1.69270
H -4.26641 -4.75924 2.00941
H -1.21193 1.16399 -2.03686
H 1.16455 0.79995 -1.63611
H 0.34175 -2.57131 0.88872
H 2.80702 0.82810 -0.35051
H 4.75611 -1.09647 0.63275
H 5.98588 1.12447 -2.84799
H 4.30110 1.20733 -2.32868
H 5.55578 2.04575 -1.40139
H 7.66271 -0.29811 -1.55729
H 7.32038 0.68125 -0.12908
H 7.18297 -1.08005 -0.04461
H 4.19354 -1.34718 -2.34217
H 5.88047 -1.38251 -2.87373
H 5.38651 -2.28670 -1.43231
H 5.21190 2.01501 3.48822
H 6.83863 1.27460 3.59968
H 6.51764 2.57906 2.41589
H -3.42297 0.63696 -2.55797
H -4.86140 -0.21756 -2.04023
H -5.13085 2.14864 -1.78630
H -2.75703 2.13251 0.12706
H -2.79430 2.85576 -1.47765
H -3.13710 4.56379 0.30406
H -4.52473 4.46200 -0.77023
H -4.30061 3.18155 1.99869
H -5.29999 4.56595 1.57714

```
H -6.64034 2.44537 1.65254
H -6.66047 3.17245 0.05241
H -4.91493 0.82693 0.95286
H -6.29588 0.74318 -0.13479
66
mmff-M1
C -3.63554 3.86651 1.15512
C -3.99696 2.73312 0.43790
C -2.97754 1.87642 0.02151
C -1.61908 2.14867 0.31247
C -1.28333 3.29265 1.03881
C -2.29523 4.14570 1.45696
N -3.06299 0.70122 -0.70921
C -1.78932 0.21025 -0.90260
C -0.85471 1.07679 -0.27861
C -1.36356 -0.92557 -1.59660
C -0.00543 -1.18053 -1.65338
C 0.93826 -0.34620 -1.02463
C 0.50624 0.79184 -0.34341
C 2.37640 -0.72694 -1.15812
N 3.23871 -0.13942 -0.27640
O 2.75692 -1.51383 -2.01831
C 4.63740 -0.51217 -0.21182
C 4.90953 -1.00047 1.20388
C 5.59527 0.64782 -0.64880
C 5.40845 1.87845 0.24528
C 7.05312 0.17646 -0.58648
C 5.25461 1.01353 -2.09883
O 5.82121 -1.97221 1.23587
O 4.35794 -0.56129 2.18694
C 6.15908 -2.47840 2.53853
C -4.30012 0.12301 -1.20507
C -5.11845 -0.63084 -0.15048
C -4.37465 -1.84215 0.41979
C -5.22428 -2.59311 1.44645
C -6.57166 -3.01281 0.85821
C -7.32071 -1.81259 0.27822
C -6.46718 -1.05699 -0.74171
H -4.41013 4.54851 1.48994
H -5.03782 2.52929 0.21438
H -0.24676 3.51414 1.27136
H -2.04955 5.03832 2.02188
H -2.06553 -1.59438 -2.08012
H 0.36443 -2.04515 -2.19166
H 1.21427 1.47734 0.11033
H 2.86183 0.27687 0.56248
H 4.78090 -1.34640 -0.90152
H 6.06110 2.68768 -0.09555
H 4.37895 2.24544 0.20451
H 5.65361 1.66734 1.28972
H 7.70685 0.95407 -0.99252
H 7.37857 -0.02970 0.43609
H 7.20393 -0.73191 -1.17762
H 4.22153 1.35702 -2.19080
H 5.91031 1.81981 -2.44135
H 5.38977 0.15789 -2.76661
H 5.27388 -2.89116 3.02401
H 6.89835 -3.25659 2.36485
H 6.57691 -1.68416 3.15879
H -4.90224 0.93241 -1.63234
H -4.04908 -0.54646 -2.03240
H -5.31837 0.06243 0.67883
H -4.12023 -2.52314 -0.40483
H -3.42838 -1.52693 0.87101
H -4.67878 -3.46892 1.81330
H -5.39439 -1.94508 2.31664
H -6.40346 -3.75190 0.06320
H -7.18240 -3.50836 1.62043
H -8.25860 -2.13587 -0.18578
H -7.59498 -1.12967 1.09331
H -6.28726 -1.70277 -1.61307
H -7.00968 -0.17879 -1.11113
66
mmff-M20
C 4.90755 3.51754 -0.33047
C 4.75225 2.31690 0.35034
C 3.50838 1.68765 0.28858
C 2.43357 2.26355 -0.43037
C 2.61690 3.46692 -1.11363
C 3.85658 4.08772 -1.06192
N 3.08347 0.50296 0.87983
C 1.74460 0.32975 0.59000
C 1.30011 1.39276 -0.23790
C 0.86208 -0.67164 1.00143
C -0.45523 -0.59781 0.57832
C -0.91244 0.44544 -0.24994
C -0.02185 1.43422 -0.66413
C -2.32096 0.55168 -0.73603
N -3.24631 -0.19689 -0.06545
O -2.62160 1.26546 -1.68671
C -4.66512 -0.08526 -0.33787
C -5.34236 0.34038 0.95716
C -5.27286 -1.39905 -0.93640
C -5.06522 -2.58260 0.01497
C -6.76987 -1.20811 -1.20813
C -4.55946 -1.68005 -2.26449
O -6.38527 1.14152 0.74072
O -4.97354 -0.00383 2.05678
C -7.10182 1.57512 1.90935
C 3.87475 -0.33173 1.77463
C 4.66014 -1.48403 1.12544
C 5.74458 -1.00397 0.15407
C 6.55004 -2.16881 -0.42474
C 5.64246 -3.20028 -1.09312
C 4.56776 -3.69150 -0.12417
C 3.75988 -2.52909 0.45690
H 5.86731 4.02241 -0.29462
H 5.57857 1.89186 0.90646
H 1.79921 3.91241 -1.67065
H 4.01515 5.02384 -1.58610
H 1.18601 -1.49506 1.62510
H -1.12915 -1.39357 0.87672
H -0.38602 2.22561 -1.30911
H -3.00766 -0.55001 0.84966
H -4.79055 0.71131 -1.07400
H -5.47447 -3.49364 -0.43173
H -4.00231 -2.75720 0.20480
H -5.56249 -2.42774 0.97631
H -7.16361 -2.08898 -1.72379
H -7.34458 -1.07877 -0.28758
H -6.95186 -0.33668 -1.84428
H -3.48533 -1.81645 -2.11825
H -4.95818 -2.59473 -2.71365
H -4.70524 -0.86075 -2.97435
H -6.43759 2.12648 2.57593
H -7.89571 2.22008 1.54052
H -7.51957 0.71801 2.43953
H 4.56900 0.32611 2.30616
H 3.19484 -0.73650 2.53037
H 5.16850 -1.97799 1.96795
H 5.27513 -0.44699 -0.66666
H 6.41850 -0.30640 0.66399
H 7.28988 -1.79064 -1.13816
H 7.11467 -2.65432 0.38281
H 5.16026 -2.74513 -1.96853
H 6.23374 -4.04395 -1.46491
H 3.89590 -4.39736 -0.62387
H 5.04672 -4.24355 0.69587
H 3.18566 -2.05132 -0.34708
H 3.03180 -2.91225 1.18082
66
mmff-M21
C -3.71057 3.79367 1.01484
C -4.05218 2.67934 0.25960
C -3.02714 1.81991 -0.13743
C -1.67892 2.08258 0.20606
C -1.36223 3.20487 0.97391
C -2.38179 4.05538 1.37629
N -3.08926 0.65985 -0.90012
C -1.80288 0.19406 -1.08757
C -0.89264 1.03642 -0.39973
C -1.34407 -0.88900 -1.84260
C 0.01813 -1.12587 -1.88110
C 0.93476 -0.32631 -1.17330
C 0.47264 0.76668 -0.44058
C 2.38035 -0.68610 -1.28519
N 3.19506 -0.19865 -0.30313
O 2.80344 -1.37484 -2.20709
C 4.59826 -0.55274 -0.22828
C 4.89934 -1.31195 1.05781
C 5.55117 0.67380 -0.43601
C 5.35438 1.71741 0.66916
C 7.01048 0.20333 -0.45415
C 5.21790 1.30025 -1.79577
O 4.09549 -0.94066 2.06561
O 5.76737 -2.14103 1.17230
C 4.30615 -1.60677 3.32071
C -4.29040 0.11628 -1.52114
C -5.05893 -0.94490 -0.71579
C -5.61824 -0.41276 0.60854
C -6.42907 -1.47469 1.35420
C -5.61389 -2.74704 1.58096
C -5.06598 -3.28761 0.26093
C -4.25284 -2.22821 -0.48555
H -4.49332 4.47470 1.33221
H -5.08552 2.49302 -0.00564
H -0.33256 3.41364 1.24570
H -2.15148 4.93281 1.97069
H -2.02416 -1.53153 -2.38757
H 0.41130 -1.94914 -2.46571
H 1.16257 1.43356 0.06615
H 2.76822 0.14886 0.54088
H 4.78317 -1.25538 -1.04397
H 5.99326 2.58505 0.47968
H 4.32026 2.07297 0.70450
H 5.61372 1.32334 1.65590
H 7.66489 1.05134 -0.67735
H 7.32169 -0.21930 0.50342
H 7.17115 -0.55734 -1.22416
H 4.18948 1.66767 -1.82635
H 5.88525 2.14592 -1.98709
H 5.34514 0.57697 -2.60630
H 5.31492 -1.41329 3.68809
H 3.56561 -1.19141 4.00018
H 4.16133 -2.68178 3.20645
H -4.95370 0.95850 -1.73950
H -3.99790 -0.30394 -2.48814
H -5.91684 -1.20573 -1.35462
H -4.78805 -0.07988 1.24449
H -6.24486 0.46665 0.42228
H -6.78103 -1.07044 2.30925
H -7.32615 -1.72043 0.76987
H -4.77696 -2.52523 2.25664
H -6.22521 -3.50707 2.07920
H -4.44779 -4.17416 0.43751
H -5.90341 -3.61205 -0.37151
H -3.35153 -1.99125 0.09410
H -3.91343 -2.63213 -1.44599
66
mmff-M22
C 3.52885 3.90456 0.70407
C 3.88692 2.77160 -0.01492
C 2.91989 1.78316 -0.20136
C 1.60928 1.93727 0.31171
C 1.27609 3.08224 1.03794
C 2.24018 4.06065 1.23286
N 3.00724 0.57534 -0.88338
C 1.76653 -0.03015 -0.85459
C 0.86715 0.77508 -0.11047
C 1.33997 -1.22669 -1.43747
C 0.02198 -1.60564 -1.25725
C -0.88704 -0.82177 -0.52230
C -0.45401 0.36995 0.05850
C -2.28202 -1.34149 -0.39292
N -3.23014 -0.43103 -0.02242
O -2.55234 -2.51856 -0.60523
C -4.63735 -0.77312 0.03342
C -5.44175 0.08273 -0.93664
C -5.22151 -0.73577 1.48747
C -5.15170 0.67950 2.07154
C -6.67459 -1.22538 1.47778
C -4.38643 -1.68727 2.35326
O -4.89480 1.29450 -1.11712
O -6.46191 -0.27113 -1.47322
C -5.58535 2.17265 -2.02009
C 4.16768 0.10217 -1.62762
C 5.15496 -0.79729 -0.86478
C 4.53803 -2.12646 -0.41484
C 5.56141 -3.02390 0.28406
C 6.22525 -2.30828 1.45956
C 6.85369 -0.98953 1.01158
C 5.83255 -0.08886 0.31362
H 4.26705 4.68433 0.85982
H 4.88926 2.66890 -0.41161
H 0.27567 3.20850 1.43893
H 1.99656 4.95607 1.79421
H 2.01260 -1.85172 -2.01134
H -0.34112 -2.53230 -1.68559
H -1.12174 0.97729 0.66073
H -3.00424 0.54860 -0.08850
H -4.72055 -1.80080 -0.32765
H -5.51617 0.67519 3.10305
H -4.12414 1.05489 2.08781
H -5.76491 1.38697 1.50601
H -7.05222 -1.26949 2.50377
H -7.33028 -0.56617 0.90528
H -6.75042 -2.22894 1.04804
H -3.34288 -1.36838 2.40673
H -4.78653 -1.70738 3.37144
H -4.41123 -2.70677 1.95818
H -6.59630 2.36819 -1.66010
H -5.00185 3.09013 -2.03926
H -5.63355 1.72860 -3.01520
H 3.79575 -0.43177 -2.50718
H 4.69545 0.98391 -2.00344
H 5.93806 -1.03119 -1.60250
H 3.70597 -1.92604 0.27212
H 4.11527 -2.65246 -1.27823
H 5.07698 -3.94621 0.62152
H 6.33282 -3.32271 -0.43862
H 5.47192 -2.10642 2.23274
H 6.98125 -2.95322 1.92012
H 7.28961 -0.46303 1.86724
H 7.68099 -1.20067 0.32046
H 5.06861 0.21974 1.03853
H 6.32511 0.82588 -0.03496
66
mmff-M23
C 5.13956 -3.19890 -0.78250
C 4.92189 -2.07487 0.00375
C 3.60965 -1.62284 0.14694
C 2.53257 -2.29701 -0.47666
C 2.77777 -3.42009 -1.26870
C 4.08324 -3.86484 -1.41932
N 3.11765 -0.54680 0.87735
C 1.74129 -0.54133 0.76747
C 1.33186 -1.60387 -0.07925
C 0.79875 0.29399 1.37126
C -0.54136 0.07754 1.09291
C -0.96417 -0.95174 0.22958
C -0.01855 -1.80219 -0.34032
C -2.39890 -1.21317 -0.09430
N -3.26290 -0.18046 0.13622
O -2.77250 -2.29309 -0.53885
C -4.66099 -0.25658 -0.23768
C -5.00420 0.80283 -1.27716
C -5.62777 -0.22089 0.99524
C -5.50036 1.10300 1.75693
C -7.07519 -0.41630 0.52836
C -5.24737 -1.38240 1.92193
O -4.25223 1.90609 -1.14503
O -5.86127 0.68088 -2.11646
C -4.50502 2.95947 -2.08845
C 3.90775 0.33021 1.73157
C 4.43113 1.62617 1.08976
C 3.31228 2.58560 0.66813
C 3.86531 3.89168 0.09433
C 4.82278 3.63244 -1.06778
C 5.94695 2.68620 -0.64836
C 5.39745 1.37870 -0.07404
H 6.15291 -3.56663 -0.90579
H 5.75274 -1.57303 0.48397
H 1.95797 -3.94063 -1.75272
H 4.29058 -4.73701 -2.02968
H 1.09162 1.08714 2.04763
H -1.27196 0.70725 1.58931
H -0.36148 -2.60956 -0.97714
H -2.87878 0.74103 0.27259
H -4.79612 -1.22045 -0.73357
H -6.14798 1.08901 2.63859
H -4.47614 1.26765 2.10474
H -5.79420 1.95912 1.14295
H -7.73607 -0.46782 1.39884
H -7.41951 0.40143 -0.10814
H -7.18666 -1.34821 -0.03420
H -4.22709 -1.27688 2.29806
H -5.92411 -1.40694 2.78132
H -5.32123 -2.34262 1.40338
H -5.53385 3.31061 -1.99821
H -3.80633 3.75326 -1.83477
H -4.33005 2.60539 -3.10517
H 3.29391 0.57936 2.60251
H 4.75181 -0.25549 2.10772
H 5.00128 2.11884 1.89252
H 2.68251 2.09818 -0.08705
H 2.66596 2.80384 1.52595
H 3.03972 4.53590 -0.22642
H 4.39745 4.43697 0.88559
H 4.26633 3.18607 -1.90278
H 5.23770 4.57633 -1.43723
H 6.60317 2.47143 -1.49840
H 6.56987 3.17996 0.10978
H 4.87595 0.82694 -0.86646
H 6.22688 0.74450 0.25830
66
mmff-M24
C 4.91568 3.46399 -0.28613
C 4.73089 2.25775 0.37714
C 3.47650 1.65236 0.29333
C 2.42071 2.25721 -0.42986
C 2.63357 3.46568 -1.09535
C 3.88367 4.06282 -1.02171
N 3.02337 0.46796 0.86406
C 1.68499 0.32313 0.55695
C 1.26951 1.40498 -0.26170
C 0.77955 -0.66738 0.94413
C -0.53141 -0.56320 0.50718
C -0.96000 0.49945 -0.31169
C -0.04620 1.47638 -0.70294
C -2.36030 0.63812 -0.81383
N -3.31182 -0.07909 -0.14580
O -2.63217 1.35398 -1.77144
C -4.72190 0.04792 -0.45504
C -5.49219 0.60386 0.73611
C -5.34943 -1.27739 -1.00784
C -5.27799 -2.39840 0.03511
C -6.80873 -1.03463 -1.41144
C -4.55491 -1.68768 -2.25398
O -4.94258 0.24702 1.90682
O -6.49015 1.27497 0.64900
C -5.60191 0.73074 3.08778
C 3.78997 -0.39313 1.75534
C 4.56058 -1.55109 1.09855
C 5.66222 -1.07830 0.14310
C 6.45190 -2.24994 -0.44368
C 5.53206 -3.25633 -1.13320
C 4.44019 -3.74056 -0.18010
C 3.64793 -2.57133 0.40873
H 5.88403 3.95060 -0.23313
H 5.54308 1.81035 0.93651
H 1.83051 3.93300 -1.65571
H 4.06529 5.00248 -1.53186
H 1.08140 -1.50627 1.55826
H -1.22240 -1.35193 0.78510
H -0.38751 2.28248 -1.34217
H -3.07761 -0.45421 0.75947
H -4.79866 0.80159 -1.24196
H -5.68086 -3.32434 -0.38589
H -4.24600 -2.59841 0.33855
H -5.85783 -2.16177 0.93178
H -7.21662 -1.94081 -1.86933
H -7.43781 -0.77656 -0.55699
H -6.88745 -0.22395 -2.14216
H -3.50778 -1.88623 -2.01380
H -4.98285 -2.59918 -2.68195
H -4.58680 -0.90614 -3.01861
H -6.62324 0.35041 3.13476
H -5.01586 0.35672 3.92402
H -5.62012 1.82133 3.08997
H 4.49089 0.24469 2.30226
H 3.09525 -0.79498 2.49911
H 5.05234 -2.06469 1.93916
H 5.21028 -0.50250 -0.67447
H 6.34391 -0.39960 0.66792
H 7.20459 -1.87597 -1.14575
H 7.00069 -2.75573 0.36234
H 5.06585 -2.78143 -2.00677
H 6.11149 -4.10564 -1.51074
H 3.76040 -4.42784 -0.69477
H 4.90186 -4.31153 0.63690
H 3.08957 -2.07328 -0.39411
H 2.90669 -2.95054 1.12134
```

66
mmff-M2
C -3.61328 3.96888 0.93643
C -3.98428 2.79791 0.28838
C -2.97329 1.91199 -0.08484
C -1.61183 2.19346 0.18768
C -1.26622 3.37593 0.84470
C -2.27034 4.25892 1.21540
N -3.06354 0.69347 -0.74291
C -1.79281 0.18700 -0.90518
C -0.85295 1.08325 -0.33584
C -1.37886 -0.98898 -1.53676
C -0.02231 -1.25632 -1.58195
C 0.92737 -0.39390 -1.00211
C 0.50576 0.78525 -0.38682
C 2.36309 -0.79090 -1.11548
N 3.22805 -0.16819 -0.26092
O 2.73873 -1.62020 -1.93698
C 4.62447 -0.54591 -0.17691
C 4.89093 -0.97538 1.25881
C 5.59010 0.58877 -0.66051
C 5.40252 1.86060 0.17379
C 7.04552 0.11525 -0.56610
C 5.26083 0.88785 -2.12827
O 5.79391 -1.95297 1.33326
O 4.34253 -0.48970 2.22153
C 6.12669 -2.40574 2.65671
C -4.27829 0.05744 -1.22392
C -4.78019 -1.09987 -0.35292
C -5.96984 -1.78770 -1.03235
C -6.50197 -2.95517 -0.19950
C -6.85966 -2.51516 1.22055
C -5.67712 -1.82507 1.90096
C -5.15393 -0.65607 1.06452
H -4.38342 4.67242 1.23502
H -5.02834 2.58789 0.08819
H -0.22722 3.60260 1.06096
H -2.01722 5.18189 1.72555
H -2.08900 -1.67532 -1.98290
H 0.34129 -2.15168 -2.07218
H 1.22119 1.49022 0.02321
H 2.85235 0.28861 0.55727
H 4.76459 -1.40962 -0.83007
H 6.06418 2.64890 -0.19740
H 4.37608 2.23191 0.10496
H 5.63560 1.69657 1.22936
H 7.70503 0.86984 -1.00488
H 7.36378 -0.04245 0.46734
H 7.19625 -0.82116 -1.11170
H 4.22884 1.22750 -2.24374
H 5.91991 1.67705 -2.50260
H 5.40031 0.00233 -2.75493
H 5.23721 -2.78712 3.15968
H 6.85724 -3.19887 2.51710
H 6.55368 -1.58996 3.24180
H -5.04961 0.82828 -1.30404
H -4.09129 -0.30056 -2.24224
H -3.96753 -1.83579 -0.27182
H -6.77264 -1.05076 -1.17795
H -5.68202 -2.13676 -2.03099
H -7.37345 -3.39906 -0.69238
H -5.73648 -3.74112 -0.15230
H -7.70850 -1.81914 1.17976
H -7.19027 -3.37573 1.81187
H -5.96307 -1.47256 2.89757
H -4.86823 -2.55309 2.04843
H -5.93160 0.11846 1.00105
H -4.29108 -0.19578 1.55641
66
mmff-M3
C 3.51720 4.04833 0.41971
C 3.87844 2.83247 -0.14660
C 2.89437 1.84943 -0.25522
C 1.56999 2.07782 0.18998
C 1.23388 3.30815 0.75773
C 2.21140 4.28700 0.87069
N 2.98539 0.57183 -0.78608
C 1.74760 -0.02922 -0.69984
C 0.83116 0.87218 -0.09861
C 1.34272 -1.30590 -1.09881
C 0.02226 -1.66194 -0.89304
C -0.90773 -0.77904 -0.31223
C -0.49354 0.48973 0.09329
C -2.30411 -1.28112 -0.14097
N -3.26172 -0.33049 0.07311
O -2.56802 -2.47796 -0.18266
C -4.67166 -0.66166 0.11901
C -5.35993 0.14298 -0.97446
C -5.30500 -0.42907 1.53270
C -5.15603 1.03325 1.96670
C -6.78849 -0.81659 1.51324
C -4.56943 -1.33424 2.52839
O -6.38126 -0.51721 -1.51987
O -5.01639 1.25226 -1.31365
C -7.10641 0.17930 -2.54768
C 4.19213 -0.00467 -1.35377
C 5.20662 -0.50757 -0.32023
C 6.49716 -0.94232 -1.02423
C 7.54256 -1.45110 -0.03021
C 6.98661 -2.57709 0.84215
C 5.69780 -2.14879 1.54431
C 4.65582 -1.64594 0.54368
H 4.26477 4.82885 0.51533
H 4.89269 2.66247 -0.48892
H 0.22292 3.49857 1.10296
H 1.96514 5.24746 1.31017
H 2.03382 -2.00778 -1.55001
H -0.32739 -2.64703 -1.17839
H -1.17896 1.17473 0.58140
H -3.04971 0.62760 -0.16405
H -4.76118 -1.72310 -0.12087
H -5.57850 1.16825 2.96685
H -4.10384 1.32851 2.01045
H -5.67405 1.71704 1.28881
H -7.19654 -0.75322 2.52634
H -7.38093 -0.15598 0.87543
H -6.92811 -1.84208 1.15808
H -3.50453 -1.09411 2.57337
H -4.98836 -1.20067 3.53039
H -4.66974 -2.38849 2.25521
H -6.44121 0.42969 -3.37498
H -7.88106 -0.51018 -2.87450
H -7.54979 1.09261 -2.14831
H 3.89370 -0.82357 -2.01411
H 4.65893 0.75371 -1.99191
H 5.45259 0.33175 0.34566
H 6.26055 -1.74078 -1.74196
H 6.90546 -0.10913 -1.60828
H 8.43516 -1.78940 -0.56713
H 7.86149 -0.61978 0.61261
H 6.77993 -3.45298 0.21235
H 7.73490 -2.89136 1.57758
H 5.28582 -2.98021 2.12584
H 5.92414 -1.34879 2.26187
H 4.35570 -2.47613 -0.11149
H 3.75362 -1.31889 1.07038
66
mmff-M4
C 3.50198 4.09216 0.11467
C 3.86858 2.84190 -0.36615
C 2.89061 1.84786 -0.40756
C 1.56561 2.10198 0.02462
C 1.22400 3.36701 0.50657
C 2.19570 4.35656 0.54968
N 2.98351 0.53327 -0.84243
C 1.74892 -0.06248 -0.70644
C 0.83043 0.87568 -0.17069
C 1.35220 -1.36619 -1.01637
C 0.03366 -1.71386 -0.78319
C -0.89886 -0.79640 -0.26305
C -0.49179 0.50084 0.05020
C -2.29199 -1.29256 -0.05066
N -3.25454 -0.33477 0.09753
O -2.54864 -2.49079 -0.00387
C -4.66197 -0.67045 0.17775
C -5.36492 0.06258 -0.95595
C -5.28295 -0.35565 1.58116
C -5.14752 1.13298 1.91927
C -6.76151 -0.76161 1.60380
C -4.52541 -1.18683 2.62391
O -6.39095 -0.63216 -1.44662
O -5.02790 1.14881 -1.36839
C -7.13016 -0.00245 -2.50705
C 4.16733 -0.12053 -1.37399
C 4.88418 -1.04749 -0.38541
C 5.40642 -0.30792 0.84997
C 6.14012 -1.25301 1.80300
C 7.27567 -1.99187 1.09428
C 6.76868 -2.72737 -0.14671
C 6.02670 -1.78372 -1.09469
H 4.24693 4.87996 0.15574
H 4.88509 2.65491 -0.69169
H 0.21285 3.57403 0.84160
H 1.94547 5.34399 0.92196
H 2.04788 -2.09111 -1.42264
H -0.31232 -2.71794 -0.99818
H -1.18125 1.21497 0.48817
H -3.05017 0.60551 -0.20799
H -4.74789 -1.74506 0.00424
H -5.56057 1.32674 2.91367
H -4.09870 1.44281 1.93156
H -5.68136 1.76601 1.20530
H -7.15966 -0.63696 2.61516
H -7.36920 -0.15180 0.93081
H -6.89138 -1.80989 1.31824
H -3.46372 -0.92986 2.64300
H -4.93699 -0.99634 3.61975
H -4.61311 -2.25735 2.41807
H -6.47338 0.20675 -3.35227
H -7.90043 -0.71665 -2.78785
H -7.57974 0.92788 -2.15684
H 3.86843 -0.68790 -2.26211
H 4.85149 0.65872 -1.72083
H 4.15921 -1.80033 -0.04472
H 6.09641 0.48415 0.52562
H 4.58111 0.18790 1.37084
H 6.52668 -0.69237 2.66062
H 5.42665 -1.98460 2.20519
H 8.04541 -1.26756 0.79506
H 7.75924 -2.69530 1.78048
H 7.60100 -3.20700 -0.67270
H 6.08955 -3.53295 0.16266
H 6.73368 -1.04329 -1.49561
H 5.63788 -2.34138 -1.95480
66
mmff-M5
C 4.92223 3.52028 0.75933
C 4.79758 2.30044 0.10650
C 3.51214 1.78674 -0.06717
C 2.37000 2.48100 0.39843
C 2.52306 3.70373 1.05412
C 3.80071 4.21622 1.23191
N 3.11374 0.61178 -0.68726
C 1.73824 0.53414 -0.63810
C 1.22826 1.67809 0.03073
C 0.87915 -0.44485 -1.14180
C -0.48361 -0.27282 -0.96112
C -1.00901 0.84935 -0.29089
C -0.14353 1.82932 0.19287
C -2.47180 1.07341 -0.08739
N -3.27806 -0.01513 -0.26327
O -2.91892 2.17595 0.21000
C -4.69769 0.02917 0.02286
C -4.97959 -1.02684 1.08202
C -5.58010 -0.14708 -1.25918
C -5.28308 -1.48211 -1.95096
C -7.06574 -0.07510 -0.88646
C -5.25153 1.00707 -2.21417
O -5.94284 -0.65194 1.92352
O -4.39341 -2.08259 1.15568
C -6.29253 -1.60028 2.94602
C 4.01607 -0.33638 -1.31735
C 4.73892 -1.27478 -0.34426
C 5.79189 -2.09651 -1.09665
C 6.54313 -3.04756 -0.16350
C 5.58264 -3.95689 0.60363
C 4.52821 -3.14207 1.35325
C 3.77847 -2.19686 0.41263
H 5.91138 3.94105 0.90700
H 5.67433 1.77298 -0.25124
H 1.65624 4.24654 1.41640
H 3.93574 5.16509 1.73952
H 1.25276 -1.31620 -1.66598
H -1.14891 -1.01944 -1.38088
H -0.56495 2.69726 0.68704
H -2.86007 -0.93366 -0.24624
H -4.91033 1.01259 0.44675
H -5.88963 -1.57337 -2.85693
H -4.23289 -1.54978 -2.24916
H -5.51051 -2.33637 -1.30760
H -7.67286 -0.09239 -1.79639
H -7.37752 -0.91768 -0.26417
H -7.29700 0.84735 -0.34531
H -4.19853 0.99542 -2.50540
H -5.85431 0.91926 -3.12320
H -5.46703 1.97609 -1.75501
H -5.42556 -1.81794 3.57104
H -7.07506 -1.12209 3.53017
H -6.65929 -2.52498 2.49812
H 3.43930 -0.91883 -2.04120
H 4.75157 0.23424 -1.89489
H 5.26247 -0.65276 0.39557
H 5.29373 -2.67977 -1.88431
H 6.49834 -1.42827 -1.60303
H 7.25908 -3.64590 -0.73696
H 7.13173 -2.45805 0.55202
H 5.08129 -4.63106 -0.10401
H 6.13842 -4.59345 1.30036
H 3.81914 -3.80730 1.85713
H 5.01695 -2.55361 2.14124
H 3.20928 -2.79133 -0.31651
H 3.04915 -1.60446 0.97442
66
mmff-M6
C 4.78214 3.71461 -0.31753
C 4.67169 2.45748 0.26305
C 3.42217 1.83751 0.23378
C 2.30080 2.46388 -0.36105
C 2.43938 3.72637 -0.94010
C 3.68183 4.34467 -0.91578
N 3.04158 0.60571 0.74523
C 1.69750 0.42431 0.49854
C 1.19119 1.55627 -0.19277
C 0.86552 -0.64933 0.82345
C -0.46946 -0.57328 0.46124
C -0.99260 0.54290 -0.22079
C -0.14960 1.60170 -0.55488
C -2.42164 0.65424 -0.64197
N -3.29366 -0.21709 -0.05398
O -2.78410 1.47652 -1.47667
C -4.72350 -0.13420 -0.27287
C -5.37901 0.05767 1.08728
C -5.29039 -1.37027 -1.05050
C -5.00769 -2.67186 -0.29228
C -6.80058 -1.20983 -1.26242
C -4.60004 -1.41686 -2.41923
O -6.46688 0.82460 1.01887
O -4.95878 -0.42596 2.11353
C -7.16699 1.03942 2.25619
C 3.92944 -0.31755 1.43048
C 4.83806 -1.13397 0.50359
C 4.05025 -2.03052 -0.45632
C 4.97985 -2.85485 -1.34844
C 5.96197 -3.68207 -0.51890
C 6.75035 -2.79885 0.44884
C 5.82104 -1.96676 1.33444
H 5.74343 4.21770 -0.30782
H 5.53214 1.98267 0.72025
H 1.58809 4.21765 -1.39976
H 3.80543 5.32545 -1.36195
H 1.24225 -1.52908 1.33104
H -1.10465 -1.42248 0.68844
H -0.56420 2.44487 -1.09532
H -3.01141 -0.68649 0.79380
H -4.90623 0.75713 -0.87638
H -5.38718 -3.52403 -0.86394
H -3.93377 -2.82222 -0.14959
H -5.48736 -2.68671 0.69015
H -7.16914 -2.02097 -1.89735
H -7.35712 -1.24580 -0.32263
H -7.03531 -0.26242 -1.75693
H -3.51840 -1.53128 -2.31574
H -4.97601 -2.26789 -2.99526
H -4.79259 -0.50551 -2.99253
H -6.51094 1.52141 2.98205
H -8.00408 1.68722 2.00739
H -7.52335 0.09124 2.66140
H 4.54008 0.26057 2.13262
H 3.31275 -0.98769 2.03586
H 5.42325 -0.42693 -0.10139
H 3.41742 -2.71136 0.13079
H 3.37603 -1.42589 -1.07117
H 4.38965 -3.50570 -2.00209
H 5.54095 -2.17837 -2.00690
H 5.40457 -4.43614 0.05323
H 6.64663 -4.23098 -1.17424
H 7.41122 -3.41066 1.07206
H 7.39934 -2.12395 -0.12498
H 5.25207 -2.63797 1.99357
H 6.40892 -1.31199 1.98844
66
mmff-M74
C -3.93320 3.83752 1.05194
C -4.14516 2.67736 0.31821
C -3.03571 1.88454 0.02262
C -1.73508 2.24606 0.44944
C -1.55025 3.41570 1.18899
C -2.65210 4.20529 1.48685
N -2.96959 0.70115 -0.69646
C -1.65390 0.29222 -0.74985
C -0.84680 1.22108 -0.04309
C -1.08620 -0.81692 -1.38267
C 0.28405 -0.98360 -1.29563
C 1.10115 -0.08844 -0.57957
C 0.52890 1.02286 0.03909
C 2.56696 -0.37716 -0.56214
N 3.27806 0.17600 0.45539
O 3.09591 -1.08101 -1.41675
C 4.71826 0.04990 0.62043
C 5.42044 0.12091 -0.74104
C 5.15787 -1.11783 1.55141
C 4.87590 -2.49410 0.93981
C 6.65390 -0.97390 1.86898
C 4.37683 -0.98598 2.86828
O 5.00663 1.20444 -1.41117
O 6.29400 -0.60900 -1.13604
C 5.57481 1.37904 -2.71532
C -4.10896 0.03916 -1.30865
C -4.98160 -0.75920 -0.33313
C -4.22233 -1.91060 0.33276
C -5.12328 -2.70697 1.27823
C -6.36946 -3.22461 0.55944
C -7.13205 -2.08533 -0.11775
C -6.22803 -1.28329 -1.05547
H -4.78011 4.47110 1.29384
H -5.14199 2.40568 -0.00931
H -0.55965 3.70650 1.52322
H -2.52374 5.11718 2.05975
H -1.68925 -1.53210 -1.92920
H 0.76235 -1.82346 -1.78569
H 1.13984 1.75806 0.55346
H 2.78197 0.68423 1.16768
H 5.03325 0.97462 1.12264
H 5.16379 -3.27567 1.65070
H 3.81414 -2.61850 0.71481
H 5.43872 -2.64005 0.01754
H 6.94899 -1.73154 2.60123
H 7.26741 -1.10240 0.97728
H 6.87345 0.00788 2.30405
H 3.30415 -1.13694 2.72029
H 4.72175 -1.74192 3.57950
H 4.52804 -0.00357 3.33068
H 5.33459 0.52289 -3.34743
H 5.12125 2.28414 -3.11350
H 6.65865 1.49061 -2.65104
H -4.71464 0.80380 -1.80725
H -3.73089 -0.61832 -2.09622
H -5.31490 -0.07342 0.45872
H -3.83730 -2.58135 -0.44849
H -3.35212 -1.52641 0.87457
H -4.56215 -3.53926 1.71599
H -5.42775 -2.06266 2.11389
H -6.06847 -3.95892 -0.19996
H -7.02278 -3.75272 1.26214
H -7.99100 -2.47791 -0.67226
H -7.53758 -1.41440 0.65129
H -5.91314 -1.92428 -1.89130
H -6.78621 -0.44857 -1.49556
66
mmff-M76
C 3.87295 3.88506 0.97893
C 4.08918 2.73073 0.23731

C 2.99553 1.89738 -0.00003
C 1.70562 2.21412 0.49021
C 1.51636 3.37929 1.23570
C 2.60294 4.20827 1.47719
N 2.93639 0.70861 -0.71071
C 1.63535 0.25253 -0.69712
C 0.82983 1.15981 0.03882
C 1.08354 -0.89662 -1.26982
C -0.27098 -1.11989 -1.10147
C -1.09254 -0.22534 -0.38959
C -0.53212 0.91291 0.18967
C -2.53743 -0.58498 -0.26661
N -3.39240 0.43686 0.00131
O -2.92885 -1.73962 -0.40533
C -4.82287 0.28170 0.21845
C -5.10609 -0.99742 1.01567
C -5.69389 0.49550 -1.05427
C -5.50948 -0.62098 -2.08727
C -7.17031 0.59842 -0.64284
C -5.28032 1.83444 -1.68459
O -4.34224 -1.02833 2.11565
O -5.95422 -1.81865 0.77484
C -4.49314 -2.19008 2.94135
C 4.06950 0.07855 -1.36662
C 5.01521 -0.67365 -0.42310
C 6.24671 -1.15965 -1.19600
C 7.22310 -1.91352 -0.29154
C 6.53687 -3.07164 0.43371
C 5.30582 -2.59206 1.20315
C 4.33213 -1.84371 0.29113
H 4.70779 4.54904 1.17729
H 5.07746 2.49342 -0.13923
H 0.53431 3.63525 1.61986
H 2.47121 5.11617 2.05565
H 1.68852 -1.60401 -1.82435
H -0.73213 -2.00704 -1.51932
H -1.13091 1.59483 0.78531
H -3.02720 1.37347 0.03269
H -5.09758 1.08658 0.91361
H -6.09869 -0.39210 -2.98146
H -4.46350 -0.71530 -2.38759
H -5.83904 -1.58217 -1.69215
H -7.77973 0.84425 -1.51778
H -7.53633 -0.33903 -0.22390
H -7.32060 1.39295 0.09710
H -4.25219 1.80735 -2.05537
H -5.93205 2.05976 -2.53356
H -5.36957 2.66160 -0.97067
H -4.23802 -3.08812 2.37654
H -3.80062 -2.05284 3.76903
H -5.51761 -2.27050 3.30906
H 3.67903 -0.60286 -2.12730
H 4.62347 0.85698 -1.90272
H 5.35784 0.03488 0.34431
H 5.91920 -1.82359 -2.00878
H 6.75141 -0.31021 -1.67123
H 8.07050 -2.28051 -0.88031
H 7.63675 -1.21706 0.44997
H 6.23004 -3.82713 -0.30221
H 7.24158 -3.56490 1.11166
H 4.79790 -3.43902 1.67623
H 5.62303 -1.92539 2.01622
H 3.93774 -2.53957 -0.46300
H 3.47370 -1.48563 0.86841
66
mmff-M7
C 4.94403 3.54036 0.63844
C 4.81375 2.30914 0.00910
C 3.52779 1.79134 -0.14714
C 2.39047 2.49505 0.31934
C 2.54919 3.72941 0.95171
C 3.82745 4.24616 1.10805
N 3.11814 0.60375 -0.73817
C 1.74462 0.53008 -0.66787
C 1.24324 1.68552 -0.01499
C 0.88269 -0.45755 -1.14956
C -0.47837 -0.28404 -0.95593
C -0.99627 0.84698 -0.29464
C -0.12658 1.83668 0.16227
C -2.45610 1.07152 -0.06782
N -3.26933 -0.01120 -0.24557
O -2.89514 2.17140 0.25094
C -4.68189 0.03232 0.07508
C -4.94445 -1.05986 1.10205
C -5.59686 -0.09301 -1.19030
C -5.33503 -1.41030 -1.92862
C -7.07217 -0.01134 -0.78025
C -5.27491 1.08473 -2.11836
O -5.89468 -0.71732 1.97132
O -4.35494 -2.11607 1.12949
C -6.22692 -1.70263 2.96439
C 3.97272 -0.39391 -1.35879
C 4.22292 -1.64136 -0.50348
C 4.94243 -1.32407 0.81080
C 5.20551 -2.58953 1.62888
C 5.98670 -3.62593 0.82072
C 5.28174 -3.94573 -0.49789
C 5.01056 -2.67911 -1.31147
H 5.93500 3.96209 0.77046
H 5.68968 1.77567 -0.34082
H 1.68496 4.27663 1.31360
H 3.96711 5.20415 1.59689
H 1.25427 -1.33326 -1.66848
H -1.14707 -1.03763 -1.35724
H -0.54485 2.71130 0.64716
H -2.85569 -0.93188 -0.25268
H -4.87854 1.00136 0.53823
H -5.96216 -1.46494 -2.82343
H -4.29256 -1.48355 -2.25138
H -5.56131 -2.28087 -1.30699
H -7.70048 0.00668 -1.67567
H -7.38191 -0.86674 -0.17494
H -7.27676 0.89831 -0.20756
H -4.23019 1.06613 -2.43756
H -5.90241 1.03323 -3.01335
H -5.46322 2.04237 -1.62477
H -5.34982 -1.94162 3.56705
H -7.00031 -1.24701 3.57800
H -6.60018 -2.61092 2.48912
H 3.51566 -0.68445 -2.31115
H 4.92282 0.08734 -1.60647
H 3.24586 -2.07953 -0.25454
H 5.90106 -0.83677 0.58264
H 4.35828 -0.60881 1.39856
H 5.74600 -2.33496 2.54665
H 4.24604 -3.02219 1.94244
H 6.99072 -3.23466 0.60744
H 6.12637 -4.54025 1.40726
H 5.87819 -4.64888 -1.08888
H 4.32820 -4.44729 -0.28541
H 5.96757 -2.23684 -1.62332
H 4.46851 -2.92976 -2.23094
66
mmff-M8
C 4.76546 3.74733 -0.09778
C 4.65803 2.45764 0.40630
C 3.41332 1.83193 0.33243
C 2.29395 2.48757 -0.23639
C 2.42952 3.78279 -0.73906
C 3.66690 4.40663 -0.66695
N 3.03150 0.56817 0.76207
C 1.69205 0.39865 0.48935
C 1.18668 1.56714 -0.13662
C 0.86454 -0.69587 0.74956
C -0.46963 -0.60322 0.38689
C -0.99304 0.54953 -0.23086
C -0.15286 1.62917 -0.50079
C -2.42329 0.68395 -0.64203
N -3.29400 -0.21754 -0.09945
O -2.78799 1.55184 -1.42812
C -4.72544 -0.11501 -0.30025
C -5.36677 -0.02309 1.07703
C -5.30340 -1.28854 -1.16230
C -5.03434 -2.64229 -0.49613
C -6.81150 -1.09831 -1.36404
C -4.61175 -1.24668 -2.53047
O -6.45867 0.74080 1.07570
O -4.93220 -0.57585 2.06161
C -7.14456 0.86419 2.33346
C 3.87375 -0.41321 1.42435
C 4.35820 -1.55300 0.52066
C 5.08158 -2.61377 1.35848
C 5.57978 -3.77507 0.49657
C 6.46387 -3.28457 -0.65037
C 5.74777 -2.22393 -1.48709
C 5.25664 -1.06397 -0.61921
H 5.72428 4.25304 -0.05062
H 5.52041 1.96236 0.83671
H 1.57875 4.29388 -1.17766
H 3.78826 5.41283 -1.05302
H 1.24392 -1.59955 1.21160
H -1.10418 -1.46493 0.56285
H -0.56987 2.50182 -0.99012
H -3.00887 -0.73394 0.71973
H -4.91106 0.81881 -0.83484
H -5.41401 -3.44954 -1.12951
H -3.96245 -2.80887 -0.35601
H -5.52210 -2.72409 0.47904
H -7.18834 -1.86327 -2.04942
H -7.36947 -1.18698 -0.42875
H -7.03498 -0.11898 -1.79801
H -3.53243 -1.38674 -2.43530
H -5.00132 -2.04680 -3.16717
H -4.78763 -0.29255 -3.03522
H -6.48172 1.29716 3.08362
H -7.98741 1.52356 2.14094
H -7.49172 -0.11209 2.67489
H 4.72985 0.11519 1.85280
H 3.30989 -0.82499 2.26857
H 3.47260 -2.02512 0.07194
H 5.93723 -2.14577 1.86598
H 4.41736 -2.98687 2.14693
H 6.12432 -4.49443 1.11743
H 4.71562 -4.31164 0.08268
H 7.38636 -2.85507 -0.23667
H 6.76762 -4.12658 -1.28146
H 6.41011 -1.84728 -2.27363
H 4.88918 -2.68207 -1.99602
H 6.12426 -0.54360 -0.18894
H 4.72321 -0.33164 -1.23359
66
mmff-M95
C -4.30167 3.83959 0.62212
C -4.35871 2.61232 -0.02610
C -3.17550 1.88096 -0.13459
C -1.95509 2.36966 0.38986
C -1.92545 3.60475 1.03954
C -3.10153 4.33292 1.15309
N -2.95640 0.64971 -0.73480
C -1.61966 0.33487 -0.61501
C -0.95362 1.37732 0.08166
C -0.92197 -0.79004 -1.05986
C 0.44081 -0.85130 -0.81640
C 1.12199 0.17694 -0.13587
C 0.41151 1.28489 0.32382
C 2.58605 0.14267 0.16009
N 3.34233 -0.68673 -0.60589
O 3.08114 0.83421 1.04489
C 4.77438 -0.88098 -0.43682
C 5.13922 -0.89355 1.05307
C 5.65715 0.02551 -1.34364
C 5.57667 1.50447 -0.95149
C 7.11232 -0.46232 -1.27962
C 5.16366 -0.13706 -2.78963
O 4.35781 -1.76398 1.70630
O 6.05411 -0.30554 1.57184
C 4.58418 -1.86151 3.11822
C -3.97924 -0.14482 -1.39280
C -4.89483 -0.92230 -0.44009
C -4.13567 -1.95291 0.40083
C -5.07667 -2.73371 1.31994
C -6.20257 -3.40241 0.53081
C -6.96294 -2.38464 -0.32001
C -6.02000 -1.59811 -1.23215
H -5.20854 4.42720 0.71983
H -5.29477 2.24400 -0.42970
H -0.99673 3.99030 1.44709
H -3.09491 5.29397 1.65564
H -1.42114 -1.60630 -1.56789
H 0.96974 -1.74441 -1.13265
H 0.94508 2.05809 0.86474
H 2.90283 -1.18408 -1.36157
H 4.96531 -1.91322 -0.75980
H 6.18394 2.09909 -1.64194
H 4.54988 1.87400 -1.00287
H 5.94598 1.66444 0.06159
H 7.72232 0.10538 -1.98879
H 7.53494 -0.33211 -0.28341
H 7.18725 -1.52084 -1.55411
H 4.14817 0.24744 -2.91698
H 5.81562 0.42094 -3.46775
H 5.17920 -1.18685 -3.10476
H 4.40563 -0.89675 3.59558
H 3.87129 -2.60014 3.47844
H 5.60632 -2.18487 3.32283
H -4.57928 0.52766 -2.01561
H -3.47834 -0.83526 -2.07712
H -5.35472 -0.19952 0.24863
H -3.62542 -2.65576 -0.27333
H -3.35600 -1.45974 0.99008
H -4.51069 -3.48151 1.88534
H -5.51003 -2.04696 2.05924
H -5.77511 -4.17324 -0.12477
H -6.89016 -3.91737 1.21018
H -7.73061 -2.88577 -0.91918
H -7.49097 -1.68397 0.34042
H -5.57622 -2.28134 -1.97047
H -6.58278 -0.84811 -1.80025
66
mmff-M9
C -3.47420 3.95583 0.94302
C -3.86541 2.79939 0.28038
C -2.86820 1.90085 -0.10035
C -1.50231 2.15412 0.17299
C -1.13652 3.32264 0.84376
C -2.12622 4.21756 1.22586
N -2.98438 0.69601 -0.77631
C -1.72351 0.16636 -0.95067
C -0.76613 1.03790 -0.36980
C -1.32775 -1.00989 -1.59319
C 0.02355 -1.30055 -1.64140
C 0.98921 -0.46229 -1.05243
C 0.58728 0.71648 -0.42429
C 2.41753 -0.88391 -1.17073
N 3.29022 -0.30061 -0.29657
O 2.77955 -1.70197 -2.00930
C 4.68529 -0.68845 -0.23625
C 5.02994 -1.26405 1.13140
C 5.66012 0.46146 -0.66493
C 5.54256 1.66362 0.27863
C 7.10390 -0.05509 -0.67326
C 5.28179 0.89023 -2.08819
O 4.29437 -0.71789 2.11151
O 5.87471 -2.10245 1.32449
C 4.54891 -1.20437 3.43893
C -4.23689 0.12474 -1.24081
C -5.06272 -0.56896 -0.15127
C -4.34135 -1.77494 0.45785
C -5.19780 -2.46736 1.51942
C -6.55971 -2.87764 0.95875
C -7.28698 -1.68283 0.34125
C -6.42664 -0.98535 -0.71385
H -4.23109 4.67047 1.24895
H -4.91203 2.61027 0.07108
H -0.09383 3.53114 1.06068
H -1.85707 5.12983 1.74700
H -2.04761 -1.68258 -2.04389
H 0.37021 -2.19744 -2.14102
H 1.31370 1.40538 -0.00601
H 2.91476 0.17034 0.51110
H 4.80959 -1.50744 -0.94825
H 6.19735 2.47057 -0.06338
H 4.52170 2.05670 0.30077
H 5.83343 1.41129 1.30229
H 7.76945 0.72692 -1.05097
H 7.44891 -0.33806 0.32344
H 7.20780 -0.92848 -1.32425
H 4.26203 1.27990 -2.13154
H 5.95974 1.67830 -2.42945
H 5.35620 0.05207 -2.78700
H 5.58385 -1.00866 3.72272
H 3.86526 -0.65996 4.08611
H 4.35456 -2.27637 3.49158
H -4.82461 0.92905 -1.69654
H -4.00597 -0.58211 -2.04251
H -5.23826 0.16000 0.65263
H -4.11036 -2.49226 -0.34259
H -3.38379 -1.46494 0.88846
H -4.66876 -3.34123 1.91400
H -5.34509 -1.78366 2.36618
H -6.41554 -3.64901 0.19017
H -7.17420 -3.33081 1.74397
H -8.23643 -2.00141 -0.10200
H -7.53781 -0.96406 1.13274
H -6.27014 -1.66682 -1.56220
H -6.95255 -0.10909 -1.11075
66
pm6-M13
C -3.66002 3.93421 -0.32773
C -4.00849 2.64449 -0.70720
C -3.02006 1.66068 -0.66211
C -1.69860 1.97071 -0.25950
C -1.37501 3.27324 0.12524
C -2.36014 4.24950 0.09154
N -3.09480 0.31343 -0.99519
C -1.83676 -0.23890 -0.85461
C -0.93740 0.75269 -0.38702
C -1.39547 -1.53975 -1.11243
C -0.06270 -1.83253 -0.88534
C 0.84662 -0.86279 -0.42334
C 0.40011 0.43333 -0.16871
C 2.25644 -1.30534 -0.19878
N 3.20479 -0.32819 -0.23938
O 2.53924 -2.48519 -0.01769
C 4.58323 -0.55607 0.13886
C 4.91177 0.42115 1.25973
C 5.57616 -0.44720 -1.06721
C 5.48813 0.93201 -1.73053
C 7.01000 -0.69888 -0.58499
C 5.19755 -1.52807 -2.08713
O 5.72849 -0.11209 2.16893
O 4.47783 1.54848 1.32201
C 6.11296 0.74931 3.25393
C -4.26553 -0.36245 -1.53982
C -5.19946 -1.04689 -0.52741
C -5.85336 -0.06617 0.45261
C -6.82442 -0.76977 1.40291
C -6.14554 -1.91466 2.15323
C -5.50410 -2.90133 1.17870
C -4.53088 -2.20083 0.22837
H -4.41473 4.71324 -0.35573
H -5.01948 2.42104 -1.02448
H -0.36622 3.51980 0.44002
H -2.12449 5.26619 0.38655
H -2.06776 -2.30788 -1.47342
H 0.31141 -2.83451 -1.05950
H 1.06740 1.19340 0.22550
H 2.92251 0.63265 -0.35224
H 4.64099 -1.57261 0.53212
H 6.18370 0.98127 -2.57357
H 4.48490 1.12289 -2.12273
H 5.74290 1.73776 -1.03710
H 7.68568 -0.72917 -1.44481
H 7.36483 0.08712 0.08665
H 7.09201 -1.65463 -0.05871
H 4.17574 -1.39279 -2.44952
H 5.87113 -1.47673 -2.94801
H 5.27468 -2.52875 -1.65291
H 6.76158 0.14808 3.88646
H 6.64776 1.62177 2.87574
H 5.23153 1.07515 3.80766
H -4.83170 0.37828 -2.11258
H -3.90893 -1.10297 -2.26208
H -6.00199 -1.47942 -1.14477
H -5.07257 0.43418 1.03941
H -6.38252 0.71728 -0.10144
H -7.24385 -0.04501 2.10874
H -7.66965 -1.16820 0.82541
H -5.37139 -1.50503 2.81578
H -6.86741 -2.42948 2.79625
H -4.98317 -3.69484 1.72479
H -6.29199 -3.39200 0.59120
H -3.68083 -1.81300 0.80402
H -4.12466 -2.92756 -0.48416
66
pm6-M14
C 3.71387 3.90333 0.69731
C 4.06812 2.73718 0.03135
C 3.05843 1.81959 -0.26107
C 1.71237 2.07582 0.09550
C 1.38288 3.25180 0.77220
C 2.38779 4.16015 1.07189

N 3.13447 0.59869 -0.92086
C 1.85754 0.08451 -1.03089
C 0.94127 0.96037 -0.39525
C 1.41099 -1.07290 -1.67459
C 0.05534 -1.34723 -1.65502
C -0.86604 -0.51124 -0.99730
C -0.41657 0.65330 -0.37572
C -2.30532 -0.91046 -1.05064
N -3.10185 -0.41451 -0.06302
O -2.73276 -1.66300 -1.92018
C -4.54322 -0.54655 -0.07887
C -5.12496 0.85863 0.00075
C -5.08626 -1.49096 1.04632
C -4.69663 -0.97496 2.43628
C -6.61244 -1.59915 0.94296
C -4.47402 -2.88009 0.82948
O -6.24285 0.98221 -0.71533
O -4.62791 1.76039 0.63548
C -6.87685 2.27208 -0.67776
C 4.33278 0.03626 -1.53016
C 5.15786 -0.92736 -0.66077
C 4.39696 -2.20357 -0.28365
C 5.26626 -3.16919 0.52451
C 5.84583 -2.49621 1.76785
C 6.61519 -1.22974 1.39501
C 5.74763 -0.26183 0.58775
H 4.48463 4.62962 0.93333
H 5.09929 2.55627 -0.24585
H 0.35487 3.45594 1.05354
H 2.14763 5.07915 1.59534
H 2.09513 -1.74317 -2.17970
H -0.32871 -2.22839 -2.15528
H -1.10959 1.34938 0.08620
H -2.71732 0.23121 0.60858
H -4.81190 -0.98359 -1.04247
H -5.07021 -1.66071 3.20255
H -3.61019 -0.91722 2.55087
H -5.11446 0.01462 2.63942
H -6.97691 -2.33770 1.66303
H -7.11051 -0.65097 1.16024
H -6.92248 -1.92157 -0.05564
H -3.38429 -2.84993 0.90141
H -4.84427 -3.57087 1.59312
H -4.73830 -3.28168 -0.15256
H -7.75162 2.18461 -1.31760
H -7.17163 2.52136 0.34263
H -6.19853 3.03724 -1.05730
H 4.02469 -0.47559 -2.44681
H 4.96322 0.87399 -1.84286
H 6.00013 -1.22445 -1.30461
H 3.51112 -1.93559 0.30642
H 4.03405 -2.70330 -1.18885
H 4.68022 -4.05094 0.80450
H 6.08930 -3.52933 -0.10755
H 5.02798 -2.23475 2.45261
H 6.49655 -3.19162 2.30876
H 6.98979 -0.73068 2.29502
H 7.49713 -1.50526 0.80094
H 4.93093 0.10592 1.22193
H 6.34132 0.61286 0.29916
66
pm6-M17
C 5.03119 -3.39585 -0.35928
C 4.84395 -2.19064 0.30521
C 3.57208 -1.61769 0.27208
C 2.50211 -2.25355 -0.40221
C 2.71740 -3.46030 -1.07000
C 3.98443 -4.02509 -1.04703
N 3.11383 -0.44158 0.85535
C 1.76023 -0.33231 0.60456
C 1.33772 -1.42960 -0.18927
C 0.84763 0.63813 1.02398
C -0.47812 0.49736 0.64599
C -0.91483 -0.58151 -0.14673
C 0.00600 -1.53760 -0.57139
C -2.33090 -0.75914 -0.59165
N -3.27892 -0.11662 0.14580
O -2.61952 -1.47057 -1.54871
C -4.66889 -0.04885 -0.25256
C -5.04659 1.42460 -0.32043
C -5.61684 -0.87262 0.68308
C -5.52110 -0.38467 2.13311
C -7.06438 -0.75435 0.19087
C -5.18550 -2.34249 0.61156
O -5.89442 1.68127 -1.31693
O -4.62258 2.26642 0.43773
C -6.32612 3.04686 -1.44232
C 3.89243 0.43991 1.71574
C 4.61211 1.61314 1.02934
C 3.65253 2.60572 0.36320
C 4.39555 3.79207 -0.25487
C 5.47034 3.33152 -1.23857
C 6.43638 2.35259 -0.57306
C 5.69560 1.16385 0.04238
H 6.01310 -3.85735 -0.34537
H 5.66744 -1.71922 0.82702
H 1.90344 -3.95131 -1.59313
H 4.16810 -4.96292 -1.55985
H 1.15362 1.48921 1.61896
H -1.17227 1.27452 0.94860
H -0.34207 -2.35622 -1.19081
H -2.99766 0.49562 0.89518
H -4.73412 -0.47417 -1.25573
H -6.17936 -0.98441 2.76869
H -4.50434 -0.49064 2.52268
H -5.81870 0.66230 2.23470
H -7.70743 -1.41311 0.78190
H -7.45322 0.26246 0.28886
H -7.15405 -1.04990 -0.85880
H -4.15740 -2.47275 0.95756
H -5.83575 -2.95045 1.24799
H -5.25060 -2.72630 -0.41023
H -5.46897 3.69959 -1.61245
H -6.99505 3.06255 -2.29939
H -6.85136 3.36414 -0.54028
H 3.21548 0.82834 2.48257
H 4.62685 -0.17981 2.23916
H 5.11628 2.14394 1.85174
H 3.08301 2.08899 -0.41981
H 2.92369 2.96954 1.09623
H 3.68252 4.45855 -0.75158
H 4.86656 4.37929 0.54511
H 4.99042 2.84029 -2.09554
H 6.01590 4.19357 -1.63713
H 7.17704 1.99478 -1.29608
H 6.99599 2.87629 0.21388
H 5.23373 0.57262 -0.75848
H 6.41062 0.50560 0.54873
66
pm6-M19
C 3.39235 4.07641 0.56359
C 3.81144 2.87010 0.01800
C 2.83832 1.91311 -0.27128
C 1.46649 2.15980 -0.01720
C 1.07234 3.37993 0.53551
C 2.03870 4.33311 0.82311
N 2.97919 0.64667 -0.82032
C 1.73074 0.07459 -0.93074
C 0.75472 0.97666 -0.43643
C 1.36610 -1.16823 -1.45568
C 0.02218 -1.49642 -1.46839
C -0.96170 -0.62954 -0.95647
C -0.59000 0.61614 -0.45047
C -2.38008 -1.09567 -1.02710
N -3.24414 -0.52046 -0.14440
O -2.73378 -1.96214 -1.82007
C -4.66968 -0.76617 -0.17678
C -5.43842 0.52699 -0.42150
C -5.19220 -1.54462 1.07873
C -4.98474 -0.73147 2.36189
C -6.68118 -1.86769 0.90487
C -4.40941 -2.86004 1.17690
O -4.81553 1.59463 0.09968
O -6.49359 0.59170 -1.00179
C -5.47525 2.85780 -0.08124
C 4.22009 0.01919 -1.24236
C 4.76485 -1.03211 -0.26871
C 5.11658 -0.44351 1.10085
C 5.68234 -1.50760 2.04270
C 6.89321 -2.21105 1.42893
C 6.55748 -2.79559 0.05647
C 5.98268 -1.73308 -0.88184
H 4.13249 4.83509 0.79559
H 4.86307 2.68788 -0.16934
H 0.02498 3.58129 0.73576
H 1.74763 5.28588 1.25174
H 2.10372 -1.85938 -1.84648
H -0.30391 -2.44447 -1.87968
H -1.33351 1.32613 -0.10209
H -2.90213 0.17724 0.49519
H -4.84969 -1.39648 -1.05021
H -5.32563 -1.30969 3.22577
H -3.92850 -0.49628 2.52553
H -5.54539 0.20731 2.34844
H -7.02427 -2.47474 1.74801
H -7.29860 -0.96795 0.86453
H -6.85759 -2.43651 -0.01309
H -3.34205 -2.68085 1.32672
H -4.77610 -3.44543 2.02542
H -4.52912 -3.46069 0.27095
H -6.45535 2.84673 0.39771
H -4.82911 3.59550 0.38892
H -5.59342 3.07364 -1.14383
H 4.05090 -0.43700 -2.22378
H 4.96014 0.80992 -1.39224
H 3.98165 -1.78883 -0.11860
H 5.86370 0.35140 0.96461
H 4.23499 0.02685 1.54803
H 5.95120 -1.05261 3.00186
H 4.90224 -2.25004 2.25786
H 7.71405 -1.48886 1.32262
H 7.25570 -2.99884 2.09789
H 7.44718 -3.24912 -0.39313
H 5.82308 -3.60304 0.17725
H 6.75622 -0.98194 -1.09660
H 5.71162 -2.18541 -1.84302
66
pm6-M1
C -3.48121 4.05068 -0.21529
C -3.86762 2.75952 -0.55159
C -2.88831 1.76586 -0.54141
C -1.54404 2.05738 -0.20672
C -1.18252 3.36308 0.13006
C -2.15524 4.35313 0.12521
N -3.00313 0.41788 -0.84468
C -1.76110 -0.16792 -0.72057
C -0.81800 0.81607 -0.32593
C -1.37314 -1.49578 -0.91854
C -0.04244 -1.81915 -0.72396
C 0.91375 -0.85626 -0.34915
C 0.51619 0.46431 -0.14122
C 2.31754 -1.33332 -0.15973
N 3.29146 -0.38453 -0.24763
O 2.57389 -2.51728 0.03438
C 4.67295 -0.64430 0.09761
C 5.05519 0.33369 1.20070
C 5.63664 -0.57112 -1.13435
C 5.56813 0.80473 -1.80696
C 7.07533 -0.85749 -0.68716
C 5.20256 -1.64920 -2.13480
O 5.88043 -0.21303 2.09407
O 4.65263 1.47257 1.26332
C 6.31390 0.64694 3.16154
C -4.23452 -0.23651 -1.25237
C -5.20764 -0.53699 -0.10670
C -4.62660 -1.50665 0.92672
C -5.62980 -1.80750 2.04161
C -6.94748 -2.34188 1.47961
C -7.53333 -1.38768 0.43823
C -6.52606 -1.07898 -0.67067
H -4.22461 4.84098 -0.21533
H -4.89703 2.54062 -0.81078
H -0.15620 3.60179 0.38942
H -1.88960 5.37190 0.38559
H -2.08416 -2.26015 -1.20849
H 0.29500 -2.84047 -0.85523
H 1.21856 1.22062 0.19486
H 3.03265 0.58257 -0.36249
H 4.71504 -1.65848 0.49882
H 6.24200 0.82844 -2.66851
H 4.56033 1.01960 -2.17425
H 5.86282 1.60878 -1.12752
H 7.72774 -0.91076 -1.56368
H 7.46771 -0.07708 -0.03002
H 7.14587 -1.81189 -0.15678
H 4.17687 -1.48678 -2.47439
H 5.85670 -1.62564 -3.01173
H 5.26041 -2.64781 -1.69280
H 5.45523 0.99957 3.73430
H 6.96282 0.03442 3.78283
H 6.86091 1.50245 2.76268
H -4.72492 0.40230 -1.99534
H -3.96654 -1.16233 -1.76865
H -5.42545 0.41040 0.40646
H -4.35705 -2.44529 0.42189
H -3.70244 -1.10007 1.34981
H -5.19834 -2.52382 2.74864
H -5.82401 -0.88775 2.60948
H -6.76984 -3.32036 1.01316
H -7.66706 -2.50810 2.28839
H -8.44741 -1.80878 0.00626
H -7.82410 -0.45002 0.93040
H -6.32026 -1.99695 -1.23960
H -6.95422 -0.36147 -1.38053
66
pm6-M21
C 3.42018 4.01089 0.73198
C 3.82926 2.82482 0.13602
C 2.84636 1.88981 -0.19026
C 1.47678 2.13628 0.07069
C 1.09295 3.33550 0.67387
C 2.06841 4.26659 1.00220
N 2.98158 0.65004 -0.79543
C 1.72915 0.09084 -0.93675
C 0.75840 0.97869 -0.40490
C 1.35204 -1.12627 -1.51070
C 0.00559 -1.44161 -1.53889
C -0.97224 -0.58831 -0.99360
C -0.58945 0.63095 -0.43539
C -2.39468 -1.03715 -1.08522
N -3.25294 -0.49447 -0.17650
O -2.75713 -1.86261 -1.91709
C -4.68118 -0.72193 -0.22023
C -5.43454 0.59167 -0.39348
C -5.21211 -1.56140 0.99183
C -5.00057 -0.81903 2.31654
C -6.70325 -1.86305 0.79890
C -4.43968 -2.88624 1.02160
O -4.79826 1.62218 0.18353
O -6.48923 0.70048 -0.96799
C -5.44126 2.90166 0.06905
C 4.24350 0.06987 -1.22175
C 5.07556 -0.54498 -0.09025
C 6.44386 -0.98256 -0.62543
C 7.31108 -1.60188 0.47206
C 6.59600 -2.76187 1.16548
C 5.22970 -2.33036 1.69888
C 4.36652 -1.71688 0.59499
H 4.16571 4.75408 0.99475
H 4.87838 2.64088 -0.06505
H 0.04732 3.53926 0.88070
H 1.78513 5.20279 1.47077
H 2.08230 -1.81124 -1.92467
H -0.32681 -2.36991 -1.98848
H -1.32657 1.33217 -0.05713
H -2.90331 0.16753 0.49600
H -4.86917 -1.30197 -1.12624
H -5.34462 -1.44091 3.14826
H -3.94317 -0.59816 2.49238
H -5.55655 0.12194 2.35359
H -7.05298 -2.51037 1.60871
H -7.31339 -0.95744 0.80436
H -6.88210 -2.38186 -0.14777
H -3.37156 -2.72350 1.18413
H -4.81376 -3.51327 1.83647
H -4.56058 -3.43658 0.08444
H -6.42363 2.87723 0.54273
H -4.78799 3.60441 0.58105
H -5.55171 3.17632 -0.98076
H 4.02529 -0.68684 -1.98031
H 4.82177 0.85346 -1.72324
H 5.24345 0.23543 0.66556
H 6.29441 -1.71872 -1.42815
H 6.96085 -0.12827 -1.07807
H 8.26394 -1.93845 0.04992
H 7.55415 -0.83105 1.21552
H 6.46007 -3.58259 0.44822
H 7.21481 -3.15713 1.97811
H 4.70961 -3.18234 2.14929
H 5.36968 -1.59194 2.49969
H 4.14370 -2.48678 -0.15742
H 3.40549 -1.39012 1.00492
66
pm6-M25
C -4.80339 3.68080 -0.25821
C -4.66274 2.40835 0.28099
C -3.39640 1.82406 0.24134
C -2.28839 2.50050 -0.32270
C -2.45737 3.77737 -0.86077
C -3.71650 4.36032 -0.82613
N -2.98590 0.58652 0.71447
C -1.63533 0.45072 0.47385
C -1.15521 1.61906 -0.17418
C -0.77635 -0.61000 0.76896
C 0.56015 -0.48213 0.42629
C 1.05847 0.67239 -0.20847
C 0.18759 1.71583 -0.51840
C 2.48891 0.84295 -0.60751
N 3.39272 0.05157 0.03575
O 2.82660 1.66908 -1.44943
C 4.79313 0.01556 -0.32602
C 5.20116 -1.38015 -0.78270
C 5.73551 0.57808 0.79248
C 5.65611 -0.27331 2.06519
C 7.18129 0.61311 0.28236
C 5.28856 2.01075 1.10916
O 4.50672 -2.34265 -0.15754
O 6.05918 -1.60476 -1.59984
C 4.83145 -3.69245 -0.52641
C -3.85008 -0.37489 1.37706
C -4.74086 -1.19048 0.43266
C -5.71460 -2.05048 1.24653
C -6.62688 -2.88375 0.34475
C -5.82018 -3.74065 -0.63135
C -4.84744 -2.88650 -1.44471
C -3.93487 -2.05998 -0.53695
H -5.77824 4.15682 -0.23985
H -5.51328 1.89488 0.71435
H -1.61658 4.30666 -1.29692
H -3.86386 5.35164 -1.24063
H -1.13337 -1.51888 1.23809
H 1.21382 -1.32477 0.62638
H 0.58233 2.58855 -1.02574
H 3.06875 -0.61302 0.71822
H 4.89848 0.66301 -1.19905
H 6.29620 0.15866 2.84013
H 4.63857 -0.30701 2.46670
H 5.98948 -1.30055 1.89357
H 7.82398 1.07617 1.03712
H 7.57171 -0.38528 0.07438
H 7.26222 1.20258 -0.63592
H 4.26716 2.03573 1.49632
H 5.94808 2.44424 1.86694
H 5.33012 2.64429 0.21884
H 5.87792 -3.90595 -0.30414
H 4.17753 -4.32429 0.07023
H 4.64792 -3.84806 -1.59033
H -3.21650 -1.04429 1.96572
H -4.47356 0.17130 2.09340
H -5.33412 -0.48315 -0.16397
H -5.13847 -2.72236 1.89878
H -6.31538 -1.41424 1.90705
H -7.28125 -3.51452 0.95579
H -7.28334 -2.21071 -0.22267
H -5.25360 -4.49379 -0.06704
H -6.49289 -4.29104 -1.29769
H -4.24413 -3.51878 -2.10459
H -5.41622 -2.20992 -2.09640
H -3.29363 -2.73900 0.04319
H -3.26802 -1.43545 -1.13993
66
pm6-M265
C -3.89110 3.96074 -0.49535
C -4.10698 2.61051 -0.73914
C -3.00928 1.75301 -0.65981
C -1.71556 2.23607 -0.34786
C -1.52659 3.59781 -0.10432
C -2.61727 4.45293 -0.17816
N -2.94987 0.38339 -0.86630
C -1.64491 -0.02961 -0.70155
C -0.83689 1.09169 -0.37933
C -1.09137 -1.30875 -0.80330

C 0.26752 -1.44753 -0.58693
C 1.09140 -0.34703 -0.28375
C 0.52939 0.92445 -0.17005
C 2.54186 -0.62740 -0.06434
N 3.39000 0.42769 -0.20408
O 2.94732 -1.75098 0.21487
C 4.83523 0.33825 -0.04729
C 5.36281 -0.97952 -0.63156
C 5.34329 0.70012 1.37815
C 4.91515 -0.32823 2.43026
C 6.87413 0.82730 1.35364
C 4.75753 2.07144 1.74792
O 4.92832 -1.12124 -1.89172
O 6.13640 -1.74042 -0.10785
C 5.32788 -2.32866 -2.55179
C -4.08510 -0.44900 -1.22663
C -5.01662 -0.79929 -0.06053
C -4.32166 -1.62948 1.02279
C -5.28152 -1.99225 2.15726
C -6.52060 -2.71716 1.63108
C -7.21957 -1.90108 0.54331
C -6.25733 -1.53032 -0.58643
H -4.72916 4.64764 -0.55028
H -5.09808 2.24443 -0.98064
H -0.54189 3.98428 0.13756
H -2.48571 5.51293 0.00984
H -1.69846 -2.17538 -1.03598
H 0.73079 -2.42536 -0.64466
H 1.13259 1.78277 0.10776
H 3.02926 1.27347 -0.61229
H 5.24437 1.10323 -0.71950
H 5.26563 -0.00932 3.41738
H 3.82743 -0.42208 2.47562
H 5.33415 -1.31145 2.21452
H 7.22835 1.18556 2.32499
H 7.35392 -0.12914 1.14619
H 7.19924 1.55117 0.59749
H 3.66809 2.03636 1.82646
H 5.15168 2.39354 2.71609
H 5.02606 2.83581 1.00952
H 6.41546 -2.38766 -2.62176
H 4.95133 -3.19648 -2.00803
H 4.88452 -2.28076 -3.54415
H -4.64984 0.07269 -2.00713
H -3.69809 -1.36363 -1.68425
H -5.34979 0.14214 0.39878
H -3.93499 -2.55330 0.56935
H -3.45691 -1.08727 1.41854
H -4.76507 -2.60931 2.89996
H -5.59078 -1.07458 2.67529
H -6.22075 -3.68922 1.21667
H -7.21505 -2.92875 2.45114
H -8.07368 -2.45524 0.13981
H -7.62522 -0.98154 0.98593
H -5.93895 -2.44522 -1.10625
H -6.77050 -0.91092 -1.33146
66
pm6-M28
C -4.79302 3.70506 -0.08431
C -4.65278 2.41226 0.40339
C -3.39090 1.82209 0.32827
C -2.28703 2.51578 -0.22542
C -2.45562 3.81334 -0.71201
C -3.71009 4.40178 -0.63874
N -2.97688 0.56365 0.74335
C -1.63173 0.43468 0.47614
C -1.15534 1.62502 -0.13123
C -0.77599 -0.64031 0.72536
C 0.55785 -0.50478 0.37402
C 1.05287 0.67056 -0.22364
C 0.18414 1.72901 -0.48647
C 2.48080 0.84831 -0.62903
N 3.38749 0.04836 -0.00072
O 2.81315 1.68640 -1.46097
C 4.78716 0.01885 -0.36580
C 5.19706 -1.37203 -0.83543
C 5.73086 0.57304 0.75592
C 5.65591 -0.29024 2.02087
C 7.17547 0.61575 0.24305
C 5.28187 2.00181 1.08701
O 4.50566 -2.34128 -0.21729
O 6.05413 -1.58790 -1.65587
C 4.83308 -3.68722 -0.59775
C -3.79569 -0.44850 1.38840
C -4.24605 -1.58875 0.46779
C -5.14914 -1.10850 -0.67212
C -5.60574 -2.26991 -1.55676
C -6.30054 -3.35785 -0.73731
C -5.41173 -3.84006 0.40948
C -4.94812 -2.67707 1.28824
H -5.76567 4.18352 -0.03608
H -5.50345 1.88765 0.82220
H -1.61712 4.35340 -1.13928
H -3.85719 5.40916 -1.01254
H -1.13352 -1.56202 1.16880
H 1.21229 -1.35487 0.53650
H 0.57838 2.61866 -0.96394
H 3.06653 -0.62174 0.67788
H 4.88938 0.67444 -1.23311
H 6.29690 0.13585 2.79832
H 4.63936 -0.32958 2.42438
H 5.99085 -1.31522 1.83913
H 7.81876 1.07323 1.00070
H 7.56754 -0.37985 0.02510
H 7.25322 1.21384 -0.66993
H 4.26124 2.02131 1.47651
H 5.94211 2.42920 1.84763
H 5.32056 2.64393 0.20275
H 5.88022 -3.90022 -0.37814
H 4.18114 -4.32550 -0.00584
H 4.64904 -3.83433 -1.66279
H -3.22495 -0.85658 2.22988
H -4.66725 0.05157 1.81973
H -3.34624 -2.03340 0.01920
H -6.03219 -0.61501 -0.24166
H -4.63019 -0.35593 -1.27430
H -6.27227 -1.90046 -2.34315
H -4.73286 -2.70052 -2.06540
H -7.23588 -2.95622 -0.32450
H -6.57947 -4.19947 -1.38025
H -5.94210 -4.58007 1.01822
H -4.53189 -4.35001 -0.00507
H -5.81847 -2.23650 1.79524
H -4.28007 -3.04280 2.07695
66
pm6-M2
C 3.48782 4.07914 0.63831
C 3.89134 2.88315 0.05915
C 2.90842 1.93727 -0.23351
C 1.54271 2.18460 0.05071
C 1.16411 3.39403 0.63681
C 2.14029 4.33622 0.92752
N 3.03296 0.68261 -0.81315
C 1.77973 0.11913 -0.91395
C 0.81757 1.01447 -0.38184
C 1.39901 -1.10944 -1.46065
C 0.05306 -1.42983 -1.45791
C -0.91653 -0.56997 -0.90830
C -0.52907 0.66114 -0.37899
C -2.33948 -1.02363 -0.96732
N -3.17872 -0.48214 -0.04102
O -2.71696 -1.85554 -1.78604
C -4.61399 -0.66585 -0.08063
C -5.24415 0.72010 -0.11296
C -5.15368 -1.54812 1.09549
C -4.81135 -0.92588 2.45404
C -6.67312 -1.71178 0.96808
C -4.49490 -2.92881 0.98790
O -6.34519 0.75541 -0.86406
O -4.79523 1.67979 0.47058
C -7.02246 2.02188 -0.93178
C 4.26321 0.05826 -1.26915
C 4.82045 -1.01408 -0.32596
C 5.19291 -0.45537 1.05059
C 5.77082 -1.54004 1.96105
C 6.97162 -2.23154 1.31481
C 6.61508 -2.78632 -0.06477
C 6.02821 -1.70321 -0.97156
H 4.23570 4.82922 0.87353
H 4.93862 2.70026 -0.15061
H 0.12146 3.59558 0.86002
H 1.86145 5.28070 1.38187
H 2.12588 -1.79543 -1.87972
H -0.28521 -2.36623 -1.88588
H -1.26196 1.36651 -0.00006
H -2.83295 0.22311 0.59062
H -4.84363 -1.17761 -1.01708
H -5.18332 -1.56767 3.25822
H -3.72991 -0.82825 2.58721
H -5.26115 0.06267 2.57841
H -7.03171 -2.40854 1.73145
H -7.20493 -0.76701 1.10607
H -6.94981 -2.11433 -0.01104
H -3.40840 -2.85902 1.07913
H -4.86056 -3.57670 1.79039
H -4.72491 -3.40572 0.03115
H -6.36061 2.78199 -1.34879
H -7.87745 1.85957 -1.58364
H -7.35084 2.33121 0.06157
H 4.07481 -0.37836 -2.25596
H 5.00408 0.84867 -1.41704
H 4.03833 -1.77286 -0.18062
H 5.93893 0.34161 0.92061
H 4.31854 0.00608 1.52055
H 6.05423 -1.10601 2.92572
H 4.99284 -2.28601 2.17164
H 7.79173 -1.50803 1.21187
H 7.34286 -3.03378 1.96144
H 7.49752 -3.23111 -0.53690
H 5.88141 -3.59539 0.04952
H 6.79977 -0.94853 -1.18102
H 5.74275 -2.13421 -1.93838
66
pm6-M3
C 3.50661 4.01229 0.83188
C 3.90257 2.84181 0.19730
C 2.91228 1.91604 -0.13283
C 1.54864 2.15623 0.16242
C 1.17790 3.33953 0.80397
C 2.16086 4.26141 1.13604
N 3.03384 0.69224 -0.77281
C 1.77820 0.13760 -0.90304
C 0.81952 1.01183 -0.32858
C 1.38798 -1.06388 -1.50064
C 0.04065 -1.37665 -1.51052
C -0.92503 -0.53657 -0.92450
C -0.52921 0.66666 -0.34095
C -2.35010 -0.97962 -1.00404
N -3.19077 -0.46546 -0.06333
O -2.72899 -1.78111 -1.85208
C -4.62688 -0.63879 -0.11691
C -5.24850 0.75137 -0.11056
C -5.17885 -1.55345 1.02828
C -4.84057 -0.97617 2.40750
C -6.69853 -1.70325 0.88749
C -4.52789 -2.93415 0.88126
O -6.34329 0.81705 -0.86877
O -4.79843 1.68961 0.50607
C -7.01236 2.08920 -0.90191
C 4.28578 0.12538 -1.24371
C 5.14303 -0.52153 -0.14953
C 6.50228 -0.93470 -0.72560
C 7.39375 -1.58520 0.33362
C 6.69743 -2.77079 1.00249
C 5.34030 -2.36367 1.57692
C 4.45263 -1.71840 0.51127
H 4.25800 4.74819 1.09857
H 4.94731 2.66270 -0.02951
H 0.13685 3.53808 1.03717
H 1.88790 5.18527 1.63428
H 2.10892 -1.73887 -1.94618
H -0.30192 -2.29269 -1.97731
H -1.25636 1.35937 0.07086
H -2.84492 0.21729 0.59248
H -4.85383 -1.12022 -1.06994
H -5.22044 -1.64086 3.18907
H -3.75935 -0.88899 2.54946
H -5.28571 0.01047 2.56054
H -7.06581 -2.42185 1.62609
H -7.22502 -0.75993 1.05314
H -6.97235 -2.07219 -0.10558
H -3.44148 -2.87385 0.97982
H -4.90152 -3.60442 1.66137
H -4.75606 -3.37951 -0.09095
H -6.34185 2.85845 -1.28710
H -7.86160 1.95356 -1.56724
H -7.34911 2.36787 0.09769
H 4.05093 -0.60906 -2.01907
H 4.85252 0.92385 -1.73488
H 5.32244 0.23502 0.62757
H 6.34029 -1.64497 -1.54896
H 7.00650 -0.06349 -1.16003
H 8.33948 -1.90315 -0.11797
H 7.64823 -0.83798 1.09710
H 6.55056 -3.56805 0.26132
H 7.33403 -3.18965 1.78916
H 4.83291 -3.23249 2.00927
H 5.49311 -1.65129 2.39869
H 4.21760 -2.46429 -0.26142
H 3.49885 -1.40934 0.95079
66
pm6-M41
C -3.53793 3.89209 -0.60494
C -3.89931 2.58691 -0.91243
C -2.92651 1.59382 -0.79238
C -1.60699 1.90919 -0.38743
C -1.27036 3.22797 -0.07636
C -2.24022 4.21409 -0.18402
N -3.01587 0.23031 -1.04562
C -1.76845 -0.32987 -0.85202
C -0.86203 0.67538 -0.42944
C -1.34196 -1.64959 -1.02507
C -0.01702 -1.94616 -0.76042
C 0.89898 -0.96364 -0.34048
C 0.46656 0.35075 -0.16893
C 2.29789 -1.41215 -0.06536
N 3.25831 -0.44670 -0.11496
O 2.56127 -2.58694 0.16971
C 4.63019 -0.69042 0.27536
C 5.02154 0.18720 1.45881
C 5.64241 -0.57505 -0.91516
C 5.66689 0.84776 -1.48600
C 7.04708 -0.96728 -0.44077
C 5.19871 -1.55518 -2.00832
O 4.37896 1.36475 1.45204
O 5.82595 -0.12519 2.30119
C 4.68981 2.25708 2.53397
C -4.18894 -0.46262 -1.56350
C -5.14837 -1.06486 -0.52299
C -5.81385 -0.01068 0.36895
C -6.81079 -0.63668 1.34619
C -6.15724 -1.72825 2.19251
C -5.50288 -2.78770 1.30703
C -4.50468 -2.16484 0.32891
H -4.28055 4.67834 -0.69173
H -4.90829 2.35872 -1.23249
H -0.26288 3.47916 0.23914
H -1.99431 5.24334 0.05347
H -2.01965 -2.42901 -1.34990
H 0.34574 -2.96136 -0.86952
H 1.13768 1.12360 0.19238
H 2.99044 0.50209 -0.31682
H 4.66540 -1.72049 0.63638
H 6.35385 0.89390 -2.33618
H 4.68111 1.15514 -1.84850
H 6.00291 1.58094 -0.74748
H 7.73558 -0.95818 -1.29106
H 7.43605 -0.28158 0.31483
H 7.05300 -1.97476 -0.01373
H 4.20730 -1.30441 -2.39301
H 5.90434 -1.52029 -2.84378
H 5.16785 -2.58127 -1.63142
H 5.75008 2.51337 2.52283
H 4.08111 3.14273 2.36706
H 4.43864 1.79424 3.48920
H -4.73502 0.24659 -2.19296
H -3.83292 -1.25439 -2.22938
H -5.94198 -1.53584 -1.12351
H -5.04128 0.52497 0.93508
H -6.32432 0.73431 -0.25177
H -7.23939 0.14018 1.98821
H -7.64662 -1.07047 0.78066
H -5.39428 -1.27618 2.84029
H -6.89677 -2.19006 2.85540
H -4.99942 -3.54192 1.92093
H -6.28191 -3.31557 0.74042
H -3.66375 -1.74131 0.89267
H -4.08941 -2.94440 -0.31955
66
pm6-M44
C 3.63939 3.89376 0.56627
C 4.00350 2.70218 -0.04715
C 2.99697 1.77728 -0.32731
C 1.64434 2.05069 -0.01015
C 1.30497 3.25264 0.61405
C 2.30639 4.16880 0.90123
N 3.08282 0.53253 -0.93918
C 1.80669 0.01809 -1.05750
C 0.88004 0.91957 -0.47487
C 1.37022 -1.16147 -1.66706
C 0.01364 -1.43189 -1.66650
C -0.91853 -0.57017 -1.05913
C -0.47881 0.61600 -0.47268
C -2.35781 -0.96694 -1.12550
N -3.16998 -0.43254 -0.17086
O -2.77262 -1.74440 -1.97892
C -4.60668 -0.60425 -0.18453
C -5.31244 0.74369 -0.27414
C -5.13832 -1.47906 1.00231
C -4.88017 -0.79895 2.35206
C -6.64101 -1.73003 0.82696
C -4.40482 -2.82549 0.96201
O -4.61639 1.72203 0.32419
O -6.38107 0.91880 -0.80486
C -5.21308 3.02833 0.29187
C 4.29177 -0.05685 -1.50049
C 5.09479 -0.98779 -0.57655
C 4.31629 -2.23936 -0.15550
C 5.16396 -3.17570 0.70808
C 5.73096 -2.45330 1.92931
C 6.51806 -1.21154 1.51291
C 5.67208 -0.27270 0.65030
H 4.40764 4.62603 0.79167
H 5.03979 2.50764 -0.29407
H 0.27187 3.47082 0.86462
H 2.05847 5.10791 1.38378
H 2.06295 -1.85169 -2.13197
H -0.36223 -2.33003 -2.14218
H -1.18088 1.32880 -0.05175
H -2.77798 0.18973 0.51616
H -4.84106 -1.13344 -1.11071
H -5.22056 -1.44770 3.16453
H -3.81432 -0.60929 2.51286
H -5.41322 0.15177 2.44183
H -6.99500 -2.39771 1.61818
H -7.22386 -0.80809 0.87794
H -6.85177 -2.20711 -0.13507
H -3.32997 -2.70072 1.11310
H -4.78443 -3.47602 1.75562
H -4.55685 -3.33099 0.00438
H -6.18262 3.01572 0.79167
H -4.51999 3.67989 0.81897
H -5.34116 3.35973 -0.73944
H 4.00058 -0.60383 -2.40220
H 4.93168 0.76583 -1.83329
H 5.94395 -1.32010 -1.19350
H 3.42418 -1.93798 0.40827
H 3.96237 -2.77543 -1.04322
H 4.56542 -4.03895 1.01768
H 5.99306 -3.57075 0.10554
H 4.90537 -2.15451 2.58909
H 6.36671 -3.12975 2.51054
H 6.88392 -0.67645 2.39565
H 7.40605 -1.52109 0.94525
H 4.84971 0.13026 1.25508
H 6.27860 0.58278 0.33208
66
pm6-M47
C -5.18869 3.24478 0.02516
C -4.95113 1.97327 -0.48063
C -3.63319 1.51559 -0.50311
C -2.57012 2.32675 -0.03884
C -2.83534 3.59827 0.47274
C -4.14647 4.05067 0.50390
N -3.12254 0.30673 -0.96244
C -1.74767 0.34183 -0.83874
C -1.35853 1.57441 -0.25327
C -0.79023 -0.59896 -1.22430
C 0.54391 -0.31161 -0.98342
C 0.94622 0.89179 -0.37284
C -0.01339 1.84143 -0.02757
C 2.37517 1.23537 -0.10163

N 3.22999 0.18120 0.02007
O 2.74494 2.39772 0.02864
C 4.65999 0.34816 0.16416
C 5.40334 -0.34023 -0.97433
C 5.19578 -0.08189 1.57307
C 4.97370 -1.57917 1.81763
C 6.69015 0.24530 1.68050
C 4.43584 0.72474 2.63349
O 4.75681 -1.42861 -1.41839
O 6.45979 0.03217 -1.42099
C 5.39267 -2.14391 -2.48954
C -3.89524 -0.75283 -1.59739
C -4.40594 -1.87565 -0.67866
C -5.37430 -1.38173 0.40203
C -5.91135 -2.53199 1.25648
C -4.77833 -3.35046 1.87411
C -3.81842 -3.85454 0.79749
C -3.27767 -2.70461 -0.05444
H -6.20673 3.61930 0.05044
H -5.77153 1.36564 -0.84205
H -2.02640 4.22492 0.83362
H -4.36925 5.03688 0.89631
H -1.06712 -1.52794 -1.70655
H 1.28564 -1.03036 -1.31631
H 0.31373 2.77823 0.40886
H 2.87445 -0.75484 -0.08162
H 4.85470 1.41706 0.05318
H 5.31980 -1.84574 2.82063
H 3.91380 -1.84544 1.75863
H 5.52165 -2.19863 1.10215
H 7.04444 0.00965 2.68849
H 7.29134 -0.32542 0.96971
H 6.87583 1.30821 1.49853
H 3.36550 0.50633 2.61221
H 4.81253 0.47206 3.62919
H 4.56610 1.79974 2.48208
H 6.37142 -2.50857 -2.17458
H 4.73107 -2.97600 -2.71914
H 5.50990 -1.49620 -3.35929
H -4.74441 -0.27988 -2.09937
H -3.27329 -1.18533 -2.38720
H -4.97120 -2.54215 -1.34833
H -4.85760 -0.66141 1.04894
H -6.20965 -0.84562 -0.06192
H -6.56879 -2.13788 2.03861
H -6.53035 -3.18850 0.63001
H -4.22585 -2.72381 2.58697
H -5.18453 -4.19147 2.44620
H -2.98710 -4.40334 1.25265
H -4.34544 -4.56753 0.14904
H -2.65403 -2.05437 0.57220
H -2.62786 -3.10215 -0.84237
66
pm6-M4
C -4.87035 3.61995 -0.44653
C -4.73680 2.37281 0.15051
C -3.46771 1.79323 0.16419
C -2.34997 2.44991 -0.40414
C -2.51189 3.70147 -1.00063
C -3.77370 4.27942 -1.01924
N -3.06313 0.57950 0.69967
C -1.70680 0.43988 0.49540
C -1.21708 1.58103 -0.19265
C -0.85018 -0.60221 0.85651
C 0.49311 -0.48329 0.53808
C 1.00008 0.64338 -0.13861
C 0.13229 1.66811 -0.51288
C 2.43697 0.80064 -0.51971
N 3.33314 0.05278 0.18275
O 2.78677 1.58402 -1.39688
C 4.73105 -0.04075 -0.18158
C 5.04581 -1.51651 -0.38589
C 5.68385 0.64102 0.85751
C 5.51840 0.01874 2.24875
C 7.13997 0.50108 0.39728
C 5.32302 2.13017 0.92007
O 5.90893 -1.71371 -1.38291
O 4.56445 -2.40788 0.27513
C 6.28287 -3.07924 -1.63283
C -3.94001 -0.36077 1.37576
C -4.80346 -1.21406 0.43915
C -5.79129 -2.05260 1.25833
C -6.67704 -2.92175 0.36388
C -5.84256 -3.80528 -0.56399
C -4.85574 -2.97227 -1.38220
C -3.96956 -2.11038 -0.48123
H -5.84717 4.09171 -0.47017
H -5.59473 1.87495 0.58748
H -1.66363 4.21553 -1.44053
H -3.91549 5.25122 -1.47942
H -1.21307 -1.49099 1.35845
H 1.14495 -1.31296 0.79140
H 0.53451 2.51870 -1.05105
H 3.00198 -0.61424 0.86159
H 4.84699 0.47761 -1.13515
H 6.18375 0.51931 2.95846
H 4.49593 0.13714 2.61929
H 5.76223 -1.04686 2.25378
H 7.79255 1.06684 1.06871
H 7.47869 -0.53815 0.40521
H 7.27769 0.89298 -0.61499
H 4.28883 2.27587 1.24080
H 5.97419 2.63873 1.63760
H 5.44734 2.61065 -0.05430
H 5.40221 -3.67196 -1.88353
H 6.97164 -3.04270 -2.47340
H 6.77095 -3.50656 -0.75567
H -3.31896 -1.00527 2.00407
H -4.58347 0.20811 2.05588
H -5.38642 -0.53121 -0.19498
H -5.22691 -2.69829 1.94626
H -6.41136 -1.39850 1.88260
H -7.34180 -3.53621 0.98031
H -7.32403 -2.27246 -0.24093
H -5.28517 -4.53565 0.03807
H -6.49623 -4.38177 -1.22725
H -4.23341 -3.62214 -2.00632
H -5.41284 -2.32126 -2.06912
H -3.33859 -2.76559 0.13626
H -3.29194 -1.50221 -1.08888
66
pm6-M5
C -4.96593 3.56383 0.13796
C -4.81316 2.26955 -0.34280
C -3.52181 1.74349 -0.38973
C -2.40138 2.49857 0.03209
C -2.58255 3.79597 0.51415
C -3.86626 4.32123 0.56538
N -3.09662 0.49928 -0.83111
C -1.72473 0.43547 -0.70851
C -1.24506 1.65967 -0.17298
C -0.84366 -0.59626 -1.03905
C 0.50891 -0.39736 -0.81357
C 1.00357 0.80415 -0.26955
C 0.11748 1.83614 0.03552
C 2.45507 1.06218 -0.02219
N 3.25193 -0.03680 0.09457
O 2.89351 2.20158 0.09938
C 4.69573 0.04597 0.15806
C 5.24714 -0.78832 -0.99029
C 5.27508 -0.38433 1.54777
C 4.86417 -1.81972 1.89592
C 6.80458 -0.27411 1.52936
C 4.71980 0.57560 2.60696
O 6.33742 -0.24256 -1.53012
O 4.75102 -1.82303 -1.37297
C 6.94212 -0.97530 -2.60914
C -3.97080 -0.53931 -1.34840
C -4.73143 -1.32548 -0.27400
C -3.79670 -2.07393 0.68097
C -4.57894 -2.87342 1.72419
C -5.56413 -3.83884 1.06504
C -6.49861 -3.10499 0.10256
C -5.71642 -2.29695 -0.93450
H -5.96030 3.99550 0.18438
H -5.67335 1.69573 -0.66756
H -1.73223 4.38529 0.84119
H -4.02316 5.32773 0.93767
H -1.19143 -1.52906 -1.46626
H 1.18913 -1.19038 -1.10644
H 0.51573 2.76378 0.43042
H 2.86268 -0.95282 -0.06247
H 4.96191 1.09122 -0.00906
H 5.27367 -2.09435 2.87256
H 3.77649 -1.91976 1.95754
H 5.23271 -2.54114 1.16197
H 7.19858 -0.47271 2.53039
H 7.26316 -0.99263 0.84499
H 7.12868 0.72797 1.23286
H 3.62856 0.53750 2.64536
H 5.10252 0.29906 3.59416
H 5.01695 1.60808 2.40290
H 6.23768 -1.08036 -3.43531
H 7.80344 -0.38575 -2.91389
H 7.25469 -1.96394 -2.26982
H -4.68113 -0.06961 -2.03756
H -3.36231 -1.22095 -1.94907
H -5.31379 -0.60700 0.32007
H -3.16827 -2.76148 0.09700
H -3.11879 -1.37057 1.17491
H -3.88594 -3.41942 2.37294
H -5.13008 -2.17864 2.37195
H -5.00334 -4.60377 0.51093
H -6.14552 -4.36922 1.82675
H -7.16256 -3.81561 -0.40104
H -7.14482 -2.42563 0.67421
H -5.15717 -2.98555 -1.58398
H -6.40768 -1.74766 -1.58445

In the following, the atomic coordinates for the optimized conformers of (S)-**3** in acetonitrile are given in XYZ format:

66
mmff-M10
C -3.45962 4.03305 0.74173
C -3.85791 2.84809 0.13642
C -2.86680 1.92749 -0.20678
C -1.49855 2.18960 0.05269
C -1.12499 3.38753 0.66609
C -2.10940 4.30422 1.00746
N -2.98548 0.68835 -0.81982
C -1.72719 0.14871 -0.96542
C -0.76561 1.04440 -0.43089
C -1.34067 -1.05913 -1.55373
C 0.00975 -1.35775 -1.59013
C 0.98006 -0.49623 -1.04259
C 0.58637 0.71456 -0.47110
C 2.40655 -0.92779 -1.14145
N 3.27703 -0.32761 -0.28108
O 2.76733 -1.77301 -1.95678
C 4.67383 -0.70643 -0.21051
C 5.00909 -1.28976 1.15682
C 5.64454 0.45122 -0.62622
C 5.50521 1.65524 0.31210
C 7.09234 -0.05415 -0.61149
C 5.28398 0.87293 -2.05626
O 4.28686 -0.73290 2.13570
O 5.83854 -2.14683 1.34399
C 4.53340 -1.21935 3.46600
C -4.21775 0.05705 -1.26511
C -4.73309 -1.05259 -0.34175
C -5.94586 -1.74059 -0.97914
C -6.49090 -2.86245 -0.09370
C -6.82111 -2.35891 1.31187
C -5.61570 -1.66765 1.94990
C -5.07978 -0.54418 1.06083
H -4.21369 4.76320 1.01647
H -4.90666 2.65427 -0.05496
H -0.08074 3.59940 0.87156
H -1.83531 5.23928 1.48368
H -2.06656 -1.74579 -1.97300
H 0.34955 -2.27991 -2.04663
H 1.31754 1.41826 -0.08750
H 2.90260 0.18790 0.49977
H 4.80820 -1.52113 -0.92502
H 6.16336 2.46283 -0.02175
H 4.48327 2.04571 0.31587
H 5.77958 1.40630 1.34090
H 7.75648 0.73084 -0.98504
H 7.42557 -0.32499 0.39278
H 7.21272 -0.93106 -1.25515
H 4.26054 1.25087 -2.11616
H 5.95774 1.66840 -2.38804
H 5.38149 0.03434 -2.75224
H 5.57042 -1.03566 3.74946
H 3.85720 -0.66276 4.11015
H 4.32214 -2.28787 3.52133
H -4.97401 0.83923 -1.37070
H -4.04619 -0.34673 -2.26850
H -3.93641 -1.80339 -0.24084
H -6.73432 -0.99257 -1.14394
H -5.67721 -2.13503 -1.96599
H -7.37793 -3.30673 -0.55773
H -5.74104 -3.66175 -0.02448
H -7.65533 -1.64674 1.25331
H -7.16168 -3.18811 1.94126
H -5.88172 -1.26979 2.93499
H -4.82029 -2.40655 2.11600
H -5.84208 0.24310 0.97522
H -4.20111 -0.08346 1.52404
66
mmff-M11
C 3.37831 4.00960 0.72549
C 3.76772 2.84654 0.07319
C 2.80351 1.85550 -0.11633
C 1.47146 2.02524 0.33324
C 1.10645 3.20355 0.98809
C 2.06431 4.18944 1.18195
N 2.92395 0.62277 -0.73919
C 1.69847 -0.00625 -0.70836
C 0.75897 0.83009 -0.04992
C 1.32173 -1.25749 -1.20495
C 0.00748 -1.65368 -1.03554
C -0.94451 -0.83557 -0.39675
C -0.55910 0.40853 0.10395
C -2.32921 -1.37944 -0.26565
N -3.31316 -0.46844 -0.02069
O -2.55981 -2.58182 -0.36976
C -4.71027 -0.84608 0.04890
C -5.51178 -0.16953 -1.05638
C -5.33774 -0.62265 1.46748
C -5.32487 0.86110 1.85195
C -6.77665 -1.15239 1.48685
C -4.50497 -1.41978 2.47932
O -5.01966 1.03289 -1.37552
O -6.48795 -0.65113 -1.57873
C -5.71279 1.75175 -2.40984
C 4.15025 0.10698 -1.32646
C 5.15874 -0.44232 -0.31166
C 6.46425 -0.81559 -1.02413
C 7.50462 -1.36988 -0.04927
C 6.95523 -2.55569 0.74439
C 5.65183 -2.18963 1.45481
C 4.61473 -1.64128 0.47302
H 4.10977 4.79485 0.88523
H 4.78722 2.72197 -0.27281
H 0.08954 3.34829 1.33791
H 1.79638 5.10966 1.68959
H 2.02874 -1.90939 -1.70369
H -0.31761 -2.62135 -1.39883
H -1.25979 1.04248 0.63734
H -3.10858 0.50850 -0.15931
H -4.75021 -1.91670 -0.16256
H -5.72600 0.98537 2.86213
H -4.30940 1.26797 1.85012
H -5.93595 1.46492 1.17528
H -7.18376 -1.06655 2.49866
H -7.43115 -0.59542 0.81300
H -6.81382 -2.20731 1.19776
H -3.47201 -1.06553 2.51478
H -4.93450 -1.30962 3.47939
H -4.49366 -2.48514 2.23066
H -6.74268 1.94758 -2.10895
H -5.16730 2.68410 -2.53288
H -5.70471 1.17812 -3.33739
H 3.87645 -0.67179 -2.04312
H 4.61090 0.91441 -1.90544
H 5.38448 0.35659 0.40721
H 6.24826 -1.57139 -1.79257
H 6.86657 0.05855 -1.54910
H 8.40861 -1.66170 -0.59440
H 7.80285 -0.57547 0.64784
H 6.76885 -3.39354 0.05900
H 7.69900 -2.90407 1.46906
H 5.24542 -3.06107 1.97907
H 5.85808 -1.43158 2.22230
H 4.33542 -2.43341 -0.23600
H 3.70156 -1.36017 1.00781
66
mmff-M12
C 3.34874 4.09189 0.34722
C 3.74507 2.87546 -0.19351
C 2.79034 1.86164 -0.28436
C 1.45932 2.06364 0.15810
C 1.08726 3.29577 0.70057
C 2.03595 4.30442 0.79296
N 2.91449 0.57281 -0.78321
C 1.69470 -0.05723 -0.67869
C 0.75324 0.83149 -0.09870
C 1.32905 -1.35335 -1.05366
C 0.01905 -1.74314 -0.83926
C -0.93597 -0.87434 -0.27664
C -0.56048 0.41568 0.10097
C -2.31683 -1.41168 -0.08804
N -3.30730 -0.48521 0.04845
O -2.53805 -2.61970 -0.05571
C -4.70271 -0.85929 0.15757
C -5.49878 -0.34041 -1.03396
C -5.33996 -0.45254 1.52997
C -5.31952 1.06824 1.72071
C -6.78295 -0.96603 1.60569
C -4.52041 -1.11857 2.64240
O -5.01627 0.81735 -1.49923
O -6.46227 -0.89828 -1.50102
C -5.70308 1.38852 -2.62570
C 4.11426 -0.02595 -1.34647
C 4.85100 -0.98636 -0.40587
C 5.36188 -0.29904 0.86398
C 6.11978 -1.27477 1.76617
C 7.26826 -1.95178 1.01764
C 6.77156 -2.63528 -0.25677
C 6.00616 -1.66155 -1.15430
H 4.07498 4.89399 0.42736
H 4.76545 2.72889 -0.52728
H 0.07141 3.46258 1.04362
H 1.76267 5.26654 1.21237
H 2.04123 -2.04014 -1.49560
H -0.30027 -2.74348 -1.10669
H -1.26687 1.09054 0.57307
H -3.10750 0.47047 -0.20126
H -4.73540 -1.94857 0.08868
H -5.73074 1.32387 2.70169
H -4.30091 1.46509 1.67884
H -5.91828 1.58349 0.96457
H -7.19502 -0.75136 2.59612
H -7.42953 -0.49268 0.86359

H -6.82698 -2.04849 1.45045
H -3.48342 -0.77439 2.63653
H -4.95232 -0.87309 3.61708
H -4.52121 -2.20757 2.53640
H -6.73868 1.60970 -2.36465
H -5.16539 2.30344 -2.86189
H -5.67715 0.70012 -3.47122
H 3.82853 -0.55313 -2.26287
H 4.78067 0.78547 -1.65065
H 4.14301 -1.77091 -0.10311
H 6.03294 0.52311 0.57774
H 4.52773 0.15098 1.41220
H 6.49770 -0.74947 2.64974
H 5.42415 -2.04158 2.13268
H 8.02020 -1.19631 0.75236
H 7.76992 -2.67780 1.66644
H 7.61166 -3.07013 -0.80871
H 6.11053 -3.46920 0.01440
H 6.69529 -0.88697 -1.51975
H 5.62451 -2.18385 -2.03925
66
mmff-M13
C 4.90540 3.49872 0.77327
C 4.75653 2.29581 0.09438
C 3.46207 1.80138 -0.07190
C 2.33529 2.49921 0.42611
C 2.51263 3.70503 1.10770
C 3.79928 4.19747 1.27864
N 3.04058 0.64609 -0.71269
C 1.66569 0.58448 -0.64645
C 1.17869 1.71911 0.05582
C 0.78709 -0.37144 -1.16204
C -0.57084 -0.18763 -0.95795
C -1.07336 0.92496 -0.25372
C -0.18909 1.88332 0.24020
C -2.53030 1.16118 -0.02574
N -3.34630 0.07885 -0.16879
O -2.96219 2.27276 0.27073
C -4.76777 0.14623 0.10297
C -5.14119 -0.75072 1.27691
C -5.64741 -0.12555 -1.16495
C -5.41758 -1.54092 -1.70648
C -7.12919 0.06373 -0.81856
C -5.25542 0.90102 -2.23513
O -4.36812 -1.84025 1.34762
O -6.03909 -0.51751 2.04966
C -4.64720 -2.75210 2.42355
C 3.92257 -0.30303 -1.37256
C 4.65372 -1.26116 -0.42536
C 5.67126 -2.09300 -1.21447
C 6.43186 -3.06547 -0.31162
C 5.47740 -3.96605 0.47314
C 4.45921 -3.14054 1.26024
C 3.69896 -2.17398 0.34989
H 5.90176 3.90395 0.91569
H 5.62134 1.76702 -0.28910
H 1.65809 4.25000 1.49517
H 3.95367 5.13253 1.80604
H 1.14171 -1.23302 -1.71460
H -1.24998 -0.91574 -1.38791
H -0.59052 2.74480 0.76157
H -2.92866 -0.83757 -0.20390
H -4.96953 1.16809 0.43051
H -6.00981 -1.69076 -2.61401
H -4.36844 -1.70524 -1.96916
H -5.71350 -2.30817 -0.98576
H -7.73272 -0.04605 -1.72440
H -7.48025 -0.67076 -0.09045
H -7.31587 1.06121 -0.40873
H -4.20723 0.79623 -2.52516
H -5.86947 0.75608 -3.12886
H -5.41212 1.92351 -1.87871
H -5.66415 -3.13710 2.33873
H -3.92420 -3.55722 2.31847
H -4.52411 -2.25028 3.38401
H 3.32614 -0.86988 -2.09234
H 4.65293 0.26787 -1.95579
H 5.20877 -0.65527 0.30472
H 5.14108 -2.65989 -1.99292
H 6.37408 -1.43084 -1.73328
H 7.12085 -3.67024 -0.91083
H 7.05006 -2.49315 0.39283
H 4.94418 -4.62349 -0.22684
H 6.04097 -4.61927 1.14797
H 3.75351 -3.79858 1.77835
H 4.98119 -2.56785 2.03855
H 3.09876 -2.75139 -0.36759
H 2.99698 -1.57433 0.93870
66
mmff-M14
C 4.77616 3.67991 -0.23490
C 4.64045 2.40922 0.31003
C 3.38183 1.80919 0.24963
C 2.27677 2.46881 -0.34086
C 2.44063 3.74489 -0.88377
C 3.69211 4.34316 -0.82844
N 2.97739 0.57033 0.72351
C 1.63434 0.41614 0.45775
C 1.15210 1.57374 -0.20914
C 0.78244 -0.65279 0.74639
C -0.54753 -0.54640 0.37361
C -1.04699 0.59541 -0.28466
C -0.18425 1.64929 -0.58373
C -2.47008 0.73742 -0.71552
N -3.36590 -0.10311 -0.12507
O -2.80653 1.56591 -1.55843
C -4.78873 -0.01844 -0.38436
C -5.54951 0.36491 0.87925
C -5.36381 -1.30671 -1.06602
C -5.18995 -2.53484 -0.16537
C -6.84940 -1.10498 -1.38847
C -4.59879 -1.52307 -2.37782
O -4.96994 -0.11641 1.98479
O -6.56543 1.01718 0.88794
C -5.61598 0.19507 3.23085
C 3.84367 -0.38319 1.39777
C 4.75579 -1.18595 0.46289
C 3.97260 -2.05124 -0.52907
C 4.90651 -2.86519 -1.42652
C 5.86749 -3.72183 -0.60187
C 6.65091 -2.86927 0.39684
C 5.71734 -2.04864 1.28837
H 5.74469 4.16772 -0.20033
H 5.48818 1.90939 0.76412
H 1.60229 4.26193 -1.33905
H 3.83570 5.33382 -1.24589
H 1.13995 -1.55123 1.23456
H -1.19714 -1.39172 0.57271
H -0.57764 2.51466 -1.10464
H -3.08050 -0.61133 0.69684
H -4.92577 0.81177 -1.08018
H -5.56213 -3.42615 -0.67900
H -4.13730 -2.71100 0.07508
H -5.74288 -2.43622 0.77283
H -7.22485 -1.97360 -1.93742
H -7.45597 -0.98994 -0.48748
H -7.00266 -0.21999 -2.01383
H -3.53300 -1.68277 -2.19769
H -4.99027 -2.40518 -2.89303
H -4.70881 -0.66390 -3.04625
H -6.62402 -0.22098 3.24858
H -5.00048 -0.26309 4.00106
H -5.66306 1.27564 3.37075
H 4.45080 0.16889 2.12301
H 3.20928 -1.06063 1.97559
H 5.35822 -0.47131 -0.11559
H 3.32377 -2.73781 0.03293
H 3.31478 -1.42473 -1.14033
H 4.31899 -3.49483 -2.10314
H 5.48489 -2.18010 -2.06081
H 5.29342 -4.48200 -0.05496
H 6.55561 -4.26312 -1.26004
H 7.29632 -3.50233 1.01505
H 7.31459 -2.18797 -0.15206
H 5.13135 -2.72877 1.92272
H 6.30173 -1.41479 1.96534
66
mmff-M15
C 4.93018 3.52121 0.64387
C 4.77519 2.30364 -0.00638
C 3.47955 1.80796 -0.15878
C 2.35756 2.52074 0.33194
C 2.54107 3.74146 0.98495
C 3.82890 4.23570 1.13766
N 3.04667 0.63771 -0.76653
C 1.67357 0.58283 -0.68337
C 1.19522 1.73417 -0.00509
C 0.79240 -0.38404 -1.17349
C -0.56384 -0.19593 -0.96067
C -1.05877 0.93082 -0.27364
C -0.17078 1.90169 0.18932
C -2.51325 1.16881 -0.02990
N -3.33004 0.08416 -0.14783
O -2.94215 2.28367 0.25821
C -4.74706 0.15251 0.14668
C -5.09997 -0.74071 1.32975
C -5.64733 -0.12446 -1.10555
C -5.41988 -1.53899 -1.65029
C -7.12378 0.05773 -0.73355
C -5.27893 0.90327 -2.18297
O -4.32704 -1.83113 1.38895
O -5.98310 -0.50440 2.11833
C -4.58703 -2.74005 2.47214
C 3.88174 -0.36453 -1.40923
C 4.11780 -1.62784 -0.57373
C 4.85006 -1.34204 0.74081
C 5.09862 -2.62434 1.53713
C 5.85941 -3.65924 0.70773
C 5.14162 -3.94726 -0.61130
C 4.88533 -2.66391 -1.40305
H 5.92877 3.92536 0.77323
H 5.63930 1.76386 -0.37523
H 1.68912 4.29520 1.36566
H 3.98827 5.18238 1.64229
H 1.14548 -1.25418 -1.71403
H -1.24741 -0.93203 -1.36940
H -0.56880 2.77421 0.69473
H -2.91228 -0.83240 -0.17722
H -4.94427 1.17545 0.47367
H -6.02824 -1.69272 -2.54643
H -4.37491 -1.69753 -1.93248
H -5.69802 -2.30728 -0.92363
H -7.74223 -0.05361 -1.62907
H -7.45907 -0.67940 -0.00066
H -7.30801 1.05375 -0.31909
H -4.23476 0.80472 -2.48931
H -5.90592 0.75311 -3.06680
H -5.43662 1.92521 -1.82543
H -5.60522 -3.12545 2.40609
H -3.86584 -3.54536 2.35661
H -4.44731 -2.23557 3.42892
H 3.41232 -0.63371 -2.36140
H 4.83703 0.10578 -1.65668
H 3.13599 -2.05624 -0.32661
H 5.81422 -0.86542 0.51418
H 4.27961 -0.62826 1.34410
H 5.64851 -2.39276 2.45552
H 4.13444 -3.04788 1.84895
H 6.86764 -3.27915 0.49435
H 5.98864 -4.58472 1.27914
H 5.72401 -4.64966 -1.21708
H 4.18167 -4.43737 -0.40085
H 5.84689 -2.23072 -1.71301
H 4.33339 -2.89109 -2.32249
66
mmff-M16
C 4.75579 3.71144 -0.03821
C 4.62398 2.41543 0.44380
C 3.37174 1.80784 0.34214
C 2.26932 2.48805 -0.23216
C 2.42927 3.78973 -0.71208
C 3.67413 4.39515 -0.61250
N 2.96741 0.54335 0.74678
C 1.63035 0.39611 0.45323
C 1.14888 1.58083 -0.16248
C 0.78452 -0.69105 0.68614
C -0.54311 -0.57518 0.30660
C -1.04243 0.59329 -0.30288
C -0.18441 1.66562 -0.54613
C -2.46413 0.74646 -0.73508
N -3.36120 -0.10658 -0.16464
O -2.79787 1.59450 -1.55918
C -4.78398 -0.01389 -0.42168
C -5.54329 0.34951 0.84883
C -5.36231 -1.28886 -1.12492
C -5.18945 -2.53283 -0.24603
C -6.84800 -1.07898 -1.44160
C -4.59941 -1.48347 -2.44134
O -4.96388 -0.15149 1.94565
O -6.55798 1.00341 0.86913
C -5.60844 0.14001 3.19731
C 3.79073 -0.46203 1.39980
C 4.26596 -1.59238 0.47974
C 4.97611 -2.67183 1.30501
C 5.46533 -3.82570 0.42818
C 6.35860 -3.32782 -0.70841
C 5.65565 -2.24871 -1.53266
C 5.17316 -1.09607 -0.65008
H 5.72053 4.20310 0.03101
H 5.47309 1.90212 0.87928
H 1.59198 4.32003 -1.15389
H 3.81467 5.40573 -0.98024
H 1.14495 -1.60658 1.13967
H -1.19128 -1.43102 0.46032
H -0.57964 2.55244 -1.02813
H -3.07682 -0.63072 0.64770
H -4.91935 0.82864 -1.10287
H -5.56453 -3.41428 -0.77436
H -4.13678 -2.71548 -0.01062
H -5.74041 -2.44932 0.69478
H -7.22541 -1.93670 -2.00613
H -7.45316 -0.98001 -0.53777
H -7.00087 -0.18224 -2.05007
H -3.53361 -1.64787 -2.26555
H -4.99294 -2.35578 -2.97142
H -4.70906 -0.61255 -3.09449
H -6.61715 -0.27462 3.20886
H -4.99308 -0.33207 3.95919
H -5.65361 1.21815 3.35543
H 4.65027 0.04715 1.84363
H 3.21383 -0.88009 2.23155
H 3.37734 -2.05016 0.02229
H 5.83450 -2.21904 1.82126
H 4.30481 -3.04881 2.08529
H 6.00067 -4.55902 1.04070
H 4.59720 -4.34737 0.00369
H 7.28326 -2.91262 -0.28520
H 6.65645 -4.16364 -1.35057
H 6.32452 -1.86769 -2.31168
H 4.79415 -2.69176 -2.05012
H 6.04400 -0.59005 -0.20986
H 4.64843 -0.35073 -1.25673
66
mmff-M17
C -3.88046 3.70568 1.14355
C -4.19076 2.60460 0.35570
C -3.14210 1.78212 -0.05898
C -1.80234 2.06973 0.29861
C -1.51677 3.17787 1.09933
C -2.55997 3.99056 1.51984
N -3.17146 0.64224 -0.85333
C -1.87366 0.21496 -1.04712
C -0.98704 1.06122 -0.33234
C -1.38430 -0.83553 -1.82929
C -0.01603 -1.03505 -1.86858
C 0.87775 -0.23070 -1.13618
C 0.38510 0.82884 -0.37406
C 2.33271 -0.54564 -1.25366
N 3.13277 -0.05936 -0.26260
O 2.77630 -1.19844 -2.19489
C 4.54420 -0.37742 -0.18386
C 4.77953 -1.07587 1.14846
C 5.46140 0.87665 -0.38263
C 5.17399 1.94297 0.68011
C 6.93619 0.46203 -0.31595
C 5.17094 1.45117 -1.77468
O 5.72101 -2.01390 1.06507
O 4.17645 -0.81232 2.16405
C 6.03493 -2.70817 2.28600
C -4.35737 0.07936 -1.48909
C -5.09852 -1.01791 -0.70728
C -5.68710 -0.52546 0.61959
C -6.47403 -1.62409 1.33724
C -5.62349 -2.87529 1.55197
C -5.04420 -3.37582 0.22950
C -4.25550 -2.27942 -0.48955
H -4.68172 4.35772 1.47530
H -5.21769 2.40075 0.07907
H -0.49396 3.40390 1.38264
H -2.35492 4.85625 2.14018
H -2.04530 -1.48084 -2.39398
H 0.39770 -1.83327 -2.47345
H 1.05496 1.49756 0.15623
H 2.70253 0.24630 0.59785
H 4.75902 -1.08395 -0.98781
H 5.80308 2.82023 0.50277
H 4.13153 2.27193 0.64348
H 5.38265 1.58020 1.69014
H 7.56846 1.32208 -0.55522
H 7.22184 0.10673 0.67718
H 7.16017 -0.33065 -1.03632
H 4.12679 1.75935 -1.86869
H 5.79910 2.32936 -1.95134
H 5.38455 0.71813 -2.55818
H 5.14923 -3.21691 2.66814
H 6.80482 -3.42838 2.02069
H 6.40750 -2.00821 3.03496
H -5.04253 0.90747 -1.69201
H -4.05118 -0.31343 -2.46301
H -5.94158 -1.29422 -1.35869
H -4.87387 -0.18059 1.27120
H -6.33732 0.33808 0.44090
H -6.84920 -1.24743 2.29480
H -7.35598 -1.88618 0.73735
H -4.80148 -2.64036 2.24155
H -6.21752 -3.66206 2.02933
H -4.40127 -4.24625 0.39834
H -5.86411 -3.71385 -0.41846
H -3.36894 -2.02484 0.10558
H -3.89319 -2.65586 -1.45259
66
mmff-M18
C 3.68669 3.93132 0.38607
C 4.01110 2.74342 -0.25655
C 3.01950 1.76698 -0.36364
C 1.71836 1.98745 0.15014
C 1.41854 3.18768 0.79840
C 2.40745 4.15404 0.91556
N 3.07192 0.51294 -0.96035
C 1.81907 -0.05963 -0.87765
C 0.94560 0.81609 -0.18258
C 1.35883 -1.28354 -1.37268
C 0.03500 -1.61848 -1.15195
C -0.84744 -0.76542 -0.46191
C -0.38221 0.45478 0.02857
C -2.24987 -1.24361 -0.27454
N -3.17215 -0.29314 0.04816
O -2.54631 -2.42945 -0.39729
C -4.58604 -0.59267 0.15097
C -5.31214 0.30227 -0.84392
C -5.13364 -0.44700 1.61150
C -4.93968 0.98159 2.13184
C -6.62127 -0.81683 1.65184
C -4.35649 -1.42432 2.50225
O -6.37727 -0.29524 -1.37430
O -4.95817 1.42529 -1.12370
C -7.14731 0.48790 -2.30448
C 4.21781 -0.04283 -1.67147
C 5.18610 -0.90624 -0.84577
C 4.54038 -2.18298 -0.29557
C 5.54774 -3.05191 0.46061
C 6.23867 -2.26918 1.57630
C 6.89307 -1.00121 1.02958
C 5.88705 -0.12983 0.27462
H 4.44396 4.70271 0.47949
H 5.00579 2.59110 -0.65660
H 0.42538 3.36480 1.19809
H 2.19065 5.09188 1.41528
H 2.00953 -1.96102 -1.91103
H -0.35228 -2.56354 -1.51398
H -1.02953 1.11837 0.59195
H -2.93983 0.67798 -0.09943
H -4.71817 -1.63170 -0.15662
H -5.29765 1.05098 3.16327
H -3.88380 1.26699 2.12915
H -5.49266 1.71271 1.53594
H -6.97114 -0.81036 2.68830
H -7.23846 -0.11245 1.08895
H -6.79400 -1.81793 1.24498
H -3.28789 -1.19611 2.50671
H -4.72027 -1.35534 3.53177
H -4.48536 -2.45700 2.16509

H -6.52854 0.77943 -3.15386
H -7.95841 -0.15996 -2.62731
H -7.54129 1.37915 -1.81454
H 3.82930 -0.63178 -2.50734
H 4.76457 0.79529 -2.11310
H 5.95913 -1.21308 -1.56671
H 3.71984 -1.91167 0.38124
H 4.09758 -2.75968 -1.11522
H 5.04436 -3.93524 0.86784
H 6.30538 -3.42093 -0.24380
H 5.49717 -1.99422 2.33852
H 6.98393 -2.89698 2.07649
H 7.34805 -0.42453 1.84197
H 7.70912 -1.28081 0.34966
H 5.13519 0.24607 0.98048
H 6.39676 0.74628 -0.14127
66
mmff-M1
C -3.64109 3.85906 1.17404
C -4.00173 2.72930 0.45044
C -2.98070 1.87710 0.02736
C -1.62213 2.15082 0.31798
C -1.28675 3.29156 1.05058
C -2.30031 4.13974 1.47536
N -3.06517 0.70641 -0.71051
C -1.79156 0.21949 -0.90870
C -0.85672 1.08387 -0.28073
C -1.36461 -0.91121 -1.61147
C -0.00575 -1.16243 -1.67202
C 0.93780 -0.33054 -1.03861
C 0.50496 0.80216 -0.34829
C 2.37699 -0.70442 -1.17673
N 3.23228 -0.14677 -0.27341
O 2.76227 -1.46462 -2.06150
C 4.63566 -0.50253 -0.21741
C 4.91053 -1.04424 1.17865
C 5.57999 0.68591 -0.60516
C 5.38101 1.87682 0.33890
C 7.04281 0.22781 -0.56209
C 5.23582 1.10792 -2.03902
O 5.82932 -2.00787 1.17249
O 4.35360 -0.64809 2.17747
C 6.17784 -2.56091 2.45466
C -4.30348 0.12555 -1.20396
C -5.11353 -0.63593 -0.14868
C -4.36056 -1.84317 0.41841
C -5.20406 -2.60171 1.44473
C -6.54891 -3.02997 0.85675
C -7.30677 -1.83374 0.27992
C -6.45965 -1.07062 -0.73969
H -4.41664 4.53736 1.51408
H -5.04237 2.52494 0.22685
H -0.25019 3.51387 1.28225
H -2.05584 5.02972 2.04498
H -2.06568 -1.57848 -2.09827
H 0.36207 -2.02405 -2.21653
H 1.21210 1.48563 0.10973
H 2.85001 0.26138 0.56688
H 4.79267 -1.30696 -0.93845
H 6.02513 2.70536 0.02996
H 4.34802 2.23563 0.31581
H 5.63226 1.62539 1.37287
H 7.68772 1.03039 -0.93177
H 7.36935 -0.02024 0.45068
H 7.20391 -0.65090 -1.19414
H 4.19987 1.44662 -2.11716
H 5.88456 1.93318 -2.34737
H 5.38054 0.28159 -2.74118
H 5.29969 -3.00616 2.92368
H 6.92760 -3.32073 2.24887
H 6.58669 -1.78548 3.10363
H -4.91031 0.93500 -1.62346
H -4.05370 -0.53861 -2.03551
H -5.31859 0.05442 0.68176
H -4.10197 -2.52062 -0.40766
H -3.41641 -1.52192 0.87047
H -4.65218 -3.47445 1.80969
H -5.37861 -1.95600 2.31583
H -6.37579 -3.76588 0.05989
H -7.15524 -3.53129 1.61881
H -8.24275 -2.16280 -0.18394
H -7.58465 -1.15414 1.09655
H -6.27532 -1.71363 -1.61200
H -7.00786 -0.19509 -1.10641
66
mmff-M20
C 4.90951 3.51624 -0.33244
C 4.75333 2.31681 0.35056
C 3.50847 1.68836 0.28990
C 2.43377 2.26478 -0.42977
C 2.61787 3.46749 -1.11512
C 3.85861 4.08684 -1.06483
N 3.08316 0.50513 0.88280
C 1.74504 0.33199 0.59327
C 1.30008 1.39450 -0.23601
C 0.86187 -0.66887 1.00638
C -0.45529 -0.59573 0.58305
C -0.91297 0.44675 -0.24707
C -0.02233 1.43577 -0.66191
C -2.32146 0.55082 -0.73309
N -3.24722 -0.19035 -0.06081
O -2.62052 1.26201 -1.68914
C -4.66576 -0.09174 -0.33958
C -5.35140 0.35359 0.94491
C -5.26493 -1.41785 -0.91915
C -5.06351 -2.58454 0.05421
C -6.75997 -1.23427 -1.20662
C -4.54060 -1.72114 -2.23651
O -6.38985 1.15295 0.70905
O -4.98998 0.02378 2.05187
C -7.12132 1.60171 1.86438
C 3.87616 -0.33219 1.77597
C 4.65991 -1.48311 1.12317
C 5.74668 -1.00298 0.15450
C 6.55201 -2.16858 -0.42311
C 5.64487 -3.19872 -1.09428
C 4.56675 -3.68882 -0.12848
C 3.75914 -2.52564 0.45151
H 5.86977 4.02016 -0.29730
H 5.57913 1.89259 0.90792
H 1.80062 3.91349 -1.67245
H 4.01820 5.02209 -1.59029
H 1.18458 -1.49095 1.63237
H -1.12809 -1.39125 0.88410
H -0.38456 2.22878 -1.30610
H -3.00421 -0.56063 0.84617
H -4.79211 0.69167 -1.08909
H -5.46620 -3.50339 -0.38210
H -4.00255 -2.75423 0.25893
H -5.57292 -2.41490 1.00660
H -7.14802 -2.12663 -1.70641
H -7.34196 -1.08642 -0.29357
H -6.93729 -0.37711 -1.86332
H -3.46728 -1.85336 -2.07964
H -4.93426 -2.64468 -2.67141
H -4.68494 -0.91573 -2.96271
H -6.46588 2.16506 2.52948
H -7.91235 2.23918 1.47744
H -7.54348 0.75050 2.39997
H 4.57087 0.32448 2.30765
H 3.19680 -0.73810 2.53102
H 5.16622 -1.97933 1.96513
H 5.27937 -0.44584 -0.66770
H 6.42000 -0.30662 0.66651
H 7.29440 -1.79112 -1.13438
H 7.11333 -2.65497 0.38605
H 5.16563 -2.74259 -1.97103
H 6.23617 -4.04314 -1.46449
H 3.89514 -4.39356 -0.63028
H 5.04270 -4.24146 0.69275
H 3.18839 -2.04626 -0.35435
H 3.02885 -2.90786 1.17333
66
mmff-M21
C -3.71541 3.78280 1.03847
C -4.05635 2.67476 0.27339
C -3.03006 1.81919 -0.13020
C -1.68198 2.08048 0.21620
C -1.36555 3.19646 0.99415
C -2.38650 4.04251 1.40325
N -3.09108 0.66566 -0.90262
C -1.80510 0.20279 -1.09346
C -0.89476 1.04004 -0.39824
C -1.34517 -0.87399 -1.85753
C 0.01741 -1.10927 -1.89673
C 0.93390 -0.31478 -1.18175
C 0.47092 0.77201 -0.43960
C 2.37973 -0.67119 -1.29517
N 3.19168 -0.20031 -0.30664
O 2.80477 -1.34712 -2.22885
C 4.59919 -0.53786 -0.23985
C 4.90576 -1.33853 1.01998
C 5.53704 0.70644 -0.40489
C 5.31660 1.72107 0.72269
C 7.00228 0.25429 -0.41746
C 5.21127 1.36197 -1.75285
O 4.11905 -0.99055 2.04460
O 5.76649 -2.18178 1.09528
C 4.33409 -1.69168 3.28119
C -4.29381 0.11955 -1.52114
C -5.05444 -0.94698 -0.71576
C -5.61985 -0.41912 0.60767
C -6.42660 -1.48672 1.34971
C -5.60455 -2.75452 1.57762
C -5.04894 -3.29012 0.25872
C -4.24015 -2.22488 -0.48434
H -4.49886 4.46054 1.36110
H -5.08930 2.49072 0.00534
H -0.33614 3.40284 1.26853
H -2.15737 4.91480 2.00565
H -2.02431 -1.51249 -2.40830
H 0.40850 -1.92932 -2.48730
H 1.15880 1.43445 0.07547
H 2.76520 0.15339 0.53521
H 4.79454 -1.21052 -1.07757
H 5.95331 2.59613 0.56242
H 4.28045 2.07072 0.75192
H 5.56458 1.30447 1.70283
H 7.64784 1.11478 -0.61663
H 7.30873 -0.18118 0.53614
H 7.18104 -0.48877 -1.20085
H 4.17697 1.71253 -1.78773
H 5.86571 2.22396 -1.91250
H 5.36449 0.66251 -2.58008
H 5.34913 -1.52011 3.64141
H 3.60727 -1.28269 3.97862
H 4.17071 -2.76086 3.14113
H -4.96111 0.95977 -1.73350
H -4.00351 -0.29632 -2.49032
H -5.90930 -1.21336 -1.35581
H -4.79305 -0.08328 1.24682
H -6.25075 0.45678 0.42021
H -6.78413 -1.08574 2.30414
H -7.31990 -1.73697 0.76168
H -4.77114 -2.52848 2.25642
H -6.21314 -3.51890 2.07268
H -4.42583 -4.17312 0.43641
H -5.88228 -3.61846 -0.37695
H -3.34273 -1.98244 0.09948
H -3.89506 -2.62558 -1.44392
66
mmff-M22
C 3.53839 3.91631 0.64838
C 3.89488 2.77541 -0.05912
C 2.92538 1.78702 -0.23597
C 1.61454 1.94966 0.27464
C 1.28225 3.10310 0.98870
C 2.24898 4.08107 1.17439
N 3.01091 0.57200 -0.90511
C 1.76999 -0.03073 -0.87021
C 0.87088 0.78387 -0.13476
C 1.34141 -1.23315 -1.44056
C 0.02396 -1.61005 -1.25164
C -0.88342 -0.81850 -0.52169
C -0.45024 0.38166 0.04216
C -2.27454 -1.34059 -0.37030
N -3.22577 -0.42759 -0.02492
O -2.53728 -2.52829 -0.54336
C -4.62694 -0.77930 0.08586
C -5.45999 -0.01904 -0.93882
C -5.18250 -0.63127 1.54354
C -5.09096 0.82012 2.02802
C -6.64011 -1.10489 1.59399
C -4.33963 -1.52867 2.45851
O -4.96480 1.19784 -1.19141
O -6.45997 -0.45379 -1.45715
C -5.68474 1.99352 -2.14834
C 4.17601 0.08401 -1.63467
C 5.15234 -0.80999 -0.85194
C 4.52961 -2.13586 -0.40014
C 5.54572 -3.02953 0.31414
C 6.19778 -2.30741 1.49235
C 6.83031 -0.99083 1.04353
C 5.81645 -0.09427 0.32971
H 4.27816 4.69607 0.79650
H 4.89697 2.66717 -0.45491
H 0.28140 3.23569 1.38641
H 2.00687 4.98306 1.72580
H 2.01157 -1.86315 -2.01172
H -0.33803 -2.54129 -1.67100
H -1.11657 0.99711 0.63737
H -3.00401 0.55224 -0.10401
H -4.70237 -1.83335 -0.18920
H -5.44884 0.88887 3.05953
H -4.05936 1.18397 2.01449
H -5.69935 1.49457 1.41926
H -6.99710 -1.07805 2.62774
H -7.30056 -0.47425 0.99477
H -6.73546 -2.13324 1.23184
H -3.29105 -1.22154 2.46435
H -4.71503 -1.46734 3.48424
H -4.38892 -2.57471 2.14170
H -6.70298 2.17294 -1.80118
H -5.13587 2.92912 -2.22158
H -5.71063 1.48832 -3.11457
H 3.80924 -0.45931 -2.51030
H 4.71049 0.95863 -2.01655
H 5.94371 -1.04949 -1.57848
H 3.69158 -1.93060 0.27849
H 4.11506 -2.66625 -1.26468
H 5.05811 -3.95025 0.65178
H 6.32422 -3.33155 -0.39938
H 5.43667 -2.10154 2.25702
H 6.94949 -2.94970 1.96367
H 7.25728 -0.45946 1.90079
H 7.66434 -1.20546 0.36181
H 5.04436 0.21692 1.04516
H 6.31211 0.81873 -0.01827
66
mmff-M24
C 4.91819 3.46120 -0.29721
C 4.73178 2.25880 0.37284
C 3.47663 1.65362 0.29087
C 2.42172 2.25591 -0.43680
C 2.63616 3.46105 -1.10892
C 3.88713 4.05728 -1.03748
N 3.02253 0.47308 0.86754
C 1.68516 0.32709 0.56007
C 1.27008 1.40482 -0.26508
C 0.77863 -0.66075 0.95322
C -0.53173 -0.55934 0.51456
C -0.95985 0.49843 -0.31204
C -0.04574 1.47396 -0.70746
C -2.35951 0.63212 -0.81624
N -3.31144 -0.07815 -0.14761
O -2.62942 1.34348 -1.78105
C -4.72016 0.03025 -0.46836
C -5.49820 0.63024 0.69679
C -5.33807 -1.31766 -0.97419
C -5.25319 -2.40511 0.10279
C -6.80117 -1.09930 -1.37823
C -4.54586 -1.76033 -2.21082
O -4.97489 0.29414 1.88123
O -6.48162 1.31957 0.57276
C -5.64229 0.81347 3.04390
C 3.79003 -0.38803 1.76034
C 4.55940 -1.54646 1.10363
C 5.66434 -1.07627 0.15076
C 6.45420 -2.25023 -0.43125
C 5.53526 -3.25666 -1.12207
C 4.43900 -3.73714 -0.17203
C 3.64674 -2.56560 0.41231
H 5.88685 3.94728 -0.24543
H 5.54270 1.81467 0.93653
H 1.83418 3.92661 -1.67240
H 4.07024 4.99410 -1.55238
H 1.07824 -1.49456 1.57519
H -1.22212 -1.34551 0.80024
H -0.38465 2.27892 -1.34951
H -3.07574 -0.47234 0.74933
H -4.79528 0.75338 -1.28301
H -5.65565 -3.34453 -0.28744
H -4.21826 -2.59236 0.40405
H -5.82693 -2.14285 0.99594
H -7.20228 -2.02170 -1.80850
H -7.42898 -0.82543 -0.52750
H -6.89048 -0.31018 -2.13123
H -3.49496 -1.93813 -1.97030
H -4.96463 -2.69117 -2.60449
H -4.59412 -1.00611 -3.00200
H -6.66977 0.44990 3.08405
H -5.07399 0.44627 3.89488
H -5.63988 1.90391 3.02380
H 4.49104 0.25006 2.30614
H 3.09522 -0.78885 2.50397
H 5.04813 -2.06031 1.94538
H 5.21541 -0.50230 -0.67010
H 6.34496 -0.39752 0.67660
H 7.21023 -1.87889 -1.13129
H 6.99872 -2.75511 0.37807
H 5.07299 -2.78271 -1.99846
H 6.11482 -4.10776 -1.49553
H 3.75980 -4.42432 -0.68777
H 4.89674 -4.30686 0.64789
H 3.09268 -2.06775 -0.39400
H 2.90258 -2.94202 1.12293
66
mmff-M2
C -3.61807 3.96235 0.95530
C -3.98829 2.79482 0.30025
C -2.97579 1.91267 -0.07947
C -1.61417 2.19543 0.19287
C -1.26898 3.37482 0.85668
C -2.27471 4.25343 1.23432
N -3.06518 0.69791 -0.74416
C -1.79458 0.19449 -0.91054
C -0.85434 1.08916 -0.33774
C -1.37989 -0.97830 -1.54840
C -0.02286 -1.24337 -1.59583
C 0.92707 -0.38240 -1.01287
C 0.50501 0.79361 -0.39106
C 2.36329 -0.77579 -1.12880
N 3.22401 -0.17268 -0.26043
O 2.74118 -1.59060 -1.96681
C 4.62359 -0.53955 -0.18172
C 4.89067 -1.00562 1.24289
C 5.58128 0.61513 -0.63307
C 5.38856 1.86147 0.23788
C 7.03946 0.14707 -0.55422
C 5.24901 0.95486 -2.09137
O 5.79814 -1.97875 1.29179
O 4.33750 -0.54779 2.21713
C 6.14059 -2.46278 2.60317
C -4.28130 0.06037 -1.22353
C -4.77563 -1.10206 -0.35528
C -5.96803 -1.78713 -1.03276
C -6.49452 -2.95988 -0.20383
C -6.84586 -2.52821 1.22036
C -5.66029 -1.84139 1.89888
C -5.14274 -0.66678 1.06659
H -4.38910 4.66267 1.25908
H -5.03214 2.58479 0.09931
H -0.23003 3.60194 1.07253
H -2.02276 5.17380 1.74978
H -2.08962 -1.66369 -1.99641
H 0.33853 -2.13770 -2.08957
H 1.21948 1.49752 0.02222
H 2.84570 0.28721 0.55467
H 4.77302 -1.38463 -0.85639
H 6.04392 2.66353 -0.11429
H 4.35981 2.22918 0.18559
H 5.62979 1.66859 1.28669
H 7.69371 0.91950 -0.96897
H 7.35890 -0.04120 0.47363
H 7.19525 -0.77027 -1.13020
H 4.21543 1.29360 -2.19629
H 5.90425 1.75749 -2.44276
H 5.39417 0.08838 -2.74328
H 5.25717 -2.86874 3.09725
H 6.87982 -3.24310 2.44052
H 6.56081 -1.65733 3.20671
H -5.05455 0.82957 -1.29677

H -4.09695 -0.29287 -2.24364
H -3.96217 -1.83802 -0.28342
H -6.77185 -1.04997 -1.17001
H -5.68354 -2.13013 -2.03427
H -7.36813 -3.40165 -0.69491
H -5.72778 -3.74510 -0.16474
H -7.69485 -1.83205 1.18738
H -7.17292 -3.39234 1.80861
H -5.94161 -1.49501 2.89906
H -4.85017 -2.56994 2.03723
H -5.92134 0.10719 1.01154
H -4.27741 -0.20924 1.55725
66
mmff-M3
C 3.52402 4.04928 0.37503
C 3.88359 2.82824 -0.18148
C 2.89753 1.84563 -0.28127
C 1.57351 2.08002 0.16292
C 1.23870 3.31587 0.72066
C 2.21827 4.29396 0.82493
N 2.98674 0.56360 -0.80146
C 1.74896 -0.03472 -0.70955
C 0.83309 0.87274 -0.11495
C 1.34212 -1.31440 -1.09848
C 0.02160 -1.66735 -0.88699
C -0.90710 -0.77910 -0.31089
C -0.49192 0.49356 0.08246
C -2.30255 -1.27834 -0.12786
N -3.26118 -0.32747 0.06099
O -2.56314 -2.47881 -0.14017
C -4.67039 -0.65626 0.13517
C -5.37305 0.10373 -0.98145
C -5.28831 -0.37134 1.54614
C -5.14440 1.10774 1.92126
C -6.76945 -0.76838 1.55885
C -4.53620 -1.23252 2.56836
O -6.39381 -0.58242 -1.49154
O -5.03839 1.20192 -1.36448
C -7.14008 0.07069 -2.53446
C 4.19512 -0.02214 -1.35936
C 5.20474 -0.51389 -0.31586
C 6.49477 -0.96363 -1.01146
C 7.53610 -1.46211 -0.00805
C 6.97428 -2.57398 0.87858
C 5.68607 -2.13067 1.57258
C 4.64783 -1.63796 0.56288
H 4.27285 4.82940 0.46366
H 4.89718 2.65414 -0.52345
H 0.22821 3.51048 1.06486
H 1.97390 5.25850 1.25648
H 2.03102 -2.01993 -1.54716
H -0.32719 -2.65445 -1.16649
H -1.17596 1.18340 0.56547
H -3.04659 0.62919 -0.17900
H -4.76150 -1.72582 -0.06331
H -5.55567 1.27729 2.92072
H -4.09447 1.41359 1.94044
H -5.67792 1.76010 1.22474
H -7.16652 -0.66486 2.57291
H -7.37266 -0.13819 0.90079
H -6.90583 -1.80898 1.24891
H -3.47233 -0.98431 2.59271
H -4.94529 -1.06147 3.56857
H -4.63523 -2.29751 2.33879
H -6.48746 0.29980 -3.37759
H -7.90964 -0.63867 -2.82845
H -7.59089 0.99023 -2.15906
H 3.89695 -0.84803 -2.01052
H 4.66488 0.72807 -2.00426
H 5.45380 0.33388 0.33800
H 6.25615 -1.77119 -1.71806
H 6.90673 -0.13958 -1.60545
H 8.42837 -1.81139 -0.53851
H 7.85656 -0.62306 0.62383
H 6.76465 -3.45728 0.26026
H 7.71986 -2.88105 1.61989
H 5.26991 -2.95241 2.16504
H 5.91487 -1.32127 2.27880
H 4.34553 -2.47573 -0.08130
H 3.74641 -1.29944 1.08417
66
mmff-M4
C 3.50763 4.09169 0.02763
C 3.87192 2.83206 -0.43048
C 2.89251 1.83808 -0.44933
C 1.56884 2.10232 -0.01793
C 1.22916 3.37706 0.44075
C 2.20246 4.36608 0.46178
N 2.98318 0.51533 -0.85876
C 1.74923 -0.07632 -0.70712
C 0.83220 0.87294 -0.18690
C 1.35061 -1.38604 -0.99007
C 0.03277 -1.72814 -0.74431
C -0.89797 -0.79978 -0.23888
C -0.48979 0.50395 0.04625
C -2.29003 -1.28923 -0.00756
N -3.25394 -0.33097 0.09756
O -2.54290 -2.48740 0.08923
C -4.66088 -0.65928 0.20877
C -5.37326 -0.00892 -0.96917
C -5.27334 -0.24869 1.59083
C -5.12923 1.25825 1.83016
C -6.75415 -0.64333 1.64553
C -4.51609 -1.01427 2.68293
O -6.38811 -0.74786 -1.41311
O -5.05020 1.05388 -1.44957
C -7.14260 -0.19826 -2.50857
C 4.16761 -0.15125 -1.37651
C 4.87994 -1.06008 -0.36821
C 5.39906 -0.29828 0.85499
C 6.12955 -1.22618 1.82729
C 7.26694 -1.97791 1.13514
C 6.76269 -2.73588 -0.09340
C 6.02417 -1.80947 -1.06084
H 4.25350 4.87933 0.05097
H 4.88702 2.63836 -0.75624
H 0.21921 3.59138 0.77476
H 1.95455 5.36083 0.81572
H 2.04377 -2.11887 -1.38599
H -0.31202 -2.73653 -0.94053
H -1.17727 1.22707 0.47223
H -3.04587 0.60178 -0.22734
H -4.74660 -1.74297 0.11000
H -5.53723 1.51728 2.81159
H -4.07947 1.56494 1.81778
H -5.66557 1.84454 1.07921
H -7.14740 -0.44729 2.64734
H -7.36022 -0.07601 0.93488
H -6.89140 -1.70796 1.43290
H -3.45221 -0.76480 2.67987
H -4.92062 -0.75433 3.66569
H -4.61552 -2.09563 2.55007
H -6.49416 -0.04163 -3.37129
H -7.90702 -0.93758 -2.73415
H -7.59999 0.74802 -2.21695
H 3.86885 -0.73498 -2.25361
H 4.85351 0.62000 -1.73656
H 4.15396 -1.80699 -0.01673
H 6.08989 0.48751 0.51811
H 4.57206 0.20645 1.36507
H 6.51405 -0.65009 2.67567
H 5.41485 -1.95073 2.24014
H 8.03711 -1.25900 0.82445
H 7.74847 -2.66871 1.83560
H 7.59632 -3.22530 -0.60825
H 6.08207 -3.53511 0.22898
H 6.73222 -1.07655 -1.47301
H 5.63638 -2.38257 -1.91099
66
mmff-M5
C 4.93636 3.50304 0.76627
C 4.80795 2.28977 0.10180
C 3.52029 1.78254 -0.07809
C 2.38013 2.47747 0.39288
C 2.53697 3.69402 1.06027
C 3.81674 4.19954 1.24432
N 3.11831 0.61511 -0.70930
C 1.74327 0.54208 -0.66233
C 1.23601 1.68171 0.01704
C 0.88099 -0.42928 -1.17676
C -0.48113 -0.25636 -0.99301
C -1.00372 0.86008 -0.30971
C -0.13561 1.83406 0.18226
C -2.46554 1.08175 -0.09907
N -3.27033 -0.00605 -0.26429
O -2.91189 2.18692 0.19909
C -4.69049 0.03400 0.02017
C -4.96812 -1.00620 1.09669
C -5.57198 -0.16591 -1.25912
C -5.27452 -1.51259 -1.92772
C -7.05773 -0.08823 -0.88768
C -5.24481 0.97159 -2.23451
O -5.92914 -0.61947 1.93336
O -4.37899 -2.05972 1.18477
C -6.28342 -1.55387 2.96902
C 4.01908 -0.34286 -1.32980
C 4.72706 -1.28079 -0.34542
C 5.76644 -2.12745 -1.08911
C 6.50286 -3.07939 -0.14506
C 5.52854 -3.96507 0.63221
C 4.48791 -3.12503 1.37329
C 3.75250 -2.17901 0.42217
H 5.92703 3.91841 0.91879
H 5.68297 1.76276 -0.26050
H 1.67208 4.23700 1.42703
H 3.95519 5.14304 1.76099
H 1.25163 -1.29498 -1.71212
H -1.14756 -0.99715 -1.42090
H -0.55249 2.69763 0.68785
H -2.85011 -0.92369 -0.26892
H -4.90827 1.02235 0.42900
H -5.87878 -1.61686 -2.83371
H -4.22370 -1.58831 -2.22172
H -5.50755 -2.35513 -1.27114
H -7.66408 -0.12587 -1.79742
H -7.36824 -0.91809 -0.24804
H -7.29022 0.84575 -0.36700
H -4.19190 0.95569 -2.52644
H -5.84777 0.86644 -3.14137
H -5.46379 1.94814 -1.79270
H -5.41771 -1.76513 3.59773
H -7.06463 -1.06528 3.54591
H -6.65421 -2.48175 2.53173
H 3.44265 -0.92464 -2.05397
H 4.76255 0.21957 -1.90443
H 5.26165 -0.65921 0.38680
H 5.25821 -2.71138 -1.86958
H 6.48275 -1.47550 -1.60239
H 7.20895 -3.69537 -0.71210
H 7.10069 -2.49103 0.56376
H 5.01571 -4.63834 -0.06796
H 6.07441 -4.60263 1.33594
H 3.76871 -3.77326 1.88514
H 4.98733 -2.53532 2.15371
H 3.17288 -2.77202 -0.29950
H 3.03380 -1.56870 0.97889
66
mmff-M6
C 4.77737 3.71642 -0.33126
C 4.66741 2.46297 0.25759
C 3.41840 1.84088 0.22927
C 2.29742 2.46216 -0.37276
C 2.43542 3.72161 -0.95988
C 3.67732 4.34157 -0.93659
N 3.03882 0.61206 0.74775
C 1.69646 0.42663 0.49875
C 1.18921 1.55327 -0.20180
C 0.86537 -0.64666 0.82915
C -0.46860 -0.57617 0.46249
C -0.99228 0.53404 -0.23008
C -0.15079 1.59298 -0.56896
C -2.41963 0.63396 -0.65843
N -3.29341 -0.21533 -0.04743
O -2.77779 1.43282 -1.52050
C -4.72148 -0.15666 -0.28403
C -5.39472 0.11858 1.05344
C -5.27313 -1.44326 -0.98707
C -4.99509 -2.69335 -0.14483
C -6.78158 -1.30332 -1.22498
C -4.56747 -1.57417 -2.34255
O -6.47543 0.88571 0.92340
O -4.99127 -0.30666 2.11222
C -7.20003 1.17230 2.13330
C 3.92851 -0.30859 1.43716
C 4.84048 -1.12335 0.51234
C 4.05705 -2.01718 -0.45377
C 4.99101 -2.84056 -1.34239
C 5.96719 -3.67070 -0.50868
C 6.75086 -2.79008 0.46527
C 5.81728 -1.95921 1.34745
H 5.73801 4.22072 -0.32235
H 5.52722 1.99263 0.72032
H 1.58477 4.20928 -1.42465
H 3.80088 5.31969 -1.38862
H 1.24163 -1.52197 1.34443
H -1.10228 -1.42518 0.69412
H -0.56308 2.43320 -1.11584
H -3.00829 -0.67360 0.80530
H -4.90254 0.69329 -0.94447
H -5.36608 -3.58035 -0.66675
H -3.92287 -2.83272 0.02036
H -5.48819 -2.64763 0.82986
H -7.14004 -2.15673 -1.80797
H -7.34748 -1.27945 -0.29043
H -7.01422 -0.39217 -1.78454
H -3.48612 -1.67417 -2.22080
H -4.93163 -2.46372 -2.86516
H -4.76253 -0.70447 -2.97687
H -6.55681 1.69372 2.84302
H -8.02942 1.80702 1.83147
H -7.56853 0.24898 2.58209
H 4.53628 0.27262 2.13870
H 3.31223 -0.97911 2.04196
H 5.43110 -0.41606 -0.08701
H 3.42008 -2.69807 0.12833
H 3.38762 -1.41036 -1.07219
H 4.40385 -3.48966 -2.00069
H 5.55726 -2.16328 -1.99573
H 5.40513 -4.42469 0.05888
H 6.65492 -4.21906 -1.16137
H 7.40720 -3.40405 1.09119
H 7.40371 -2.11451 -0.10337
H 5.24313 -2.63102 2.00117
H 6.40158 -1.30581 2.00567
66
mmff-M74
C -3.92878 3.84322 1.04358
C -4.14321 2.68250 0.31107
C -3.03486 1.88652 0.01799
C -1.73371 2.24596 0.44590
C -1.54601 3.41640 1.18439
C -2.64685 4.20873 1.47986
N -2.97106 0.70154 -0.69884
C -1.65725 0.28916 -0.74928
C -0.84753 1.21763 -0.04373
C -1.09187 -0.82416 -1.37781
C 0.27790 -0.99410 -1.28784
C 1.09759 -0.09817 -0.57436
C 0.52764 1.01670 0.04097
C 2.56270 -0.38760 -0.55562
N 3.27754 0.17271 0.45212
O 3.08912 -1.09936 -1.40896
C 4.71903 0.05024 0.61271
C 5.41572 0.11133 -0.75145
C 5.16367 -1.10596 1.55583
C 4.88682 -2.49148 0.96298
C 6.65935 -0.95209 1.87036
C 4.38358 -0.96156 2.87204
O 5.00743 1.19390 -1.42437
O 6.28292 -0.62687 -1.14918
C 5.58807 1.37732 -2.72281
C -4.11257 0.03904 -1.30932
C -4.98424 -0.75701 -0.33131
C -4.22537 -1.90748 0.33684
C -5.12712 -2.70213 1.28317
C -6.37255 -3.22113 0.56398
C -7.13464 -2.08269 -0.11518
C -6.23015 -1.28229 -1.05377
H -4.77451 4.47902 1.28374
H -5.14028 2.41294 -0.01716
H -0.55513 3.70443 1.51997
H -2.51719 5.12089 2.05208
H -1.69623 -1.53973 -1.92223
H 0.75160 -1.83926 -1.77323
H 1.13884 1.75063 0.55629
H 2.78694 0.69106 1.16165
H 5.03296 0.98037 1.10453
H 5.17004 -3.26182 1.68769
H 3.82639 -2.61989 0.73329
H 5.45784 -2.65174 0.04786
H 6.95902 -1.70341 2.60720
H 7.27341 -1.08294 0.97910
H 6.87329 0.03379 2.29800
H 3.31155 -1.11963 2.72726
H 4.73432 -1.70686 3.59162
H 4.52952 0.02767 3.32034
H 5.35426 0.52665 -3.36485
H 5.13882 2.28527 -3.11880
H 6.67080 1.48981 -2.64668
H -4.71820 0.80374 -1.80713
H -3.73580 -0.61942 -2.09637
H -5.31877 -0.06965 0.45857
H -3.83994 -2.57936 -0.44309
H -3.35588 -1.52234 0.87959
H -4.56634 -3.53377 1.72287
H -5.43288 -2.05611 2.11707
H -6.07041 -3.95598 -0.19443
H -7.02622 -3.74838 1.26713
H -7.99318 -2.47617 -0.66978
H -7.54025 -1.41047 0.65267
H -5.91425 -1.92436 -1.88825
H -6.78736 -0.44754 -1.49453
66
mmff-M75
C -3.90883 3.94143 0.75250
C -4.13023 2.73190 0.10658
C -3.02881 1.90672 -0.12502
C -1.72630 2.28774 0.28289
C -1.53156 3.50764 0.93460
C -2.62587 4.32908 1.16600
N -2.96766 0.66810 -0.74743
C -1.65730 0.24568 -0.75583
C -0.84440 1.21875 -0.11962
C -1.10123 -0.91546 -1.30045
C 0.26719 -1.08887 -1.19331
C 1.09122 -0.14808 -0.54551
C 0.52894 1.01456 -0.01664
C 2.55467 -0.44550 -0.50307
N 3.27490 0.19126 0.45403
O 3.07494 -1.22928 -1.29482
C 4.71684 0.07823 0.61765
C 5.40754 0.03013 -0.75023
C 5.16388 -1.00004 1.64782
C 4.87866 -2.42757 1.17008
C 6.66214 -0.82736 1.93986
C 4.39277 -0.74704 2.95285
O 4.99669 1.05634 -1.50498
O 6.27228 -0.73829 -1.09218
C 5.57371 1.13880 -2.81534
C -4.08103 -0.06323 -1.33112
C -4.59893 -1.22518 -0.47607
C -5.66989 -2.00239 -1.25022
C -6.21692 -3.17721 -0.43777
C -6.74487 -2.72135 0.92294
C -5.68053 -1.94352 1.69746
C -5.14237 -0.76728 0.88077
H -4.75098 4.59869 0.94229
H -5.12999 2.44782 -0.19990
H -0.53941 3.80968 1.25378
H -2.49091 5.27982 1.67011
H -1.71209 -1.66084 -1.79590
H 0.73669 -1.97035 -1.61381
H 1.14485 1.78103 0.44249
H 2.79019 0.77466 1.11536
H 5.03438 1.04367 1.03301
H 5.16593 -3.13850 1.95168
H 3.81605 -2.57046 0.95988
H 5.44177 -2.66209 0.26618
H 6.96326 -1.51658 2.73454
H 7.27014 -1.03425 1.05891
H 6.88282 0.18946 2.28343
H 3.31946 -0.91314 2.82814
H 4.74644 -1.43234 3.72847
H 4.54372 0.27510 3.31800
H 5.34031 0.24039 -3.38887
H 5.12166 2.01231 -3.27969
H 6.65636 1.25919 -2.75090
H -4.88655 0.65116 -1.51983
H -3.76213 -0.43752 -2.30970
H -3.75754 -1.90769 -0.28950
H -6.49416 -1.31962 -1.50079
H -5.25914 -2.35974 -2.20162

H -7.00570 -3.68615 -1.00195
H -5.41613 -3.91331 -0.28663
H -7.62270 -2.07843 0.77255
H -7.08545 -3.58393 1.50576
H -6.08805 -1.58280 2.64787
H -4.85069 -2.61746 1.94926
H -5.95275 -0.04385 0.71322
H -4.36267 -0.24293 1.44294
66
mmff-M76
C 3.87168 3.88971 0.97555
C 4.08982 2.73364 0.23680
C 2.99655 1.89867 0.00114
C 1.70608 2.21596 0.49033
C 1.51434 3.38338 1.23254
C 2.60063 4.21361 1.47235
N 2.93875 0.70813 -0.70691
C 1.63885 0.25126 -0.69234
C 0.83143 1.15960 0.04139
C 1.08779 -0.89958 -1.26340
C -0.26666 -1.12322 -1.09390
C -1.08968 -0.22784 -0.38345
C -0.53065 0.91329 0.19248
C -2.53412 -0.58695 -0.25921
N -3.39066 0.43237 0.00087
O -2.92293 -1.74609 -0.38962
C -4.82286 0.27832 0.21008
C -5.11090 -0.99667 1.01119
C -5.68534 0.49087 -1.06870
C -5.48922 -0.62087 -2.10463
C -7.16511 0.58779 -0.66775
C -5.27267 1.83309 -1.69293
O -4.36642 -1.01479 2.12342
O -5.94891 -1.82786 0.76233
C -4.53993 -2.15738 2.97260
C 4.07286 0.07766 -1.36331
C 5.01757 -0.67551 -0.41983
C 6.24680 -1.16318 -1.19540
C 7.22340 -1.91933 -0.29320
C 6.53654 -3.07705 0.43208
C 5.30820 -2.59555 1.20475
C 4.33392 -1.84494 0.29504
H 4.70589 4.55491 1.17250
H 5.07837 2.49677 -0.13903
H 0.53146 3.63953 1.61433
H 2.46792 5.12326 2.04787
H 1.69266 -1.60635 -1.81869
H -0.72510 -2.01100 -1.51339
H -1.12984 1.59881 0.78314
H -3.02831 1.37058 0.03247
H -5.10006 1.08579 0.90041
H -6.07726 -0.39349 -2.99983
H -4.44153 -0.70434 -2.40304
H -5.81324 -1.58632 -1.71474
H -7.76821 0.83562 -1.54645
H -7.53252 -0.35190 -0.25472
H -7.32205 1.37858 0.07433
H -4.24122 1.81161 -2.05461
H -5.91881 2.05623 -2.54674
H -5.37070 2.65725 -0.97743
H -4.28219 -3.07089 2.43430
H -3.86101 -2.00641 3.80882
H -5.57066 -2.22070 3.32521
H 3.68240 -0.60260 -2.12470
H 4.62730 0.85666 -1.89736
H 5.36338 0.03250 0.34657
H 5.91618 -1.82559 -2.00799
H 6.75143 -0.31356 -1.66989
H 8.06894 -2.28743 -0.88403
H 7.63936 -1.22419 0.44824
H 6.22614 -3.83069 -0.30423
H 7.24153 -3.57246 1.10828
H 4.79982 -3.44204 1.67848
H 5.62906 -1.92968 2.01709
H 3.93674 -2.53935 -0.45881
H 3.47774 -1.48534 0.87521
66
mmff-M77
C 3.85938 3.97782 0.69990
C 4.08134 2.77580 0.04034
C 2.99385 1.91858 -0.13376
C 1.70413 2.26128 0.34308
C 1.50855 3.47499 1.00605
C 2.58930 4.32765 1.18144
N 2.93607 0.67904 -0.75450
C 1.63972 0.21893 -0.69520
C 0.83204 1.17099 -0.02170
C 1.09572 -0.97115 -1.18695
C -0.25655 -1.19179 -0.99513
C -1.08089 -0.25464 -0.34251
C -0.52785 0.92722 0.15192
C -2.52316 -0.61189 -0.18851
N -3.38623 0.42030 -0.01569
O -2.90403 -1.78030 -0.22207
C -4.81938 0.27573 0.19354
C -5.10963 -0.92700 1.09864
C -5.67077 0.37353 -1.10621
C -5.46174 -0.82241 -2.04117
C -7.15440 0.49903 -0.72811
C -5.25666 1.65887 -1.83985
O -4.37419 -0.84671 2.21401
O -5.94357 -1.77923 0.91570
C -4.55634 -1.90882 3.16032
C 4.04035 -0.02377 -1.38831
C 4.63279 -1.16647 -0.55570
C 5.22767 -0.68700 0.77174
C 5.84036 -1.84318 1.56424
C 6.88840 -2.59211 0.74063
C 6.30801 -3.06995 -0.59084
C 5.68698 -1.91559 -1.37908
H 4.69090 4.65924 0.84635
H 5.07119 2.52125 -0.31961
H 0.52612 3.74807 1.37719
H 2.45372 5.27352 1.69446
H 1.70464 -1.70582 -1.70043
H -0.71209 -2.10799 -1.35192
H -1.13112 1.64725 0.69538
H -3.03059 1.36038 -0.06644
H -5.10712 1.13770 0.80970
H -6.04502 -0.67636 -2.95624
H -4.41168 -0.92505 -2.32507
H -5.78261 -1.75303 -1.57196
H -7.75055 0.66538 -1.63041
H -7.52208 -0.40184 -0.23641
H -7.32090 1.35170 -0.06038
H -4.22257 1.60965 -2.19110
H -5.89710 1.80687 -2.71405
H -5.36144 2.54065 -1.19770
H -4.29342 -2.86685 2.70909
H -3.88587 -1.68294 3.98642
H -5.59063 -1.94079 3.50672
H 3.68527 -0.41202 -2.34880
H 4.81490 0.71243 -1.61852
H 3.82200 -1.87243 -0.32632
H 6.00642 0.06010 0.56303
H 4.46073 -0.18441 1.37014
H 6.28284 -1.46638 2.49251
H 5.04449 -2.54056 1.85829
H 7.73763 -1.92354 0.54483
H 7.28341 -3.44124 1.30863
H 7.08292 -3.55786 -1.19165
H 5.53800 -3.82882 -0.39808
H 6.47735 -1.21014 -1.67236
H 5.24054 -2.28933 -2.30784
66
mmff-M7
C 4.94547 3.53847 0.61171
C 4.81481 2.30248 -0.00843
C 3.52768 1.78674 -0.16624
C 2.38999 2.49813 0.28907
C 2.54909 3.73769 0.91229
C 3.82845 4.25182 1.07055
N 3.11804 0.59567 -0.74916
C 1.74459 0.52615 -0.68505
C 1.24233 1.68880 -0.04444
C 0.88258 -0.46381 -1.16305
C -0.47893 -0.28628 -0.97696
C -0.99764 0.85215 -0.32776
C -0.12843 1.84479 0.12443
C -2.45864 1.07902 -0.11455
N -3.26338 -0.01389 -0.24017
O -2.90400 2.19271 0.15147
C -4.68185 0.03436 0.05161
C -4.95240 -0.97668 1.15733
C -5.57120 -0.19964 -1.21660
C -5.28077 -1.56557 -1.84819
C -7.05460 -0.10740 -0.83918
C -5.24691 0.90871 -2.22597
O -5.91160 -0.57036 1.98667
O -4.35943 -2.02549 1.27207
C -6.25837 -1.47696 3.04928
C 3.97456 -0.41260 -1.35303
C 4.21682 -1.64900 -0.47980
C 4.92914 -1.31635 0.83467
C 5.18640 -2.57201 1.66972
C 5.97164 -3.61842 0.87849
C 5.27336 -3.95376 -0.43981
C 5.00826 -2.69711 -1.27064
H 5.93707 3.95836 0.74467
H 5.69066 1.76416 -0.35074
H 1.68497 4.29056 1.26588
H 3.96887 5.21330 1.55232
H 1.25440 -1.34371 -1.67439
H -1.14774 -1.04071 -1.37634
H -0.54455 2.72512 0.60088
H -2.84273 -0.93111 -0.21830
H -4.89721 1.03345 0.43494
H -5.89014 -1.69389 -2.74764
H -4.23176 -1.65234 -2.14547
H -5.51242 -2.38857 -1.16677
H -7.66620 -0.17034 -1.74399
H -7.36334 -0.91713 -0.17349
H -7.28189 0.84217 -0.34506
H -4.19619 0.88061 -2.52470
H -5.85666 0.78013 -3.12525
H -5.45922 1.89819 -1.81049
H -5.38960 -1.66680 3.68060
H -7.03972 -0.97570 3.61502
H -6.62656 -2.41829 2.63944
H 3.52098 -0.71431 -2.30309
H 4.92669 0.06357 -1.60154
H 3.23788 -2.08371 -0.23236
H 5.88915 -0.83237 0.60600
H 4.34146 -0.59430 1.41099
H 5.72203 -2.30669 2.58742
H 4.22476 -3.00069 1.98234
H 6.97668 -3.22968 0.66568
H 6.10751 -4.52554 1.47712
H 5.87257 -4.66452 -1.01885
H 4.31801 -4.45150 -0.22639
H 5.96718 -2.25916 -1.58216
H 4.47022 -2.95820 -2.18933
66
mmff-M8
C 4.75310 3.75624 -0.12390
C 4.64865 2.47111 0.39269
C 3.40631 1.83954 0.31938
C 2.28673 2.48556 -0.26118
C 2.41920 3.77688 -0.77606
C 3.65419 4.40608 -0.70470
N 3.02790 0.57915 0.76040
C 1.69094 0.40140 0.48397
C 1.18300 1.56134 -0.15698
C 0.86646 -0.69382 0.75315
C -0.46637 -0.61098 0.38361
C -0.99184 0.53261 -0.25062
C -0.15525 1.61332 -0.52887
C -2.42020 0.65136 -0.67169
N -3.29243 -0.22410 -0.09644
O -2.78050 1.48920 -1.49477
C -4.72260 -0.14627 -0.31564
C -5.38028 0.05856 1.04218
C -5.28784 -1.38981 -1.08206
C -4.99978 -2.68597 -0.31635
C -6.79937 -1.23451 -1.28883
C -4.60274 -1.44413 -2.45319
O -6.45765 0.83740 0.96638
O -4.96793 -0.42622 2.07158
C -7.16659 1.06218 2.19849
C 3.87428 -0.39453 1.43172
C 4.36071 -1.54062 0.53737
C 5.09765 -2.58488 1.38414
C 5.59967 -3.75285 0.53360
C 6.47333 -3.26979 -0.62445
C 5.74341 -2.22593 -1.47032
C 5.24836 -1.05886 -0.61406
H 5.70971 4.26611 -0.07711
H 5.51076 1.98407 0.83282
H 1.56847 4.28086 -1.22302
H 3.77350 5.40904 -1.09978
H 1.24713 -1.59048 1.22737
H -1.09805 -1.47322 0.56657
H -0.57101 2.48046 -1.02906
H -3.00353 -0.72117 0.73311
H -4.90627 0.73944 -0.92659
H -5.38140 -3.54135 -0.88159
H -3.92540 -2.83600 -0.17653
H -5.47708 -2.69466 0.66722
H -7.16810 -2.05198 -1.91529
H -7.35133 -1.26493 -0.34611
H -7.03854 -0.29190 -1.79049
H -3.51979 -1.55082 -2.35335
H -4.97531 -2.30289 -3.01954
H -4.80652 -0.53999 -3.03448
H -6.51127 1.53860 2.92848
H -7.99393 1.71893 1.94157
H -7.53783 0.11835 2.59985
H 4.72892 0.14061 1.85389
H 3.31214 -0.80019 2.27955
H 3.47611 -2.02600 0.10106
H 5.95234 -2.10333 1.88011
H 4.44027 -2.95284 2.18042
H 6.15373 -4.45987 1.16023
H 4.73726 -4.30109 0.13153
H 7.39419 -2.82698 -0.22141
H 6.78034 -4.11713 -1.24691
H 6.39808 -1.85403 -2.26563
H 4.88536 -2.69800 -1.96745
H 6.11397 -0.52587 -0.19590
H 4.70473 -0.33944 -1.23521
66
mmff-M95
C -4.32232 3.82867 0.65419
C -4.37298 2.60708 -0.00548
C -3.18507 1.88392 -0.12246
C -1.96651 2.37529 0.40522
C -1.94322 3.60509 1.06628
C -3.12393 4.32493 1.18804
N -2.96012 0.66011 -0.73459
C -1.62229 0.35152 -0.61929
C -0.96023 1.39085 0.08693
C -0.91928 -0.76567 -1.07639
C 0.44350 -0.82380 -0.83361
C 1.12065 0.20062 -0.14189
C 0.40575 1.30193 0.32782
C 2.58381 0.16369 0.15656
N 3.34167 -0.65097 -0.61940
O 3.07420 0.84134 1.05802
C 4.77116 -0.86269 -0.44615
C 5.12526 -0.92185 1.04448
C 5.66863 0.05589 -1.32612
C 5.60414 1.52653 -0.90119
C 7.11774 -0.45053 -1.26786
C 5.18006 -0.06628 -2.77803
O 4.33214 -1.80186 1.66749
O 6.04092 -0.35568 1.58875
C 4.56168 -1.96332 3.07388
C -3.98120 -0.13616 -1.39631
C -4.88603 -0.92848 -0.44569
C -4.11573 -1.96280 0.38055
C -5.04676 -2.75940 1.29642
C -6.17163 -3.42780 0.50553
C -6.94330 -2.40605 -0.33021
C -6.01063 -1.60374 -1.23919
H -5.23269 4.40966 0.75853
H -5.30705 2.23653 -0.41147
H -1.01636 3.99312 1.47587
H -3.12260 5.28154 1.69911
H -1.41406 -1.57810 -1.59444
H 0.97561 -1.71058 -1.16138
H 0.93412 2.07422 0.87515
H 2.90331 -1.13674 -1.38378
H 4.95287 -1.88764 -0.79525
H 6.21744 2.12961 -1.57871
H 4.58108 1.90745 -0.94503
H 5.97680 1.66158 0.11468
H 7.73698 0.12735 -1.96054
H 7.53859 -0.35050 -0.26719
H 7.18058 -1.50264 -1.56758
H 4.17004 0.33368 -2.90070
H 5.84323 0.49922 -3.43887
H 5.18209 -1.10834 -3.11704
H 4.40522 -1.01677 3.59371
H 3.83551 -2.70180 3.40606
H 5.57712 -2.31739 3.25879
H -4.58872 0.53864 -2.00863
H -3.47843 -0.81607 -2.08927
H -5.34814 -0.21563 0.25186
H -3.60441 -2.65483 -0.30354
H -3.33671 -1.47052 0.97189
H -4.47277 -3.50927 1.85109
H -5.48194 -2.08324 2.04450
H -5.74143 -4.18782 -0.16068
H -6.85205 -3.95499 1.18287
H -7.71053 -2.90614 -0.93086
H -7.47228 -1.71591 0.34049
H -5.56580 -2.27590 -1.98670
H -6.58101 -0.85081 -1.79535
66
mmff-M96
C -4.37209 3.71281 -1.03704
C -4.42020 2.54669 -0.28354
C -3.21886 1.87581 -0.05019
C -1.99015 2.36350 -0.55727
C -1.96959 3.53657 -1.31455
C -3.16325 4.20464 -1.55116
N -2.98933 0.71409 0.67126
C -1.63878 0.44211 0.64648
C -0.97251 1.44325 -0.10944
C -0.92511 -0.60026 1.24263
C 0.44790 -0.63878 1.06122
C 1.12575 0.33636 0.30230
C 0.40709 1.38585 -0.26898
C 2.60513 0.33346 0.09690
N 3.23499 -0.85781 0.25353
O 3.21652 1.35735 -0.20315
C 4.67387 -1.05283 0.15391
C 5.41682 0.12937 0.78710
C 5.16787 -1.49418 -1.25501
C 5.02187 -0.38777 -2.30482
C 6.63392 -1.94244 -1.15688
C 4.32787 -2.70634 -1.68690
O 4.94948 0.35383 2.02115
O 6.35494 0.72392 0.31646
C 5.56193 1.43693 2.73417
C -4.01859 -0.05672 1.35006
C -4.85177 -0.95574 0.42966
C -5.99250 -1.60069 1.22536
C -6.85529 -2.50761 0.34629
C -6.01324 -3.56872 -0.36295
C -4.87217 -2.93028 -1.15546
C -4.01115 -2.02861 -0.26905
H -5.29271 4.25275 -1.23232
H -5.36238 2.17851 0.10549
H -1.03498 3.92102 -1.70964
H -3.16412 5.11692 -2.13771
H -1.41586 -1.35994 1.83879
H 0.99994 -1.43143 1.55522
H 0.94271 2.14304 -0.83028
H 2.67990 -1.67688 0.43659
H 4.89659 -1.88950 0.82896
H 5.33565 -0.76833 -3.28236
H 3.98438 -0.05611 -2.38983
H 5.63867 0.47641 -2.05609
H 6.95991 -2.34578 -2.12035
H 7.29127 -1.11368 -0.89302
H 6.75542 -2.73419 -0.40919
H 3.27720 -2.44058 -1.83040
H 4.70444 -3.09551 -2.63718
H 4.38185 -3.51666 -0.95117
H 5.41435 2.37471 2.19623
H 5.06179 1.47188 3.69938
H 6.62995 1.25560 2.86479
H -3.53200 -0.65941 2.12153
H -4.67474 0.64704 1.87308
H -5.30076 -0.32011 -0.34667
H -5.56385 -2.19258 2.04651
H -6.61170 -0.82415 1.68916
H -7.63546 -2.98151 0.95149
H -7.37137 -1.89458 -0.40458
H -5.59292 -4.25500 0.38465
H -6.64353 -4.17386 -1.02342

H -4.24965 -3.70357 -1.61802
H -5.29117 -2.33385 -1.97697
H -3.51316 -2.64256 0.49474
H -3.21950 -1.56117 -0.86379
66
mmff-M97
C -4.35662 3.79177 -0.84330
C -4.41149 2.58918 -0.15040
C -3.21540 1.89839 0.04925
C -1.98465 2.40470 -0.43698
C -1.95724 3.61470 -1.13365
C -3.14583 4.30274 -1.33345
N -2.98740 0.69676 0.70527
C -1.63937 0.42056 0.65772
C -0.97005 1.45600 -0.04538
C -0.93337 -0.65559 1.20069
C 0.43957 -0.69295 1.01578
C 1.12139 0.31636 0.30680
C 0.40897 1.39881 -0.20906
C 2.60092 0.31758 0.10039
N 3.22762 -0.88143 0.19752
O 3.21549 1.35297 -0.14918
C 4.66559 -1.07407 0.08110
C 5.41456 0.07438 0.76711
C 5.15148 -1.44701 -1.35027
C 5.00457 -0.29026 -2.34433
C 6.61647 -1.90407 -1.28103
C 4.30548 -2.63399 -1.83685
O 4.95399 0.23990 2.01294
O 6.35228 0.68829 0.32112
C 5.57367 1.28493 2.77488
C -3.98334 -0.12898 1.36957
C -4.40659 -1.37494 0.58347
C -5.06513 -1.03590 -0.75716
C -5.50434 -2.29705 -1.50350
C -6.43275 -3.15875 -0.64723
C -5.78938 -3.49723 0.69794
C -5.34191 -2.23658 1.43967
H -5.27468 4.34560 -1.00965
H -5.35770 2.20852 0.21546
H -1.02030 4.01080 -1.51134
H -3.14162 5.24390 -1.87238
H -1.43039 -1.43893 1.76040
H 0.98809 -1.51259 1.46785
H 0.94983 2.18133 -0.72904
H 2.67152 -1.70763 0.34116
H 4.88996 -1.94309 0.71340
H 5.31508 -0.62333 -3.34008
H 3.96736 0.04652 -2.41066
H 5.62337 0.56006 -2.05571
H 6.93704 -2.26012 -2.26474
H 7.27762 -1.09148 -0.97915
H 6.73854 -2.73225 -0.57405
H 3.25504 -2.35835 -1.96225
H 4.67642 -2.97765 -2.80670
H 4.36006 -3.47927 -1.14159
H 5.42816 2.24824 2.28345
H 5.07737 1.27524 3.74266
H 6.64141 1.09309 2.89234
H -3.57952 -0.42579 2.34341
H -4.85424 0.49921 1.57396
H -3.50282 -1.96528 0.37551
H -5.94403 -0.40249 -0.57140
H -4.37940 -0.44976 -1.37771
H -5.99694 -2.02269 -2.44239
H -4.61607 -2.88248 -1.77646
H -7.37014 -2.61374 -0.47187
H -6.70017 -4.07685 -1.18133
H -6.48550 -4.06937 1.32049
H -4.91755 -4.14331 0.52923
H -6.22594 -1.64200 1.71053
H -4.84670 -2.50704 2.37946
66
mmff-M98
C -4.31361 3.88180 0.43749
C -4.36721 2.62853 -0.15892
C -3.18331 1.89356 -0.23592
C -1.96552 2.40673 0.27525
C -1.93931 3.66854 0.87308
C -3.11589 4.40036 0.95116
N -2.95653 0.63700 -0.77988
C -1.62156 0.33201 -0.63661
C -0.96050 1.40304 0.01945
C -0.92288 -0.80902 -1.03824
C 0.43876 -0.86083 -0.78557
C 1.11497 0.19286 -0.13863
C 0.40353 1.31969 0.27306
C 2.57602 0.16229 0.17150
N 3.33716 -0.67575 -0.57571
O 3.06087 0.86519 1.05629
C 4.76526 -0.88454 -0.38775
C 5.10939 -0.90601 1.10618
C 5.66994 0.01027 -1.28472
C 5.60637 1.49107 -0.89633
C 7.11754 -0.49763 -1.20525
C 5.19002 -0.14657 -2.73611
O 4.30785 -1.76526 1.74702
O 6.02419 -0.32981 1.64135
C 4.52735 -1.89085 3.15869
C -3.94228 -0.22217 -1.41646
C -4.44114 -1.37704 -0.54046
C -5.35355 -2.29642 -1.36059
C -5.87234 -3.46984 -0.52775
C -6.57566 -2.98942 0.74216
C -5.67074 -2.06902 1.56169
C -5.15974 -0.89571 0.72360
H -5.22238 4.47038 0.50785
H -5.30356 2.24328 -0.54474
H -1.01245 4.07080 1.26874
H -3.11234 5.38201 1.41223
H -1.41997 -1.64041 -1.52355
H 0.97097 -1.76290 -1.06839
H 0.93357 2.11518 0.78439
H 2.90262 -1.18093 -1.32972
H 4.94775 -1.91831 -0.70938
H 6.22593 2.07577 -1.58419
H 4.58470 1.87337 -0.95636
H 5.97248 1.64987 0.11847
H 7.74210 0.06146 -1.90851
H 7.53273 -0.37343 -0.20495
H 7.17970 -1.55703 -1.47825
H 4.18130 0.25142 -2.87471
H 5.85801 0.40191 -3.40638
H 5.19300 -1.19658 -3.04956
H 4.37046 -0.93062 3.65265
H 3.79644 -2.61809 3.50493
H 5.54032 -2.24317 3.35974
H -4.78289 0.40510 -1.72451
H -3.49842 -0.61990 -2.33525
H -3.56639 -1.96471 -0.22757
H -6.20613 -1.71170 -1.73419
H -4.81758 -2.66675 -2.24216
H -6.55005 -4.08434 -1.12990
H -5.02788 -4.11510 -0.25118
H -7.48792 -2.44400 0.46498
H -6.89474 -3.84578 1.34593
H -6.20431 -1.69473 2.44183
H -4.81374 -2.64408 1.93734
H -6.01037 -0.26452 0.43001
H -4.49224 -0.26725 1.32221
66
mmff-M9
C -3.47836 3.94251 0.99328
C -3.86917 2.79561 0.31369
C -2.87079 1.90333 -0.07986
C -1.50488 2.15408 0.19695
C -1.13904 3.31311 0.88496
C -2.13000 4.20138 1.28001
N -2.98657 0.70813 -0.77280
C -1.72615 0.18211 -0.95478
C -0.76821 1.04604 -0.36185
C -1.32963 -0.98521 -1.61396
C 0.02219 -1.27353 -1.66616
C 0.98808 -0.44258 -1.06595
C 0.58568 0.72703 -0.42029
C 2.41684 -0.85883 -1.19133
N 3.28646 -0.29983 -0.30281
O 2.78075 -1.65662 -2.05194
C 4.68657 -0.67001 -0.25862
C 5.03566 -1.31328 1.07791
C 5.64524 0.51411 -0.62533
C 5.50363 1.67061 0.37066
C 7.09687 0.02024 -0.64548
C 5.27037 1.00144 -2.03061
O 4.31228 -0.81159 2.08541
O 5.87615 -2.16816 1.22050
C 4.57195 -1.35633 3.39034
C -4.24078 0.13919 -1.23927
C -5.05978 -0.56881 -0.15396
C -4.33119 -1.77628 0.44367
C -5.18296 -2.48210 1.50033
C -6.54320 -2.89496 0.93736
C -7.27730 -1.69857 0.33102
C -6.42189 -0.98777 -0.71911
H -4.23583 4.65194 1.30968
H -4.91544 2.60868 0.10125
H -0.09653 3.51860 1.10526
H -1.86175 5.10602 1.81477
H -2.04866 -1.65259 -2.07362
H 0.36665 -2.16470 -2.17740
H 1.31073 1.41013 0.00946
H 2.91200 0.17031 0.50606
H 4.82320 -1.44901 -1.01148
H 6.15204 2.49905 0.07055
H 4.47841 2.05122 0.40124
H 5.78885 1.37542 1.38417
H 7.75224 0.82773 -0.98502
H 7.43956 -0.29671 0.34194
H 7.21903 -0.82331 -1.33199
H 4.24406 1.37484 -2.06404
H 5.93621 1.81659 -2.32893
H 5.36745 0.19805 -2.76694
H 5.60837 -1.17372 3.67671
H 3.89304 -0.83825 4.06313
H 4.37334 -2.42870 3.39708
H -4.83209 0.94800 -1.68141
H -4.01178 -0.55715 -2.05026
H -5.23956 0.15205 0.65619
H -4.09710 -2.48512 -0.36319
H -3.37518 -1.46469 0.87717
H -4.64895 -3.35656 1.88711
H -5.33359 -1.80599 2.35263
H -6.39530 -3.65854 0.16175
H -7.15430 -3.35811 1.71950
H -8.22543 -2.01848 -0.11418
H -7.53076 -0.98787 1.12892
H -6.26200 -1.66115 -1.57311
H -6.95209 -0.11040 -1.10725
66
pm6-M13
C -3.66771 3.93677 -0.28273
C -4.01415 2.65212 -0.68127
C -3.02427 1.66867 -0.64752
C -1.70350 1.97509 -0.23848
C -1.38169 3.27287 0.16528
C -2.36853 4.24804 0.14368
N -3.09697 0.32641 -0.99977
C -1.83929 -0.22594 -0.86578
C -0.94074 0.75975 -0.38232
C -1.39589 -1.52239 -1.14376
C -0.06326 -1.81711 -0.91806
C 0.84467 -0.85380 -0.43822
C 0.39685 0.43865 -0.16603
C 2.25393 -1.29703 -0.21448
N 3.20134 -0.32143 -0.23964
O 2.53532 -2.48094 -0.04367
C 4.58310 -0.55440 0.12442
C 4.91649 0.38644 1.27512
C 5.56903 -0.40494 -1.08278
C 5.47591 0.99404 -1.70287
C 7.00553 -0.66857 -0.61490
C 5.18825 -1.45415 -2.13456
O 5.72481 -0.18216 2.16879
O 4.49197 1.51546 1.36975
C 6.12145 0.64139 3.28018
C -4.27041 -0.34684 -1.54520
C -5.19590 -1.04357 -0.53367
C -5.84498 -0.07568 0.46218
C -6.81079 -0.79248 1.40805
C -6.12734 -1.94646 2.14017
C -5.48956 -2.91977 1.14984
C -4.52165 -2.20601 0.20385
H -4.42330 4.71522 -0.30181
H -5.02388 2.43271 -1.00520
H -0.37360 3.51650 0.48450
H -2.13488 5.26093 0.45311
H -2.06579 -2.28469 -1.52100
H 0.30993 -2.81610 -1.11065
H 1.06317 1.19359 0.23894
H 2.92033 0.63900 -0.35980
H 4.64658 -1.58214 0.48566
H 6.16598 1.06834 -2.54845
H 4.47032 1.19779 -2.08260
H 5.73682 1.77744 -0.98669
H 7.67759 -0.66522 -1.47796
H 7.36012 0.09434 0.08300
H 7.09268 -1.64281 -0.12436
H 4.16486 -1.30917 -2.48926
H 5.85833 -1.37433 -2.99580
H 5.27126 -2.46790 -1.73223
H 6.76279 0.01289 3.89302
H 6.66816 1.51679 2.92730
H 5.24456 0.96003 3.84497
H -4.84139 0.39843 -2.10641
H -3.91647 -1.07878 -2.27694
H -6.00149 -1.46943 -1.15105
H -5.06134 0.41661 1.05245
H -6.37711 0.71448 -0.07890
H -7.22762 -0.07740 2.12530
H -7.65792 -1.18423 0.82900
H -5.35036 -1.54463 2.80443
H -6.84586 -2.47042 2.77967
H -4.96549 -3.72005 1.68315
H -6.27967 -3.40289 0.55933
H -3.66989 -1.82401 0.78135
H -4.11811 -2.92267 -0.52011
66
pm6-M14
C 3.72284 3.89503 0.71817
C 4.07451 2.73379 0.04203
C 3.06237 1.82045 -0.25715
C 1.71675 2.07727 0.10208
C 1.38956 3.24839 0.78924
C 2.39704 4.15181 1.09611
N 3.13570 0.60488 -0.92655
C 1.85848 0.09456 -1.04005
C 0.94319 0.96744 -0.39748
C 1.40945 -1.05747 -1.69252
C 0.05307 -1.32884 -1.67458
C -0.86732 -0.49561 -1.01059
C -0.41546 0.66334 -0.37968
C -2.30756 -0.88967 -1.06544
N -3.10067 -0.40493 -0.07248
O -2.73824 -1.62988 -1.94683
C -4.54112 -0.55027 -0.07287
C -5.14005 0.84859 -0.00378
C -5.06377 -1.48828 1.06693
C -4.65761 -0.96079 2.44792
C -6.59091 -1.60232 0.98543
C -4.44986 -2.87705 0.85213
O -6.25171 0.95444 -0.73052
O -4.65950 1.75875 0.63229
C -6.90955 2.23395 -0.70042
C 4.33522 0.03923 -1.53365
C 5.15236 -0.93016 -0.66358
C 4.38336 -2.20183 -0.28770
C 5.24777 -3.17408 0.51792
C 5.83316 -2.50679 1.76169
C 6.60959 -1.24425 1.38995
C 5.74685 -0.26976 0.58537
H 4.49538 4.61764 0.95954
H 5.10496 2.55349 -0.23784
H 0.36218 3.45188 1.07318
H 2.15947 5.06683 1.62768
H 2.09200 -1.72554 -2.20250
H -0.32977 -2.20722 -2.18066
H -1.10669 1.35624 0.08915
H -2.70833 0.21575 0.61806
H -4.81405 -0.99947 -1.02928
H -5.02133 -1.64158 3.22315
H -3.57003 -0.90024 2.55011
H -5.07600 0.02908 2.64766
H -6.94231 -2.33574 1.71700
H -7.08887 -0.65389 1.20225
H -6.91335 -1.93493 -0.00595
H -3.35884 -2.84215 0.90215
H -4.80160 -3.56094 1.63036
H -4.73512 -3.29070 -0.11933
H -7.77638 2.13022 -1.34827
H -7.21916 2.47693 0.31686
H -6.24147 3.01016 -1.07533
H 4.02822 -0.46868 -2.45249
H 4.97007 0.87519 -1.84110
H 5.99240 -1.23194 -1.30756
H 3.50044 -1.92949 0.30501
H 4.01632 -2.69747 -1.19331
H 4.65684 -4.05288 0.79724
H 6.06774 -3.53743 -0.11610
H 5.01775 -2.24159 2.44813
H 6.48063 -3.20711 2.30026
H 6.98856 -0.74901 2.29032
H 7.48857 -1.52397 0.79366
H 4.93279 0.10071 1.22172
H 6.34515 0.60171 0.29712
66
pm6-M17
C 5.03079 -3.39205 -0.37132
C 4.84263 -2.19131 0.30123
C 3.57075 -1.61716 0.26869
C 2.50190 -2.24848 -0.41267
C 2.71803 -3.45131 -1.08837
C 3.98509 -4.01664 -1.06620
N 3.11197 -0.44525 0.85891
C 1.75985 -0.33334 0.60594
C 1.33769 -1.42497 -0.19685
C 0.84658 0.63451 1.03151
C -0.47824 0.49789 0.64935
C -0.91439 -0.57429 -0.15380
C 0.00646 -1.52888 -0.58303
C -2.32985 -0.74477 -0.60222
N -3.27764 -0.11258 0.14037
O -2.61621 -1.44337 -1.57205
C -4.67205 -0.05514 -0.24476
C -5.05917 1.41505 -0.33610
C -5.60721 -0.86677 0.71373
C -5.49723 -0.35837 2.15582
C -7.06023 -0.75691 0.23576
C -5.17515 -2.33720 0.65848
O -5.89964 1.65011 -1.34288
O -4.64700 2.27029 0.41398
C -6.34847 3.00933 -1.49056
C 3.89156 0.43591 1.72109
C 4.61124 1.60884 1.03484
C 3.65293 2.60006 0.36492
C 4.39736 3.78796 -0.24862
C 5.47783 3.33001 -1.22742
C 6.44167 2.35125 -0.55834
C 5.69922 1.16097 0.05221
H 6.01240 -3.85412 -0.35768
H 5.66489 -1.72478 0.82923
H 1.90522 -3.93895 -1.61651
H 4.16980 -4.95119 -1.58465
H 1.15091 1.47939 1.63601
H -1.17252 1.27185 0.95901
H -0.33865 -2.34496 -1.20756
H -2.99693 0.47618 0.90860
H -4.74496 -0.49713 -1.23979
H -6.14854 -0.95057 2.80532
H -4.47691 -0.45673 2.53816
H -5.79703 0.68898 2.24473
H -7.69579 -1.40617 0.84493
H -7.44834 0.26127 0.32244
H -7.16142 -1.07008 -0.80782
H -4.14137 -2.46123 0.99028
H -5.81410 -2.93529 1.31510
H -5.25942 -2.73759 -0.35586
H -5.49785 3.67125 -1.65690
H -7.00520 3.00582 -2.35691
H -6.89200 3.32769 -0.60003
H 3.21433 0.82399 2.48727
H 4.62530 -0.18435 2.24408
H 5.11152 2.14039 1.85864
H 3.08870 2.08311 -0.42214
H 2.92015 2.96190 1.09466
H 3.68592 4.45416 -0.74816
H 4.86359 4.37442 0.55456
H 5.00285 2.83929 -2.08763
H 6.02447 4.19337 -1.62182
H 7.18652 1.99527 -1.27807
H 6.99619 2.87437 0.23241
H 5.24111 0.57125 -0.75226
H 6.41211 0.50267 0.56114

66
pm6-M19
C 3.39416 4.07710 0.56611
C 3.81367 2.87163 0.01839
C 2.84002 1.91494 -0.27212
C 1.46802 2.16189 -0.01752
C 1.07300 3.38123 0.53746
C 2.04002 4.33372 0.82640
N 2.98092 0.64960 -0.82360
C 1.73300 0.07838 -0.93529
C 0.75621 0.97968 -0.43933
C 1.36823 -1.16389 -1.46259
C 0.02391 -1.49095 -1.47653
C -0.96049 -0.62420 -0.96372
C -0.58888 0.62026 -0.45382
C -2.37946 -1.08579 -1.03738
N -3.24038 -0.52332 -0.14666
O -2.73601 -1.94015 -1.84566
C -4.66631 -0.77009 -0.17516
C -5.43501 0.52261 -0.42504
C -5.18543 -1.54510 1.08353
C -4.96372 -0.73691 2.36746
C -6.67830 -1.85548 0.91893
C -4.41293 -2.86723 1.17384
O -4.83016 1.58485 0.11807
O -6.47732 0.58480 -1.03151
C -5.48829 2.85073 -0.05915
C 4.22363 0.02100 -1.24285
C 4.76225 -1.03333 -0.26923
C 5.10478 -0.45053 1.10519
C 5.66541 -1.51872 2.04565
C 6.88104 -2.21781 1.43621
C 6.55432 -2.79630 0.05899
C 5.98488 -1.72978 -0.87798
H 4.13440 4.83537 0.79908
H 4.86512 2.69011 -0.17020
H 0.02566 3.58182 0.73833
H 1.74920 5.28578 1.25683
H 2.10584 -1.85499 -1.85318
H -0.29946 -2.43983 -1.88809
H -1.33176 1.32873 -0.10176
H -2.89385 0.15766 0.50914
H -4.84782 -1.40291 -1.04615
H -5.30709 -1.31497 3.23040
H -3.90472 -0.51272 2.52774
H -5.51506 0.20715 2.35841
H -7.01966 -2.46427 1.76131
H -7.28811 -0.94981 0.89090
H -6.86673 -2.41786 -0.00083
H -3.34218 -2.69682 1.30978
H -4.77340 -3.44752 2.02832
H -4.55174 -3.46923 0.27118
H -6.47605 2.83165 0.40301
H -4.85139 3.58201 0.43241
H -5.58533 3.07996 -1.12097
H 4.05706 -0.43310 -2.22537
H 4.96490 0.81118 -1.38779
H 3.97962 -1.79232 -0.12812
H 5.85171 0.34569 0.97737
H 4.21938 0.01589 1.54949
H 5.92786 -1.06799 3.00868
H 4.88486 -2.26334 2.25174
H 7.70125 -1.49382 1.33808
H 7.24008 -3.00831 2.10397
H 7.44744 -3.24671 -0.38708
H 5.81965 -3.60475 0.17145
H 6.75864 -0.97653 -1.08352
H 5.71946 -2.17747 -1.84272
66
pm6-M1
C -3.48500 4.05356 -0.18575
C -3.87038 2.76499 -0.53348
C -2.89024 1.77154 -0.52870
C -1.54662 2.06122 -0.18842
C -1.18579 3.36474 0.15957
C -2.15943 4.35419 0.16039
N -3.00414 0.42617 -0.84340
C -1.76301 -0.15986 -0.72136
C -0.82005 0.82095 -0.31610
C -1.37423 -1.48619 -0.93074
C -0.04393 -1.81091 -0.73491
C 0.91175 -0.85127 -0.34833
C 0.51395 0.46809 -0.13107
C 2.31503 -1.32777 -0.15729
N 3.28844 -0.38124 -0.24118
O 2.56955 -2.51411 0.03793
C 4.67395 -0.64346 0.08691
C 5.06691 0.31184 1.20649
C 5.62616 -0.54553 -1.15213
C 5.54833 0.84101 -1.80160
C 7.06946 -0.83422 -0.72148
C 5.18793 -1.60844 -2.16701
O 5.88737 -0.25946 2.08730
O 4.67514 1.45356 1.28959
C 6.33984 0.57637 3.16773
C -4.23635 -0.22678 -1.25558
C -5.20724 -0.53687 -0.11072
C -4.62326 -1.51179 0.91618
C -5.62566 -1.82327 2.02904
C -6.94143 -2.35816 1.46296
C -7.53009 -1.39813 0.42855
C -6.52404 -1.07901 -0.67846
H -4.22882 4.84345 -0.18148
H -4.89876 2.54798 -0.79785
H -0.16010 3.60199 0.42254
H -1.89502 5.37113 0.42916
H -2.08372 -2.24756 -1.23177
H 0.29118 -2.83154 -0.87748
H 1.21610 1.22163 0.21100
H 3.03069 0.58394 -0.37391
H 4.72157 -1.66466 0.46850
H 6.21415 0.87886 -2.66877
H 4.53709 1.06131 -2.15614
H 5.84989 1.63365 -1.11203
H 7.71483 -0.86660 -1.60410
H 7.46327 -0.06494 -0.05217
H 7.14815 -1.79919 -0.21160
H 4.15807 -1.44575 -2.49429
H 5.83321 -1.56566 -3.04955
H 5.25814 -2.61437 -1.74313
H 5.48946 0.93185 3.75073
H 6.98177 -0.05657 3.77536
H 6.89967 1.42807 2.77925
H -4.72784 0.41808 -1.99197
H -3.96773 -1.14779 -1.77952
H -5.42869 0.40656 0.40809
H -4.35049 -2.44583 0.40489
H -3.70082 -1.10444 1.34298
H -5.19211 -2.54318 2.73132
H -5.82350 -0.90781 2.60264
H -6.76003 -3.33238 0.98915
H -7.66037 -2.53249 2.27069
H -8.44286 -1.81907 -0.00640
H -7.82325 -0.46477 0.92738
H -6.31526 -1.99227 -1.25357
H -6.95369 -0.35699 -1.38252
66
pm6-M21
C 3.42375 4.01119 0.75029
C 3.83315 2.82846 0.14750
C 2.84939 1.89624 -0.18568
C 1.47957 2.14244 0.07555
C 1.09495 3.33839 0.68579
C 2.07130 4.26659 1.02060
N 2.98469 0.66017 -0.79848
C 1.73286 0.10266 -0.94377
C 0.76116 0.98785 -0.40734
C 1.35549 -1.11177 -1.52438
C 0.00873 -1.42630 -1.55357
C -0.96964 -0.57543 -1.00394
C -0.58697 0.64107 -0.43881
C -2.39188 -1.02246 -1.09647
N -3.24816 -0.49226 -0.18171
O -2.75549 -1.83902 -1.94002
C -4.67514 -0.73153 -0.21574
C -5.43959 0.57393 -0.40436
C -5.19256 -1.56029 1.00909
C -4.96333 -0.81094 2.32695
C -6.68706 -1.85805 0.83650
C -4.42422 -2.88759 1.03761
O -4.82715 1.60919 0.18071
O -6.48504 0.66656 -1.00148
C -5.47984 2.88453 0.06024
C 4.24867 0.07931 -1.22185
C 5.07087 -0.54647 -0.08924
C 6.44057 -0.98511 -0.62012
C 7.29912 -1.61563 0.47780
C 6.57608 -2.77842 1.15816
C 5.20863 -2.34557 1.68759
C 4.35381 -1.72090 0.58332
H 4.16960 4.75209 1.01855
H 4.88224 2.64482 -0.05350
H 0.04927 3.54101 0.89318
H 1.78837 5.20011 1.49471
H 2.08525 -1.79533 -1.94135
H -0.32101 -2.35424 -2.00585
H -1.32317 1.33944 -0.05418
H -2.89628 0.15630 0.50341
H -4.86275 -1.32391 -1.11349
H -5.30580 -1.42574 3.16448
H -3.90292 -0.59793 2.49317
H -5.51121 0.13455 2.36221
H -7.02718 -2.50357 1.65160
H -7.29388 -0.95010 0.85239
H -6.88094 -2.37722 -0.10724
H -3.35252 -2.72677 1.17762
H -4.78396 -3.50431 1.86649
H -4.56766 -3.44823 0.10940
H -6.46621 2.85030 0.52447
H -4.83786 3.59109 0.58050
H -5.57908 3.15952 -0.99047
H 4.03333 -0.67024 -1.98797
H 4.83197 0.86589 -1.71197
H 5.23815 0.22805 0.67267
H 6.29265 -1.71462 -1.42900
H 6.96241 -0.12868 -1.06258
H 8.25307 -1.95257 0.05834
H 7.54037 -0.85127 1.22849
H 6.44048 -3.59266 0.43351
H 7.18881 -3.18228 1.97127
H 4.68290 -3.19897 2.12891
H 5.34758 -1.61326 2.49422
H 4.13247 -2.48419 -0.17604
H 3.39195 -1.39349 0.99138
66
pm6-M25
C -4.81012 3.67504 -0.26299
C -4.66648 2.40463 0.28057
C -3.39851 1.82274 0.24196
C -2.29205 2.50016 -0.32528
C -2.46402 3.77541 -0.86762
C -3.72479 4.35553 -0.83422
N -2.98548 0.58807 0.71928
C -1.63539 0.45422 0.47894
C -1.15719 1.62120 -0.17423
C -0.77384 -0.60347 0.77972
C 0.56208 -0.47507 0.43585
C 1.05810 0.67717 -0.20630
C 0.18554 1.71859 -0.51994
C 2.48731 0.84317 -0.60937
N 3.39238 0.06263 0.04052
O 2.82173 1.65880 -1.46620
C 4.79335 0.02440 -0.32033
C 5.19347 -1.36794 -0.79580
C 5.73651 0.56923 0.80561
C 5.64754 -0.29019 2.07231
C 7.18391 0.59464 0.29911
C 5.30184 2.00382 1.13084
O 4.51420 -2.33308 -0.16641
O 6.03500 -1.58160 -1.63503
C 4.82871 -3.68332 -0.54705
C -3.84906 -0.37598 1.38185
C -4.73746 -1.19198 0.43577
C -5.70480 -2.05865 1.25028
C -6.61576 -2.89334 0.34859
C -5.80775 -3.74454 -0.63141
C -4.84126 -2.88392 -1.44551
C -3.92993 -2.05550 -0.53799
H -5.78603 4.14885 -0.24520
H -5.51535 1.89122 0.71700
H -1.62482 4.30577 -1.30566
H -3.87474 5.34528 -1.25158
H -1.12778 -1.50991 1.25551
H 1.21734 -1.31452 0.64279
H 0.57623 2.59181 -1.02970
H 3.07077 -0.58674 0.73925
H 4.90245 0.68164 -1.18524
H 6.29161 0.13133 2.84963
H 4.62989 -0.31746 2.47370
H 5.97148 -1.31898 1.89342
H 7.82807 1.04887 1.05774
H 7.56639 -0.40687 0.09040
H 7.27262 1.18759 -0.61645
H 4.27809 2.03569 1.51152
H 5.96020 2.42422 1.89676
H 5.35822 2.64437 0.24595
H 5.87714 -3.90087 -0.33941
H 4.18126 -4.31486 0.05631
H 4.62742 -3.83238 -1.60854
H -3.21451 -1.04423 1.97015
H -4.47391 0.16928 2.09718
H -5.33658 -0.48608 -0.15666
H -5.12331 -2.72937 1.89864
H -6.30617 -1.42605 1.91341
H -7.26552 -3.52865 0.95990
H -7.27645 -2.22165 -0.21556
H -5.23572 -4.49568 -0.07009
H -6.47952 -4.29655 -1.29751
H -4.23672 -3.51216 -2.10829
H -5.41549 -2.20840 -2.09368
H -3.28393 -2.73246 0.03875
H -3.26814 -1.42624 -1.14215
66
pm6-M265
C -3.89269 3.95807 -0.49346
C -4.10954 2.60830 -0.74022
C -3.01148 1.75039 -0.66293
C -1.71761 2.23304 -0.34983
C -1.52711 3.59458 -0.10389
C -2.61812 4.44984 -0.17586
N -2.95217 0.38129 -0.87320
C -1.64797 -0.03188 -0.70970
C -0.83916 1.08854 -0.38375
C -1.09372 -1.31073 -0.81712
C 0.26527 -1.44919 -0.60022
C 1.08921 -0.34965 -0.29076
C 0.52712 0.92193 -0.17405
C 2.53921 -0.62960 -0.07035
N 3.38801 0.42201 -0.20503
O 2.94295 -1.75775 0.20437
C 4.83235 0.33722 -0.03727
C 5.36888 -0.97337 -0.62824
C 5.32952 0.69503 1.39323
C 4.90076 -0.33778 2.44069
C 6.85975 0.83050 1.37780
C 4.73512 2.06228 1.76465
O 4.95113 -1.10410 -1.89369
O 6.13737 -1.74029 -0.10238
C 5.38046 -2.29111 -2.57385
C -4.08914 -0.45163 -1.23150
C -5.01718 -0.80048 -0.06237
C -4.31902 -1.62721 1.02156
C -5.27647 -1.98868 2.15864
C -6.51516 -2.71692 1.63598
C -7.21703 -1.90411 0.54760
C -6.25731 -1.53501 -0.58478
H -4.73061 4.64523 -0.54700
H -5.10068 2.24326 -0.98297
H -0.54169 3.97953 0.13729
H -2.48636 5.50960 0.01342
H -1.69955 -2.17628 -1.05669
H 0.72728 -2.42714 -0.66558
H 1.12904 1.78071 0.10422
H 3.02734 1.27954 -0.58899
H 5.24220 1.10787 -0.70194
H 5.24036 -0.01688 3.43088
H 3.81327 -0.43908 2.47711
H 5.33040 -1.31746 2.22874
H 7.20647 1.18430 2.35344
H 7.34660 -0.12165 1.16629
H 7.18384 1.56064 0.62772
H 3.64567 2.02190 1.83976
H 5.12559 2.38339 2.73464
H 5.00085 2.82858 1.02790
H 6.46942 -2.32665 -2.63440
H 5.01539 -3.17788 -2.05327
H 4.94670 -2.23144 -3.56953
H -4.65540 0.07066 -2.01001
H -3.70307 -1.36615 -1.68951
H -5.35228 0.14128 0.39478
H -3.93165 -2.55112 0.56925
H -3.45486 -1.08206 1.41528
H -4.75800 -2.60339 2.90210
H -5.58693 -1.07014 2.67450
H -6.21395 -3.68907 1.22281
H -7.20782 -2.92771 2.45786
H -8.07091 -2.46064 0.14677
H -7.62295 -0.98400 0.98876
H -5.93785 -2.45057 -1.10250
H -6.77198 -0.91741 -1.32994
66
pm6-M28
C -4.80769 3.69766 -0.10550
C -4.66357 2.40898 0.39237
C -3.39865 1.82321 0.32480
C -2.29593 2.51784 -0.23137
C -2.46839 3.81163 -0.72801
C -3.72586 4.39524 -0.66251
N -2.98103 0.57005 0.75058
C -1.63531 0.44424 0.48807
C -1.16129 1.63180 -0.12777
C -0.77581 -0.62561 0.74895
C 0.55778 -0.48912 0.39808
C 1.05014 0.68263 -0.21043
C 0.17869 1.73716 -0.48196
C 2.47695 0.85672 -0.61937
N 3.38510 0.06378 0.01082
O 2.80612 1.68845 -1.46249
C 4.78434 0.03120 -0.35723
C 5.18163 -1.35361 -0.85656
C 5.73289 0.55756 0.77301
C 5.64731 -0.32064 2.02699
C 7.17837 0.58830 0.26134
C 5.30196 1.98781 1.12151
O 4.50537 -2.32837 -0.23881
O 6.01878 -1.55425 -1.70331
C 4.81763 -3.67254 -0.64217
C -3.80060 -0.44347 1.39566
C -4.23315 -1.59280 0.47808
C -5.11878 -1.12596 -0.68100
C -5.55890 -2.29749 -1.56086
C -6.26509 -3.37795 -0.74116
C -5.39352 -3.84652 0.42439
C -4.94649 -2.67374 1.29862
H -5.78241 4.17235 -0.06287
H -5.51296 1.88460 0.81393
H -1.63106 4.35260 -1.15659
H -3.87623 5.39939 -1.04372
H -1.12989 -1.54384 1.20195
H 1.21414 -1.33516 0.57197
H 0.56854 2.62627 -0.96415
H 3.06688 -0.59598 0.70141
H 4.88971 0.70213 -1.21203
H 6.29586 0.08753 2.80769
H 4.63136 -0.35131 2.43242
H 5.96780 -1.34743 1.83123
H 7.82592 1.03117 1.02379
H 7.55890 -0.41065 0.03733
H 7.26434 1.19381 -0.64622
H 4.27936 2.01584 1.50557
H 5.96328 2.39505 1.89198
H 5.35724 2.64182 0.24646
H 5.86698 -3.89354 -0.44301
H 4.17291 -4.31335 -0.04570
H 4.61121 -3.80481 -1.70490
H -3.23584 -0.84137 2.24547
H -4.67959 0.05441 1.81340
H -3.32564 -2.04133 0.04945
H -6.00913 -0.62936 -0.27008
H -4.59088 -0.37940 -1.28340
H -6.21351 -1.93757 -2.36172
H -4.67702 -2.73233 -2.05018
H -7.20710 -2.97307 -0.34720
H -6.53240 -4.22660 -1.37993
H -5.93236 -4.58113 1.03228
H -4.50610 -4.35866 0.02917
H -5.82543 -2.22915 1.78663
H -4.28960 -3.02954 2.10081
66
pm6-M2
C 3.49691 4.07669 0.65996
C 3.89860 2.88389 0.07259

C 2.91335 1.94219 -0.22765
C 1.54760 2.19130 0.05671
C 1.17061 3.39761 0.65132
C 2.14916 4.33524 0.94981
N 3.03591 0.69154 -0.81585
C 1.78221 0.13195 -0.92190
C 0.82041 1.02584 -0.38501
C 1.39970 -1.09287 -1.47666
C 0.05296 -1.41034 -1.47679
C -0.91632 -0.55160 -0.92337
C -0.52718 0.67560 -0.38555
C -2.34000 -1.00130 -0.98465
N -3.17661 -0.46970 -0.05346
O -2.71948 -1.82382 -1.81498
C -4.61017 -0.67038 -0.07495
C -5.26192 0.70547 -0.12184
C -5.12590 -1.54235 1.11931
C -4.76983 -0.90212 2.46604
C -6.64588 -1.71748 1.01530
C -4.45940 -2.91991 1.01883
O -6.35914 0.71485 -0.87757
O -4.83082 1.67739 0.45553
C -7.06494 1.96651 -0.95574
C 4.26728 0.06556 -1.27075
C 4.81465 -1.01600 -0.33252
C 5.17711 -0.47165 1.05250
C 5.74605 -1.56679 1.95628
C 6.95118 -2.25302 1.31239
C 6.60463 -2.79304 -0.07558
C 6.02674 -1.69972 -0.97579
H 4.24637 4.82325 0.90117
H 4.94550 2.70042 -0.13817
H 0.12823 3.59970 0.87524
H 1.87236 5.27708 1.41088
H 2.12572 -1.77800 -1.89826
H -0.28372 -2.34553 -1.90860
H -1.25879 1.37910 -0.00128
H -2.82440 0.21503 0.59704
H -4.84270 -1.19810 -1.00145
H -5.12921 -1.53676 3.28139
H -3.68750 -0.79699 2.58623
H -5.22479 0.08478 2.58360
H -6.98836 -2.40719 1.79220
H -7.18156 -0.77442 1.15034
H -6.93345 -2.13400 0.04514
H -3.37166 -2.84199 1.08832
H -4.80487 -3.55814 1.83762
H -4.70759 -3.41292 0.07456
H -6.41751 2.73883 -1.37252
H -7.91140 1.78205 -1.61252
H -7.40792 2.27068 0.03405
H 4.08180 -0.36325 -2.26120
H 5.01133 0.85441 -1.40856
H 4.03124 -1.77604 -0.20154
H 5.92504 0.32544 0.93665
H 4.29918 -0.01472 1.52082
H 6.02252 -1.14321 2.92768
H 4.96552 -2.31417 2.15229
H 7.77255 -1.52919 1.22315
H 7.31611 -3.06241 1.95382
H 7.49036 -3.23390 -0.54531
H 5.86878 -3.60205 0.02458
H 6.80047 -0.94364 -1.17086
H 5.74728 -2.12011 -1.94883
66
pm6-M3
C 3.51579 4.00844 0.85202
C 3.91002 2.84193 0.20883
C 2.91737 1.92129 -0.13008
C 1.55367 2.16316 0.16492
C 1.18435 3.34263 0.81537
C 2.16965 4.25915 1.15610
N 3.03720 0.70215 -0.77904
C 1.78125 0.15161 -0.91484
C 0.82274 1.02394 -0.33542
C 1.38905 -1.04549 -1.52103
C 0.04092 -1.35533 -1.53340
C -0.92431 -0.51712 -0.94261
C -0.52681 0.68175 -0.35060
C -2.35015 -0.95593 -1.02363
N -3.18695 -0.45396 -0.07608
O -2.73204 -1.74554 -1.88439
C -4.62189 -0.64274 -0.11152
C -5.26276 0.73904 -0.11349
C -5.15027 -1.55180 1.04901
C -4.79634 -0.96190 2.41905
C -6.67094 -1.71174 0.93158
C -4.49346 -2.92997 0.90362
O -6.35433 0.78452 -0.87610
O -4.82817 1.68611 0.50136
C -7.04962 2.04391 -0.91405
C 4.29089 0.13215 -1.24550
C 5.13613 -0.52605 -0.14883
C 6.49596 -0.94505 -0.71945
C 7.37677 -1.60679 0.34174
C 6.66906 -2.79161 0.99991
C 5.31144 -2.37850 1.56896
C 4.43415 -1.72205 0.50138
H 4.26878 4.74009 1.12569
H 4.95450 2.66172 -0.01782
H 0.14350 3.54170 1.04890
H 1.89864 5.17982 1.66128
H 2.10861 -1.71948 -1.97011
H -0.30006 -2.26966 -2.00468
H -1.25278 1.37193 0.06696
H -2.83363 0.20576 0.59916
H -4.85355 -1.13598 -1.05703
H -5.16335 -1.62270 3.20987
H -3.71392 -0.86815 2.54750
H -5.24562 0.02312 2.56954
H -7.02256 -2.42506 1.68268
H -7.20032 -0.76984 1.09635
H -6.95660 -2.09265 -0.05368
H -3.40555 -2.86267 0.98100
H -4.84777 -3.59384 1.69788
H -4.74065 -3.38762 -0.05851
H -6.39300 2.82557 -1.29777
H -7.89253 1.89023 -1.58321
H -7.39763 2.31473 0.08364
H 4.05821 -0.59523 -2.02774
H 4.86466 0.93188 -1.72554
H 5.31695 0.22531 0.63290
H 6.33373 -1.64981 -1.54731
H 7.00757 -0.07420 -1.14541
H 8.32299 -1.92853 -0.10622
H 7.63124 -0.86525 1.11073
H 6.52076 -3.58345 0.25326
H 7.29823 -3.21892 1.78811
H 4.79593 -3.24634 1.99386
H 5.46476 -1.67107 2.39496
H 4.19895 -2.46206 -0.27665
H 3.48006 -1.40909 0.93805
66
pm6-M41
C -3.53596 3.89889 -0.57773
C -3.89822 2.59642 -0.89641
C -2.92617 1.60142 -0.78129
C -1.60706 1.91290 -0.37085
C -1.26913 3.22916 -0.04846
C -2.23851 4.21669 -0.15085
N -3.01612 0.24021 -1.04619
C -1.77022 -0.32204 -0.85550
C -0.86293 0.67880 -0.42220
C -1.34382 -1.64039 -1.04190
C -0.01915 -1.93966 -0.77837
C 0.89726 -0.96157 -0.34681
C 0.46551 0.35191 -0.16346
C 2.29616 -1.41086 -0.07603
N 3.25575 -0.44727 -0.11573
O 2.55921 -2.59009 0.14935
C 4.63280 -0.69826 0.25188
C 5.02941 0.13021 1.46900
C 5.63088 -0.52828 -0.94344
C 5.62825 0.91111 -1.47180
C 7.04612 -0.91102 -0.49333
C 5.19269 -1.48275 -2.06138
O 4.40874 1.31464 1.49986
O 5.82130 -0.23123 2.30591
C 4.72513 2.16907 2.61194
C -4.19140 -0.44918 -1.56704
C -5.14900 -1.05664 -0.52826
C -5.81125 -0.00880 0.37350
C -6.80959 -0.64177 1.34490
C -6.15884 -1.74277 2.18119
C -5.50587 -2.79498 1.28597
C -4.50636 -2.16473 0.31385
H -4.27758 4.68648 -0.66087
H -4.90622 2.37231 -1.22227
H -0.26199 3.47692 0.27061
H -1.99228 5.24396 0.09483
H -2.02039 -2.41565 -1.37867
H 0.34129 -2.95415 -0.90156
H 1.13676 1.12199 0.20287
H 2.99029 0.50223 -0.31937
H 4.67749 -1.74171 0.56988
H 6.30984 0.99216 -2.32360
H 4.63604 1.21344 -1.82048
H 5.95645 1.62708 -0.71351
H 7.72551 -0.86810 -1.34968
H 7.43191 -0.23725 0.27487
H 7.07263 -1.92935 -0.09329
H 4.19259 -1.23820 -2.42746
H 5.88763 -1.40926 -2.90307
H 5.18650 -2.52057 -1.71566
H 5.78761 2.41568 2.60976
H 4.12549 3.06503 2.47139
H 4.46519 1.67804 3.55045
H -4.73763 0.26407 -2.19117
H -3.83696 -1.23718 -2.23776
H -5.94419 -1.52117 -1.13107
H -5.03703 0.51883 0.94532
H -6.31983 0.74317 -0.23991
H -7.23681 0.12996 1.99410
H -7.64573 -1.06849 0.77467
H -5.39533 -1.29836 2.83385
H -6.89992 -2.20957 2.83894
H -5.00422 -3.55608 1.89305
H -6.28546 -3.31555 0.71362
H -3.66572 -1.74654 0.88253
H -4.09181 -2.93864 -0.34155
66
pm6-M44
C 3.63652 3.89134 0.59069
C 4.00181 2.70567 -0.03381
C 2.99592 1.78110 -0.31927
C 1.64332 2.05024 0.00281
C 1.30228 3.24624 0.63844
C 2.30328 4.16142 0.93130
N 3.08273 0.54149 -0.94130
C 1.80803 0.02615 -1.06104
C 0.88006 0.92171 -0.46988
C 1.37213 -1.15007 -1.67839
C 0.01575 -1.42217 -1.67682
C -0.91749 -0.56566 -1.06224
C -0.47855 0.61701 -0.46734
C -2.35632 -0.96189 -1.12879
N -3.16755 -0.43791 -0.17050
O -2.77171 -1.73257 -1.99136
C -4.60343 -0.61982 -0.17655
C -5.31877 0.72093 -0.29950
C -5.12402 -1.47139 1.03107
C -4.83533 -0.77872 2.36818
C -6.63264 -1.70546 0.88527
C -4.40756 -2.82714 0.99431
O -4.65031 1.70910 0.30534
O -6.37383 0.87679 -0.86576
C -5.25253 3.01323 0.24651
C 4.29404 -0.04502 -1.50374
C 5.09513 -0.98003 -0.58259
C 4.31805 -2.23550 -0.17069
C 5.16711 -3.17617 0.68694
C 5.73210 -2.46127 1.91357
C 6.51677 -1.21490 1.50627
C 5.66945 -0.27180 0.64955
H 4.40406 4.62299 0.82048
H 5.03779 2.51556 -0.28518
H 0.26934 3.45961 0.89351
H 2.05497 5.09576 1.42284
H 2.06473 -1.83620 -2.14932
H -0.35660 -2.31954 -2.15677
H -1.17976 1.32541 -0.03825
H -2.77413 0.17312 0.52648
H -4.83625 -1.17136 -1.08960
H -5.18061 -1.41017 3.19206
H -3.76405 -0.61061 2.51507
H -5.34654 0.18426 2.45047
H -6.97845 -2.36666 1.68526
H -7.20299 -0.77614 0.94831
H -6.86877 -2.18228 -0.07109
H -3.32751 -2.71231 1.11380
H -4.77204 -3.45928 1.80937
H -4.59399 -3.34790 0.05061
H -6.23040 3.00075 0.72940
H -4.57233 3.67303 0.77946
H -5.36051 3.33214 -0.79087
H 4.00476 -0.58755 -2.40843
H 4.93409 0.77955 -1.83060
H 5.94558 -1.30720 -1.19995
H 3.42550 -1.93951 0.39566
H 3.96594 -2.76584 -1.06243
H 4.57026 -4.04296 0.99018
H 5.99731 -3.56458 0.08175
H 4.90548 -2.16884 2.57510
H 6.36921 -3.14058 2.49009
H 6.88108 -0.68521 2.39298
H 7.40532 -1.51855 0.93649
H 4.84541 0.12423 1.25701
H 6.27415 0.58701 0.33752
66
pm6-M47
C -5.18912 3.24405 0.04854
C -4.94933 1.97967 -0.47417
C -3.63146 1.52018 -0.49266
C -2.57078 2.32353 -0.00811
C -2.83832 3.58822 0.52035
C -4.14941 4.04185 0.54763
N -3.11920 0.31813 -0.96635
C -1.74601 0.34937 -0.83248
C -1.35874 1.57264 -0.22541
C -0.78690 -0.58713 -1.22614
C 0.54588 -0.30553 -0.97276
C 0.94635 0.88835 -0.34105
C -0.01455 1.83368 0.01395
C 2.37354 1.22558 -0.05611
N 3.23145 0.17402 0.03857
O 2.73945 2.38766 0.10651
C 4.65973 0.34244 0.20078
C 5.41379 -0.26039 -0.97908
C 5.19081 -0.17396 1.58146
C 4.96155 -1.68183 1.73903
C 6.68709 0.13761 1.70819
C 4.43491 0.57433 2.68676
O 4.80054 -1.34120 -1.47400
O 6.45263 0.17575 -1.41348
C 5.44428 -1.98238 -2.58819
C -3.89007 -0.73631 -1.61518
C -4.40731 -1.86494 -0.70779
C -5.38859 -1.38028 0.36540
C -5.93306 -2.53793 1.20516
C -4.80555 -3.35897 1.82955
C -3.83237 -3.85308 0.76017
C -3.28414 -2.69599 -0.07724
H -6.20684 3.61952 0.07092
H -5.76747 1.37910 -0.85189
H -2.03173 4.20850 0.89731
H -4.37428 5.02240 0.95291
H -1.06115 -1.50845 -1.72407
H 1.28701 -1.02216 -1.31080
H 0.30859 2.76260 0.46986
H 2.87946 -0.76099 -0.08667
H 4.84548 1.41760 0.16049
H 5.31463 -2.00743 2.72192
H 3.90001 -1.93945 1.67412
H 5.50030 -2.26015 0.98359
H 7.03712 -0.15176 2.70342
H 7.28583 -0.40303 0.97186
H 6.88091 1.20720 1.58116
H 3.36213 0.37038 2.64680
H 4.80279 0.25525 3.66640
H 4.58113 1.65520 2.60446
H 6.43599 -2.33420 -2.30115
H 4.80396 -2.82037 -2.85241
H 5.53028 -1.28781 -3.42467
H -4.73516 -0.25840 -2.11869
H -3.26312 -1.16328 -2.40346
H -4.96391 -2.52762 -1.38785
H -4.88011 -0.66470 1.02438
H -6.21944 -0.84240 -0.10440
H -6.60043 -2.15103 1.98262
H -6.54303 -3.19064 0.56619
H -4.26237 -2.73661 2.55346
H -5.21677 -4.20515 2.39046
H -3.00510 -4.40372 1.22056
H -4.35072 -4.56164 0.10016
H -2.66919 -2.04943 0.56205
H -2.62496 -3.08647 -0.86058
66
pm6-M4
C -4.87172 3.61717 -0.45654
C -4.73787 2.37220 0.14524
C -3.46855 1.79205 0.15910
C -2.35121 2.44679 -0.41342
C -2.51340 3.69664 -1.01473
C -3.77542 4.27465 -1.03372
N -3.06398 0.58033 0.69850
C -1.70879 0.43945 0.49307
C -1.21855 1.57803 -0.19999
C -0.85181 -0.60164 0.85818
C 0.49109 -0.48472 0.53821
C 0.99873 0.63916 -0.14395
C 0.13110 1.66356 -0.52092
C 2.43594 0.79378 -0.52370
N 3.32991 0.04422 0.17527
O 2.78646 1.57907 -1.40193
C 4.73337 -0.03439 -0.17163
C 5.06203 -1.50199 -0.41223
C 5.66777 0.62557 0.89763
C 5.48168 -0.02586 2.27289
C 7.13120 0.49589 0.45787
C 5.30482 2.11296 0.98587
O 5.92762 -1.66391 -1.41210
O 4.58812 -2.41430 0.22578
C 6.32364 -3.01801 -1.69528
C -3.94129 -0.35922 1.37810
C -4.80567 -1.21334 0.44338
C -5.78603 -2.05611 1.26726
C -6.67332 -2.92743 0.37655
C -5.84050 -3.80770 -0.55591
C -4.86114 -2.97056 -1.37894
C -3.97329 -2.10615 -0.48186
H -5.84847 4.08900 -0.48026
H -5.59520 1.87665 0.58574
H -1.66579 4.20936 -1.45753
H -3.91785 5.24483 -1.49716
H -1.21378 -1.48730 1.36596
H 1.14219 -1.31290 0.79737
H 0.53140 2.51459 -1.05995
H 2.99780 -0.61368 0.86257
H 4.85941 0.50874 -1.10966
H 6.13887 0.45837 3.00116
H 4.45492 0.08642 2.63340
H 5.72476 -1.09144 2.25769
H 7.77187 1.04819 1.15146
H 7.47035 -0.54324 0.45105
H 7.28481 0.90879 -0.54377
H 4.26401 2.25169 1.28855
H 5.94042 2.60524 1.72804
H 5.45210 2.61485 0.02528
H 5.45285 -3.61667 -1.96497
H 7.01425 -2.94968 -2.53211
H 6.81661 -3.45820 -0.82756
H -3.31966 -1.00259 2.00647
H -4.58363 0.21157 2.05709
H -5.39525 -0.53214 -0.18635
H -5.21525 -2.70006 1.95125
H -6.40467 -1.40389 1.89454
H -7.33250 -3.54473 0.99615
H -7.32551 -2.27988 -0.22454
H -5.27691 -4.53572 0.04312
H -6.49536 -4.38624 -1.21632
H -4.23970 -3.61806 -2.00661
H -5.42477 -2.32111 -2.06211
H -3.33666 -2.75900 0.13193
H -3.30161 -1.49496 -1.09356
66
pm6-M5
C -4.97273 3.55785 0.16763
C -4.81824 2.26821 -0.32524
C -3.52528 1.74562 -0.37988
C -2.40543 2.50000 0.04645
C -2.58841 3.79309 0.54086
C -3.87358 4.31465 0.59945

N -3.09859 0.50689 -0.83405
C -1.72710 0.44503 -0.71549
C -1.24784 1.66547 -0.16949
C -0.84493 -0.58242 -1.05820
C 0.50767 -0.38369 -0.83395
C 1.00212 0.81394 -0.27974
C 0.11531 1.84208 0.03756
C 2.45412 1.06811 -0.03444
N 3.24669 -0.03094 0.08422
O 2.89573 2.20926 0.08127
C 4.69011 0.04401 0.16661
C 5.25613 -0.76821 -0.99107
C 5.25050 -0.41342 1.55517
C 4.84136 -1.85726 1.86917
C 6.77968 -0.29692 1.55993
C 4.67706 0.52239 2.62634
O 6.34660 -0.20639 -1.51124
O 4.76910 -1.79947 -1.39502
C 6.97146 -0.91812 -2.59457
C -3.97254 -0.53215 -1.35474
C -4.72339 -1.32842 -0.28117
C -3.78099 -2.07818 0.66529
C -4.55536 -2.88820 1.70643
C -5.53773 -3.85464 1.04446
C -6.47965 -3.11921 0.09046
C -5.70549 -2.30094 -0.94453
H -5.96809 3.98647 0.22020
H -5.67777 1.69546 -0.65338
H -1.73905 4.38167 0.87180
H -4.03232 5.31731 0.98126
H -1.19215 -1.51147 -1.49360
H 1.18837 -1.17303 -1.13485
H 0.51063 2.76664 0.44267
H 2.84852 -0.94708 -0.04876
H 4.96135 1.09107 0.02260
H 5.23640 -2.14705 2.84727
H 3.75351 -1.96586 1.91081
H 5.22777 -2.56234 1.12874
H 7.16047 -0.51940 2.56095
H 7.25004 -0.99650 0.86412
H 7.10317 0.71398 1.29378
H 3.58555 0.47657 2.65210
H 5.05035 0.22879 3.61203
H 4.97234 1.56008 2.44632
H 6.27722 -1.01771 -3.42978
H 7.82967 -0.31549 -2.88129
H 7.29155 -1.90708 -2.26407
H -4.68884 -0.05942 -2.03498
H -3.36471 -1.20633 -1.96396
H -5.30841 -0.61720 0.31898
H -3.15139 -2.75854 0.07458
H -3.10524 -1.37442 1.16218
H -3.85709 -3.43486 2.34910
H -5.10838 -2.20045 2.36017
H -4.97456 -4.61230 0.48292
H -6.11331 -4.39307 1.80505
H -7.14168 -3.83011 -0.41534
H -7.12688 -2.44662 0.66893
H -5.14462 -2.98231 -1.59994
H -6.40146 -1.75013 -1.58781

8 Bibliography

[1] Sheldrick, G. M. *Acta Cryst. A* **2015**, *71*, 3–8.

[2] Sheldrick, G. M. *Acta Cryst. C* **2015**, *71*, 3–8.
